# Comparative functional genomics analysis of cytochrome P450 gene superfamily in wheat and maize

**DOI:** 10.1186/s12870-020-2288-7

**Published:** 2020-03-02

**Authors:** Yixuan Li, Kaifa Wei

**Affiliations:** School of Biological Sciences and Biotechnology, Minnan Normal University, 36 Xian-Qian-Zhi Street, Zhangzhou, 363000 Fujian China

**Keywords:** Cytochrome P450, Gramineae crops, Expression regulation, Growth and development, Drought stress

## Abstract

**Background:**

The cytochrome P450s (CYP450s) as the largest enzyme family of plant metabolism participate in various physiological processes, whereas no study has demonstrated interest in comprehensive comparison of the genes in wheat and maize. Genome-wide survey, characterization and comparison of wheat and maize CYP450 gene superfamily are useful for genetic manipulation of the Gramineae crops.

**Results:**

In total, 1285 and 263 full-length *CYP450*s were identified in wheat and maize, respectively. According to standard nomenclature, wheat *CYP450*s (*TaCYP450*s) were categorized into 45 families, while maize *CYP450*s (*ZmCYP450*s) into 43 families. A comprehensive analysis of wheat and maize CYP450s, involved in functional domains, conserved motifs, phylogeny, gene structures, chromosome locations and duplicated events was performed. The result showed that each family/subfamily in both species exhibited characteristic features, suggesting their phylogenetic relationship and the potential divergence in their functions. Functional divergence analysis at the amino acid level of representative clans CYP51, CYP74 and CYP97 in wheat, maize and rice identified some critical amino acid sites that are responsible for functional divergence of a gene family. Expression profiles of *Ta*-, *ZmCYP450*s were investigated using RNA-seq data, which contribute to infer the potential functions of the genes during development and stress responses. We found in both species *CYP450*s had preferential expression in specific tissues, and many tissue-specific genes were identified. Under water-deficit condition, 82 and 39 significantly differentially expressed *CYP450*s were respectively detected in wheat and maize. These genes may have some roles in protecting plants against drought damage. Thereinto, fourteen *CYP450s* were selected to validate their expression level through qRT-PCR. To further elucidating molecular mechanisms of CYP450 action, gene co-expression network was constructed. In total, 477 *TaCYP450*s were distributed in 22 co-expression modules, and some co-expressed genes that likely take part in the same biochemical pathway were identified. For instance, the expression of *TaCYP74A98_4D* was highly correlated with *TaLOX9*, *TaLOX36*, *TaLOX39*, *TaLOX44* and *TaOPR8*, and all of them may be involved in jasmonate (JA) biosynthesis. *TaCYP73A201_3A* showed coexpression with *TaPAL1.25*, *TaCCoAOMT1.2*, *TaCOMT.1*, *TaCCR1.6* and *TaLAC5*, which probably act in the wheat stem and/or root lignin synthesis pathway.

**Conclusion:**

Our study first established systematic information about evolutionary relationship, expression pattern and function characterization of *CYP450*s in wheat and maize.

## Background

At the global level, *Triticum aestivum* (bread wheat) and *Zea mays* (maize) as two of the most important crops, yielded 749 and 1060 million tonnes in 2016, respectively (http://faostat.fao.org/). However, current climate change and abiotic stress adversely affect agriculture and crop production. Therefore, to improve crop management and enhance yield and quality of wheat and maize, it is urgently needed to elucidate the molecular mechanisms of development and stress resistance.

The cytochrome P450s (CYP450s) are found in different life forms ranging from prokaryotes to eukaryotes but it is in plants that their number has exploded. By far, CYP450s constitute the largest family of enzymes in plant metabolism, and an increasing amount of evidence supports the importance of plant P450s in all domains of life. For instance, oxidized fatty acids have important biological activities, and production of hydroxy-fatty acid is mainly catalyzed by CYP450s such as families CYP703, CYP704, CYP92, CYP81, CYP77, CYP78, CYP96, CYP709, CYP726, CYP86 and CYP94, of which CYP86 and CYP94 contribute the essential biomolecules to cover surfaces of pollen, aerial parts and root [1–3]. In the synthesis of diverse secondary compounds, CYP450s play an important role as well. Subfamilies CYP73A, CYP98A, CYP84A are involved in hydroxylation of the aromatic ring of the cinnamates, a core reaction in phenylpropanoid pathway, which provides a vast number of phenolic compounds functioning as structural components (lignin, suberin), flavors (benzenoids, phenylpropenes), UV protectants (flavonoids), antioxidants (polyphenols) and antimicrobials (coumarins, lignans, isoflavonoids) [4]. Flavonoid 3′-hydroxylases (F3’Hs) and flavonoid 3′,5′-hydroxylases (F3’5’Hs) competitively control the synthesis of the precursors of blue and red anthocyanins, and CYP75B and CYP75A respectively function as F3’Hs and F3’5’Hs in the determination of flower colour [5]. Currently, Thapsigargin become a research hotspot of the anticancer drug Mipsagargin, and it is *TenctlyThapsia garganica* CYP76AE2 that mediates the conversion of epikunzeaol to epidihydrocostunolide compounds which are possible intermediates in thapsigargin biosynthesis [6]. Another essential role of CYP450s is the regulation of plant hormone homeostasis. CYP97 family performs the key enzymatic steps in the biosynthesis of xanthophyll [7], the precursor of abscisic acid (ABA). On the other hand, CYP707As encoding ABA 8′-hydroxylases catalyze the first committed step in ABA catabolic pathway. Recently, strigolactones (SLs) have been identified as branching inhibition hormones in plants, and several CYP711As have been experimentally confirmed as SLs biosynthetic enzymes [8, 9]. In gibberellins (GAs) formation, *ent*-kaurene oxidase (KO) (CYP701A) and *ent*-kaurenoic acid oxidase (KAO) (CYP88A) are required, and the involvement of subfamily CYP735A in the production of trans-zeatin, one kind of natural cytokinin, has been elucidated [10–12]. And the biosynthesis and catabolism of brassinosteroids (BRs) need the participation of CYP450s including subfamilies CYP90A, CYP90B, CYP90C, CYP90D, CYP724B, CYP85A and CYP734A [13, 14]. Also, CYP450s promote plants growth and development at various stages of the plant life cycle through different mechanisms. *ZmCYP78A1* has ability to stimulate leaf growth by extending cell division duration [15]. In rice, mutation in *cyp96B4* leads to altered cell wall composition by affecting the expression of cell wall-related genes like *CESA1*, *CESA3*, *CESA4*, *CESA7*, *CESA8*, *CESA9*, *BC1* and *BC10* [16]. Arabidopsis CYP78A5 acts through SWR1-mediated activation of *WRKY28* expression to restrict megaspore mother cell fate to a single cell [17]. *TaCYP78A3* participates in influencing wheat seed size through affecting the extent of integument cell proliferation [18]. CYP450s function in stress responses as well, such as drought, salinity, fungal infection and pest infestation. For instance, down-regulated expression of *OsCYP707A7* by RNAi confers drought tolerance in rice with higher ABA content and antioxidant enzyme activities [19]. Overexpression of *Spinacia oleracea CYP85A1* can enhance resistance to dehydration stress in transgenic tobacco accompanied by increased castasterone content [20]. And loss-of-function mutation in *AtCYP709B3* via T-DNA insertion results in a salt intolerance phenotype in Arabidopsis with delayed germination under 150 or 200 mM NaCl condition [21]. As for production of phytoalexin, CYP79B2, CYP71A13 and CYP71B15 respectively catalyze the conversion of tryptophan to indole-3-acetaldoxime, indole-3-acetaldoxime to indole-3-acetonitrile, cysteine-indole-3-acetonitrile to camalexin in Arabidopsis [22–24]. In maize, the role of *CYP71Z18* in antibiotic zealexin biosynthesis by catalyzing oxidation of C15 in (*S*)-β-macrocarpene is verified using in vitro assays [25]. And both in vitro and in vivo experiments revealed that ZmCYP79A61 accept L-phenylalanine as substrate to produce phenylacetaldoxime, and simulated herbivory on maize leaves cause an increased accumulation of *ZmCYP79A61* transcripts and phenylacetaldoxime, which suggests the enzyme contributes to maize herbivore-induced aldoxime formation [26].

For nomenclature and classification of CYP450 genes, a universal system has been set up by P450 nomenclature committee (David Nelson: dnelson@uthsc.edu) according to the protein sequence identity and phylogeny. The root symbol *CYP* is followed by an Arabic numeral for family, a letter for subfamily and a number for the gene. Pseudogenes are assigned by the addition of the letter “P” immediately after the numeral family designation. To distinguish from other organisms, plant CYP450s are being assigned names from CYP71A1 to CYP99XY, then from CYP701A1 and above [27]. Originally, CYP450s in plant are grouped in two main clades: A-type and non-A type. The majority of CYP450s involved in plant specialized metabolism are distributed in A-type, and non-A-type P450s are thought to be associated with more basic metabolism like sterol and lipid oxygenation and hormone biosynthesis. Recently, the plant CYP450s have been re-classified into 11 clans (clans, which represent the deepest diverging gene clades in the *CYP* nomenclature). The A-type is now grouped as CYP71 clan, whereas the non-A type has 10 clans, namely CYP51, CYP72, CYP74, CYP85, CYP86, CYP97, CYP710, CYP711, CYP727 and CYP746 [28, 29].

Despite many efforts have been made to discover the functional characterization of CYP450s, there is only a little information about the wheat and maize CYP450s. And with more and more genome sequences available, an ever-growing number of CYP450 sequences were identified. To date, the genes have been identified in many plant species at a whole-genome scale, such as *Arabidopsis* [30], *Oryza sativa* [31], *Nelumbo nucifera* [32], *Glycine max* [33] and *Taxus chinensis* [34], whereas no systematical investigation of *CYP450*s has been performed in wheat and maize. In the present study, we carried out the first comprehensive analysis of wheat and maize genomes for CYP450 superfamily. By integrating these data, we try to uncover the roles of *CYP450*s in growth and development as well as in drought adaption, and this work will contribute to advanced research and applications of the genes in the Gramineae crops.

## Results

### Classification and characterization of identified CYP450s in wheat and maize

The wheat and maize genomes respectively contained 1285, 263 full-length CYP450 genes and 2, 7 designated pseudogenes (Additional files 1 and 2: Table S1 and S2). According to standard nomenclature, there are 45 families in wheat, and due to lack of CYP723 and CYP729, only 43 families existed in maize (Additional file 3: Figure S1). CYP71 was the largest A-type family in wheat (404 genes) and maize (56 genes), while wheat CYP96 and maize CYP72 emerged as the largest non-A type family with 82 and 16 members respectively. Families CYP703, CYP715, CYP85, CYP722, CYP724 and CYP733 consisted of a single gene in maize implying a unique highly conserved function for each, but they were found with gene duplication in wheat. The TaCYP450s ranged in molecular weights from 10.92–102.06 kDa with coding sequences of 100–897 amino acids. In maize, the proteins were 135 to 1122 amino acids long with molecular weights of 14.92–126.44 kDa. And most proteins fell in the range of 400 to 600 amino acids in both species. In isoelectric point (pI) values, CYP450s had large variations ranging from 4.55 to 11.52 for wheat and from 4.99 to 10.59 for maize, implying the diversity of the biochemical properties of CYP450s. A total of 465 (36.0%), 709 (54.9%), 110 (8.5%), 8 (0.6%) TaCYP450s; and 94 (34.8%), 150 (55.6%), 25 (9.3%) and 1 (0.3%) ZmCYP450s possessed zero, one, two and three transmembrane domains, respectively. About subcellular localization, the vast majority of CYP450s were likely positioned on the endoplasmic reticulum. Of particular interest is that all members of CYP74 and CYP701 families were targeted to chloroplasts. Also, TaCYP727A2_2B, ZmCYP75B87 and ZmCYP88A56P were detected as chloroplast-localized proteins. Only three (ZmCYP81A17, ZmCYP81A2 and ZmCYP81A36) were located in mitochondria, two (ZmCYP71T29-fusion and ZmCYP72A351P) in cytoplasm and one (TaCYP710A8_3A) in nucleus. TaCYP71BU11_3D was only considered secreted, whose unusual localization might reflect a unique biological function.

Further, we searched for the conserved motifs of A-type and non-A-type CYP450s in wheat and maize. As for A-type, the relatively conserved C-terminal region usually consisted of motifs 1, 14, 23, 3, 30, 12, 5, 4, 2, 7, 13 and 27. The motifs 1, 3, 4, 2 were associated with the functionally defined domains, which corresponded to characteristic p450 consensus sequence Ala/Gly-Gly-X-Asp/Glu-Thr/Ser (AGxD/ET), Glu-X-X-Arg (ExxR), Pro-X-Arg-X (PxRx) and Phe-X-X-Gly-X-Arg-X-Cys-X-Gly (CxG) respectively (Additional files 4 and 5: Figure S2 and S3). As shown in Additional files 6 and 7: Figure S4 and S5, each clan in non-A-type had the specific arrangement of motifs, and that may be the main cause of the protein functional diversification among different clans. The specific motif 16–27–15-4-5-22-8-24–10-17-2-9-3-12-20-6-1-19-13 layout was universally conserved in CYP72 clan, motif 21–15–8-17-9-3-12-6-1-13 layout in CYP711 clan, and motif 21–15–18-14–10-17-2-3-12-6-1-13 layout in CYP51 clan. One thing to be noted is that motif 14 was a characteristic feature of CYP51 clan in both species. In CYP85 clan, the motif 21–15–4-5 arrangement within N-terminal region appeared to be specific to CYP707 family. CYP86 clan generated a unique motif 3–7–6-23–1-13 layout in its C-terminal region. Motif 26 occurred only in subfamilies CYP94E and CYP94C. Obviously, the members of clans CYP727, CYP710 and CYP74 seemed to have lost many motifs. The most highly conserved motifs 2, 3, 6 and 1 respectively represented AGxD/ET, ExxR, PxRx and CxG in non-A-type.

### Phylogenetic and gene structure analysis

To dissect the evolutionary relationships of plant CYP450s, an unrooted maximum likelihood (ML) tree including seven species (*C.reinhardtii*, *P.patens*, Arabidopsis, maize, wheat, rice and poplar) was reconstructed. In the tree, there exist 91 families as defined in the standardized nomenclature, of which 19 are green alga-specific, 15 are moss-specific and the remaining 57 encompass the CYP450 diversity existing in the five angiosperms. As illustrated in Additional file 8: Figure S6, families within the same clan are usually closely clustered together. With only a few exceptions, green algae-specific families CYP737, CYP738, CYP739 and CYP740 were clearly separated from the others in CYP85 clan; CYP743 and CYP744 families specific to green algae in CYP711 clan did not cluster with CYP711 family. It seemed that some ancient families or clans exhibited a closer evolutionary relationship, for instance, CYP710 clan clustered with CYP51 clan; CYP97 family was adjacent to some green algae-specific families in clans CYP711 and CYP85 (i.e. CYP743, − 744, − 737, − 738, − 739 and − 740). Regarding the clustering of families within the same clan, some interesting phenomena were also observed. For example, CYP93, CYP705 and CYP712 were closely clustered together just as CYP735 was phylogenetically closer to CYP714 and CYP715. Similarly, CYP77, CYP89 and CYP723 showed a close evolutionary relationship in the global tree. In addition, the phylogenetic tree placed CYP83 and CYP99 inside the CYP71 family, and CYP723 inside the CYP89 family. Notably, monocot specific CYP99 family was obviously closest to poplar CYP71D subfamily.

We further analyzed exon-intron structure and splicing site mapping for discovering wheat and maize CYP450 gene structure diversity and evolutionary divergence. As displayed in Additional files 9-10: Figure S7–8, all members of CYP74 and CYP710 clans were intronless with the exception of *TaCYP74A97_4D*. In CYP86 clan, families CYP86, CYP94 and CYP96 were generally characterized by being intronless, whereas CYP704 family contained 4–6 exons. Within CYP71 clan (A-type), most genes had only a single phase 0 intron, while CYP701 family had more sophisticated structure due to harboring a greater number of exons, and CYP89 family was generally intronless. In contrast, families in CYP97, CYP727, CYP72 and CYP85 clans had very complex gene structures, particularly CYP85, CYP87, CYP88, CYP90, CYP707 and CYP724. Wheat *CYP97A59_6A*, *CYP97A59_6B*, *CYP97A60_6A* and *CYP97A60_6B* contained the maximum number of exons (16) and the most structurally complex maize *CYP450*s were *ZmCYP97A16* and *ZmCYP71T29-fusion* with 15 exons.

### Chromosomal distribution and gene duplication

All *ZmCYP450*s were unevenly scattered over the ten chromosomes, and chromosome 1 contained the most genes with the number of 47 whereas the chromosome 7 had the least with the number of 13 (Additional file 11: Table S3). The relatively high densities of *ZmCYP450*s were observed in chromosomes 1, 2 and 4 with approximately 0.15 gene per Mb, while chromosome 7 had the lowest density (0.07 gene per Mb). As depicted in Additional file 12: Figure S9, we defined 36 gene clusters (designated as cluster a to aj), of which 31 included tandemly repeated genes (Additional file 13: Table S4). *ZmCYP72A*s formed two of the largest clusters (cluster q and ad) on chromosomes 3 and 8, and CYP72A also showed large clusters in Arabidopsis and rice. Among 79 pairs of genes that underwent tandem duplication, 76 were within the same subfamily except for *ZmCYP709E8*/*ZmCYP709H1*, *ZmCYP709E9*/*ZmCYP709H1* and *ZmCYP71K29*/*ZmCYP71Y10*. A total of 29 segmental duplication events were detected, and only three pairs (*ZmCYP71Y10*/*ZmCYP71AF7*, *ZmCYP709E9*/*ZmCYP709C14*, and *ZmCYP709E4*/*ZmCYP709C23*) were from different subfamily. Taken together, seven *ZmCYP450*s (*ZmCYP96B23*, −*709E9*, −*74A38*, −*92A1*, −*72A5*, −*71Y10* and -*709E4*) that underwent both tandem duplication and segmental duplication were identified. Duplication events in families CYP71, CYP72 and CYP709 outnumbered the others, implying their major role in expansion of maize *CYP450*s. The Ka/Ks of 108 duplicated gene pairs is 0.07 to 1.08, and their corresponding duplication events might occur in 0.38 to 255.05 Mya (millions of years ago). *ZmCYP74A18*/*ZmCYP74A38* not only suffered positive selection with Ka/Ks ratio greater than 1, but also was the youngest pair that might undergo a more recent duplication estimated at 0.38 Mya. In wheat, genome wide distribution indicated the sharing of 387, 431 and 423 *TaCYP450*s from A, B and D sub-genomes respectively, and the remaining 46 were located on scaffolds; they were distributed on each chromosome with different frequencies (Additional file 11: Table S3). A maximum of 128 genes were positioned on chromosome 2B, while a minimum 136 on chromosome 4B. On account of the incomplete genome sequence of wheat, only 172 adjacent gene pairs were detected, including 140 pairs of tandemly duplicated *TaCYP450*s. The duplicated pairs having a Ka/Ks ratio between 0.08 and 0.89 could occur within last 180.05 to 3.45 Mya.

GoGe SynMap program was used to detect synteny regions among Arabidopsis, maize, rice and wheat (Additional file 13: Table S4). None of the collinear CYP450 gene pairs were detect between Arabidopsis and maize. And the absence of synteny between maize and wheat genomes involving the CYP450 gene regions could be due to unavailability of wheat complete chromosome sequences. Using the rice genome as a reference to investigate the syntenic regions, 134 *ZmCYP450*s (49.4%) had their syntenic counterparts in rice genome. And these pairs were mainly distributed in families CYP71 and CYP78. The Ka/Ks ratio for all the pairs varies from 0.08 to 5.53, and the estimated divergence time was approximately between 23.38 and 240.40 Mya. *ZmCYP86B22*/*OsCYP86B3* and *ZmCYP94C20*/*OsCYP71Z5* were under positive selection with Ka/Ks ratio greater than 1.

### Functional divergence analyses of CYP51, CYP74 and CYP97 clans among wheat, maize and rice

**Fig. 1:**
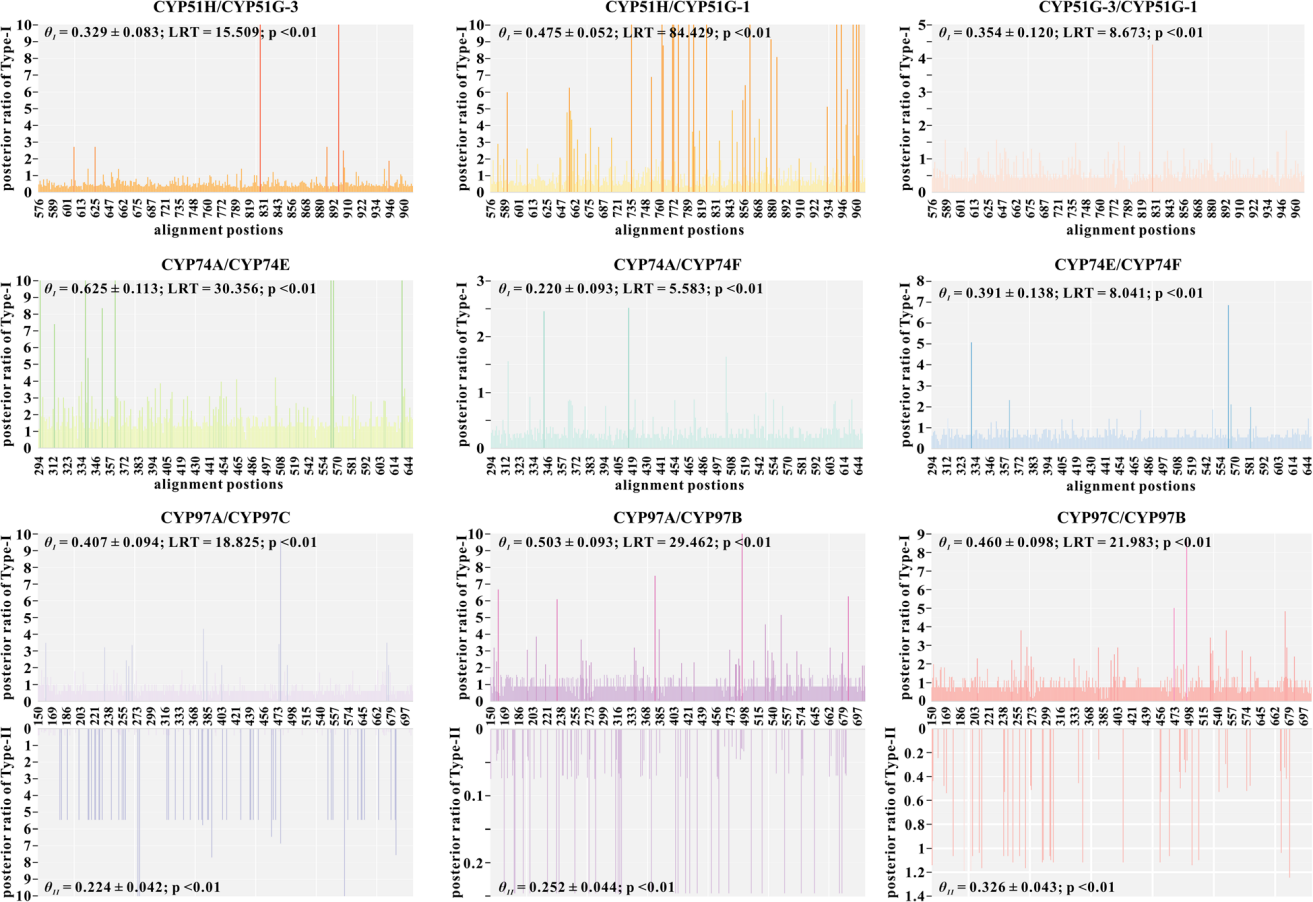
Site-specific profiles for two types of functional divergence (Type-I and Type-II), measured by the posterior ratio. *θ*_*I*_ and *θ*_*II*_, the coefficients of Type-I and Type-II functional divergence between two gene clusters; LRT, Likelihood Ratio Statistic

Functionally divergent sites may contribute to explaining specific functional classes of protein family and the substrate specificity of each protein. Here, we focused on calculated Type-I and Type-II functional divergence for clans CYP51, CYP74 and CYP97, because these clans are conserved across evolution and classified in distinct subfamilies acting on different substrates. As illustrated in Fig. 1 and Additional files 14, 15, 16: Figure S10–12, each family was divided into three gene clusters named as CYP51H/CYP51G-1/CYP51G-3, CYP74A/CYP74E/CYP74F and CYP97A/CYP97B/CYP97C. In CYP51 clans, we found evidences for Type-I functional divergence between pairs CYP51H/51G-3 (*θ*_*I*_ = 0.329 ± 0.083), CYP51H/51G-1 (*θ*_*I*_ = 0.475 ± 0.052) and CYP51G-1/51G-3 (*θ*_*I*_ = 0.354 ± 0.120), indicative of site-specific altered selective constraints; a total of 6, 48 and 1 CAASs (critical amino acid sites) were detected between the three group pairs, respectively. And the coefficients of Type-II functional divergence (*θ*_*II*_) between them were less than 0 or insignificant. The similar cases were found between CYP74 clusters as well, that is, the Type-I functional divergence tests of CYP74A/E (*θ*_*I*_ = 0.625 ± 0.113), CYP74A/F (*θ*_*I*_ = 0.220 ± 0.093) and CYP74E/F (*θ*_*I*_ = 0.391 ± 0.138) were statistically significant and identified 66, 2 and 4 CAASs respectively, while all the pairs showed no Type-II functional divergence. These findings support the hypothesis that shifts of evolutionary rate and changes of amino acid property should not uniformly act on CYP51 and CYP74 clans during long-term evolution. The degree of both types of functional divergence between CYP97A/C (*θ*_*I*_ = 0.407 ± 0.094; *θ*_*II*_ = 0.224 ± 0.042), CYP97A/B (*θ*_*I*_ = 0.503 ± 0.093; *θ*_*II*_ = 0.252 ± 0.044) and CYP97C/B (*θ*_*I*_ = 0.460 ± 0.098; *θ*_*II*_ = 0.326 ± 0.043) were remarkably significant. There are 13, 40 and 21 Type-I functional divergence-related residues for CYP97A/C, CYP97A/B and CYP97C/B, respectively, and the CAASs that may have radically changed their amino acid properties were only found in CYP97A/C. In comparison with the number of CAASs for Type-I, 54 Type-II CAASs were identified for CYP97A/C, indicating that functional divergence between CYP97A and CYP97C was mainly attributed to the changes of amino acid property.

### Assigning protein secondary structure elements and homology modeling

**Fig. 2:**
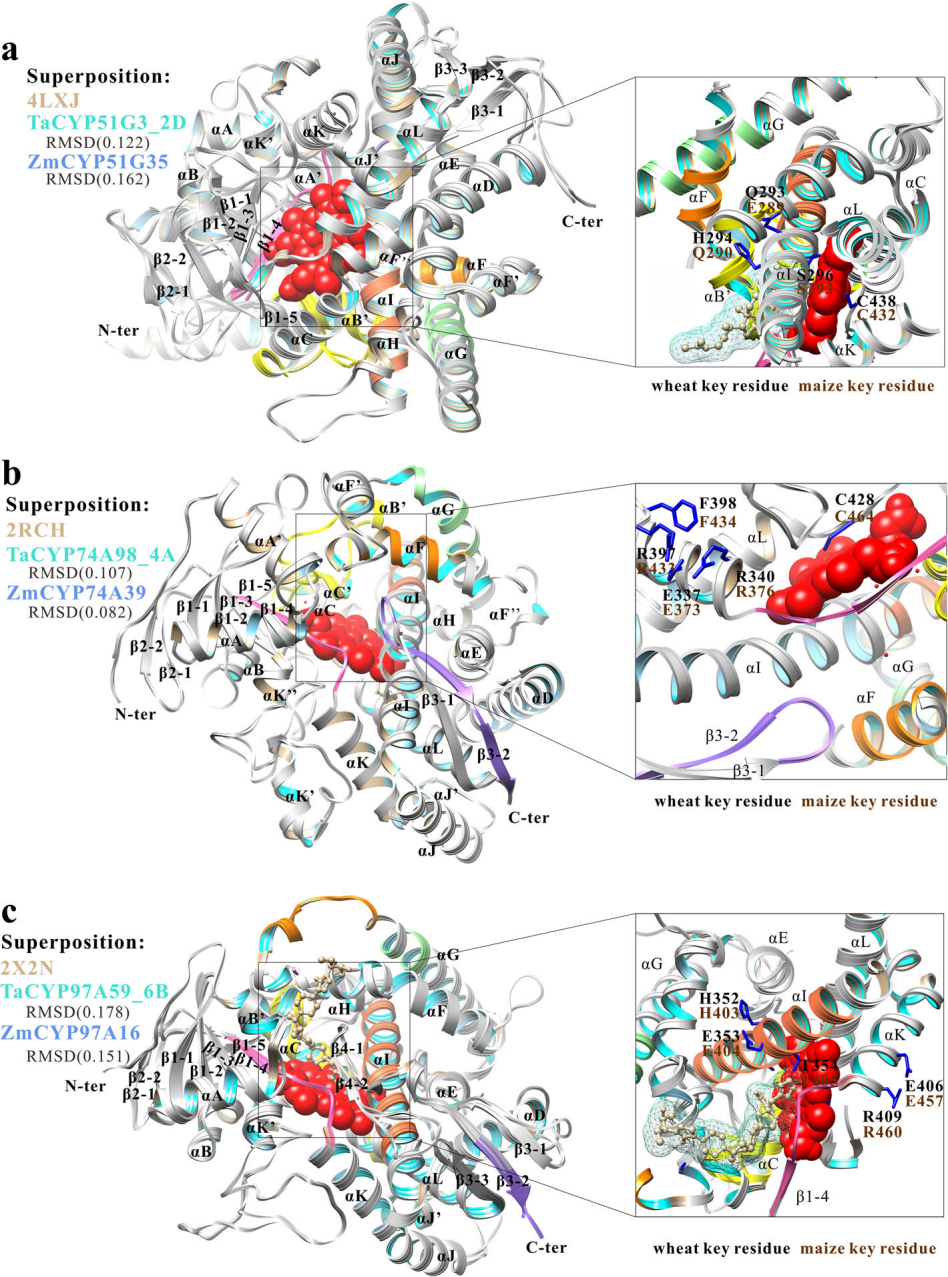
Overview of structures. Key residues are shown in stick presentation, substrate molecule in ball-and-stick model, and heme molecule as spheres. Single-letter abbreviations for the amino acid residues are as follows: C, Cys; H, His; T, Thr; Q, Gln; R, Arg; E, Glu; and F, Phe. **a** Superposition of 4LXJ, TaCYP51G3_2D and ZmCYP51G35. Lanosterol molecule is shown in ball-and-stick model. The RMSD between the 4LXJ and TaCYP51G3_2D is 0.122 Å. The RMSD between the 4LXJ and ZmCYP51G35 is 0.162 Å. **b** Superposition of 2RCH, TaCYP74A98_4A and ZmCYP74A39. The RMSD between the 2RCH and TaCYP74A98 is 0.107 Å. The RMSD between the 2RCH and ZmCYP74A39 is 0.082 Å. **c** Superposition of 2X2N, TaCYP97A59_6B and ZmCYP97A16. The RMSD between the 2X2N and TaCYP97A59 is 0.178 Å. The RMSD between the 2X2N and ZmCYP97A16 is 0.151 Å. POSACONAZOLE molecule is shown in ball-and-stick model. SRS1–6: six putative SRS (substrate recognition sites) regions which are involved in recognition and binding of substrates according to Gotoh’s predicted models

With a better understanding of the structure/function relationship of CYP450s from wheat and maize, we deciphered the 3D structure of CYP51, CYP74 and CYP97 clans of interest by homology modeling using Phyre2 server. The results revealed that 490, 479, 462, 465, 437 and 435 residues of TaCYP51G3_2D, ZmCYP51G35, TaCYP74A98_4A, ZmCYP74A39, TaCYP97A59_6B and ZmCYP97A16 (99, 98, 96, 91, 79 and 68% of amino acid sequence) have been modelled with 100.0% confidence by the single highest scoring template. As shown in Additional file 17: Figure S13, the Ramachandran plot analysis for nine predicted models (TaCYP51G3_2D, ZmCYP51G35, TaCYP74A98_4A, ZmCYP74A39, TaCYP97A59_6B and ZmCYP97A16) showed that the vast majority of residues fell in favoured regions. And the qualities of the models were further supported by high ERRAT scores, which signify acceptable protein environment. In spite of relatively low amino acid sequence identity among different organisms, they appear to take on a similar secondary and tertiary structural fold throughout evolution (Additional files 17, 18, 19, 20: Figure S13C, S14–16 and Fig. 2). Sixteen α-helices (A-L, B′, J’, K′, K″) and 3 β-sheets including a five-stranded sheet (β-sheet 1), a three-stranded sheet (β-sheet 3) and a two-stranded sheet (β-sheet 2) are in common, while β-sheet 4 also containing two strands is not present in CYP74. The four-helix bundle (helices D, E, I, and L) and helices J and K form CYP450 conserved structural core. With exception of CYP74 family, the central part of the I-helix contains consensus sequence (A/GGxD/ET/S) involved in oxygen binding. At the beginning of L helix occurs a heme binding region FxxGxRxCxG with the absolutely conserved cysteine that serves as fifth ligand to the heme iron. Notably, the nine-residue insertion in heme-binding loop is specific to CYP74 family. Helix K contains the conserved ExxR motif that is on the proximal side of the heme. Furthermore, Glu and Arg of ExxR motif and the Arg in the “PxRx” consensus sequence comprise E-R-R triade that is probably involved in locking the heme pockets into position and stabilizing the core structure. We also identified six putative SRS (substrate recognition sites) regions which are involved in recognition and binding of substrates according to Gotoh’s predicted models. The highly variable loop region between helices B and C lines the SRS1; helices F and G and their loop house SRS2 and SRS3; SRS4 lies in I helix and SRS5 in β1–4; and the β-turn between β4–1 and β4–2 or between β3–1 and β3–2 protrudes into the SRS6.

### Expression profiles of CYP450 genes in various organs and developmental stages

**Fig. 3:**
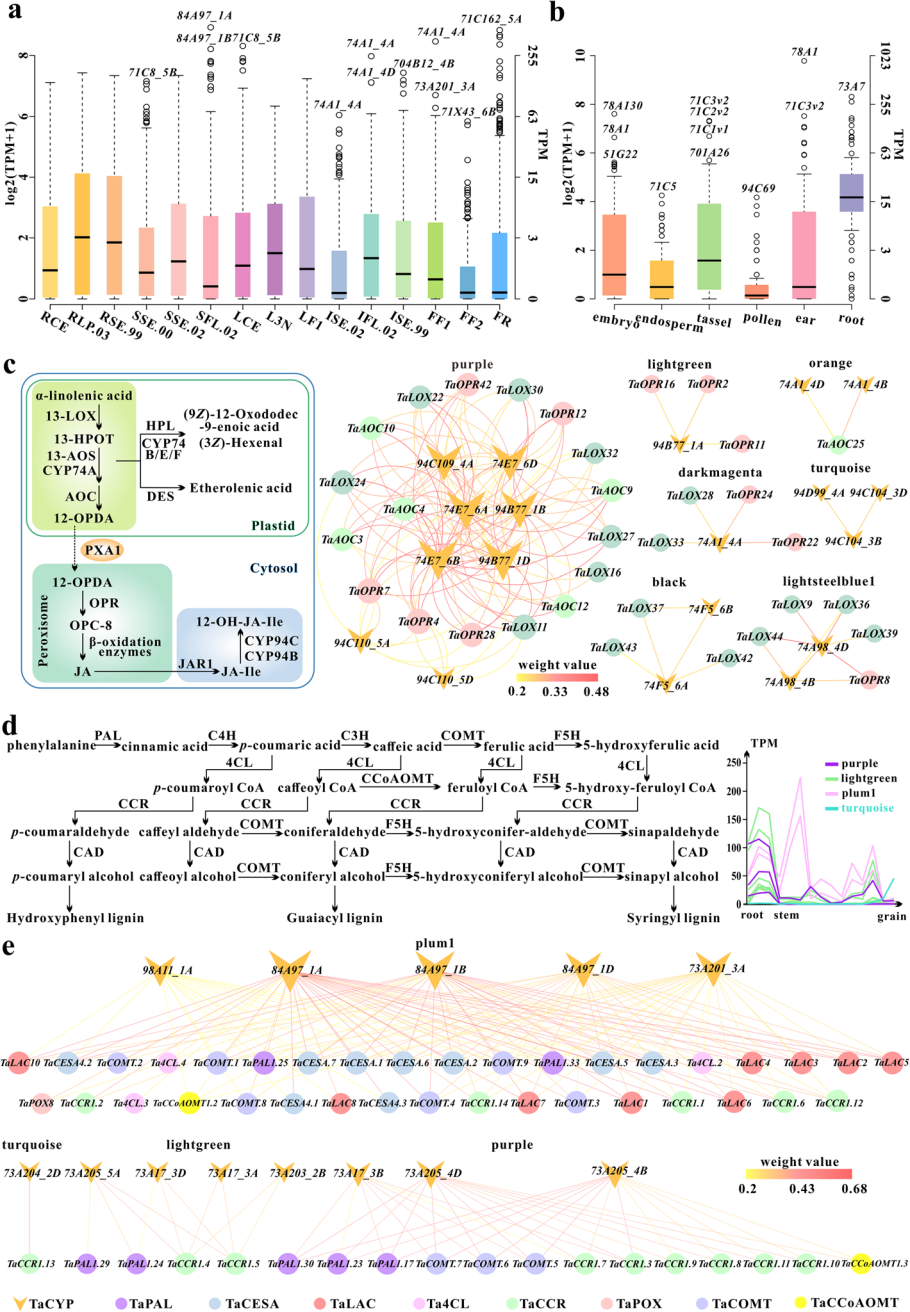
Expression analysis of *CYP450*s during development. **a** Box plot of *TaCYP450*s expression. RCE: Root at cotyledon emergence stage; RLP.03: Root at three leaves visible stage; RSE.99: Root at maximum stem length reached stage; SSE.00:Stem at stem elongation begins stage; SSE.02: Stem at two nodes or internodes visible stage; SFL.02:Stem at 1/2 of flowers open stage; LCE: Leaf at cotyledon emergence stage; L3N:Leaf at main shoot and axillary shoots visible at three nodes stage; LF1: Leaf at whole plant fruit formation stage 30 to 50%; ISE.02: Inflorescence at two nodes or internodes visible stage; ISE.99: Inflorescence at maximum stem length reached stage; IFL.02: Inflorescence at 1/2 of flowers open stage; FF1: Fruit at whole plant fruit formation stage 30 to 50%; FF2: Fruit at whole plant fruit formation stage 70% to final size; FR: Fruit at whole plant fruit ripening stage. **b** Box plot of *ZmCYP450*s expression. **c** The diagram of metabolic pathway involved in JA biosynthesis and co-expression analysis of *TaCYP450*s. **d** The diagram of lignin biosynthesis pathway and expression patterns of *TaCYP73A*s in three sets. **e** Co-expression analysis of *TaCYP450*s involved in lignin biosynthesis.

To gain insights into the spatial and temporal expression patterns of *CYP450*s, we investigated the expression profiles of *TaCYP450*s in root, stem, leaf, inflorescence and grain at three developmental stages; *ZmCYP450*s in root, ear, tassel, pollen, embryo, and endosperm. A total of 402 *TaCYP450*s and 101 *ZmCYP450*s were chosen for expression analysis, because their expression values were ≥ 10 TPM (Transcripts Per Kilobase Million) in at least one tissue (Additional files 21, 22, 23: Table S5, Table S6 and Figure S17). A comparison of gene expression profiles among different organs revealed that wheat CYP450 transcripts were detected in all tissues, but the highest in root, which was followed by stem, leaf, inflorescence and grain (Fig. 3a). Thereinto, *TaCYP73A17_3A*, *−73A17_3B*, *−73A17_3D*, *−74E7_6A* and *-76H2_7D* displayed peak transcript levels in root. *TaCYP89J13_2A*, *−73A205_4B* and *-71F39_2D* were specifically and abundantly expressed throughout root development. Also, in maize, more *CYP450*s were expressed in high quantity in root as compared to other organs such as *ZmCYP73A7* (318 TPM), *ZmCYP707A5* (275 TPM), *− 71C2v2* (173 TPM), −*98A29* (162 TPM), − *71C5* (139 TPM) (Fig. 3b). While in endosperm and pollen, a large majority of *ZmCYP450*s are transcribed at low levels. Only 4 genes (*ZmCYP724B3*, −*72A353*, −*707A116* and -*71C5*) in endosperm and 3 (*ZmCYP84A34*, −*71 T29-fusion* and -*94C69*) in pollen displayed medium level expression (10 to 20 TPM). Additionally, in both species, a considerable proportion of *CYP450*s (356 *TaCYP450*s, 76 *ZmCYP450*s) showed high alterations in expression levels among different tissues (CV (coefficient of variation) > 100%) suggesting that they may have more specific functions. For example, *TaCYP84A97_1B*, −*75B125_7D*, −*93G18_2B* and -*93G18_2D* expressed predominantly in stem, particularly in SFL.02 (Stem at 1/2 of flowers open stage). Expression of *TaCYP71C162_5A*, −*71Y18_1B*, −*71Y18_1D* and -*71Y18_1A* had a high degree of specificity to FR (Fruit at whole plant fruit ripening stage) with TPM value > 300. *ZmCYP89M2*, *− 81 N5* and -*78A131* showed organ-specific expression in embryo; *ZmCYP96B18*, *−86A35* and *-92A47* in tassel; and *ZmCYP707A5*, *−72A5*, −*71C62*, −*92A1*, −*72A123*, −*75B89*, −*94B41*, −*728C14*, −*89B19* in root. The transcripts of *ZmCYP94C69* and *-84A34* were specifically detected in pollen and root. Only a tiny fraction of genes had a relatively stable expression, of which *TaCYP727A2_2A* and *TaCYP96B64/72_3B* exhibited the least variation with a CV value of 42%. The average expression level of *TaCYP96B64/72_3B* is approximately three times higher than that of *TaCYP727A2_2A.*

And then we compared the expression profiles of the homologous genes in wheat and maize. The results showed that members of CYP73A in both species showed preferential expression patterns in the root. Transcripts of *TaCYP51H*s (8) were mainly detected in root, especially *TaCYP51H39_5B*, *−51H43_5B*, *−51H49_4B* and *-51H49_4D*, while *ZmCYP51H12* showed significant expression divergence, which were predominantly expressed in embryo. *TaCYP99A*s without *TaCYP99A42_5D* were highly expressed in the specific tissues, root, whereas *ZmCYP99A*s had no expression in any tissues but slight expression (1 < TPM < 10) in root. Also, the expressional diversity of members from CYP78A in different organs and species was found. Specifically, *TaCYP78A263_4B*, *TaCYP78A263_4D*, *TaCYP78A263_5A* showed preferential transcript accumulation in root; *TaCYP78A265_2D*, *TaCYP78A265_7B* in stem; *TaCYP78A264_5A*, *TaCYP78A264_5B*, *TaCYP78A264_5D* in inflorescence. In contrast, *ZmCYP78A130* expression was confined to embryo and root with much higher expression level in embryo (195 TPM). *ZmCYP78A1* not only displayed high expression level in embryo with 99 TPM but also exhibited extremely high transcript abundance in ear with 880 TPM. We also detected the expression of *ZmCYP78A53* mostly in ear and tassel, with the strongest expression in ear (131 TPM).

### Expression profiles of CYP450 genes under drought stress

**Fig. 4:**
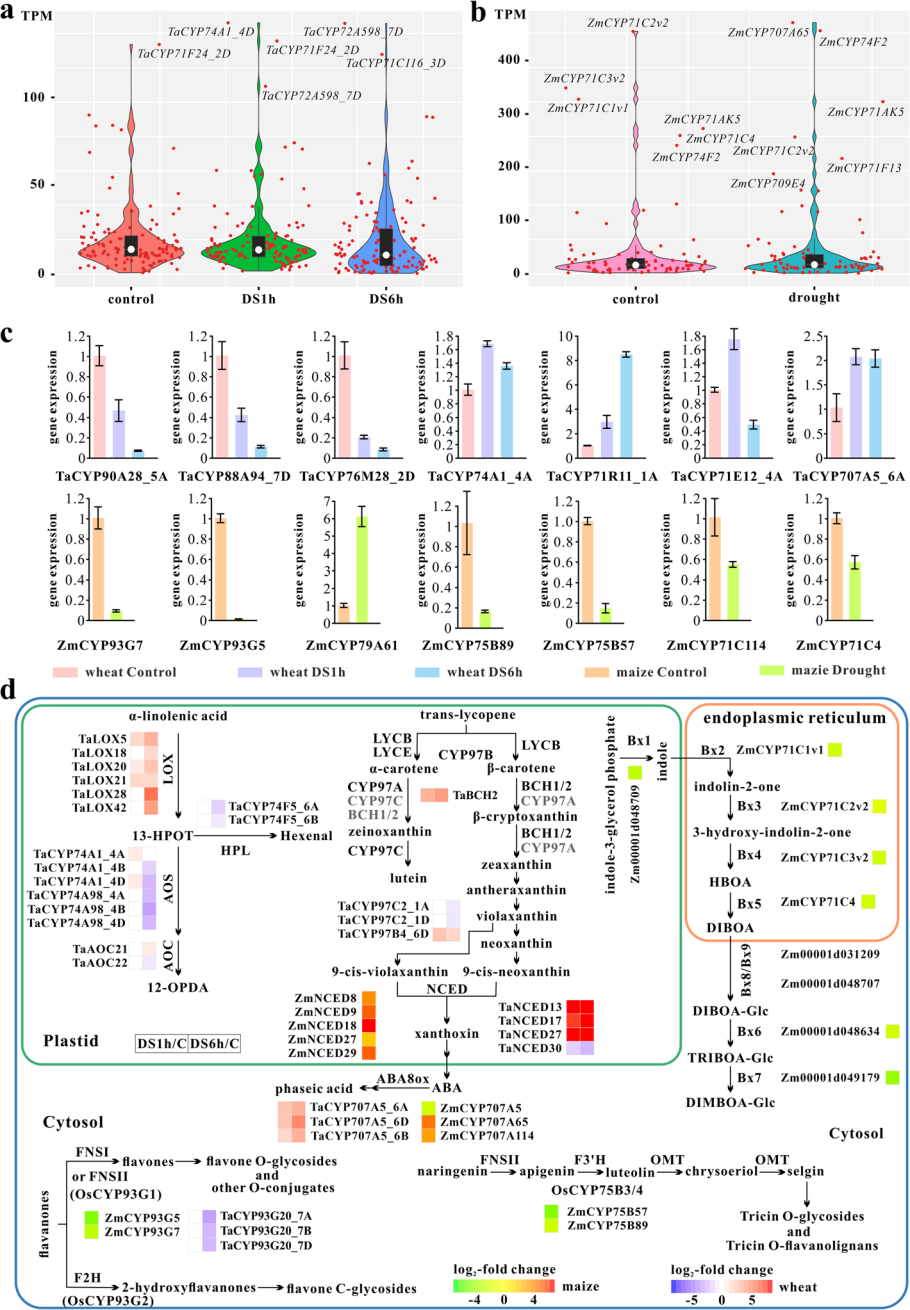
Analysis of the *CYP450*s expression under drought stress. **a** Violin plots showing expression levels of *TaCYP450*s. DS1h: after drought treatment at 1 h; DS6h: after drought treatment at 1 h. **b** Violin plots showing expression levels of *ZmCYP450*s. **c** Validation of the expression level of 14 *CYP450s* by qRT-PCR analysis. **d** A schematic diagram of CYP450-mediated signaling pathways under drought stress

Drought as one of the prevailing abiotic stresses affects various physiological and biochemical processes of plants. To analyzed expression of *CYP450*s in response to drought treatment, 119 TaCYP450 and 86 ZmCYP450 genes whose expression values were ≥ 10 TPM in one or more conditions were selected (Additional files 24-25: Table S7 and S8). For wheat, twenty-six *TaCYP450*s (17 up-regulated and 9 down-regulated) and 77 (27 up-regulated and 50 down-regulated) showed significant changes in their expression levels after drought treatment at 1 h (DS1h) and 6 h (DS6h), respectively (Additional file 26: Figure S18A). Five genes with significant up-regulation at DS1h, i.e. *TaCYP71T45_3B*, −*73A17_3B*, −*73A201_3A*, −*74A1_4A* and -*89E25_7B* were considered as drought sensitive genes. While *TaCYP701A66_U*, −*704A152_3A*, −*704A160_3A*, −*704A164_3D*, −*706C1_7B*, −*71AK14_5A*, −*71C10_3A*, −*71C10_3D*, −*71Y16_1D*, −*72A578_1B*, −*72A598_7A*, −*89E20_1A*, −*89E24_7A*, −*90B46_4B*, −*96B38_2A*, −*96B38_2D*, −*96B58_5A* and -*96B64/72_3B* were significantly induced at DS6h. The transcript levels of *TaCYP71E12_4A*, *−74A1_4D*, *−78A264_5B* and *-84A103_2A* were initially elevated markedly at DS1h and then reduced at DS6h. As the drought continues, the expression of *TaCYP707A5_6A*, *−707A5_6B*, *−707A5_6D*, *−71R11_1A*, *−71T45_U*, *−71Y18_1D*, *−73A17_3A*, *−73A201_3B* and *-97B4_6D* were elevated continuously, whereas *TaCYP714C17_5D*, *−71C142_2D*, *−71C166_6D*, *−71C170_6B*, *−71W30_5B*, *−76M28_2D*, *−88A94_7D*, *−90A28_5A* and *-90A28_5B* exhibited continuously decreased expression. Of note, *TaCYP71Y18_1D* was the most significantly up-regulated gene in both DS1h and DS6h (9.32- and 118.60-fold), and *TaCYP76M28_2D* showed the largest fold change (4.06- and 39.12-fold) among all down-regulated genes. For maize, 39 *ZmCYP450*s were DEGs (19 up-regulated and 20 down-regulated) under dehydration stress (Additional file 26: Figure S18B). Some genes including *ZmCYP81N4* (12 fold up), −*81A1* (10 fold up), −*71C62* (9 fold up) and -*78A53* (4 fold up) were expressed only under drought treatment. And the transcripts of *ZmCYP707A65* (8 fold up, 452.52 TPM) and *ZmCYP71F13* (5 fold up, 208.58 TPM) massively accumulated under water deficiency treatment (Fig. 4b). In contrast, *ZmCYP86A35*, −*93G5*, −*78A55*, −*86B21*, −*71C114*, −*86A97* and -*77B2* expression dropped to undetectable level after exposure to drought.

In a comparison with *CYP450*s expression patterns between the two species, we found wheat differentially expressed genes (DEGs) were only detected in clans CYP51, 74, 97, 71, 72, 85 and 86, while maize DEGs in clans CYP51,727, 97, 71, 72, 85 and 86. There are more than 40 families in wheat and maize, but the DEGs only distributed in less than 26 families, and the overlapping families with DEGs between both species were CYP51, CYP71, CYP76, CYP78, CYP81, CYP89, CYP93, CYP72, CYP707, CYP96 and CYP97. Some *TaCYP450*s had similar expression patterns with their homologs in maize, which were principally occurred in subfamilies CYP71C, CYP93G, CYP72A, CYP704A. For instance, *TaCYP71C142_2D*, *TaCYP71C162_5B*, *TaCYP71C166_6D*, *TaCYP71C170_6B*, *ZmCYP71C114*, *ZmCYP71C1v1*, *ZmCYP71C2v2*, *ZmCYP71C3v2* and *ZmCYP71C4*; *TaCYP93G20_7A*, *TaCYP93G20_7B*, *TaCYP93G20_7D*, *ZmCYP93G5* and *ZmCYP93G7*; *TaCYP72A578_1B*, *TaCYP72A598_7A*, *ZmCYP72A353*, *ZmCYP72A354*, *ZmCYP72A5* and *ZmCYP72A16* were expressed in the same trend that the expression level was significantly decreased under drought stress. *TaCYP704A152_3A*, *TaCYP704A160_3A*, *TaCYP704A164_3D* and *ZmCYP704A105* transcripts significantly increased in response to dehydration. And *TaCYP72A598_7D* showed a high level of transcript accumulation across all three conditions (Control, DS1h and DS6h) with no significant expression changes, whose average expression values were up to 111.24 TPM (Fig. 4a). And *ZmCYP72A124*, the homologous gene most closely related to *TaCYP72A598_7D*, had similar expression trend, although the expression abundance was differentiated. Meanwhile, many genes presented quite different expression profiles to their homologs in maize. For example, *TaCYP96B38_2A*, *TaCYP96B38_2D*, *TaCYP96B58_5A*, *TaCYP96B64/72_3B* and *ZmCYP96B23* showed a totally contrary expression trend. *TaCYP707A5_6A*, *TaCYP707A5_6B*, *TaCYP707A5_6D* exhibited similar expression patterns to *ZmCYP707A5* but opposite to *ZmCYP707A114* and *ZmCYP707A65*. Finally, 14 genes were selected for qRT-PCR analysis to confirm the expression patterns (Fig. 4c). Primers are listed in Additional file 27: Table S9.

### Co-expression modules of *TaCYP450*s

**Fig. 5:**
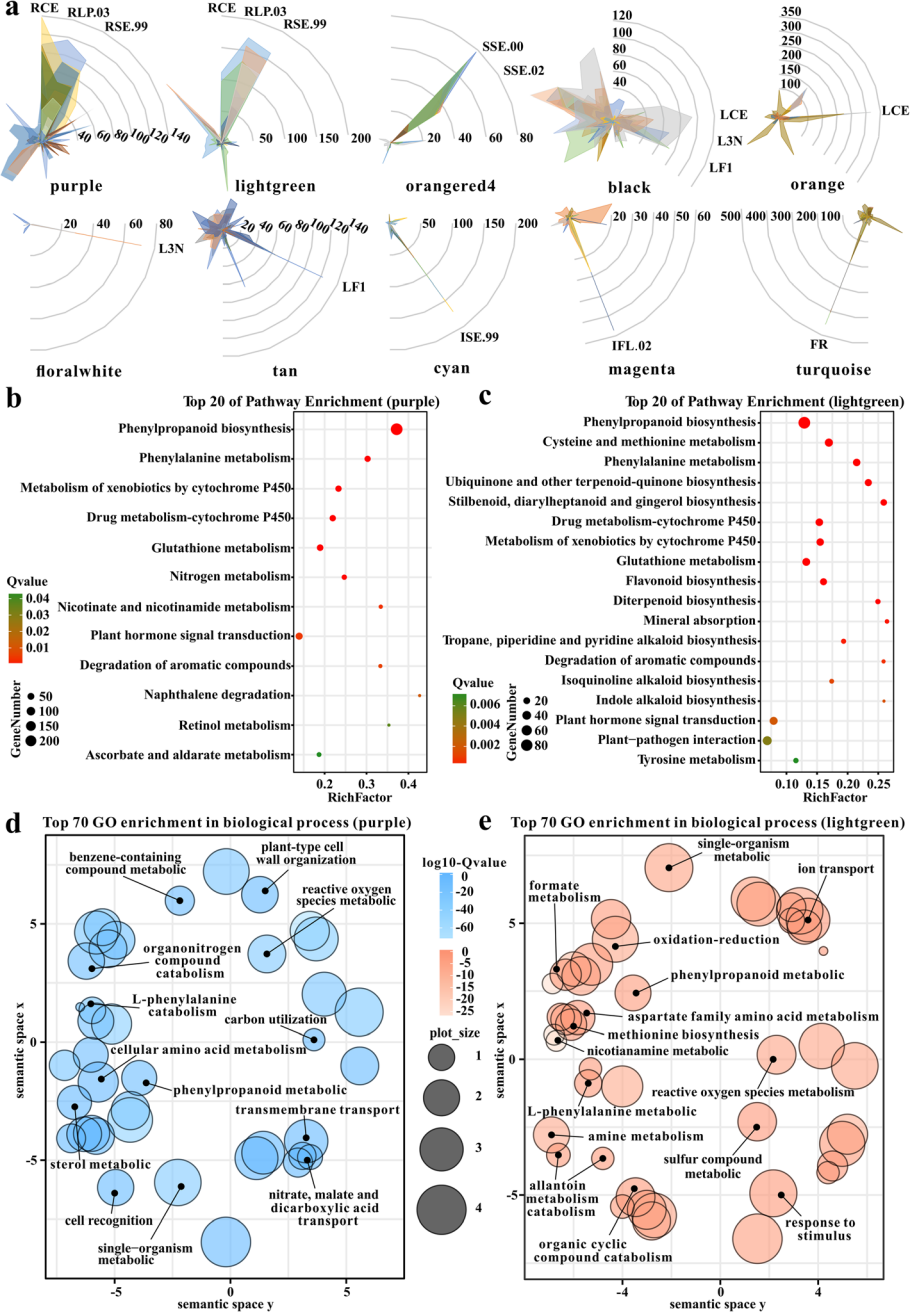
Co-expression modules of *TaCYP450*s. **a** Radar charts of expression values of *TaCYP450*s in 10 modules with the most obvious tissue-specific expression trends. The radar chart displays over a gray polygonal layout. Each axis represents one kind of tissue and each point in gray polygonal layout is labeled with the tissue**.** But we only showed part of the tissues with tissue-specific genes. The larger the radius of a ring represents the higher level of gene expression. Each ring is labeled with a number that represents gene expression level. The expression values of each gene in the corresponding module along each axis of the radar chart are connected linearly to visualize the data set as a polygon, and the different color polygons represent different genes. RCE: Root at cotyledon emergence stage; RLP.03: Root at three leaves visible stage; RSE.99: Root at maximum stem length reached stage; SSE.00: Stem at stem elongation begins stage; SSE.02: Stem at two nodes or internodes visible stage; LCE: Leaf at cotyledon emergence stage; L3N: Leaf at main shoot and axillary shoots visible at three nodes stage; LF1: Leaf at whole plant fruit formation stage 30 to 50%; ISE.99: Inflorescence at maximum stem length reached stage; IFL.02: Inflorescence at 1/2 of flowers open stage; FR: Fruit at whole plant fruit ripening stage. **b** Top 20 most significantly enriched pathways in purple module. **c** Top 20 most significantly enriched pathways in lightgreen module. **d** Top 70 most significantly enriched GO terms in biological process category in a two-dimensional semantic space of purple module. Color intensity reflects the significance of enrichment test. Circle radiuses depict the sizes of the aggregated GO terms. **e** Top 70 most significantly enriched GO terms in biological process category in a two-dimensional semantic space of lightgreen module

Co-expression analysis can further provide valuable clues for functional annotation of genes that have significant and as yet unappreciated roles. In total, 22 co-expression modules were identified and covered 477 *TaCYP450*s belonging to 39 families (Additional file 28: Table S10). These genes showed distinct expression patterns mirroring each of the modules to which they belong. To identify the major molecular and biochemical pathways and functional categories for each module, the gene ontology (GO) enrichment and Kyoto Encyclopedia of Genes and Genomes (KEGG) analyses were performed (Additional file 29: Table S11). Here, the discussion focused on 10 modules because they showed the most clear and distinct characteristics in terms of gene expression profiles (Fig. 5a). Purple module contained 62 *TaCYP450*s with the strongest expression in root. KEGG enrichment analysis revealed significant overrepresentations of phenylpropanoid biosynthesis (ko00940), phenylalanine metabolism (ko00360), metabolism of xenobiotics by cytochrome P450 metabolic pathways (ko00980) and so on (Fig. 5b and c). *TaCYP73A17_3B*, *−73A205_4B* and *-73A205_4D* were annotated into degradation of aromatic compounds pathway (ko01220) with Q-values < 0.007. Lightgreen module where 39 *TaCYP450*s were predominantly expressed in root was highly positively correlated with purple module. Many enriched GO terms and pathways were common such as lignin metabolic process (GO:0009808), phenylpropanoid metabolic process (GO:0009698) and metabolism of xenobiotics by cytochrome P450 metabolic pathway (ko00980). Besides, diterpenoid biosynthesis (ko00904) was unique metabolic pathway in the module (Fig. 5d and e), and more *TaCYP450*s (*TaCYP73A17_3A*, −*73A17_3D*, −*73A202_7A*, *−73A202_7D*, −*73A203_2B*, *−73A205_5A*, −*73A206_7B*, −*75A82_6B*, −*75A84_4A*, −*701A63_2B*) were annotated into the significantly enriched pathways, i.e. phenylalanine metabolism, ubiquinone and other terpenoid-quinone biosynthesis, diarylheptanoid and gingerol biosynthesis, flavonoid biosynthesis, diterpenoid biosynthesis, degradation of aromatic compounds and alpha-Linolenic acid metabolism. In orangered4 module, eight *TaCYP450*s had their peak expression in SSE.00 (stem at stem elongation begins stage) and SSE.02 (stem at two nodes or internodes visible stage).

Fifty-nine *TaCYP450*s with a maximal transcript abundance in leaf were grouped in black module, and carbon fixation in photosynthetic organisms (ko00710), photosynthesis (ko00195), carotenoid biosynthesis (ko00906) and porphyrin and chlorophyll metabolism (ko00860) were the most significantly enriched in the module. Orange module had 20 *TaCYP450*s exhibiting the highest transcript abundance in LCE (Leaf at cotyledon emergence stage), and aminoacyl-tRNA biosynthesis (ko00970), porphyrin and chlorophyll metabolism (ko00860), ribosome (ko03010), protein export (ko03060), RNA degradation (ko03018), ubiquinone and other terpenoid-quinone biosynthesis (ko00130), monobactam biosynthesis (ko00261), selenocompound metabolism (ko00450) and biotin metabolism (ko00780) were significantly overrepresented pathways. Four highly enriched metabolic pathways monoterpenoid biosynthesis (ko00902), glucosinolate biosynthesis (ko00966), diterpenoid biosynthesis (ko00904) and 2-Oxocarboxylic acid metabolism (ko01210) were identified in floralwhite module that harbored three L3N (Leaf at main shoot and axillary shoots visible at three nodes stage)*-*specific expressed *TaCYP450*s, namely *TaCYP71V29_3B*, −*79A141_2A* and -*79A141_2B*, of which the latter two were respectively annotated into glucosinolate biosynthesis and 2-Oxocarboxylic acid metabolism pathways. Terpenoid backbone biosynthesis (ko00900), plant-pathogen interaction (ko04626) and regulation of autophagy (ko04140) showed significant enrichment in tan module including 20 *TaCYP450*s that displayed peak transcript levels in LF1 (leaf at whole plant fruit formation stage 30 to 50%).

Cyan module contained 27 *TaCYP450*s whose expression peak appeared at maximum stem length reached stage of inflorescence (ISE.99) when the anthers release their pollen, and Go terms associated with pollen development (GO:0009555, GO:0010208, GO:0010584, GO:0080110) were significantly enriched, indicating the biological roles of the tissue. Fatty acid metabolism-related pathways (ko01212, ko01040, ko00062, ko00061, ko00071, ko00073) were significantly over-represented in the module. Thereinto, *TaCYP86A154_2A* was annotated into cutin, suberin and wax biosynthesis metabolite pathways (ko00073). Another sexual reproduction related module, magenta, possessed 16 *TaCYP450*s with a high degree of specificity to IFL.02. Forty-six *TaCYP450*s in turquoise module had been described as FR-specific in their expression. The pathways of folate and arginine biosynthesis (ko00790, ko00220), alanine, aspartate, glutamate, thiamine, ether lipid and inositol phosphate metabolism (ko00250, ko00730, ko00565, ko00562) were specifically enriched in the module.

### Prediction of miRNA targets and cis-regulatory elements

To obtain in-depth understanding of the regulation mechanisms of gene expression, miRNA target and cis-regulatory elements predictions were performed (Additional file 30: Table S12). MiRNAs control gene expression through affecting both translation and stability of mRNAs. A total of 46 *TaCYP450*s were predicted to be regulated by 28 miRNAs in 55 miRNA-target interactions in wheat. Seven genes seemed to be targeted by multiple miRNAs, of which *TaCYP51H39_5D*, −*71C9_4A*, −*71S9_2D* and -*75A90_6B* had two regulatory miRNAs, *TaCYP96E3_3D* had three, and *TaCYP97A59_6A* had four. According to the gene expression profiles, a total of 15 predicted target genes seemed to be involved in diverse developmental processes, such as *TaCYP76H2_7D*, −*73A201_3A*, −*74E7_6D*, −*96B53_7A*, −*93G18_2D*, −*92A150_2B* and -*72A597_6B*. And drought-responsive *TaCYP73A201_3A* was targeted by tae-miR5049–3p. In maize, 11 miRNA-target interactions representing nine miRNAs and eight *CYP450*s were found. The transcriptional regulation of *ZmCYP72A28v2* was mediated by four miRNAs. *ZmCYP72A353* possibly as a target of zma-miR399e-5p was significantly up-regulated under drought stress; Root-specific *ZmCYP72A28v2* was targeted by zma-miR172a, zma-miR172b-3p, zma-miR172c-3p and zma-miR172d-3p; zma-miR156j-5p acted as potential regulator of *ZmCYP78A53* that had its peak expression in ear.

Cis-regulatory elements serve as binding sites for transcription factor to regulate gene expression. A promoter scan revealed 75 and 97 kinds of trans-acting binding sites in drought-responsive *CYP450*s in wheat (82) and maize (39), respectively. Among them, 15 elements have high frequency of occurrence (> 50) such as the drought-related ABRE (ABA responsive element), TGACG- and CGTCA-motif (MeJA-responsiveness), and MBS (MYB binding site involved in drought-inducibility). ABA has a central role in the complex cascade of drought response, and there is a hormone cross-talk in plant adaptation to drought stress. Of hormone-responsive elements, ABRE was the most abundant element of the drought-responsive genes, e.g. *TaCYP707A5_6A*, *TaCYP707A5_6B*, *TaCYP707A5_6D*, *ZmCYP707A65*, *TaCYP97B4_6D*, *TaCYP97C2_1A*, *TaCYP97C2_1D* and *ZmCYP81N4*. The second most abundant hormone-responsive element is MeJA-responsiveness element possessed by 66 *TaCYP450*s and 26 *ZmCYP450*s, among which the drought-inducible gene *TaCYP74A1_4D*, a homolog of JA biosynthesis-related *AOS*, had two MeJA response elements CGTCA- and TGACG-motif. Others hormone response elements like GA-responsive P-box, TATC-box, GARE-motif and SA-responsive TCA-element were also found to be apparently abundant. P-box resided in *TaCYP88A94_7D* that represents an orthologue of GA biosynthetic gene *KAO1* but had significantly decreased expression. MBS was distributed in 38 *TaCYP450*s and 26 *ZmCYP450*s, such as *TaCYP71R11_1A*, *TaCYP71C10_3D*, *TaCYP71Y18_1D*, *ZmCYP81A1*, *ZmCYP71C62* and *ZmCYP71F13*, and *TaCYP71Y18_1D* was the most highly induced gene under dehydration stress. Only *ZmCYP51H12*, *TaCYP97C2_1A* and *TaCYP714C17_5D* harbored MBSI involved in flavonoid biosynthetic gene regulation, of which *ZmCYP51H12* was upregulated after exposure to drought.

## Discussion

### Phylogenetic analysis

This review presents a comparison of CYP450s from seven sequenced genomes of *C.reinhardtii*, *P.patens*, Arabidopsis, maize, wheat, rice and poplar. Nelson classified the 11 land-plant clans into two groups: single-family clans (CYP51, CYP74, CYP97, CYP710, CYP711, CYP727, CYP746) and multi-family clans (CYP71, CYP72, CYP85, CYP86) [35]. CYP51 clan may represent the oldest eukaryotic CYP, and CYP51Gs are the only CYP450 enzymes with a conserved sterol demethylation function across the fungal, plant and animal [36]. Notable is, *C.reinhardtii*, *P.patens*, Arabidopsis and poplar contain a subfamily CYP51G with one to two genes, but maize, rice and wheat each have at least 3 *CYP51G*s plus additional a functionally divergent subfamily CYP51H that mediates the synthesis of defense-related antimicrobial triterpene glycosides (avenacins). The three grass species have from 7 to 37 *CYP51*s, and the number of *CYP51H*s varies from 3 (42%) in maize, 7 (70%) in rice and up to 29 (78%) in wheat. CYP710 clan contains sterol C22-desaturases [37], which is conserved from green algae to vascular plants, and CYP85 clan can also acts on sterols [38]. It is not surprising that clans CYP51, CYP710 and CYP85 are closely related in the phylogenetic tree. These observations suggest that they may have evolved from a sterol metabolizing CYP51 ancestor. In CYP85 clan, CYP724B/CYP90 couple functioning in the biosynthesis of BRs are found to be phylogenetically close. Further, CYP724B shares higher phylogenetic identity with CYP90B in the tree, and coincidentally, both can mediate the same C-22 hydroxylation steps in the pathway [39]. Additionally, the members of families CYP88 and CYP729 in CYP85 clan form a strong cluster with a bootstrap confidence of 996/1000. Howbeit, CYP88 family involved in GA biosynthesis [11] has one to three genes in each of the five land plants but none in *C.reinhardtii* and *P.patens*, while CYP729 whose function remains unclear exists only in poplar (1 genes), wheat (9 genes) and rice (2 genes), suggesting the function of CYP729 is possibly related to the GA formation.

It is well known that CYP97, one of the oldest plant-specific clans that emerged before the higher plant/green algae split, functions in xanthophyll biosynthesis and comprises three distinct subfamilies [7]. Each subfamily is present in *P.patens*, Arabidopsis, poplar, maize and rice in single copy, but wheat has 12 (6 *CYP97A*s, 3 *CYP97B*s and 3 *CYP97C*s) members. CYP711 clan probably appeared after the CYP97 clan during early plant evolution, which has a single (sub) family (CYP711A) involved in strigolactone signalling in land plant [35]. CYP711As are usually found singly or in low copies in most dicots, but with gene duplication in monocots, i.e. 4 genes in maize, 6 in rice and 12 in wheat. On the global tree, CYP97 clan shares the highest phylogenetic identity with some green algae-specific families in clans CYP711 and CYP85. One potential explanation for this result is that they share a common ancestor. It is well documented that the atypical CYP450s (CYP74s) can catalyze the conversion of already oxygenated polyunsaturated C18 fatty acid hydroperoxides into other oxylipins [40]. In our phylogenetic tree, CYP74 clan is adjacent to CYP711 family, and further functional analysis of these families might provide some hint about the evolution of the oxylipins and strigolactone signals and their ancestor’s function(s). CYP71 clan is absent in *C.reinhardtii* while already dominant in early land plants and involved in the plant specialized metabolism [35]. It is the largest land plants CYP clan with a current total of 1615 members in the six species (*P.patens*, Arabidopsis, maize, wheat, rice and poplar), and particularly wheat contains in excess of 780 members which is estimated at up to 60.64% of total *TaCYP450*s. Our phylogenetic analysis showed that CYP84 clusters with a bootstrap value of 985/1000 to the CYP736. CYP736 only exist in poplar among seven species, and its function has been not studied, while CYP84 has been confirmed to involve in lignin biosynthesis in angiosperms.

### Expression analysis of *CYP450*s during development

Analyzing gene expression profiles can advance our understanding of CYP450s functions in wheat and maize growth and development. It is widely accepted that cytochrome P450 proteins implicated in a number of developmental events through the biosynthesis and/or catabolism of phytohormones and other secondary compounds. For example, CYP51G can catalyze the essential 14α-demethylation of obtusifoliol, which is required for the synthesis of phytosterol and membrane sterols. In our research, *CYP51G*s were ubiquitously expressed in wheat and maize tissues. Moreover, *TaCYP51G1_4A* and -*51G1_4D* exhibited relatively high expression abundance in root and inflorescence; *ZmCYP51G1* in tassel and ear; *ZmCYP51G22* in embryo. It seems that each gene performs a prominent role in different tissues or species. Unlike the conserved function of CYP51Gs, oats CYP51H10 is indispensable to produce avenacins [41]. As expected, wheat *CYP51H*s transcripts were mainly detected in root where the avenacins accumulate, whereas maize *CYP51H12* had a higher expression in embryo than other organs.

Xanthophylls play critical roles in photosynthesis, photoprotection and as precursors to ABA. Recently, some genes associated with xanthophyll biosynthesis have been experimentally defined. In Arabidopsis, LYCB and LYCE catalyze the conversion of trans-lycopene to α- or β-carotene, and subsequently, BCH1/2 and CYP97s participate in the hydroxylation of α- and β-carotene [7, 42]. In our study, *TaCYP97*s had their peak expression in leaf, particularly *TaCYP97C2_1D* whose expression level was more than 93 TPM in LCE. Among them, *TaCYP97A60_6A*, −*97A60_6B* and -*97A60_6D* showed coexpression with the wheat homologs of Arabidopsis *LYCB* (6BS_TGACv1_513174_AA1632970), *LYCE* (3AL_TGACv1_193581_AA0613700, 3B_TGACv1_221427_AA0740510 and 3DL_TGACv1_250106_AA0862170) and *BCH1* (2AL_TGACv1_093571_AA0282760) in black module. By contrast, all members of maize CYP97 family (*ZmCYP97A16*, −*97B21* and -*97C19*) were expressed at a low to moderate level in all tissues.

Mounting evidence suggests *CYP707A*s encode ABA 8′-hydroxylases in ABA catabolism. We detected the expression of *TaCYP707A5_6A*, −*707A5_6B* and -*707A5_6D* mostly in root and grain. What’s more, they were evenly expressed in three phases of root development but exhibited great disparities in abundance among different grain development stages, that is, the transcripts were nearly absent in grain during the early period of fruit development while accumulated significantly at whole fruit ripening stage. As is well known, increased amounts of ABA are associated with the maturation of seeds. Accordingly, it is reasonable to envision that *TaCYP707A5_6A*, −*707A5_6B* and -*707A5_6D* play the major role in maintaining optimal ABA levels of ripening wheat grains. In maize, *CYP707A5* transcript was also specifically expressed in root with 275 TPM. And its closest homologue in rice, *OsCYP707A5*, showed preferential expression not only in root but also in palea and ovary [31]. *ZmCYP707A114* had a higher expression in tassel. These inspire us to assess their potential importance in the control of organ-specific processes.

Strigolactones (SL) are secreted from root with various biological roles, like regulating shoot branching or tillering and root architecture. To the best of our knowledge, AtCYP711A1 (MAX1) has been identified as a SL biosynthetic enzyme, and the enzymatic functions of its homologs such as OsCYP711A2, OsCYP711A3, OsCYP711A5, OsCYP711A6 and ZmCYP711A18 for SL formation were also confirmed in vitro [8]. In our study, *TaCYP711A68_3B*, *TaCYP711A68_4A, TaCYP711A68_4D* and *ZmCYP711A18* expressions were concentrated to root. Besides, *TaCYP711A67_6B,* 53% identical to *MAX1* at amino-acid level, was coexpressed with *TaCYP97A60_6B* and reached the highest expression level in LF1.

Functional studies in Arabidopsis have demonstrated that CYP74A1 functions as an allene oxide synthase (AOS) in the JA biosynthetic pathway [43]. In wheat, *TaCYP74A1*s were expressed at varying intensities throughout wheat development, particularly *TaCYP74A1_4A* and -*74A1_4D* with extremely high expression levels in LCE (220, 223 TPM), IFL.02 (250, 137 TPM) and FF1 (351, 64 TPM). While the expression of *TaCYP74A98_4A*, −*74A98_4B* and -*74A98_4D* were restricted to LCE and L3N, and *TaCYP74A99_5A* and -*74A99_5B* exhibited relatively high transcript abundance in SSE.02. The first step of JA biosynthesis is the oxidation of α-linolenic acid in plastids, which is initially driven by lipoxygenases (LOXs). And then the product, 13-HPOT ((13S)-hydroperoxy octadecatrienoic acid), can be metabolized by AOSs and AOCs (allene oxide cyclases) to generate 12-OPDA (12-oxo-phytodienoic acid) (Fig. 3c) [43]. With the aim to better explore the formation of JA by CYP74s in wheat, 49 *TaLOX*s (*TaLOX1* -*TaLOX49*) and 25 *TaAOC*s (*TaAOC1*-*TaAOC25*) were identified (Additional file 31: Table S13). As revealed by our coexpression analysis, some of them showed a significant correlation of expression with *TaCYP74A*s (homologs of *AOS*) (Fig. 3c), for example, *TaAOC25* connected to *TaCYP74A1_4B* and *TaCYP74A1_4D* in orange module; *TaLOX28* and *TaLOX33* connected to *TaCYP74A1_4A* in darkmagenta module. And 12-OPDA can be transported into peroxisomes by PXA1 [44], then OPR and β-oxidation enzymes catalyze the formation of JA from 12-OPDA [43]. In this report, a total of 55 *TaOPR*s (*TaOPR1–55*) were identified in wheat (Additional file 31: Table S13), among which *TaOPR22* and *TaOPR24* connected to *TaCYP74A1_4A* in darkmagenta module; *TaOPR88* connected to *TaCYP74A98_4B* and *TaCYP74A98_4D* in lightsteelblue1 module. Additionally, previous researches have provided compelling evidence that Arabidopsis CYP94B1, CYP94B3 and CYP94C1 contribute to partial inactivation of the JA-Ile hormone when JA signaling must be switched off [45, 46]. In co-expression network, *TaCYP94B77_1B*, −*94B77_1D*, −*94C109_4A*, −*94C110_5A* and -*94C110_5D* from purple module showed connection to some JA synthesis-related genes.

CYP73A, CYP98A and CYP84A may act as cinnamate 4-hydroxylase (C4H), *p*-coumaroyl ester 3′-hydroxylase (C3′H) and ferulate/coniferaldehyde/coniferyl alcohol 5-hydroxylase (F5H or Cald5H) in lignin biosynthesis, respectively (Fig. 3d) [47, 48]. The latest research finds that C4H, C3′H and F5H are in spatial proximity to each other on the ER membrane, and it is MSBP1/2 (membrane steroid-binding proteins) that physically organize and stabilize them [49]. In this study, 1BL_TGACv1_030938_AA0104050, 1AL_TGACv1_002731_AA0044810 and 1DL_TGACv1_063516_AA0228130 share sequence homology with MSBP1/2, two of which showed a significant correlation of expression with *TaCYP73A204_2D*. According to the expression profile, wheat *CYP73A*s were divided into three sets: (1) *TaCYP73A17_3A*, *−73A17_3B*, *−73A17_3D*, *−73A202_7A*, *−73A202_7D*, *−73A203_2B*, −*73A205_4B*, −*73A205_4D*, −*73A205_5A* and -*73A206_7B* distributed in lightgreen or purple modules have maximal expression levels in root; (2) *TaCYP73A201_3A* and *TaCYP73A201_3B* belonging to plum1 module exhibited maximal transcript abundance in stem and root; (3) the expression of *TaCYP73A204_2D* assigned to turquoise module was mostly confined to grain (Fig. 3d). To further understand the roles of *TaCYP73A*s, special focus is placed on co-expression subnetworks produced by all the connections for *TaCYP73A*s with an edge weight > 0.2 (Fig. 3e). In the subnetworks, we identified a suite of wheat genes homologous to Arabidopsis lignin biosynthesis-related genes such as *PAL1/2* (phenylalanine ammonia-lyase1/2), *4CL1/2* (4-coumarate: CoA ligase1/2), *CCoAOMT1* (caffeoyl-CoA O-methyltransferase1), *CCR1* (cinnamoyl-CoA reductase1), *COMT* (caffeic acid O-methyltransferase) (Additional file 32: Table S14). Most wheat genes (41) with putative role in lignin synthesis were present in the subnetwork from purple module, among which *TaCYP73A17_3B*, *TaCYP73A205_4B* and *TaCYP73A205_4D* had the highest correlation for coexpression with *TaPAL1.13*, *TaPAL1.31*, *TaCCoAOMT1.3*, *TaCOMT.7* and *TaCCR1.3*. In lightgreen module, there are direct connections between *TaPAL1.24*, *TaPAL1.29*, *TaCCR1.5*, *TaCCR1.4* and the *TaCYP73As* (*TaCYP73A17_3A*, −*73A17_3D* -*73A203_2B* and -*73A205_5A*). Based on our results, we propose that they most likely act in concert with each other for root lignin formation. A striking observation was that all kinds of lignin biosynthesis-related *TaCYP450*s (*TaCYP73A201_3A*, *TaCYP98A11_1A* and *TaCYP84A97_1A*/*TaCYP84A97_1B*/*TaCYP84A97_1D*) were simultaneously present in plum1 module. And they were coexpressed with *TaPAL1.25*, *TaPAL1.33*, *Ta4CL.2*, *Ta4CL.3*, *Ta4CL.4*, *TaCCoAOMT1.2*, *TaCOMT.3*, *TaCOMT.1*, *TaCOMT.2*, *TaCOMT.4*, *TaCOMT.8*, *TaCOMT.9*, *TaCCR1.1*, *TaCCR1.2*, *TaCCR1.6*, *TaCCR1.12* and *TaCCR1.14*. Additionally, the oxidation of monolignols is the last step of lignin biosynthesis, which is driven by laccases and peroxidases [50–52]. In Arabidopsis, the involvement of LAC17 in the constitutive lignification of stems has been proved [53]. Fortunately, nine *LAC17* orthologues in wheat (Additional file 31: Table S13) were identified here as being coexpressed with *TaCYP73A201_3A*, *TaCYP84A97_1A*, *TaCYP84A97_1B*, *TaCYP84A97_1D* and *TaCYP98A11_1A*. In the meanwhile, the five *TaCYP450*s also showed correlation of expression with nine wheat cellulose synthase genes (Additional file 32: Table S14) involved in secondary cell wall biosynthesis. Only one peroxidase (*TaPOX8*) was detected showing coexpression with *TaCYP84A97_1A* and *TaCYP84A97_1B*. All connections between these genes produced a lignin biosynthesis co-expressed network, which can provide candidate genes that encode enzymes active in wheat stem and/or root lignin biosynthetic pathway. In maize, all CYP450 genes with potential role for lignin biosynthesis (*ZmCYP73A122*, *ZmCYP73A8*, *ZmCYP73A7*, *ZmCYP98A29*, *ZmCYP98A7* and *ZmCYP84A34*) were maximally expressed in root. AtCYP703A2 has been experimentally verified to function as a lauric acid in-chain-hydroxylase involved in sporopollenin synthesis along with AtCYP704B1 that catalyzes ω-hydroxylation of long-chain fatty acids [2, 54]. And in rice, the key role of anther-specific CYP703A3 and CYP704B2 in synthesizing anther cuticle and pollen exine has also been proved [55, 56]. Consistent with previous researches, the expression of *TaCYP703A3_7A* and *-703A3_7B* were mostly restricted to inflorescence at maximum stem length reached stage, and they exhibited a similar expression pattern to *TaCYP704B12_4A*, −*704B12_4B* and -*704B12_4D*. Likewise, the coexpression analysis showed that the five wheat genes mentioned above assigned to cyan module are closely associated with each other.

Besides, many CYP450s have been shown to function in diverse biological processes by producing an as-yet-unknown signalling molecule, which is thought to be independent from any classical phytohormone. In Arabidopsis, *CYP85A1* is expressed in the female gametophyte and necessary for the initiation of megagametogenesis [57]. Correspondingly, relatively high expression of *OsCYP85A1* was detected in pistil, inflorescence and anther [58]. Not only that, it reached peak expression in seed at 10 days after pollination with 112 TPM [59]. Of note, *ZmCYP85A1* transcript was the most abundant in root, with a relatively lower level in embryo, tassel and ear, and nearly absent in pollen and endosperm. And *TaCYP85A51_2D*, as a close homologue to *OsCYP85A1* and *ZmCYP85A1*, showed a low or undetectable level of expression in all wheat tissues. Extensive research efforts have shown that several CYP78As are involved in regulation of seed development and organ size. Arabidopsis *CYP78A6*, *CYP78A8* and *CYP78A9* perform overlapping functions in integument development by promoting cell proliferation [60, 61]. In rice, *CYP78A13* is expressed in both embryo and endosperm and required for proper size balance between them [62]. We found that the expression value of *ZmCYP78A130* sharing close amino acid identity (70%) with *OsCYP78A13* was up to 195 TPM in embryo. A striking accumulation of *ZmCYP78A1* transcripts in ear (880 TPM) and embryo (99 TPM) were detected. And *ZmCYP78A53* was mainly expressed in ear (131 TPM), which overlaps with the expression trends of *ZmCYP78A54*, but not with the *ZmCYP78A133* that appeared to be regulated only by ear-specific transcriptional activation.

### Expression analysis of *CYP450*s in response to drought stress

The concentration of ABA markedly increases under drought stress conditions, which plays a crucial role in preventing water loss. ABA 8′-hydroxylase (ABA8Ox) activity, functionally assigned to CYP707A, has been demonstrated, and the induction of *CYP707A*s seems to be important for the maintenance of optimal ABA levels [63]. We discovered that *TaCYP707A5_6A*, −*707A5_6B* and -*707A5_6D* were incrementally up-regulated with exposure time, and *OsCYP707A5* also showed an ascent as 19.7-fold of the control under drought stress [31]. Their orthologous gene *ZmCYP707A5* showed significantly decreased expression of at least 2.6-fold. Some ABA-biosynthesis-related genes (33 *TaNCED*s and 29 *ZmNCED*s) encoding 9-cisepoxycarotenoid dioxygenases (NCEDs) were identified (Additional file 33: Table S15), which cleave C40 caroteniods to form the first 15-carbon precursor xanthoxin. Thereinto, *TaNCED13*, *TaNCED17* and *TaNCED27* showed sharp increase of transcripts in drought-treated leaves, whereas *TaNCED30* exhibited significantly reduced expression. In maize, all significantly differentially expressed *NCED*s were upregulated, including *ZmNCED8*, *ZmNCED9*, *ZmNCED18*, *ZmNCED27* and *ZmNCED29*. Also, wheat *BCH2* (5AL_TGACv1_376349_AA1235590) acting upstream of xanthoxin production showed significantly strong induction. Another upstream gene *TaCYP97B4_6D*, homologous to *AtCYP97B3* capable of hydroxylation of both α- and β-carotene [42, 64], was expressed at 5.5- and 2.77-fold increased levels after exposure 1 h and 6 h of drought stress, respectively. It has been reported that 12-Oxophytodienoic acid (12-OPDA) acts as a drought-responsive regulator of stomatal closure and functions most effectively together with ABA [65]. In accordance with expectation, *OsCYP74A4* (*OsAOS*) was up-regulated 5.9-fold when exposed to drought stress [31], and *TaCYP74A1*_*4A* and *TaCYP74A1_4D* were significantly up-regulated in DS1h. Six *TaLOX*s (*TaLOX5*, *TaLOX18*, *TaLOX20*, *TaLOX21*, *TaLOX28* and *TaLOX42*) were up regulated in at least one condition. And there are only three *TaAOC*s expressed in our experiment, among which *TaAOC21* had a 2-fold increase in expression while *TaAOC22* had a reduction, two-fold, of expression after drought treatment at 6 h.

ROS production results in various degree of oxidative damage under drought stress, and flavonoids may act as ROS scavengers in Arabidopsis [66]. In rice, of identified flavonoids biosynthesis-related genes *OsCYP93G1* [67], *OsCYP93G2* [68] and *OsCYP75B4* [69], *OsCYP93G2* was significantly down-regulated in response to dehydration [31]. In wheat, putative flavonoid biosynthesis genes *TaCYP93G20_7A*, *TaCYP93G20_7B* and *TaCYP93G20_7D* had more than 5-fold decrease in expression within 6 h of drought exposure. Also, four maize potential flavonoid biosynthetic genes *ZmCYP93G5*, *ZmCYP93G7*, *ZmCYP75B57* and *ZmCYP75B89* showed significant downregulation. Overaccumulation of antioxidant flavonoids enhance oxidative and drought tolerance in Arabidopsis, but the mechanism of flavonoid accumulation in Gramineae crops remains to be clarified.

The benzoxazinoids 2,4-dihydroxy-1,4-benzoxazin-3-one-glucoside (DIBOA-glucoside) is one of the potential metabolite biomarkers serving as indicators of drought tolerance, which shows a decreased level in wheat under drought stress [70]. Its biosynthesis in maize involves 7 enzymes (Bx1-Bx5, Bx8 and Bx9) which act in the synthesis of DIBOA-glucoside from indole-3-glycerol phosphate by sequential reactions, and four of them are CYP450s, namely ZmCYP71C1v1 (Bx2), ZmCYP71C2v2 (Bx3), ZmCYP71C3v2 (Bx4) and ZmCYP71C4 (Bx5) [71]. In this study, there are apparent downregulation of *Bx1* (Zm00001d048709) and *Bx2*-*Bx5* expression, and *Bx8* (Zm00001d048707) and *Bx9* (Zm00001d031209) showed no significant changes in their expression levels. While in wheat, the identified *Bx2*-*Bx5* [72] corresponding to *TaCYP71C9_4A*/*4B*/*U*, *−71C7_5A*/*5B*/*5D*, *−71C6_5A*/*5B*/*5D* and *-71C8_5A*/*5B*/*5D* showed low expression level under water deficiency conditions. For ease of interpretation, a schematic diagram of CYP450-mediated signaling pathways under drought stress is shown in Fig. 4d.

## Conclusion

The first complete overview of CYP450 superfamily in wheat and maize is presented in our study, including protein characterization, phylogeny, gene structure, chromosome location, duplicated event, functional divergence and gene expression pattern, which could provide important features for the genes in corresponding species. What’s more, a comparative analysis between these genes in wheat and maize was also performed. As a result of these analyses, we found cytochrome P450s belong to a large superfamily with hundreds to thousands of members in wheat and maize, and most CYP450 families are present in both species, except for CYP723 and CYP729 which may have been lost in maize. The comparative analyses of the phylogenetic tree, conserved motifs and gene structures of wheat CYP450s with those of maize reveal that each family/subfamily possesses specific features that may have related molecular functions, and the characteristics features of each family/subfamily in both species are conserved. High-throughput transcriptome data in various development stages and drought stress can offer an in-depth insight into functions of *CYP450*s in different physiological processes. In both species, *CYP450*s exhibit great disparities in abundance among different tissues, and most of them are considered as highly tissue-specific genes. It seems that a minority of *CYP450*s are significantly differentially expressed under drought stress. Based on the analyses of gene expression profile and co-expression network, we focused on the discussion of the expression patterns and functions of interesting *CYP450*s. Some homologous genes in wheat and maize with common trends in expression were identified, suggesting that these genes are highly conserved during evolution and may have similar functions. The divergences in expression profiles between homologous genes also exist, revealing that some of them may lose function or acquire new function in evolutionary process. In summary, our work represents the first comparative genomics analysis of CYP450 superfamily in wheat and maize. These results not only extend previous findings related to the role of the genes but also provide valuable information to investigate Gramineae crops development and stress physiology and make a prospect for genetic improvement programs.

## Methods

### Identification and characterization of TaCYP450s and ZmCYP450s

The wheat (TGACv1.32) and maize (AGPv4.36) protein sequences were retrieved from the EnsemblPlants database [73]. The known CYP450 protein sequences from Pfam database [74] (Pfam code: PF00067), The Arabidopsis Information Resource (TAIR) (https://www.Arabidopsis.org/), P450 Homepage (http://drnelson.uthsc.edu/cytochromeP450.html) and the report published by Wei et al. [31] were utilized to build HMM profiles with hmmbuild implemented in HMMER 3.0 [75], which was then used to identify CYP450 proteins in each genome (wheat and maize) by hmmsearch. Subsequently, we merged the results and deleted redundant sequences. To confirm the domain organization, the candidate CYP450 protein sequences were characterized using Pfam database and ScanProsite server [76], and the sequences lacking or containing incomplete CYP450 domain were eliminated manually. Then all maize and wheat candidates were submitted to the Standardized Cytochrome P450 Nomenclature Committee (David Nelson: dnelson@uthsc.edu) for uniform nomenclature. TMHMM server v. 2.0 (http://www.cbs.dtu.dk/services/TMHMM/) and LocTree3 [77] were used to predict trans-membrance regions and subcellular localization, respectively. The isoelectric point (pI) and molecular weight (Mw) were estimated by IPC (Isoelectric Point Calculator) [78]. The program of MEME tool in Galaxy web-based platform was exploited [79] to analyze the motif compositions with the parameters: number of different motifs as 30, range of motif width as 8 to 50.

### Phylogenetic tree construction and gene structure analysis

The CYP450 protein sequences from *Chlamydomonas reinhardtii* (green alga), *Physcomitrella patens* (moss), *Populus trichocarpa* (poplar), *Arabidopsis thaliana* (Arabidopsis), *Oryza sativa* (rice), *Triticum aestivum* (wheat) and *Zea mays* (maize) were aligned by using MAFFT with default parameters (−auto) [80]. Based on the aligned sequences, a maximum-likelihood (ML) tree was constructed by FastTree with JTT substitution model [81] and visualized using iTOL [82], and topological robustness was assessed by boot-strapping with 1000 bootstrap replicates. Besides, the available annotation information of wheat and maize genomes was utilized to display CYP450 gene structures by TBtools [83].

### Chromosomal localization, gene duplication, synteny and selective pressure analyses

The genes were mapped on chromosomes based on their physical positions provided in the EnsemblePlants database. Visualization of chromosome location was implemented with all *CYP450*s using MapDraw 2.2. The adjacent genes in the same CYP family located within 10 genes apart were considered as tandemly duplicated genes [31]. The prediction of segmentally duplicated genes and syntenic regions was performed by GoGe SynMap program [84]. To explore the mechanism of gene divergence after duplication, the ratio of non-synonymous to synonymous nucleotide substitutions (Ka/Ks) between duplicated gene pairs was calculated by DnaSP 5.0 [85]. The dates of the duplication events for gene pairs were calculated by the equation T = Ks/2λ × 10^− 6^ Mya (λ = 6.5 × 10^− 9^) [86]. Circos-0.67 program [87] was used to visualize duplicated regions between maize and rice.

### Estimation of functional divergence

The software DIVERGE 3.0 was utilized to detect critical amino acid sites (CAASs) responsible for the functional divergence of CYP450 family [88]. The coefficients of Type-I and Type-II functional divergence (*θ*_*I*_ and *θ*_*II*_, respectively) between two chosen clusters were calculated to measure the level of functional divergence. If *θ*_*I*_ and *θ*_*II*_ are significantly superior to 0, it means that some amino acids may have experienced shifted functional constraints (Type-I functional divergence) or a radical shift of amino acids physicochemical properties (Type-II functional divergence) during evolution. Then an empirical cutoff of posterior probability Q_k_ > 0.67 was used to verify the functional divergence-related sites by a site-specific posterior analysis.

### Assigning protein secondary structure elements and homology modeling

To better understand the structure and functional relationship of CYP450 enzymes from wheat and maize, CYP51, CYP74 and CYP97 sequences as the representatives were selected and aligned. Assignment of secondary structure elements onto the corresponding aligned sequences was performed using the program ESPript [89], and substrate recognition sites (SRSs) were manually indicated according to Gotoh’s predicted models [90]. To screen the best target and templates for homology modeling, all sequences from wheat and maize CYP51, CYP74 and CYP97 clans were submitted to Phyre2 [91]. The strategy used in the model building was based on confidence in the model, coverage of the query sequence and target-template sequence similarity. Finally, TaCYP51G3_2D, ZmCYP51G35, TaCYP74A98_4A, ZmCYP74A39, TaCYP97A59_6B, ZmCYP97A16 as the best representatives for each clan were chosen. And the homology models for TaCYP51G3_2D and ZmCYP51G35, TaCYP74A98_4A and ZmCYP74A39, TaCYP97A59_6B and ZmCYP97A16 based on the crystal structure of *Saccharomyces cerevisiae* lanosterol 14-alpha demethylase (CYP51, PDB: 4LXJ, chain: A), Arabidopsis allene oxide synthase (CYP74A, PDB: 2RCH, chain: A), and Trypanosoma brucei lanosterol 14-alpha-demethylase (CYP51, PDB: 2X2N, chain: B) were generated using the Phyre2. To validate the quality of homology model, stereochemical quality and accuracy was evaluated by Ramachandran plot analysis in the RAMPAGE server (http://mordred.bioc.cam.ac.uk/~rapper/rampage.php), and ERRAT at the UCLA-DOE Structure Analysis and Verificantion (https://servicesn.mbi.ucla.edu/SAVES/) was used to assess the amino acid environment. UCSF Chimera (http://www.rbvi.ucsf.edu/chimera) was employed to visualize the three-dimensional (3D) structures.

### Alignment of RNA-seq reads and expression analysis

To study the expression of wheat and maize CYP450 genes in various developmental stages and response to drought stress, the raw sequencing data in FASTQ and SAM formats were obtained from ArrayExpress [92] with accession numbers E-MTAB-4484, E-MTAB-3826 and E-MTAB-4198, and NCBI Sequence Read Archive (SRA) database (https://www.ncbi.nlm.nih.gov/sra) with accession number SRP045409. Then we aligned high quality paired-end reads from each sample to wheat reference genome sequence TGACv1 and maize reference genome sequence AGPv4 using HISAT2 [93]. The raw counts of unique mapped reads were aggregated using featureCounts [94]. And using a custom-made Perl script, we calculated TPM (transcripts per million) for each gene. As for drought stress, differential gene expression analysis was performed with the Bioconductor package DESeq2, and fold change cutoff of two and Q-value (false discovery rate adjusted *p*-value) < 0.05 were taken as statistically significant threshold. To identify differentially expressed genes (DEGs) in different developmental stages, the coefficient of variation (CV) value of expression level across all tissues for each gene was calculated (CV = S/Xmean, where S represents the standard deviation and X mean indicates the mean expression of a gene across all the tissues). In this study, genes expressed in all tissues with a CV ≥100% as the criterion were regarded as DEGs. The log2-transformed (TPM + 1) and log2-transformed fold change values of *TaPK*s were used for heat map generation by “pheatmap” package.

### Plant material and real-time PCR analysis

Seeds of the bread wheat cultivar ‘TAM 107’ and maize inbred line B73 were surface-sterilized in 1% sodium hypochlorite for 20 min followed by six washes with distilled water, and soaked in dark overnight at room temperature. Prior to stress treatments, the germinated seeds were cultured in water and grown in a growth chamber at condition of 22 °C for 16 h of light, 18 °C for 8 h of darkness, and 50% humidity. To create drought stress, water was replaced with 20% (m/V) PEG-6000 solution and roots were totally covered by PEG solution, and the seedlings were subjected to drought stress for 1 h and 6 h, and total RNA from fresh leaf samples was isolated. The maize seeds were placed on the paper towel at 28 °C for 24 h, and then, transplanted to pots containing a mixture of vermiculite and soil (1:1, v/v). Seedings were grown in a greenhouse with 30 °C/25 °C (day/night) and 16 h/8 h (light/dark). A progressive drought stress was introduced after sowing 4 days by water deficit until 14 day, and total RNA was isolated from aerial tissues. Wheat actin (TRIAE_CS42_1AL_TGACv1_001447_AA0030680), maize ubiquitin (Zm00001d047373) and folylpolyglutamate synthase (Zm00001d048514) were respectively used as internal reference genes to normalize Ct values of each reaction [95, 96].

### Co-expression network, KEGG and GO enrichment analyses

Co-expression networks were constructed using the WGCNA package in R [97]. The modules were obtained using the step-by-step network construction method with following parameters: the power = 12, minModuleSize = 30 and mergeCutHeight = 0.25. The eigengene value was calculated for each module and used to test the association with each sample. Finally, the networks were visualized using Cytoscape 3.4.0 [98]. To further examine the functional distribution of genes in each module, BioMart data mining tool (http://plants.ensembl.org/biomart/martview/) and BlastKOALA [99] were used to obtain Gene Ontology (GO) annotation and K numbers, respectively. GO and KEGG enrichment analyses for each module were performed using the OmicShare tools (www.omicshare.com/tools), and GO terms and KEGG pathways with Q-values < 0.05 were considered statistically significant.

### Prediction of cis-regulatory elements and miRNA targets

For investigating the cis-regulatory elements, the upstream regions (1500 bp) of wheat and maize *CYP450*s were extracted using TBtools. Then the sequences were submitted to PlantCARE database to identify putative cis-regulatory elements [100]. Targets of miRNA were identified using psRNATarget server [101] with default settings (maximum expectation = 3, allowed maximum energy to unpair the target site (UPE) = 25). The available wheat and maize mature miRNA sequences (119 and 321) were downloaded from miRBase database (release 21) [102] to match against mRNA sequences of *Ta*- and *ZmCYP450s*, respectively.

## Supplementary information

**Additional file 1: Table S1.** Summary of identified *TaCYP450*s.

**Additional file 2: Table S2.** Summary of identified *ZmCYP450*s.

**Additional file 3: Figure S1.** Comparison of CYP450 families among green alga, moss, poplar, Arabidopsis, rice, wheat and maize.

**Additional file 4: Figure S2.** Schematic diagram of 30 conserved motifs in A-type P450s between wheat and maize.

**Additional file 5: Figure S3.** Thirty conserved motifs of A-type P450s.

**Additional file 6: Figure S4.** Schematic diagram of 30 conserved motifs in non-A-type P450s between wheat and maize.

**Additional file 7: Figure S5.** Thirty conserved motifs of non-A-type P450s.

**Additional file 8: Figure S6.** Phylogeny of CYP450s from *C.reinhardtii*, *P.patens*, poplar, Arabidopsis, rice, wheat and maize.

**Additional file 9: Figure S7.** Gene structure of TaCYP450s.

**Additional file 10: Figure S8.** Gene structure of ZmCYP450s.

**Additional file 11: Table S3.** The distribution of maize and wheat *CYP450*s on chromosomes.

**Additional file 12: Figure S9**. Chromosomal locations and region duplication for maize *CYP450*s. **A.** Physical map of *ZmCYP450*s. The chromosome number is indicated at the top of each chromosome. The scale bar represents the physical distance in million base pairs (Mb). The CYP450 gene clusters are indicated as vertical lines and tandemly duplicated gene pairs are colored in red. Genes lying on duplicated segments of the genome is joined by dashed lines. **B.** Circos diagram of CYP450 genes between maize and rice genomes. **a.** The chromosomes of maize and rice. **b.** The dates of the duplication events for CYP450 gene pairs. **c.** Ka/Ks ratios of duplicated CYP450 gene pairs. **d.** The distribution of *ZmCYP450*s and *OsCYP450*s on chromosomes. **e.** Colored lines represent the collinear relationships of CYP450 gene pairs between maize and rice. Colors are assigned to the syntenic regions according to the colors of the corresponding chromosomes.

**Additional file 13: Table S4.** The Ka/Ks ratios and estimated dates for the duplication events in maize and wheat CYP450 genes.

**Additional file 14: Figure S10.** The multiple sequence alignment of CYP51 members. Sites whose posterior probability is larger than cutoff value (0.67) are marked in star or triangle.

**Additional file 15: Figure S11.** The multiple sequence alignment of CYP74 members. Sites whose posterior probability is larger than cutoff value (0.67) are marked in star or triangle.

**Additional file 16: Figure S12.** The multiple sequence alignment of CYP97 members. Sites whose posterior probability is larger than cutoff value (0.67) are marked in star or triangle.

**Additional file 17: Figure S13.** Validation of structures **A.** Stereo chemical quality of the structures assessed by Ramachandran plot using RAMPAGE server. **B.** Amino acid environment assessed by ERRAT using UCLA-DOE Institute for Genomics and Proteomics Server. **C.** Superposition of 4LXJ, TaCYP51G3_2D and ZmCYP51G35; Superposition of 2RCH, TaCYP74A98_4A and ZmCYP74A39; c. Superposition of 2X2N, TaCYP97A59_6B and ZmCYP97A16.

**Additional file 18: Figure S14.** Multiple sequence alignment and secondary structure elements assignment of CYP51 members. Assignment of secondary structure elements was based on 4LXJ. *Cyan* frames localize Gotoh’s Substrate recognition sites (SRSs) 1–6 that were manually determined. *Purple* frames localize the main CYP450 motifs. The η symbol refers to a 3_10_-helix. α-helices, 3_10_-helices and π-helices are displayed as medium, small and large squiggles, respectively. β-strands are rendered as arrows, strict β-turns as TT letters and strict α-turns as TTT. White characters on the red background show strict identity. Red characters on the white background show similarity in a group, while blue frames show similarity across groups.

**Additional file 19: Figure S15.** Multiple sequence alignment and secondary structure elements assignment of CYP74 members. Assignment of secondary structure elements was based on 2RCH. *Cyan* frames localize Gotoh’s Substrate recognition sites (SRSs) 1–6 that were manually determined. *Purple* frames localize the main CYP450 motifs. The η symbol refers to a 3_10_-helix. α-helices, 3_10_-helices and π-helices are displayed as medium, small and large squiggles, respectively. β-strands are rendered as arrows, strict β-turns as TT letters and strict α-turns as TTT. White characters on the red background show strict identity. Red characters on the white background show similarity in a group, while blue frames show similarity across groups.

**Additional file 20: Figure S16.** Multiple sequence alignment and secondary structure elements assignment of CYP97 members. Assignment of secondary structure elements was based on 2X2N. *Cyan* frames localize Gotoh’s Substrate recognition sites (SRSs) 1–6 that were manually determined. *Purple* frames localize the main CYP450 motifs. The η symbol refers to a 3_10_-helix. α-helices, 3_10_-helices and π-helices are displayed as medium, small and large squiggles, respectively. β-strands are rendered as arrows, strict β-turns as TT letters and strict α-turns as TTT. White characters on the red background show strict identity. Red characters on the white background show similarity in a group, while blue frames show similarity across groups.

**Additional file 21: Table S5.** List of TPM values of 402 *TaCYP450*s in five organs at three development stages.

**Additional file 22: Table S6.** List of TPM values of 101 *ZmCYP450*s in six organs.

**Additional file 23: Figure S17.** Expression profiles of *TaCYP450*s and *ZmCYP450*s in various organs. **a.** Hierarchical clustering of the *TaCYP450*s based on log2-transformed (TPM + 1) values from digital gene expression profiling. **b.** Hierarchical clustering of *ZmCYP450*s based on log2-transformed (TPM + 1) values from digital gene expression profiling.

**Additional file 24: Table S7.** The TPM and log2FoldChange values of 119 *TaCYP450*s under drought stress.

**Additional file 25: Table S8.** The TPM and log2FoldChange values of 86 *ZmCYP450*s under drought stress.

**Additional file 26: Figure S18.** Expression profiles of *TaCYP450*s and *ZmCYP450*s under drought stress. **A. a.** Hierarchical clustering of the expression profiles of *TaCYP450*s based on log2-transformed (TPM + 1) values under drought. **b.** Heat map of log2-fold change of *TaCYP450*s. **B. a.** Hierarchical clustering of the expression profiles of *ZmCYP450*s based on log2-transformed (TPM + 1) values under drought. **b.** Heat map of log2-fold change of *ZmCYP450*s.

**Additional file 27: Table S9.** List of primers used in this study for qPCR.

**Additional file 28: Table S10.** List of 477 *TaCYP450*s in 22 modules.

**Additional file 29: Table S11.** Significantly enriched KEGG pathways in 22 modules.

**Additional file 30: Table S12.** List of predicted miRNA-target interactions.

**Additional file 31: Table S13.** List of identified wheat JA biosynthesis-related genes.

**Additional file 32: Table S14.** List of putative wheat lignin biosynthesis-related genes.

**Additional file 33: Table S15.** List of identified wheat and maize NCED genes and their TPM and log2FoldChange values under drought stress.

## Data Availability

All the datasets supporting the results of this article are included within the article and its Additional files.

## Abbreviations

ABA: Abscisic acid
BR: Brassinosteroid
CAAS: Critical amino acid site
CV: Coefficient of variation
CYP450, P450: Cytochrome P450
DEG: Differentially expressed genes
FF1: Fruit at whole plant fruit formation stage 30 to 50%
FF2: Fruit at whole plant fruit formation stage 70% to final size
FR: Fruit at whole plant fruit ripening stage
GO: Gene ontology
IFL.02: Inflorescence at 1/2 of flowers open stage
ISE.02: Inflorescence at two nodes or internodes visible stage
ISE.99: Inflorescence at maximum stem length reached stage
JA: Jasmonate
KEGG: Kyoto Encyclopedia of Genes and Genomes
L3N: Leaf at main shoot and axillary shoots visible at three nodes stage
LCE: Leaf at cotyledon emergence stage
LF1: Leaf at whole plant fruit formation stage 30 to 50%
RCE: Root at cotyledon emergence stage
RLP.03: Root at three leaves visible stage
RSE.99: Root at maximum stem length reached stage
SFL.02: Stem at 1/2 of flowers open stage
SL: Strigolactone
SRS: Substrate recognition site
SSE.00: Stem at stem elongation begins stage
SSE.02: Stem at two nodes or internodes visible stage
Ta/ZmCYP450: wheat/maize CYP450
WGCNA: Weight gene co-expression network analysis

## References

[1] Kandel S, Sauveplane V, Olry A (2006). Cytochrome P450-dependent fatty acid hydroxylases in plants. Phytochem Rev.

[2] Morant M, Schaller H, Pinot F (2007). CYP703 is an ancient cytochrome P450 in land plants catalyzing in-chain hydroxylation of Lauric acid to provide building blocks for Sporopollenin synthesis in pollen. Plant Cell.

[3] Anna AD, Jay S, Marc M, Franck P, Michiyo M, Robert S (2009). CYP704B1 is a long-chain fatty acid omega-hydroxylase essential for sporopollenin synthesis in pollen of Arabidopsis. Plant Physiol.

[4] Article LB, Vanholme R, Vanholme B, Sundin L, Goeminne G, Halpin C (2012). A systems biology view of responses to lignin biosynthesis perturbations in Arabidopsis. Plant Cell.

[5] Ueyama Y, Suzuki KI, Fukuchi-Mizutani M, Fukui Y, Miyazaki K, Ohkawa H (2002). Molecular and biochemical characterization of torenia flavonoid 3′-hydroxylase and flavone synthase II and modification of flower color by modulating the expression of these genes. Plant Sci.

[6] Andersen TB, Martinez-Swatson KA, Rasmussen SA, Boughton BA, Jørgensen K, Andersen-Ranberg J (2017). Localization and in-vivo characterization of *Thapsia garganica* CYP76AE2 indicates a role in Thapsigargin biosynthesis. Plant Physiol.

[7] Kim J, Smith JJ, Tian L (2009). The evolution and function of carotenoid hydroxylases in Arabidopsis. Plant Cell Physiol.

[8] Yoneyama K, Mori N, Sato T, Yoda A, Xie X, Okamoto M (2018). Conversion of carlactone to carlactonoic acid is a conserved function of MAX1 homologs in strigolactone biosynthesis. New Phytol.

[9] Flematti GR, Scaffidi A, Waters MT, Smith SM (2016). Stereospecificity in strigolactone biosynthesis and perception. Planta.

[10] Morrone D, Chen X, Coates RM, Peters RJ (2010). Characterization of the kaurene oxidase CYP701A3, a multifunctional cytochrome P450 from gibberellin biosynthesis. Biochem J.

[11] Heintz D, Lange T, Achard P (2014). The gibberellin biosynthetic genes AtKAO1 and AtKAO2 have overlapping roles throughout Arabidopsis development. Plant J Cell Mol Biol.

[12] Kentaro T, Tomoyuki Y, Hitoshi S (2004). Arabidopsis CYP735A1 and CYP735A2 encode cytokinin hydroxylases that catalyze the biosynthesis of trans-Zeatin. J Biol Chem.

[13] Thornton LE, Neff MM (2011). Rice CYP734A cytochrome P450s inactivate brassinosteroids in Arabidopsis. Planta.

[14] Toshiyuki O, Blanka G, Bunta W, Shozo F, Lidia H, Kouhei I (2012). CYP90A1/CPD, a brassinosteroid biosynthetic cytochrome P450 of Arabidopsis, catalyzes C-3 oxidation. J Biol Chem.

[15] Sun X, Cahill J, Van Hautegem T, Feys K, Whipple C, Nova O (2017). Altered expression of maize PLASTOCHRON1 enhances biomass and seed yield by extending cell division duration. Nat Commun.

[16] Wang X, Cheng Z, Zhao Z, Gan L, Qin R (2016). BRITTLE SHEATH1 encoding OsCYP96B4 is involved in secondary cell wall formation in rice. Plant Cell Rep.

[17] Zhao L, Cai H, Su Z, Wang L, Huang X, Zhang M (2018). KLU suppresses megasporocyte cell fate through SWR1-mediated activation of WRKY28 expression in Arabidopsis. Proc Natl Acad Sci U S A.

[18] Ma M, Wang Q, Li Z, Cheng H, Li Z, Liu X (2015). Expression of TaCYP78A3, a gene encoding cytochrome P450 CYP78A3 protein in wheat ( *Triticum aestivum* L .), affects seed size. Plant J Cell Mol Biol.

[19] Cai S, Jiang G, Ye N, Chu Z, Xu X (2015). A Key ABA Catabolic Gene , OsABA8ox3 , Is Involved in Drought Stress Resistance in Rice. PLoS One.

[20] Duan F, Ding J, Lee D, Lu X, Feng Y, Song W (2017). Overexpression of SoCYP85A1, a spinach cytochrome p450 gene in transgenic tobacco enhances root development and drought stress tolerance. Front Plant Sci.

[21] Mao G, Seebeck T, Schrenker D, Yu O (2013). CYP709B3, a cytochrome P450 monooxygenase gene involved in salt tolerance in Arabidopsis thaliana. BMC Plant Biol.

[22] Nafisi M, Goregaoker S, Botanga CJ, Glawischnig E, Olsen CE, Halkier BAGlazebrook J. (2007). Arabidopsis cytochrome P450 monooxygenase 71A13 catalyzes the conversion of indole-3-acetaldoxime in camalexin synthesis. Plant Cell.

[23] Christoph B, Lore W, Constanze S, Elke P, Dierk S, Erich G (2009). The multifunctional enzyme CYP71B15 (PHYTOALEXIN DEFICIENT3) converts cysteine-indole-3-acetonitrile to camalexin in the indole-3-acetonitrile metabolic network of Arabidopsis thaliana. Plant Cell.

[24] Papers JBC, Doi M, Mikkelsen MD, Hansen CH, Wittstock U, Halkier BA (2000). Cytochrome P450 CYP79B2 from Arabidopsis Catalyzes the Conversion of Tryptophan to Indole-3-acetaldoxime , a Precursor of Indole Glucosinolates and Indole-3-acetic Acid. J Biol Chem.

[25] Mao H, Liu J, Ren F, Peters RJ, Wang Q (2016). Characterization of CYP71Z18 indicates a role in maize zealexin biosynthesis. Phytochemistry.

[26] Irmisch S, Zeltner P, Handrick V, Gershenzon J, Köllner TG (2015). The maize cytochrome P450 CYP79A61 produces phenylacetaldoxime and indole-3-acetaldoxime in heterologous systems and might contribute to plant defense and auxin formation. BMC Plant Biol.

[27] Durst F, Nelson DR (1995). Diversity and evolution of plant P450 and P450-reductases. Drug Metabol Drug Interact.

[28] Paquette SM, Bak S, Feyereisen R (2000). Intron-exon organization and phylogeny in a large superfamily, the paralogous cytochrome P450 genes of Arabidopsis thaliana. DNA Cell Biol.

[29] Nelson DR (1999). Cytochrome P450 and the individuality of species. Arch Biochem Biophys.

[30] Xu W, Bak S, Decker A, Paquette SM, Feyereisen R, Galbraith DW (2001). Microarray-based analysis of gene expression in very large gene families: the cytochrome P450 gene superfamily of Arabidopsis thaliana. Gene..

[31] Wei K, Chen H (2018). Global identification, structural analysis and expression characterization of cytochrome P450 monooxygenase superfamily in rice. BMC Genomics.

[32] Nelson DR (2013). Cytochrome P450 genes from the sacred Lotus genome. Trop Plant Biol.

[33] Guttikonda SK, Trupti J, Bisht NC, Chen H, An YC, Pandey S (2010). Whole genome co-expression analysis of soybean cytochrome P450 genes identifies nodulation- specific P450 monooxygenases. BMC Plant Biol.

[34] Liao W, Zhao S, Zhang M, Dong K, Chen Y (2017). Transcriptome Assembly and Systematic Identification of Novel Cytochrome P450s in Taxus chinensis. Front Plant Sci.

[35] Nelson D, Werck-Reichhart D (2011). A P450-centric view of plant evolution. Plant J.

[36] Martin O, Sier-Ching C, Alain R, Matton DP (2005). Lipid signaling in plants. Cloning and expression analysis of the obtusifoliol 14alpha-demethylase from Solanum chacoense bitt., a pollination- and fertilization-induced gene with both obtusifoliol and lanosterol demethylase activity. Plant Physiol.

[37] Morikawa T, Mizutani M, Aoki N, Watanabe B, Saga H, Saito S (2006). Cytochrome P450 CYP710A encodes the sterol C-22 Desaturase in Arabidopsis and tomato. Plant Cell.

[38] Hamberger B, Bak S (2013). Plant P450s as versatile drivers for evolution of species-specific chemical diversity. Philos Trans R Soc Lond Ser B Biol Sci.

[39] Atanabe BW, Akata KS, Izutani MM, Hnishi TO (2006). CYP724B2 and CYP90B3 function in the early C-22 hydroxylation steps of Brassinosteroid biosynthetic pathway in tomato. J Agric Chem Soc Japan.

[40] Lee D, Nioche P, Hamberg M, Raman CS (2008). Structural insights into the evolutionary paths of oxylipin biosynthetic enzymes. NATURE.

[41] Qi X, Bakht S, Qin B, Leggett M, Hemmings A, Mellon F (2006). A different function for a member of an ancient and highly conserved cytochrome P450 family: from essential sterols to plant defense. Proc Natl Acad Sci U S A.

[42] Kim JE, Cheng KM, Craft NE, Hamberger B, Douglas CJ (2010). Over-expression of Arabidopsis thaliana carotenoid hydroxylases individually and in combination with a o-carotene ketolase provides insight into in vivo functions. Phytochemistry.

[43] Wasternack C, Feussner I (2018). The Oxylipin pathways: biochemistry and function. Annu Rev Plant Biol.

[44] Theodoulou FL, Job K, Slocombe SP, Footitt S, Holdsworth M, Baker A (2005). Jasmonic acid levels are reduced in COMATOSE ATP-binding cassette transporter mutants. Implications for transport of jasmonate precursors into peroxisomes. Plant Physiol.

[45] Heitz T, Widemann E, Lugan R, Miesch L, Ullmann P, Désaubry L (2012). Cytochromes P450 CYP94C1 and CYP94B3 catalyze two successive oxidation steps of plant hormone Jasmonoyl-isoleucine for catabolic turnover. J Biol Chem.

[46] Koo Abraham JK, Cooke Thomas F, Howe GA (2011). Cytochrome P450 CYP94B3 mediates catabolism and inactivation of the plant hormone jasmonoyl-L-isoleucine. Proc Natl Acad Sci U S A.

[47] Mizutani M, Ohta D (2010). Diversification of P450 genes during land plant evolution. Annu Rev Plant Biol.

[48] Ruben V, Brecht D, Kris M, John R, Wout B (2010). Lignin biosynthesis and structure. Plant Physiol.

[49] Gou M, Ran X, Martin DW, Liu CJ (2018). The scaffold proteins of lignin biosynthetic cytochrome P450 enzymes. Nat Plants.

[50] Berthet S, Thevenin J, Baratiny D, Demont-Caulet N, Debeaujon I, Bidzinski P, et al. Role of Plant Laccases in Lignin Polymerization. Chapter 5. Lignins — Biosynthesis, Biodegradation and Bioengineering, Vol 61, Advances in Botanical Research. Elsevier Science & Technology; 2012.

[51] Fagerstedt KV, Kukkola EM, Koistinen VV, Takahashi J, Marjamaa K (2010). Cell wall lignin is polymerised by class III secretable plant peroxidases in Norway spruce. J Integr Plant Biol.

[52] Fernández-pérez F, Vivar T, Pomar F, Pedre MA, Novo-uzal E (2015). Peroxidase 4 is involved in syringyl lignin formation in Arabidopsis thaliana. J Plant Physiol.

[53] Berthet S, Demont-caulet N, Pollet B, Bidzinski P, Ce L, Blondet E (2011). Disruption of LACCASE4 and 17 results in tissue-specific alterations to lignification of Arabidopsis thaliana stems. Plant Cell.

[54] Dobritsa AA, Shrestha J, Morant M, Pinot F, Matsuno M, Swanson R (2009). CYP704B1 is a long-chain fatty acid v -hydroxylase essential for Sporopollenin synthesis in pollen. Plant Physiol.

[55] Yang X, Wu D, Shi J, He Y, Pinot F, Grausem B (2014). Rice CYP703A3 , a cytochrome P450 hydroxylase , is essential for development of anther cuticle and pollen exine. J Integr Plant Biol.

[56] Hui L, Franck P, Vincent S, Danièle WR, Patrik D, Lukas S (2010). Cytochrome P450 family member CYP704B2 catalyzes the {omega}-hydroxylation of fatty acids and is required for anther cutin biosynthesis and pollen exine formation in rice. Plant Cell.

[57] Pérez-españa VH, Sánchez-león N (2011). CYP85A1 is required for the initiation of female gametogenesis in Arabidopsis thaliana. Plant Signal Behav.

[58] Davidson RM, Gowda M, Moghe G, Lin H, Vaillancourt B, Shiu S-H (2012). Comparative transcriptomics of three Poaceae species reveals patterns of gene expression evolution. Plant J.

[59] Li W, Zhou F, Liu B, Feng D, He Y, Qi K (2011). Comparative characterization, expression pattern and function analysis of the 12-oxo-phytodienoic acid reductase gene family in rice. Plant Cell Rep.

[60] Wenjuan F, Zhibiao W, Rongfeng C, Jie L, Yunhai L (2012). Maternal control of seed size by EOD3/CYP78A6 in Arabidopsis thaliana. Plant J.

[61] Mariana SS, Mara C, Anne-Laure C, Montes Ricardo AC, Lucia C, Nayelli MM (2013). Cytochrome P450 CYP78A9 is involved in Arabidopsis reproductive development. Plant Signal Behav.

[62] Nagasawa N, Hibara K, Heppard EP, Velden KA, Vander LS, Beatty M (2013). GIANT EMBRYO encodes CYP78A13 , required for proper size balance between embryo and endosperm in rice. Plant J.

[63] Kushiro T, Okamoto M, Nakabayashi K, Yamagishi K, Kitamura S, Asami T, Hirai N (2014). The Arabidopsis cytochrome P450 CYP707A encodes ABA 8′-hydroxylases: key enzymes in ABA catabolism. EMBO J.

[64] Quinlan RF, Shumskaya M, Bradbury LMT (2012). Synergistic interactions between carotene ring hydroxylases drive lutein formation in plant carotenoid biosynthesis. Plant Physiol.

[65] Savchenko T, Kolla VA, Wang C, Nasa Z, Hicks DR, Phadungchob B (2014). Functional convergence of Oxylipin and Abscisic acid pathways controls Stomatal closure in response to drought. Plant Physiol.

[66] Nakabayashi R, Yonekura-sakakibara K, Urano K, Suzuki M, Yamada Y, Nishizawa T (2014). Enhancement of oxidative and drought tolerance in Arabidopsis by overaccumulation of antioxidant flavonoids. Plant J.

[67] Lam PY, Zhu F, Chan WL, Liu H, Lo C (2014). Cytochrome P450 93G1 is a flavone synthase II that channels Flavanones to the biosynthesis of Tricin O -linked conjugates in Rice. Plant Physiol.

[68] Du Y, Chu H, Chu IK, Lo C (2010). CYP93G2 is a Flavanone 2-hydroxylase required for C -Glycosylflavone biosynthesis in Rice. Plant Physiol.

[69] Lam PY, Liu H, Lo C (2015). Completion of Tricin biosynthesis pathway in Rice: cytochrome P450 75B4 is a unique Chrysoeriol 5′-hydroxylase. Plant Physiol.

[70] Rahman MA, Akond M, Babar MA, Beecher C, Erickson J, Thomason K (2017). LC-HRMS Based Non-Targeted Metabolomic Profiling of Wheat (*Triticum aestivum* L.) under Post-Anthesis Drought Stress. Am J Plant Sci.

[71] Dutartre L, Hilliou F, Feyereisen R (2012). Phylogenomics of the benzoxazinoid biosynthetic pathway of Poaceae : gene duplications and origin of the Bx cluster. BMC Evol Biol.

[72] Nomura T, Ishihara A, Yanagita RC, Endo TR, Iwamura H (2005). Three genomes differentially contribute to the biosynthesis of benzoxazinones in hexaploid wheat. Proc Natl Acad Sci.

[73] Kersey PJ, Allen JE, Allot A, Barba M, Boddu S, Bolt BJ (2017). Ensembl genomes 2018: an integrated omics infrastructure for non-vertebrate species. Nucleic Acids Res.

[74] Bateman A, Birney E, Durbin R, Eddy SR, Howe KL, Sonnhammer ELL (2000). The Pfam protein families database. Nucleic Acids Res.

[75] Potter SC, Luciani A, Eddy SR, Park Y, Lopez R, Finn RD (2018). HMMER web server: 2018 update. Nucleic Acids Res.

[76] Sigrist CJA, Cerutti L, Hulo N, Gattiker A, Falquet L, Pagni M (2002). PROSITE: a documented database using patterns and profiles as motif descriptors. Brief Bioinform.

[77] Goldberg T, Hecht M, Hamp T, Karl T, Yachdav G, Ahmed N (2014). LocTree3 prediction of localization. Nucleic Acids Res.

[78] Kozlowski LP (2016). IPC--isoelectric point calculator. Biol Direct.

[79] Afgan E, Baker D, den Beek M, Blankenberg D, Bouvier D, Čech M (2016). The galaxy platform for accessible, reproducible and collaborative biomedical analyses: 2016 update. Nucleic Acids Res.

[80] Katoh K, Standley DM (2013). MAFFT multiple sequence alignment software version 7: improvements in performance and usability. Mol Biol Evol.

[81] Price MN, Dehal PS, Arkin AP (2010). FastTree 2--approximately maximum-likelihood trees for large alignments. PLoS One.

[82] Letunic I, Bork P (2016). Interactive tree of life (iTOL) v3: an online tool for the display and annotation of phylogenetic and other trees. Nucleic Acids Res.

[83] Chen C, Xia R, Chen H, He Y. TBtools, a Toolkit for Biologists integrating various biological data handling tools with a user-friendly interface. BioRxiv. 2018:289660. 10.1101/289660.

[84] Haug-Baltzell A, Stephens SA, Davey S, Scheidegger CE, Lyons E (2017). SynMap2 and SynMap3D: web-based whole-genome synteny browsers. Bioinformatics.

[85] Julio R, Juan CS-D, Xavier M, Ricardo R (2003). DnaSP, DNA polymorphism analyses by the coalescent and other methods. Bioinformatics.

[86] Yang Z, Gu S, Wang X, Li W, Tang Z, Xu C (2008). Molecular evolution of the CPP-like gene family in plants: insights from comparative genomics of Arabidopsis and Rice. J Mol Evol.

[87] Krzywinski M, Schein J, Birol I, Connors J, Gascoyne R, Horsman D (2009). Circos: an information aesthetic for comparative genomics. Genome Res.

[88] Xun G, Yangyun Z, Zhixi S, Wei H, Zhan Z, Zebulun A (2013). An update of DIVERGE software for functional divergence analysis of protein family. Mol Biol Evol.

[89] Robert X, Gouet P (2014). Deciphering key features in protein structures with the new ENDscript server. Nucleic Acids Res.

[90] Gotoh O (1992). Substrate recognition sites in cytochrome P450 family 2 (CYP2) proteins inferred from comparative analyses of amino acid and coding nucleotide sequences. J Biol Chem.

[91] Kelley LA, Mezulis S, Yates CM, Wass MN, Sternberg MJE (2015). The Phyre2 web portal for protein modeling, prediction and analysis. Nat Protoc.

[92] Parkinson H, Kapushesky M, Shojatalab M, Abeygunawardena N, Coulson R, Farne A (2007). ArrayExpress—a public database of microarray experiments and gene expression profiles. Nucleic Acids Res.

[93] Pertea M, Kim D, Pertea GM, Leek JT, Salzberg SL (2016). Transcript-level expression analysis of RNA-seq experiments with HISAT, StringTie and Ballgown. Nat Protoc.

[94] Yang L, Smyth Gordon K, Wei S (2014). featureCounts: an efficient general purpose program for assigning sequence reads to genomic features. Bioinformatics.

[95] Wei K, Chen H (2018). Comparative functional genomics analysis of bHLH gene family in rice , maize and wheat. BMC Plant Biol.

[96] Manoli A, Sturaro A, Trevisan S, Quaggiotti S, Nonis A (2012). Evaluation of candidate reference genes for qPCR in maize. J Plant Physiol.

[97] Langfelder P, Horvath S (2008). WGCNA: An R package for weighted correlation network analysis. BMC Bioinformatics.

[98] Shannon P, Markiel A, Ozier O, Baliga NS, Wang JT, Ramage D (2003). Cytoscape: a software environment for integrated models of biomolecular interaction networks. Genome Res.

[99] Kanehisa M, Sato Y, Morishima K (2016). BlastKOALA and GhostKOALA: KEGG tools for functional characterization of genome and metagenome sequences. J Mol Biol.

[100] Rombauts S, Déhais P, Van Montagu M, Rouzé P (1999). PlantCARE, a plant cis-acting regulatory element database. Nucleic Acids Res.

[101] Dai X, Zhao PX (2011). psRNATarget: a plant small RNA target analysis server. Nucleic Acids Res.

[102] Griffiths-Jones S (2006). miRBase: the microRNA sequence database. In: MicroRNA Protocols. Springer.

