# Supplementary material for: Comparative functional genomics analysis of cytochrome P450 gene superfamily in wheat and maize
Source: BMC Plant Biol. 2020 Mar 2;20:93. doi: 10.1186/s12870-020-2288-7 (PMC7052972; doi:10.1186/s12870-020-2288-7)
Supplement: Supplementary file 3 — Additional file 3: Figure S1. Comparison of CYP450 families among green alga, moss, poplar, Arabidopsis, rice, wheat and maize. [file 12870_2020_2288_MOESM3_ESM.pdf]

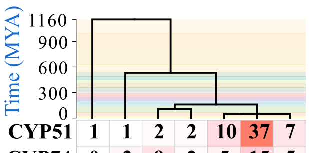

gene number (non-A type scale: 20, 40, 60, 80; A-type scale: 20, 40, 60, 80)

Type	Family	C.reinhardtii	P.patens	poplar	Arabidopsis	rice	wheat	maize	Clan
non-A type	CYP51	1	1	2	2	10	37	7	CYP51 clan
	CYP74	0	3	9	2	5	15	5	CYP74 clan
	CYP97	4	3	3	3	11	12	3	CYP97 clan
	CYP710	1	2	1	4	4	7	1	CYP710 clan
	CYP711	0	0	3	1	6	12	4	CYP711 clan
	CYP743	6	0	0	0	0	0	0	
	CYP744	7	0	0	0	0	0	0	
	CYP727	0	1	2	0	1	3	2	CYP727 clan
	CYP72	0	0	16	10	14	58	16	CYP72 clan
	CYP709	0	0	2	3	11	44	13	
	CYP714	0	0	7	2	6	22	5	
	CYP715	0	0	3	1	1	3	1	
	CYP721	0	0	11	1	2	7	2	
	CYP734	0	0	2	2	4	12	5	
	CYP735	0	0	2	4	2	6	3	
	CYP749	0	0	16	0	0	0	0	
	CYP765	0	1	0	0	0	0	0	
	CYP766	0	5	0	0	0	0	0	
	CYP85	0	0	4	1	1	14	1	CYP85 clan
	CYP87	0	0	17	1	10	29	8	
	CYP88	0	0	3	2	1	3	3	
	CYP90	0	0	9	4	6	12	5	
	CYP702	0	0	0	8	0	0	0	
	CYP707	0	0	8	4	5	6	5	
	CYP708	0	0	0	3	0	0	0	
	CYP716	0	2	25	2	0	0	0	
	CYP718	0	0	2	1	0	0	0	
	CYP720	0	0	1	1	0	0	0	
	CYP722	0	0	2	1	1	3	1	
	CYP724	0	0	2	1	1	3	1	
	CYP728	0	0	14	0	11	16	4	
	CYP729	0	0	1	0	3	9	0	
	CYP733	0	0	1	0	1	2	1	
	CYP737	1	0	0	0	0	0	0	
	CYP738	1	0	0	0	0	0	0	
	CYP739	6	0	0	0	0	0	0	
	CYP740	1	0	0	0	0	0	0	
	CYP762	0	1	0	0	0	0	0	
	CYP763	0	5	0	0	0	0	0	
	CYP86	0	2	9	10	7	16	7	CYP86 clan
	CYP94	0	4	16	6	22	46	12	
	CYP96	0	0	11	14	11	82	7	
	CYP704	0	9	9	3	7	28	5	
	CYP746	1	1	0	0	0	0	0	CYP746 clan
	CYP741	1	0	0	0	0	0	0	like animal CYP4 clan
	CYP742	1	0	0	0	0	0	0	
	CYP748	1	0	0	0	0	0	0	CYP741 clan
	CYP767	1	0	0	0	0	0	0	
	CYP768	1	0	0	0	0	0	0	
	CYP769	1	0	0	0	0	0	0	
	CYP770	1	0	0	0	0	0	0	Uncertain lateral transfer
	CYP771	1	0	0	0	0	0	0	
	CYP745	1	0	0	0	0	0	0	
	CYP747	1	0	0	0	0	0	0	
	CYP772	1	0	0	0	0	0	0	
	CYP55	1	0	0	0	0	0	0	
A-type	CYP71	0	0	54	54	80	404	56	CYP71 clan
	CYP73	0	7	4	1	4	14	3	
	CYP75	0	0	3	1	3	26	5	
	CYP76	0	0	14	9	30	59	6	
	CYP77	0	0	4	6	2	5	2	
	CYP78	0	3	13	6	8	11	8	
	CYP79	0	0	6	13	4	24	4	
	CYP80	0	0	6	0	0	0	0	
	CYP81	0	0	42	16	10	43	12	
	CYP82	0	0	30	5	0	0	0	
	CYP83	0	0	7	2	0	0	0	
	CYP84	0	0	4	1	3	27	2	
	CYP89	0	0	19	7	19	55	16	
	CYP92	0	0	13	0	9	19	11	
	CYP93	0	0	9	1	4	15	7	
	CYP98	0	1	6	3	2	10	2	
	CYP99	0	0	0	0	2	27	4	
	CYP703	0	4	2	1	1	2	1	
	CYP701	0	1	1	1	6	10	2	
	CYP705	0	0	0	31	0	0	0	
	CYP706	0	0	5	7	2	3	2	
	CYP712	0	0	15	2	0	0	0	
	CYP723	0	0	0	0	2	27	0	
	CYP736	0	0	18	0	0	0	0	
	CYP752	0	1	0	0	0	0	0	
	CYP753	0	2	0	0	0	0	0	
	CYP754	0	2	0	0	0	0	0	
	CYP755	0	1	0	0	0	0	0	
	CYP756	0	3	0	0	0	0	0	
	CYP757	0	1	0	0	0	0	0	
	CYP758	0	17	0	0	0	0	0	
	CYP759	0	3	0	0	0	0	0	
	CYP760	0	2	0	0	0	0	0	
	CYP761	0	10	0	0	0	0	0	

Figure S1. Comparison of CYP450 families among green alga, moss, poplar, Arabidopsis, rice, wheat and maize.
