# Supplementary figures and images for: Comparative functional genomics analysis of cytochrome P450 gene superfamily in wheat and maize
Source: BMC Plant Biol. 2020 Mar 2;20:93. doi: 10.1186/s12870-020-2288-7 (PMC7052972; doi:10.1186/s12870-020-2288-7)

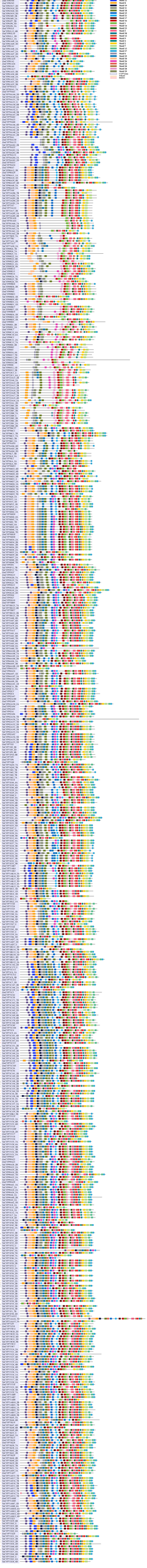

Figure S2: Schematic diagram of 30 conserved motifs in A-type P450s between wheat and maize.

Supplement: Supplementary file 4 — Additional file 4: Figure S2. Schematic diagram of 30 conserved motifs in A-type P450s between wheat and maize. [file 12870_2020_2288_MOESM4_ESM.pdf]

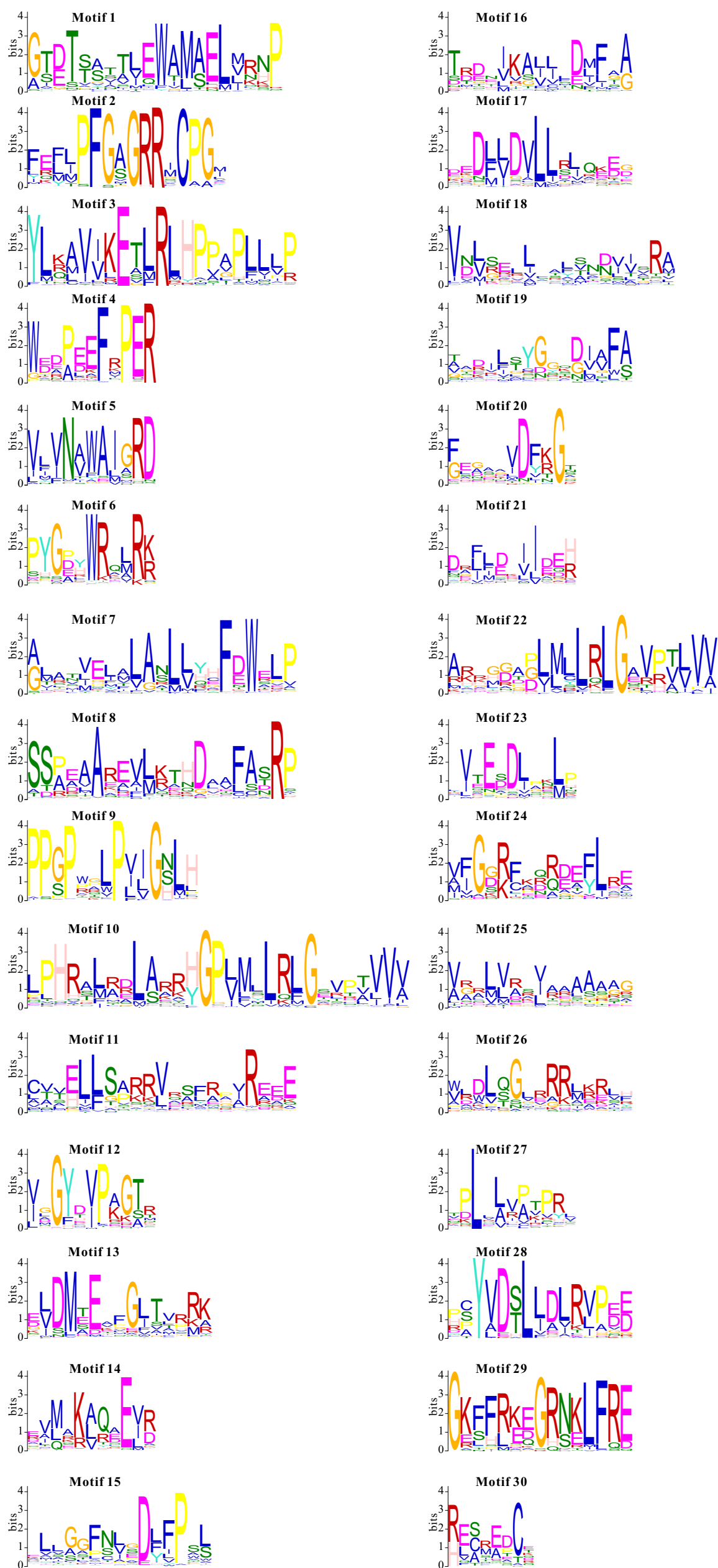

**Figure S3. Thirty conserved motifs of A-type P450s.**

Supplement: Supplementary file 5 — Additional file 5: Figure S3. Thirty conserved motifs of A-type P450s. [file 12870_2020_2288_MOESM5_ESM.pdf]

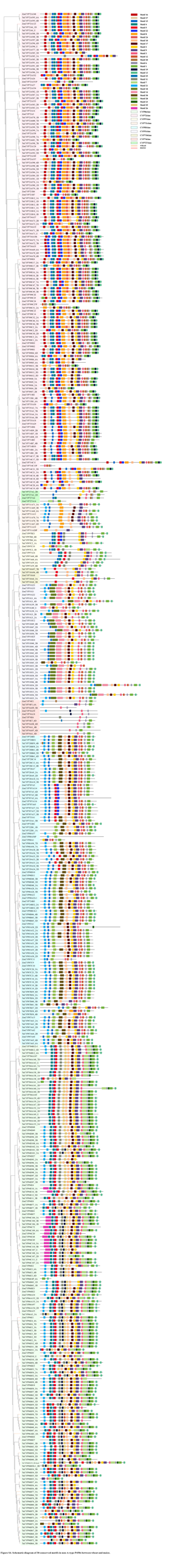

Supplement: Supplementary file 6 — Additional file 6: Figure S4. Schematic diagram of 30 conserved motifs in non-A-type P450s between wheat and maize. [file 12870_2020_2288_MOESM6_ESM.pdf]

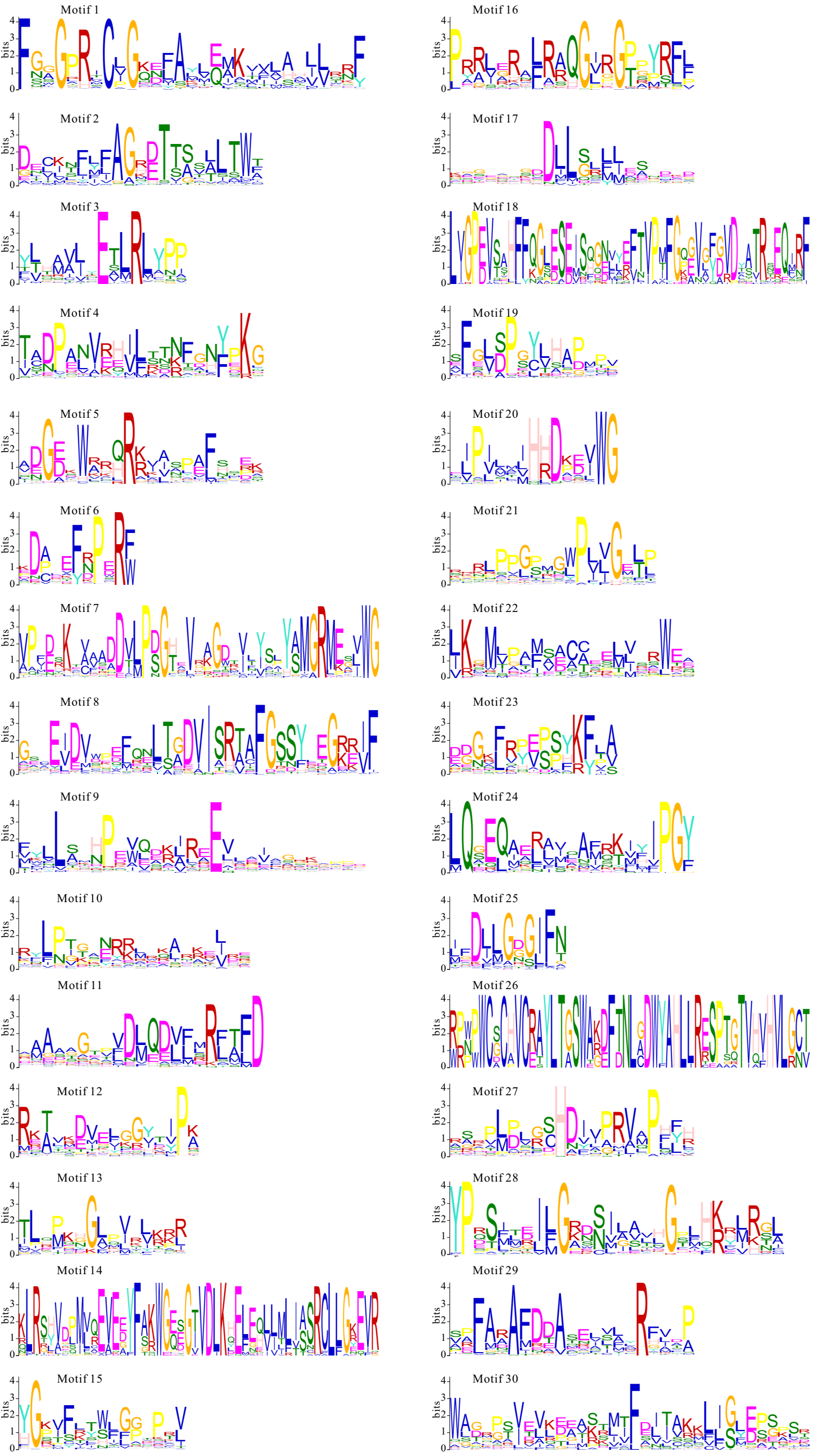

Figure S5. Thirty conserved motifs of non-A-type P450s.

Supplement: Supplementary file 7 — Additional file 7: Figure S5. Thirty conserved motifs of non-A-type P450s. [file 12870_2020_2288_MOESM7_ESM.pdf]

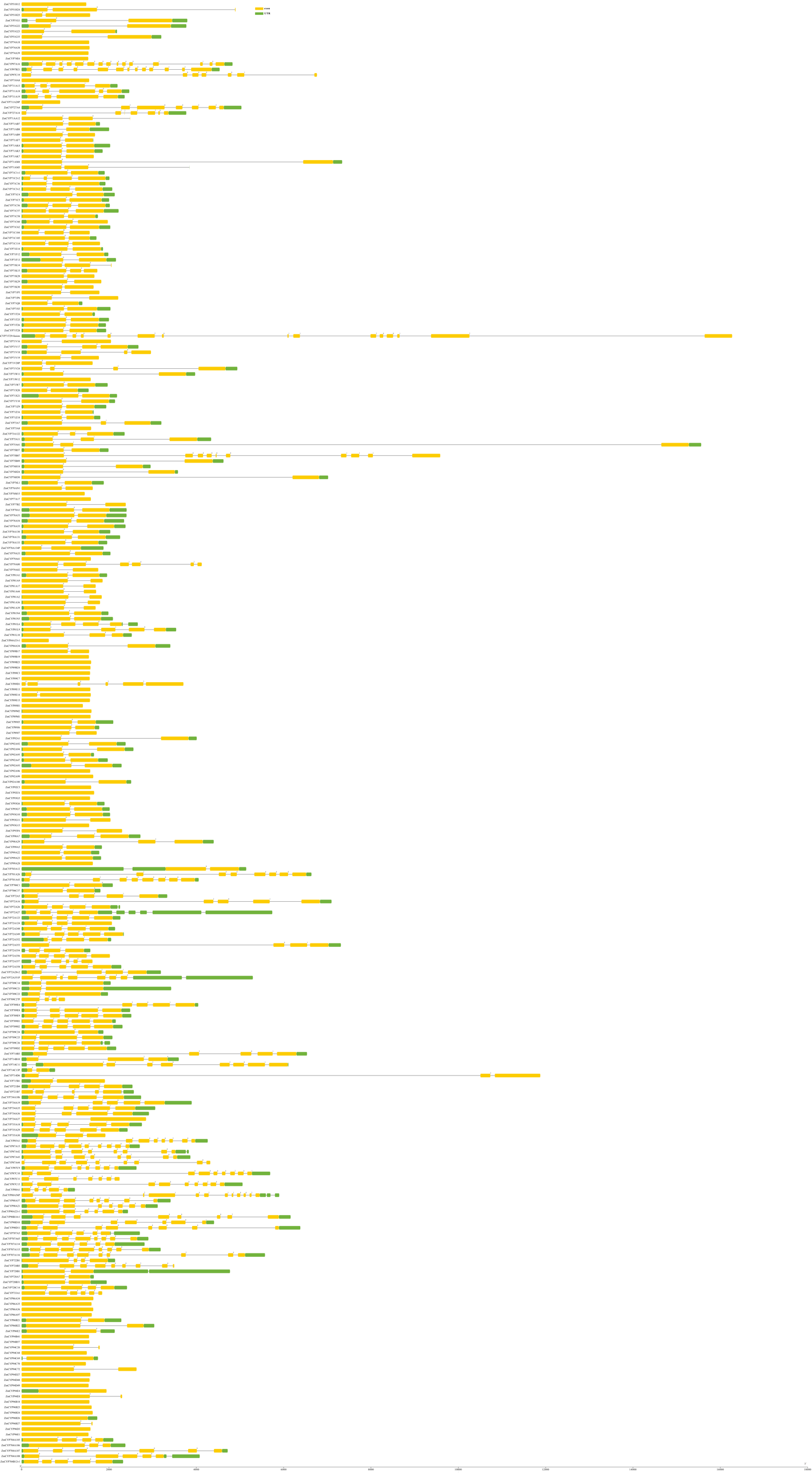

Figure S8. Gene structure of ZmCYF480b.

Supplement: Supplementary file 10 — Additional file 10: Figure S8. Gene structure of ZmCYP450s. [file 12870_2020_2288_MOESM10_ESM.pdf]

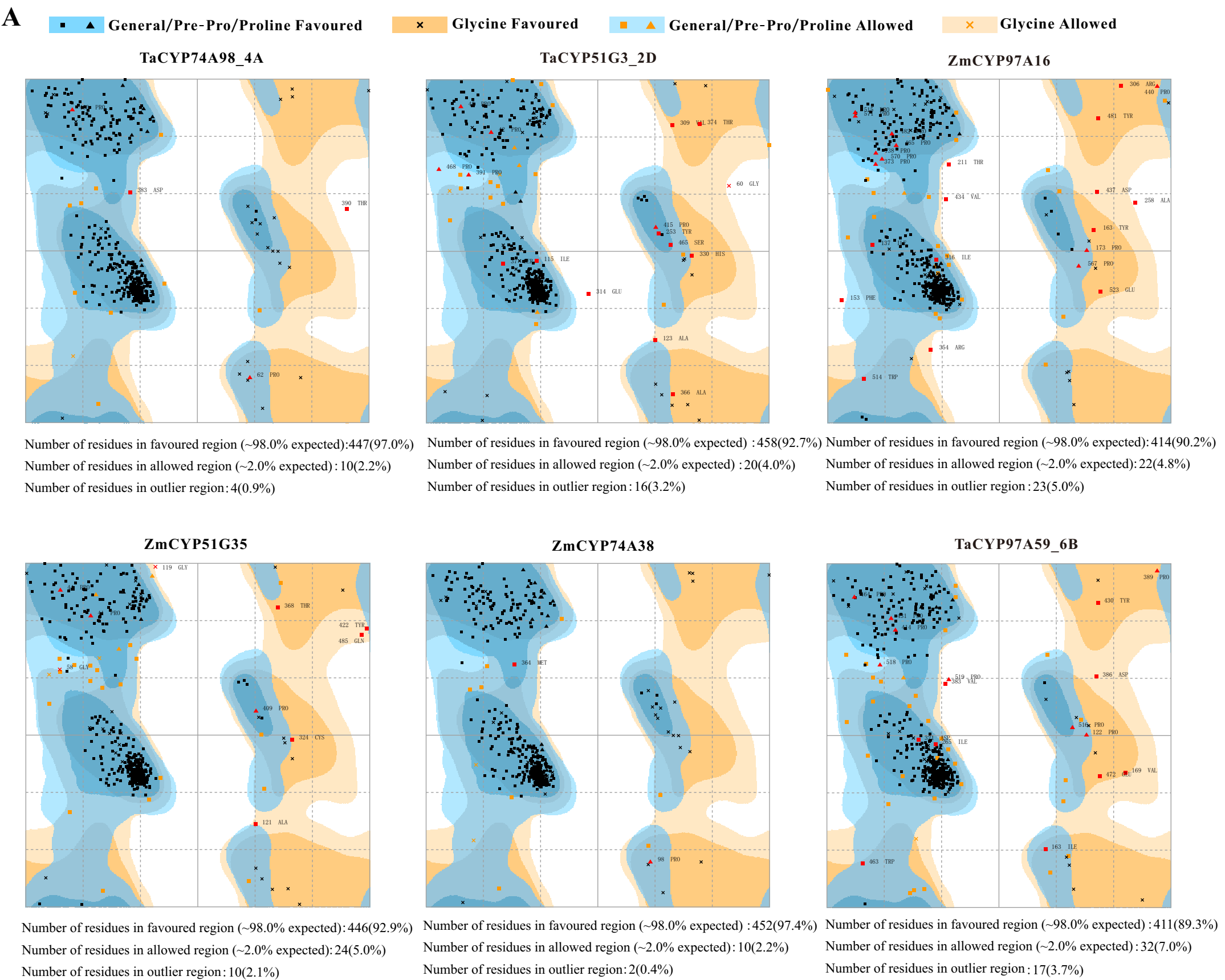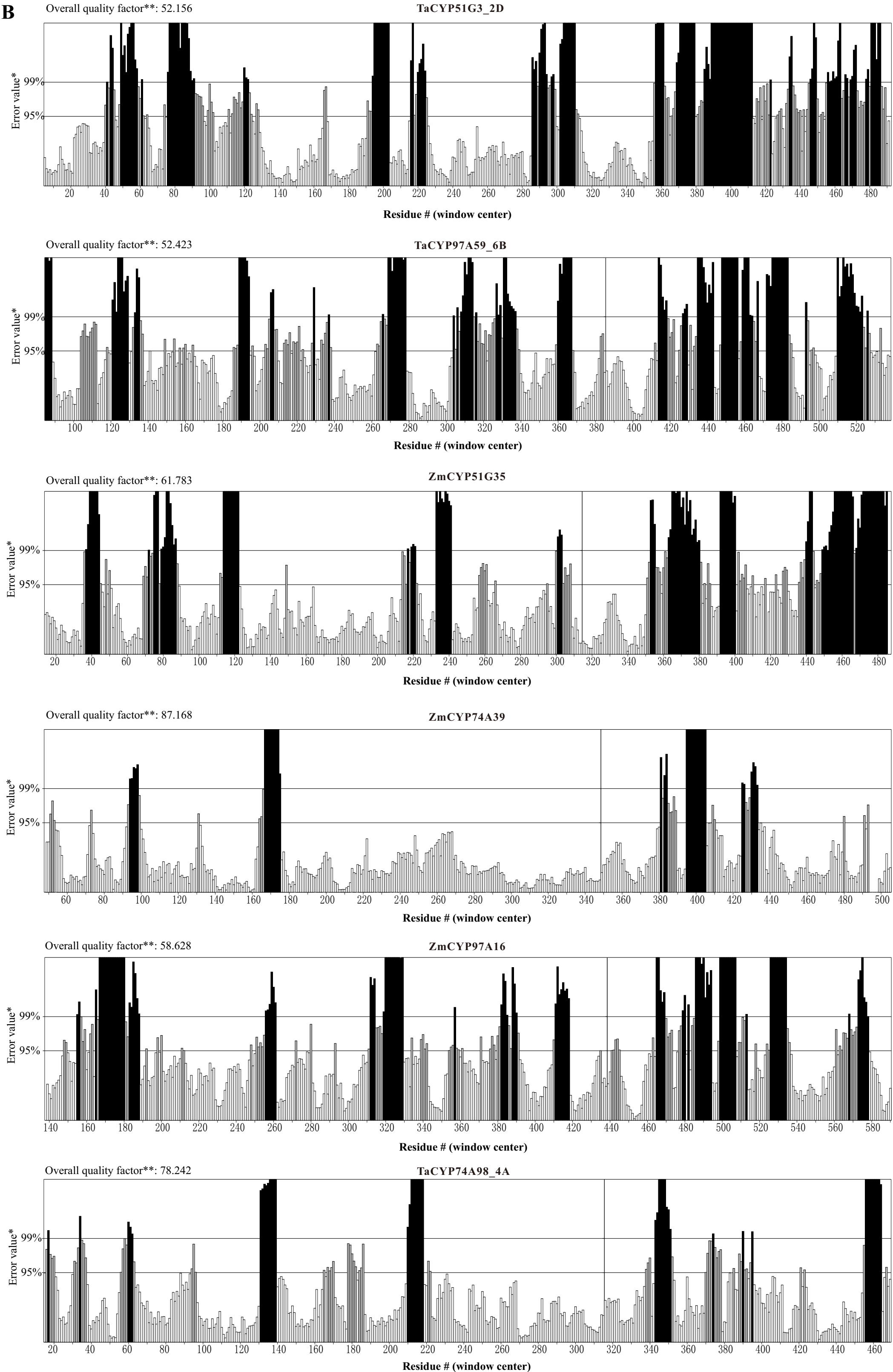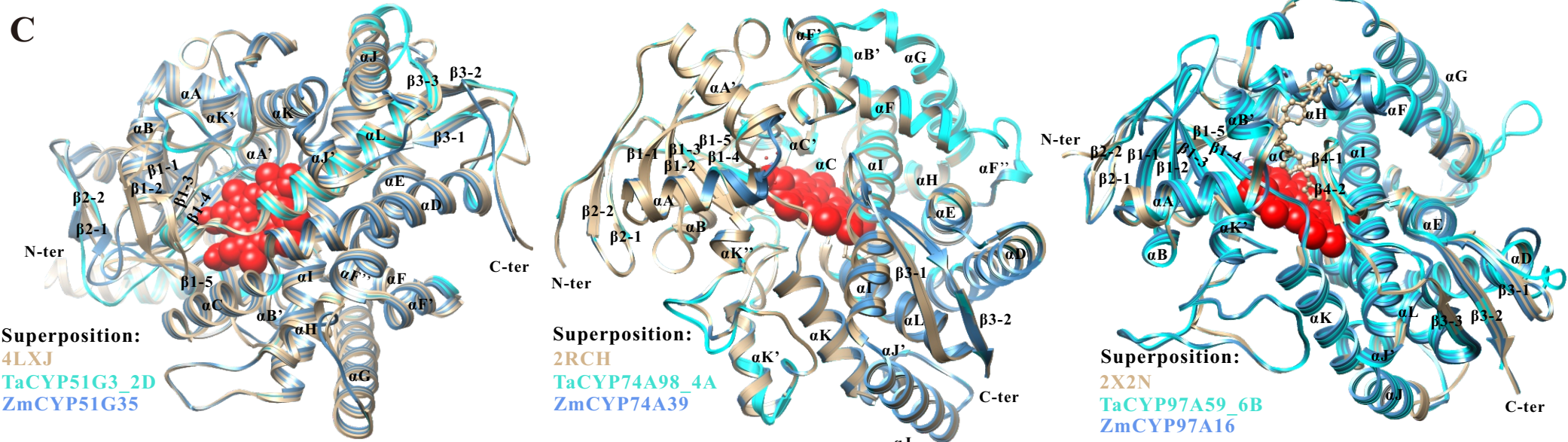

Supplement: Supplementary file 17 — Additional file 17: Figure S13. Validation of structures A. Stereo chemical quality of the structures assessed by Ramachandran plot using RAMPAGE server. B. Amino acid environment assessed by ERRAT using UCLA-DOE Institute for Genomics and Proteomics Server. C. Superposition of 4LXJ, TaCYP51G3_2D and ZmCYP51G35; Superposition of 2RCH, TaCYP74A98_4A and ZmCYP74A39; c. Superposition of 2X2N, TaCYP97A59_6B and ZmCYP97A16. [file 12870_2020_2288_MOESM17_ESM.pdf]
