# Supplementary material for: Comparative functional genomics analysis of cytochrome P450 gene superfamily in wheat and maize
Source: BMC Plant Biol. 2020 Mar 2;20:93. doi: 10.1186/s12870-020-2288-7 (PMC7052972; doi:10.1186/s12870-020-2288-7)
Supplement: Supplementary file 12 — Additional file 12: Figure S9. Chromosomal locations and region duplication for maize CYP450s. A. Physical map of ZmCYP450s. The chromosome number is indicated at the top of each chromosome. The scale bar represents the physical distance in million base pairs (Mb). The CYP450 gene clusters are indicated as vertical lines and tandemly duplicated gene pairs are colored in red. Genes lying on duplicated segments of the genome is joined by dashed lines. B. Circos diagram of CYP450 genes between maize and rice genomes. a. The chromosomes of maize and rice. b. The dates of the duplication events for CYP450 gene pairs. c. Ka/Ks ratios of duplicated CYP450 gene pairs. d. The distribution of ZmCYP450s and OsCYP450s on chromosomes. e. Colored lines represent the collinear relationships of CYP450 gene pairs between maize and rice. Colors are assigned to the syntenic regions according to the colors of the corresponding chromosomes. [file 12870_2020_2288_MOESM12_ESM.pdf]

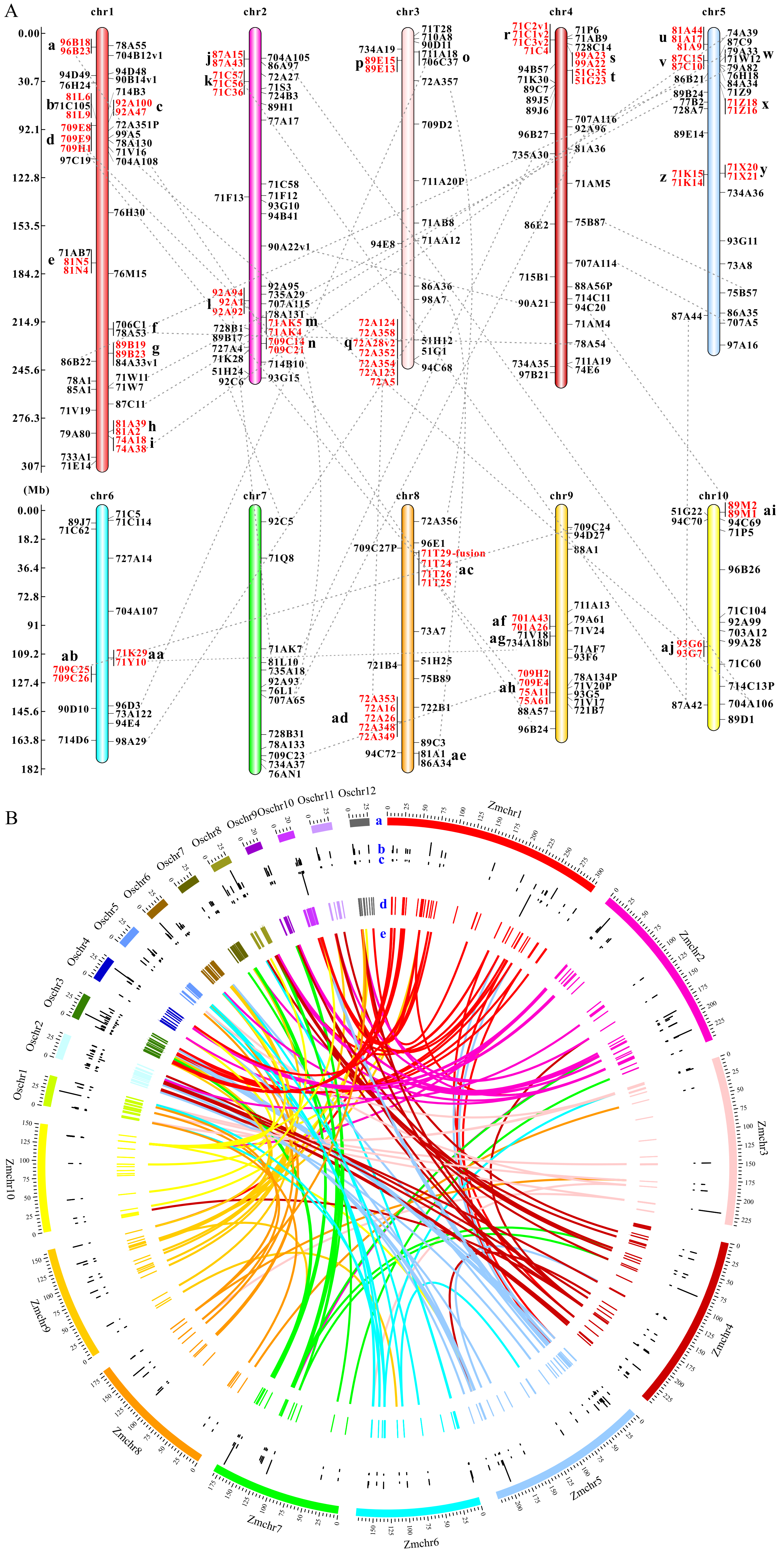


**Figure S9. Chromosomal locations and region duplication for maize CYP450s.** A. Physical map of ZmCYP450s. The chromosome number is indicated at the top of each chromosome. The scale bar represents the physical distance in million base pairs (Mb). The CYP450 gene clusters are indicated as vertical lines and tandemly duplicated gene pairs are colored in red. Genes lying on duplicated segments of the genome is joined by dashed lines. B. Circos diagram of CYP450 genes between maize and rice genomes. a. The chromosomes of maize and rice. b. The dates of the duplication events for CYP450 gene pairs. c. Ka/Ks ratios of duplicated CYP450 gene pairs. d. The distribution of ZmCYP450s and OsCYP450s on chromosomes. e. Colored lines represent the collinear relationships of CYP450 gene pairs between maize and rice. Colors are assigned to the syntenic regions according to the colors of the corresponding chromosomes.
