# Supplementary material for: Comparative functional genomics analysis of cytochrome P450 gene superfamily in wheat and maize
Source: BMC Plant Biol. 2020 Mar 2;20:93. doi: 10.1186/s12870-020-2288-7 (PMC7052972; doi:10.1186/s12870-020-2288-7)
Supplement: Supplementary file 18 — Additional file 18: Figure S14. Multiple sequence alignment and secondary structure elements assignment of CYP51 members. Assignment of secondary structure elements was based on 4LXJ. Cyan frames localize Gotoh’s Substrate recognition sites (SRSs) 1–6 that were manually determined. Purple frames localize the main CYP450 motifs. The η symbol refers to a 310-helix. α-helices, 310-helices and π-helices are displayed as medium, small and large squiggles, respectively. β-strands are rendered as arrows, strict β-turns as TT letters and strict α-turns as TTT. White characters on the red background show strict identity. Red characters on the white background show similarity in a group, while blue frames show similarity across groups. [file 12870_2020_2288_MOESM18_ESM.pdf]

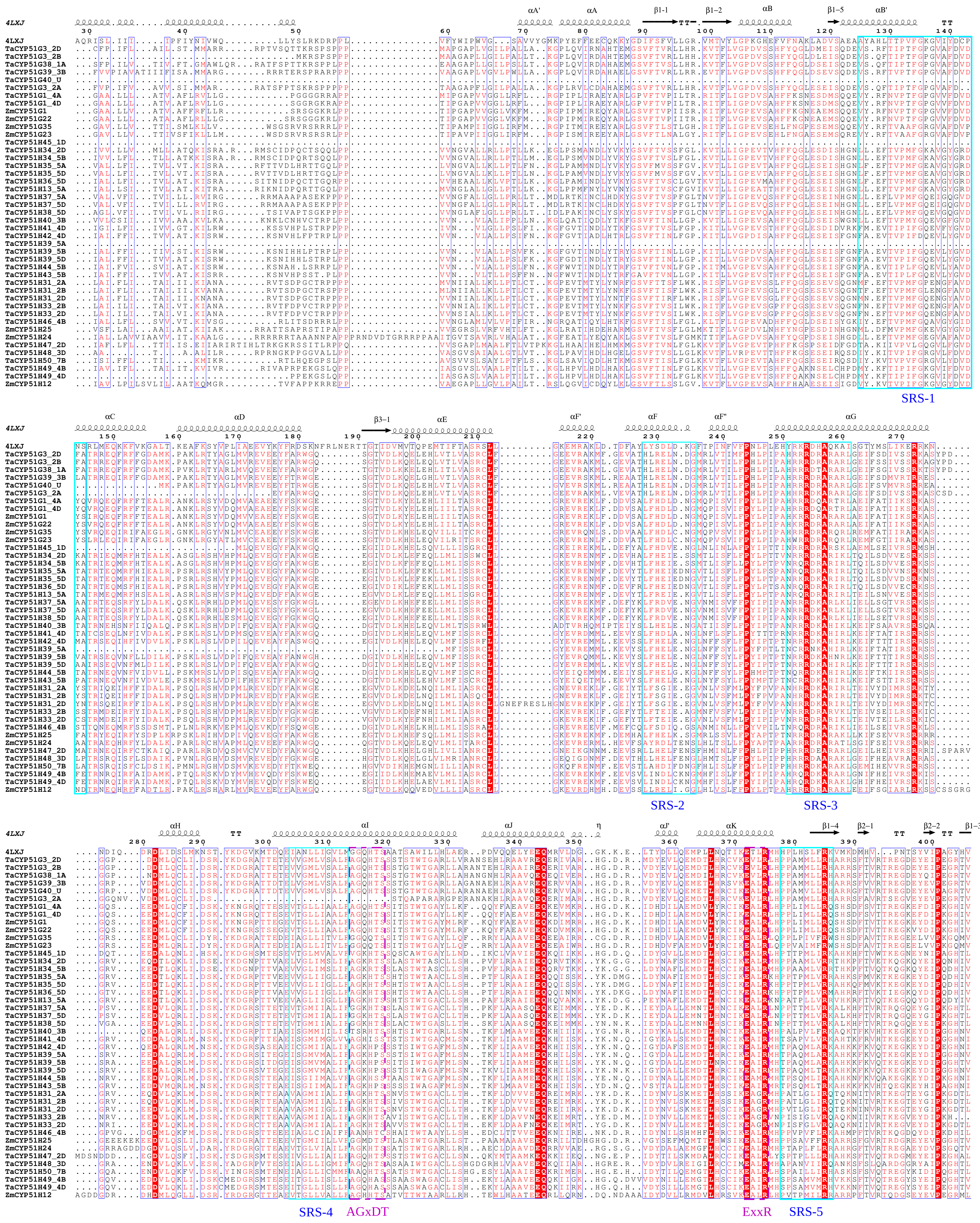
4LXJ
SRS-1
SRS-2
SRS-3
SRS-4
AGxDT
ExxR
SRS-5

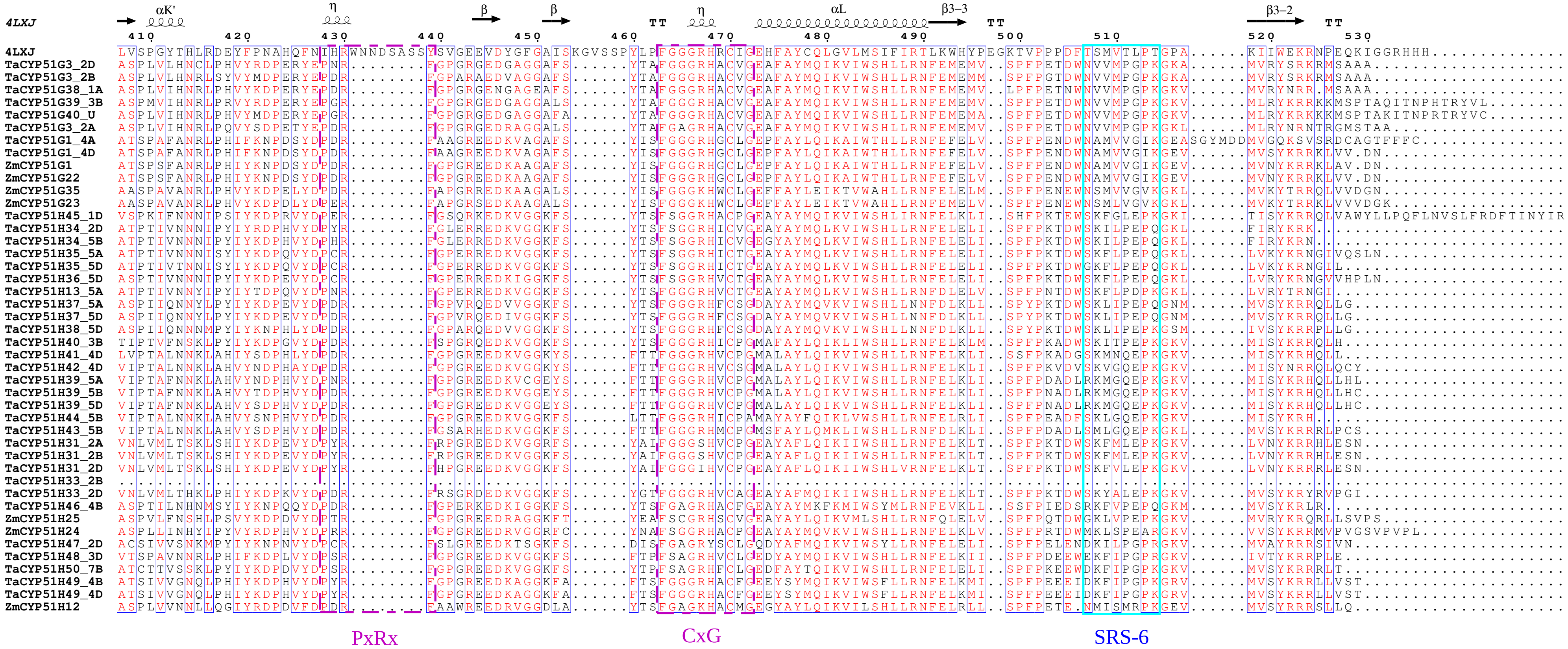

Figure S14. Multiple sequence alignment and secondary structure assignment of CYP51 members. Assignment of secondary structure elements was based on 4LXJ. *Cyan* frames localize Gotoh's Substrate recognition sites (SRSs) 1–6 that were manually determined. *Purple* frames localize the main CYP450 motifs.The η symbol refers to a 3_{10}-helix. α-helices, 3_{10}-helices and π-helices are displayed as medium, small and large squiggles, respectively. β-strands are rendered as arrows, strict β-turns as TT letters and strict α-turns as TTT. White characters on the red background show strict identity. Red characters on the white background show similarity in a group, while blue frames show similarity across groups.
