# Supplementary material for: Comparative functional genomics analysis of cytochrome P450 gene superfamily in wheat and maize
Source: BMC Plant Biol. 2020 Mar 2;20:93. doi: 10.1186/s12870-020-2288-7 (PMC7052972; doi:10.1186/s12870-020-2288-7)
Supplement: Supplementary file 19 — Additional file 19: Figure S15. Multiple sequence alignment and secondary structure elements assignment of CYP74 members. Assignment of secondary structure elements was based on 2RCH. Cyan frames localize Gotoh’s Substrate recognition sites (SRSs) 1–6 that were manually determined. Purple frames localize the main CYP450 motifs. The η symbol refers to a 310-helix. α-helices, 310-helices and π-helices are displayed as medium, small and large squiggles, respectively. β-strands are rendered as arrows, strict β-turns as TT letters and strict α-turns as TTT. White characters on the red background show strict identity. Red characters on the white background show similarity in a group, while blue frames show similarity across groups. [file 12870_2020_2288_MOESM19_ESM.pdf]

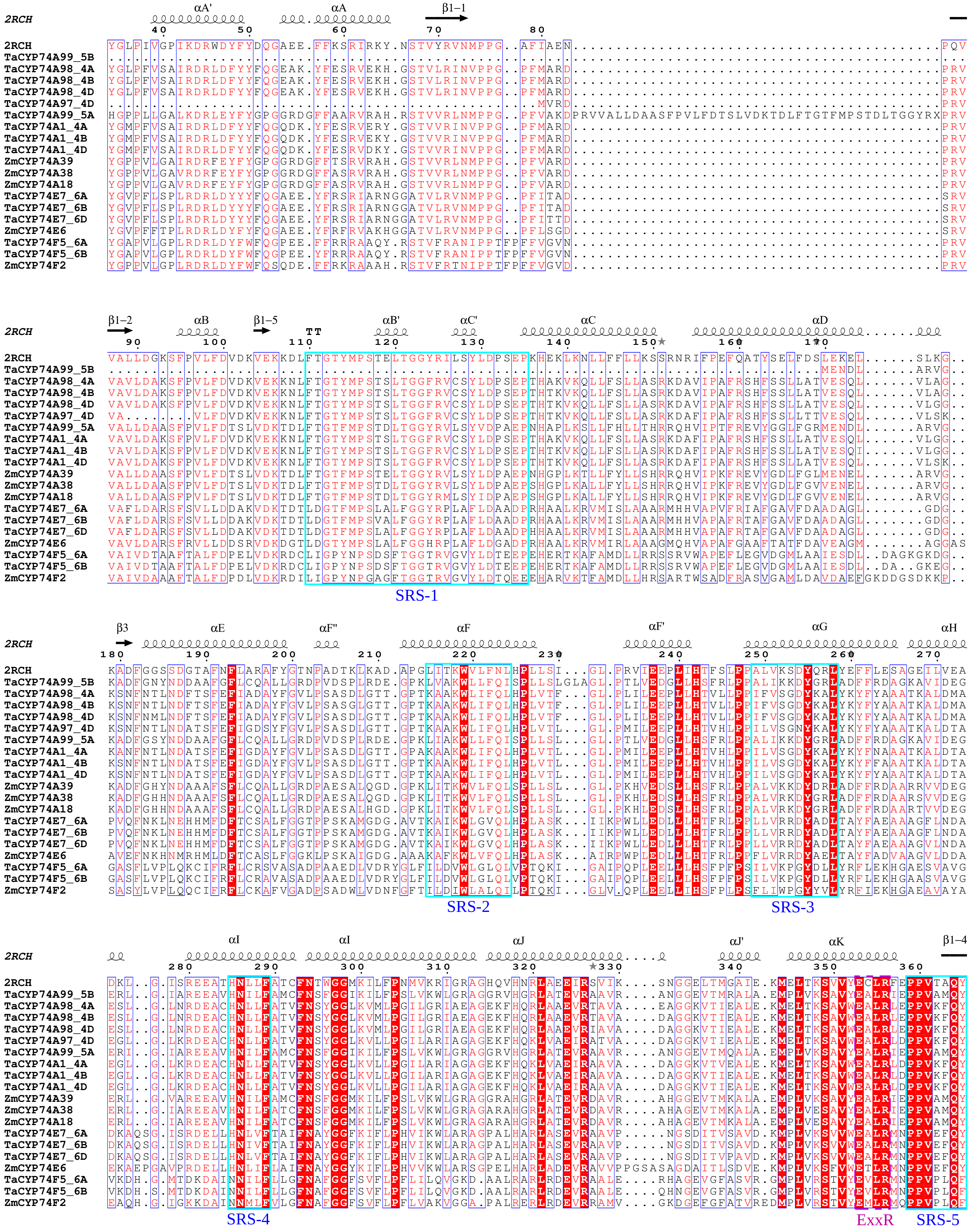

2RCH
TaCYP74A99_5B
TaCYP74A98_4A
TaCYP74A98_4B
TaCYP74A98_4D
TaCYP74A97_4D
TaCYP74A99_5A
TaCYP74A1_4A
TaCYP74A1_4B
TaCYP74A1_4D
ZmCYP74A39
ZmCYP74A38
ZmCYP74A18
TaCYP74E7_6A
TaCYP74E7_6B
TaCYP74E7_6D
ZmCYP74E6
TaCYP74F5_6A
TaCYP74F5_6B
ZmCYP74F2
αA'
αA
β1–1
β1–2
αB
β1–5
TT
αB'
αC'
αC
αD
β3
αE
αF''
αF
αF'
αG
αH
αI
αJ
αJ'
αK
β1–4
SRS-1
SRS-2
SRS-3
SRS-4
SRS-5
ExxR

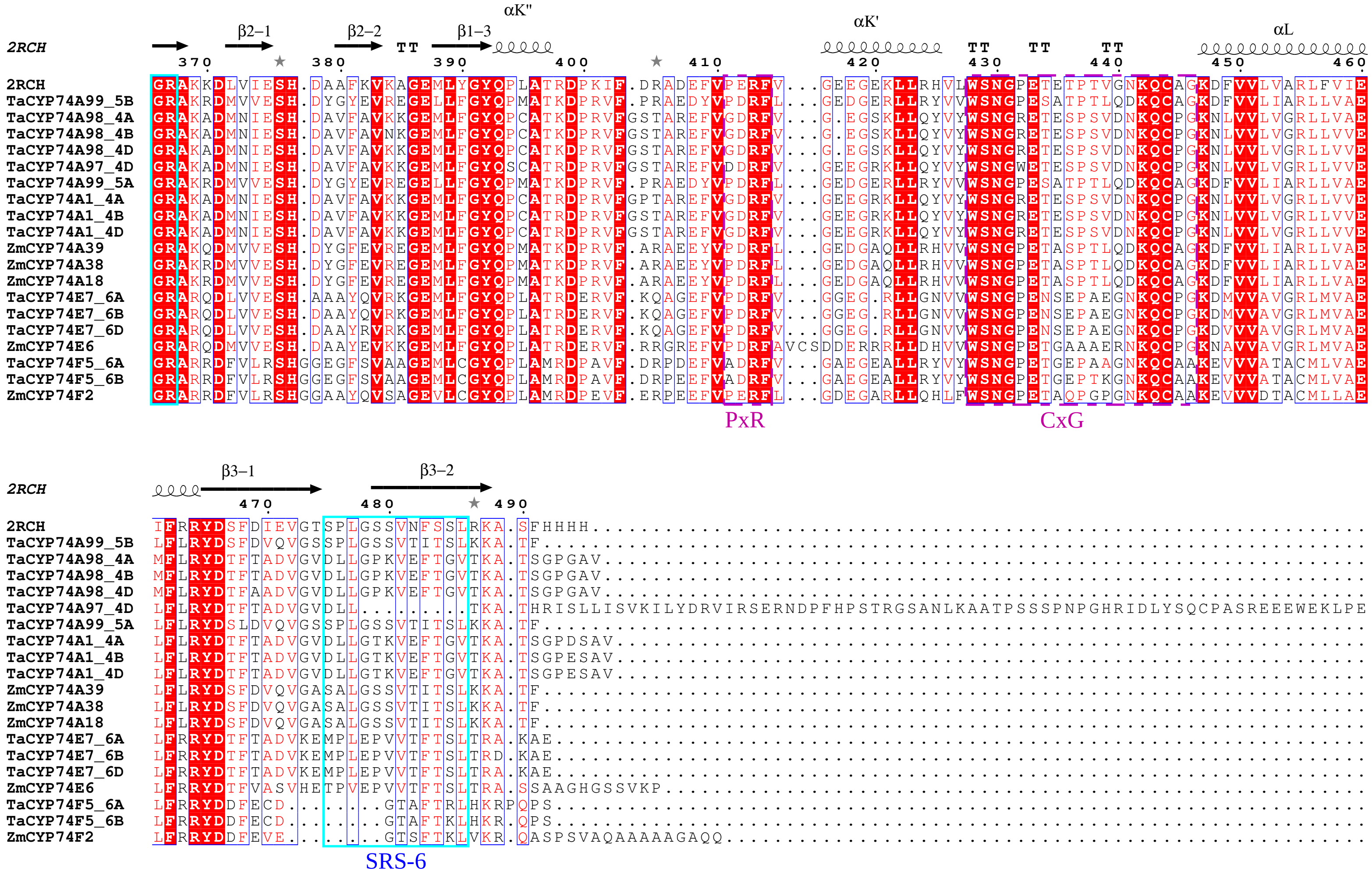

Figure S15. Multiple sequence alignment and secondary structure assignment of CYP74 members. Assignment of secondary structure elements was based on 2RCH. *Cyan* frames localize Gotoh's Substrate recognition sites (SRSs) 1–6 that were manually determined. *Purple* frames localize the main CYP450 motifs.The η symbol refers to a 3_{10}-helix. α-helices, 3_{10}-helices and π-helices are displayed as medium, small and large squiggles, respectively. β-strands are rendered as arrows, strict β-turns as TT letters and strict α-turns as TTT. White characters on the red background show strict identity. Red characters on the white background show similarity in a group, while blue frames show similarity across groups.
