# Supplementary material for: Comparative functional genomics analysis of cytochrome P450 gene superfamily in wheat and maize
Source: BMC Plant Biol. 2020 Mar 2;20:93. doi: 10.1186/s12870-020-2288-7 (PMC7052972; doi:10.1186/s12870-020-2288-7)
Supplement: Supplementary file 20 — Additional file 20: Figure S16. Multiple sequence alignment and secondary structure elements assignment of CYP97 members. Assignment of secondary structure elements was based on 2X2N. Cyan frames localize Gotoh’s Substrate recognition sites (SRSs) 1–6 that were manually determined. Purple frames localize the main CYP450 motifs. The η symbol refers to a 310-helix. α-helices, 310-helices and π-helices are displayed as medium, small and large squiggles, respectively. β-strands are rendered as arrows, strict β-turns as TT letters and strict α-turns as TTT. White characters on the red background show strict identity. Red characters on the white background show similarity in a group, while blue frames show similarity across groups. [file 12870_2020_2288_MOESM20_ESM.pdf]

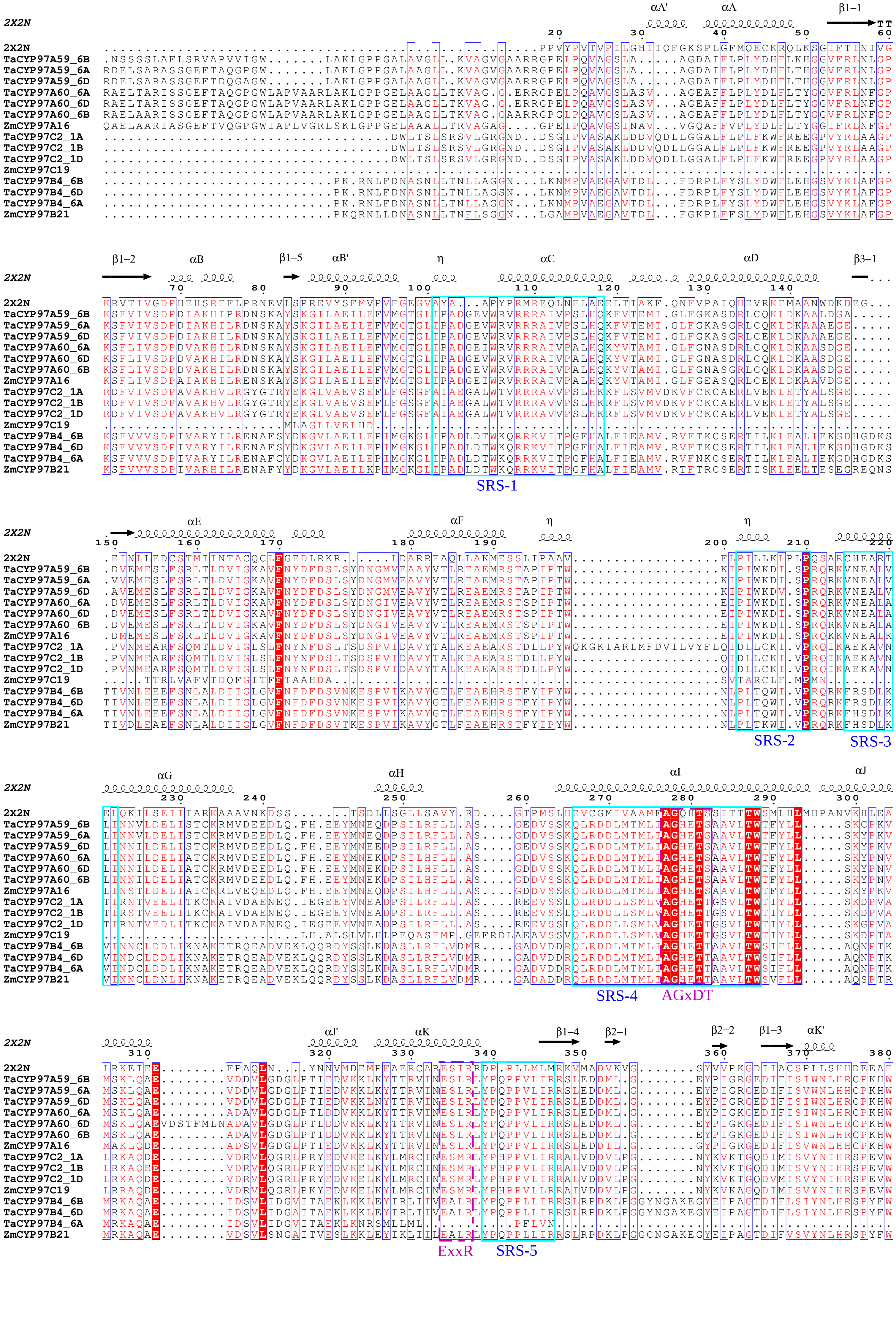
2X2N
TaCYP97A59_6B
TaCYP97A59_6A
TaCYP97A59_6D
TaCYP97A60_6A
TaCYP97A60_6D
TaCYP97A60_6B
ZmCYP97A16
TaCYP97C2_1A
TaCYP97C2_1B
TaCYP97C2_1D
ZmCYP97C19
TaCYP97B4_6B
TaCYP97B4_6D
TaCYP97B4_6A
ZmCYP97B21
αA'
αA
β1–1
β1–2
αB
β1–5
αB'
η
αC
αD
β3–1
αE
αF
αG
αH
αI
αJ
αJ'
αK
β1–4
β2–1
β2–2
β1–3
αK'
SRS-1
SRS-2
SRS-3
SRS-4
SRS-5
AGxDT
ExxR

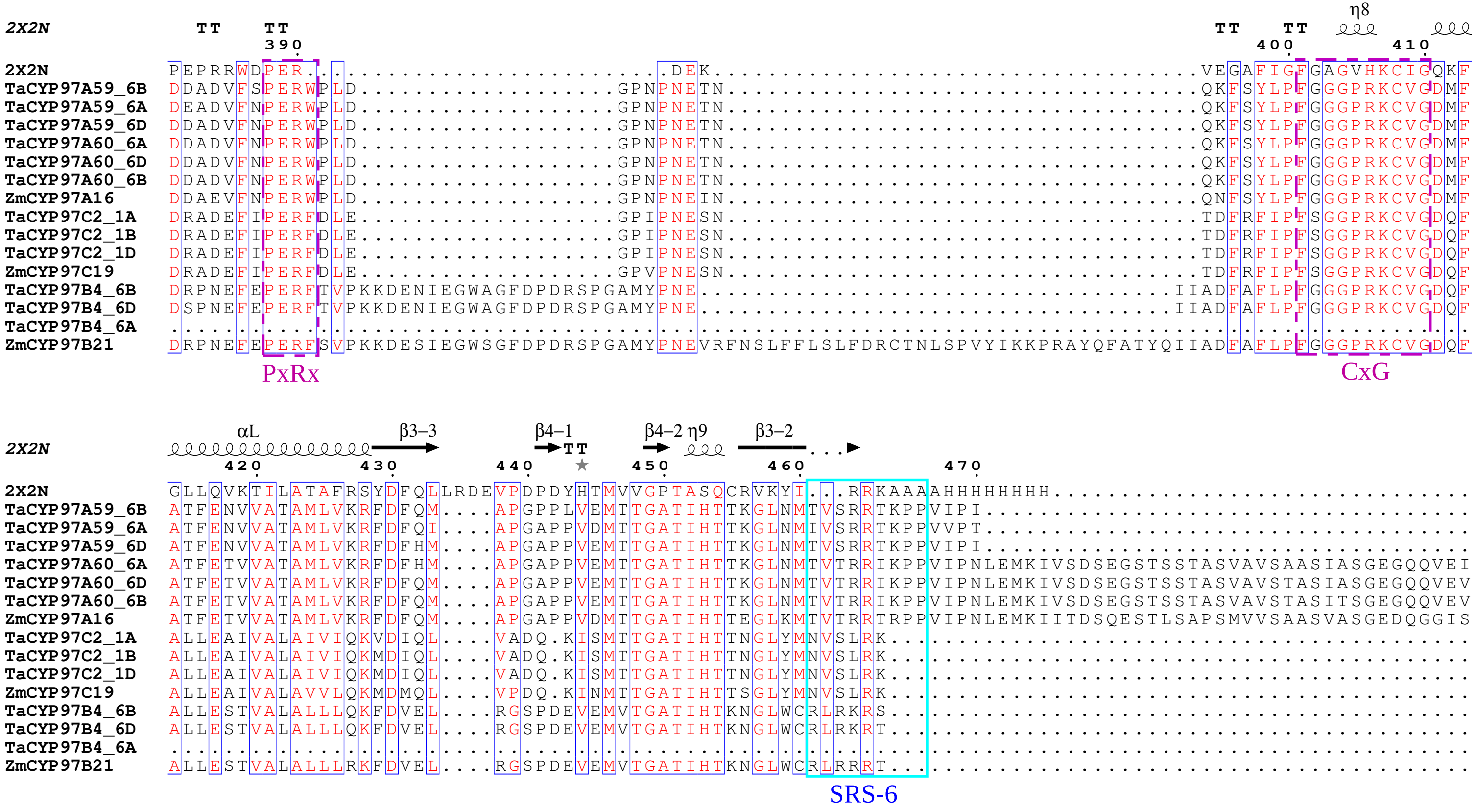

Figure S16. Multiple sequence alignment and secondary structure assignment of CYP97 members. Assignment of secondary structure elements was based on 2X2N. *Cyan* frames localize Gotoh's Substrate recognition sites (SRSs) 1–6 that were manually determined. *Purple* frames localize the main CYP450 motifs. The η symbol refers to a 3_{10}-helix. α-helices, 3_{10}-helices and π-helices are displayed as medium, small and large squiggles, respectively. β-strands are rendered as arrows, strict β-turns as TT letters and strict α-turns as TTT. White characters on the red background show strict identity. Red characters on the white background show similarity in a group, while blue frames show similarity across groups.
