# Supplementary material for: Comparative functional genomics analysis of cytochrome P450 gene superfamily in wheat and maize
Source: BMC Plant Biol. 2020 Mar 2;20:93. doi: 10.1186/s12870-020-2288-7 (PMC7052972; doi:10.1186/s12870-020-2288-7)
Supplement: Supplementary file 23 — Additional file 23: Figure S17. Expression profiles of TaCYP450s and ZmCYP450s in various organs. a. Hierarchical clustering of the TaCYP450s based on log2-transformed (TPM + 1) values from digital gene expression profiling. b. Hierarchical clustering of ZmCYP450s based on log2-transformed (TPM + 1) values from digital gene expression profiling. [file 12870_2020_2288_MOESM23_ESM.pdf]

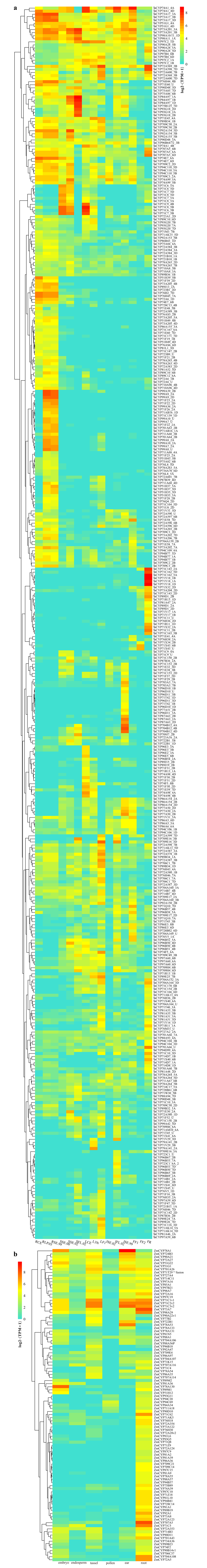

Figure S17. Expression profiles of *TaCYP450*s and *ZmCYP450*s in various organs. a. Hierarchical clustering of the *TaCYP450*s based on log2-transformed (TPM+1) values from digital gene expression profiling. **b.** Hierarchical clustering of *ZmCYP450*s based on log2-transformed (TPM+1) values from digital gene expression profiling.
