# Supplementary material for: Comparative functional genomics analysis of cytochrome P450 gene superfamily in wheat and maize
Source: BMC Plant Biol. 2020 Mar 2;20:93. doi: 10.1186/s12870-020-2288-7 (PMC7052972; doi:10.1186/s12870-020-2288-7)
Supplement: Supplementary file 26 — Additional file 26: Figure S18. Expression profiles of TaCYP450s and ZmCYP450s under drought stress. A. a. Hierarchical clustering of the expression profiles of TaCYP450s based on log2-transformed (TPM + 1) values under drought. b. Heat map of log2-fold change of TaCYP450s. B. a. Hierarchical clustering of the expression profiles of ZmCYP450s based on log2-transformed (TPM + 1) values under drought. b. Heat map of log2-fold change of ZmCYP450s. [file 12870_2020_2288_MOESM26_ESM.pdf]

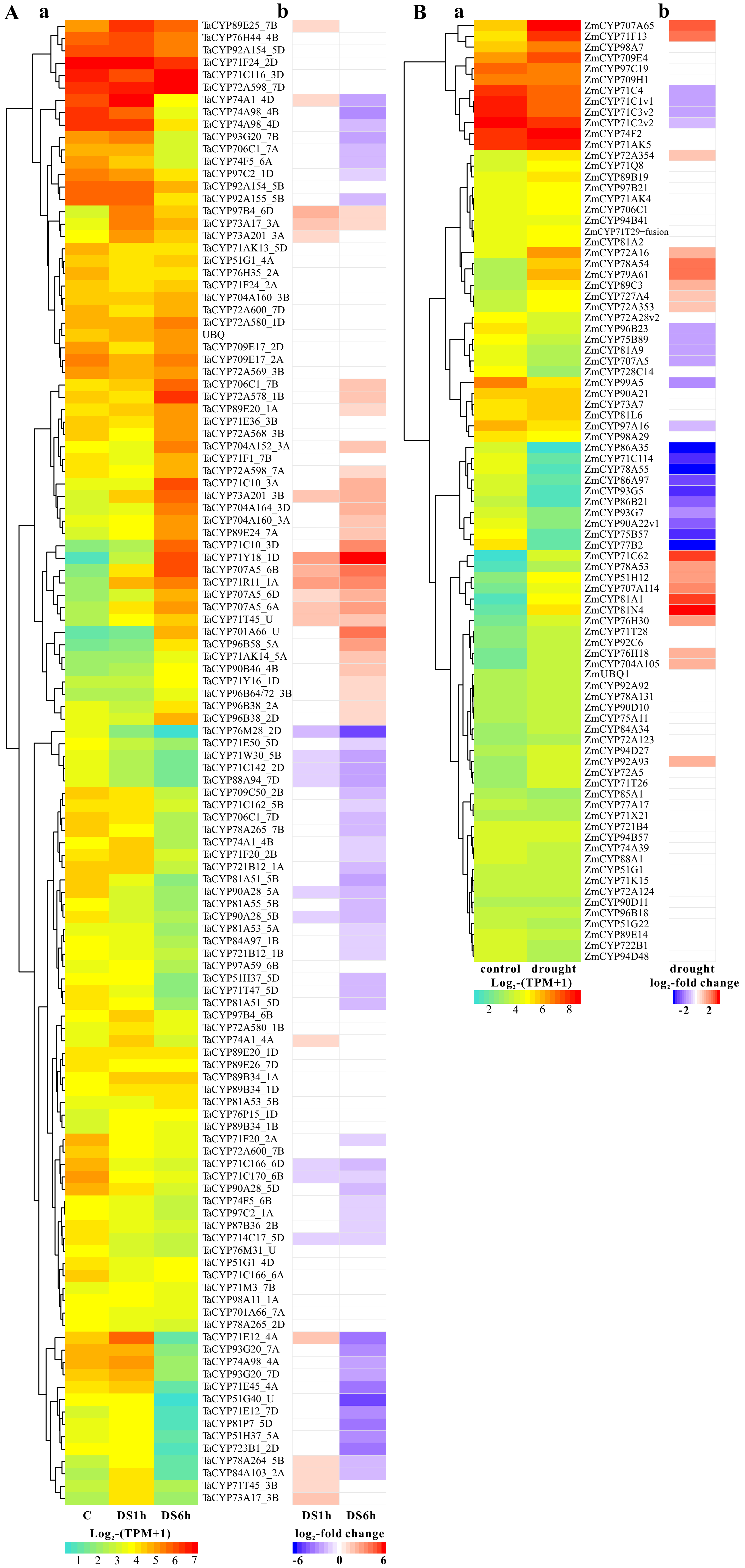


**Figure S18. Expression profiles of *TaCYP450* s and *ZmCYP450* s under drought stress.** A. a. Hierarchical clustering of the expression profiles of *TaCYP450*s based on log2-transformed (TPM+1) values under drought. b. Heat map of log2-fold change of *TaCYP450*s. B. a. Hierarchical clustering of the expression profiles of *ZmCYP450*s based on log2-transformed (TPM+1) values under drought. b. Heat map of log2-fold change of *ZmCYP450*s.
