# Supplementary material for: Comparative functional genomics analysis of cytochrome P450 gene superfamily in wheat and maize
Source: BMC Plant Biol. 2020 Mar 2;20:93. doi: 10.1186/s12870-020-2288-7 (PMC7052972; doi:10.1186/s12870-020-2288-7)
Supplement: Supplementary file 8 — Additional file 8: Figure S6. Phylogeny of CYP450s from C.reinhardtii, P.patens, poplar, Arabidopsis, rice, wheat and maize. [file 12870_2020_2288_MOESM8_ESM.pdf]

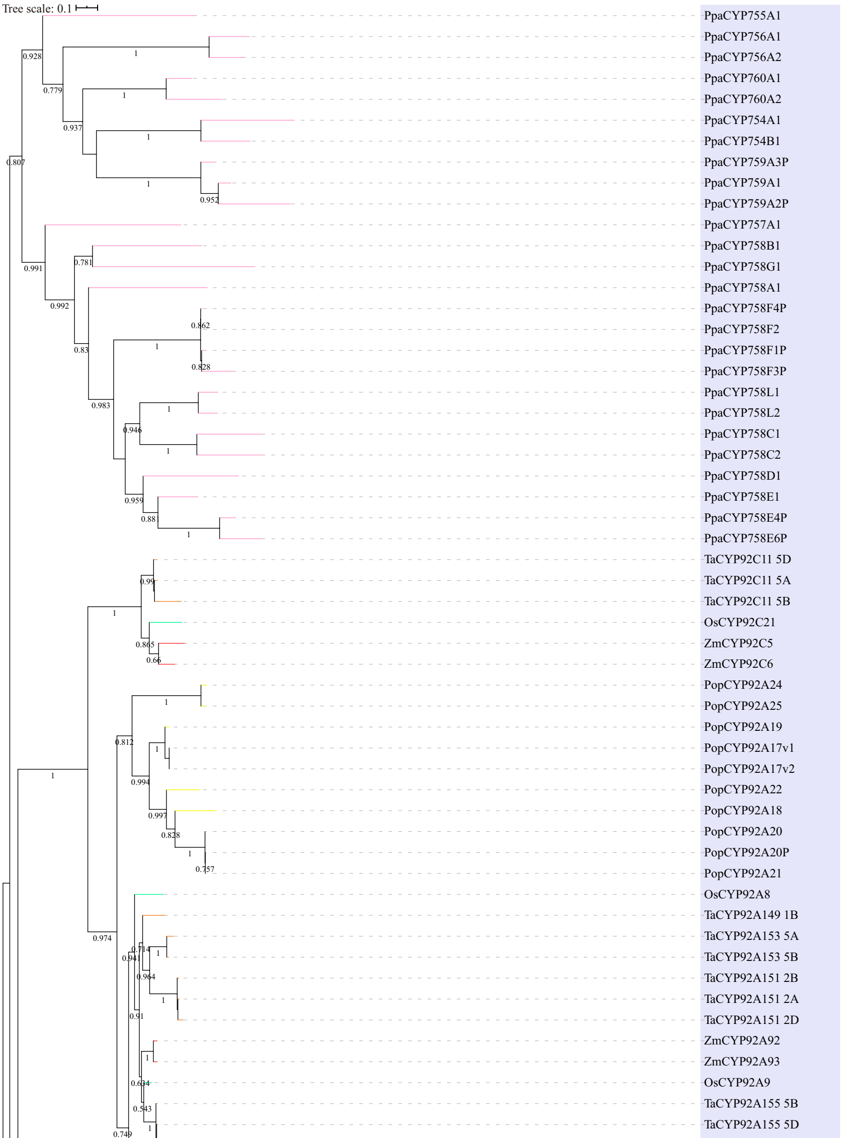
Tree scale: 0.1
PpaCYP755A1
PpaCYP756A1
PpaCYP756A2
PpaCYP760A1
PpaCYP760A2
PpaCYP754A1
PpaCYP754B1
PpaCYP759A3P
PpaCYP759A1
PpaCYP759A2P
PpaCYP757A1
PpaCYP758B1
PpaCYP758G1
PpaCYP758A1
PpaCYP758F4P
PpaCYP758F2
PpaCYP758F1P
PpaCYP758F3P
PpaCYP758L1
PpaCYP758L2
PpaCYP758C1
PpaCYP758C2
PpaCYP758D1
PpaCYP758E1
PpaCYP758E4P
PpaCYP758E6P
TaCYP92C11 5D
TaCYP92C11 5A
TaCYP92C11 5B
OsCYP92C21
ZmCYP92C5
ZmCYP92C6
PopCYP92A24
PopCYP92A25
PopCYP92A19
PopCYP92A17v1
PopCYP92A17v2
PopCYP92A22
PopCYP92A18
PopCYP92A20
PopCYP92A20P
PopCYP92A21
OsCYP92A8
TaCYP92A149 1B
TaCYP92A153 5A
TaCYP92A153 5B
TaCYP92A151 2B
TaCYP92A151 2A
TaCYP92A151 2D
ZmCYP92A92
ZmCYP92A93
OsCYP92A9
TaCYP92A155 5B
TaCYP92A155 5D
0.928
1
0.779
1
0.937
1
0.807
1
0.952
0.781
0.991
0.992
0.862
1
0.83
0.828
0.983
1
0.946
1
0.959
0.881
1
0.99
1
0.865
0.66
1
0.812
1
1
0.994
0.997
0.828
1
0.757
0.974
0.714
0.941
1
0.964
1
0.91
1
0.634
0.543
0.749
1

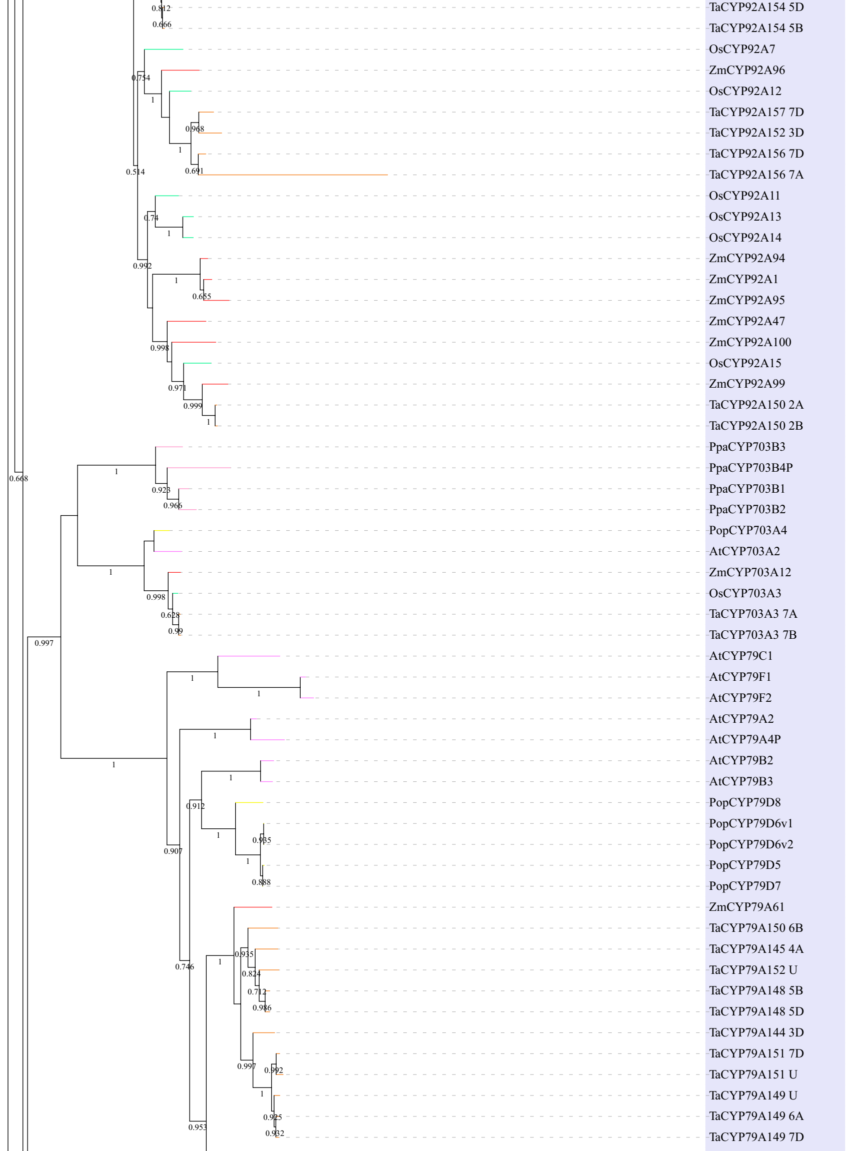

TaCYP92A154 5D
TaCYP92A154 5B
OsCYP92A7
ZmCYP92A96
OsCYP92A12
TaCYP92A157 7D
TaCYP92A152 3D
TaCYP92A156 7D
TaCYP92A156 7A
OsCYP92A11
OsCYP92A13
OsCYP92A14
ZmCYP92A94
ZmCYP92A1
ZmCYP92A95
ZmCYP92A47
ZmCYP92A100
OsCYP92A15
ZmCYP92A99
TaCYP92A150 2A
TaCYP92A150 2B
PpaCYP703B3
PpaCYP703B4P
PpaCYP703B1
PpaCYP703B2
PopCYP703A4
AtCYP703A2
ZmCYP703A12
OsCYP703A3
TaCYP703A3 7A
TaCYP703A3 7B
AtCYP79C1
AtCYP79F1
AtCYP79F2
AtCYP79A2
AtCYP79A4P
AtCYP79B2
AtCYP79B3
PopCYP79D8
PopCYP79D6v1
PopCYP79D6v2
PopCYP79D5
PopCYP79D7
ZmCYP79A61
TaCYP79A150 6B
TaCYP79A145 4A
TaCYP79A152 U
TaCYP79A148 5B
TaCYP79A148 5D
TaCYP79A144 3D
TaCYP79A151 7D
TaCYP79A151 U
TaCYP79A149 U
TaCYP79A149 6A
TaCYP79A149 7D

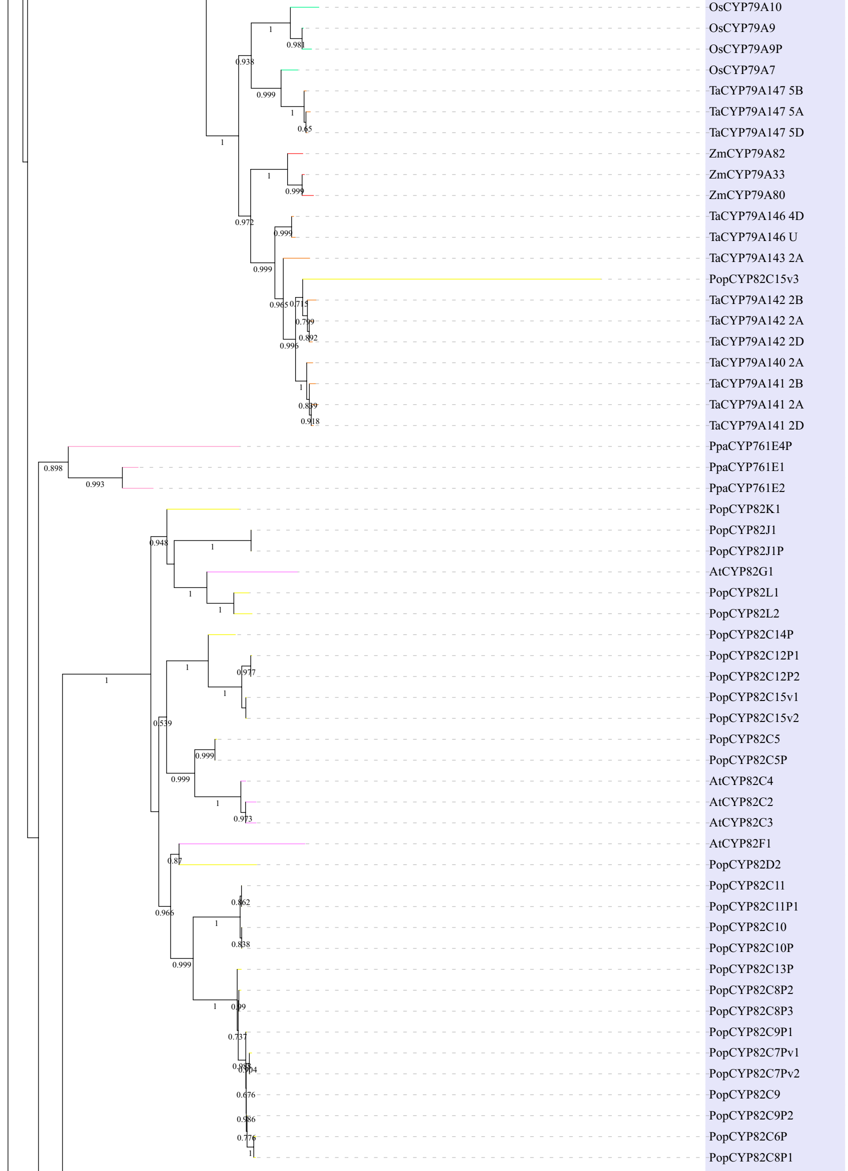

OsCYP79A10
OsCYP79A9
OsCYP79A9P
OsCYP79A7
TaCYP79A147 5B
TaCYP79A147 5A
TaCYP79A147 5D
ZmCYP79A82
ZmCYP79A33
ZmCYP79A80
TaCYP79A146 4D
TaCYP79A146 U
TaCYP79A143 2A
PopCYP82C15v3
TaCYP79A142 2B
TaCYP79A142 2A
TaCYP79A142 2D
TaCYP79A140 2A
TaCYP79A141 2B
TaCYP79A141 2A
TaCYP79A141 2D
PpaCYP761E4P
PpaCYP761E1
PpaCYP761E2
PopCYP82K1
PopCYP82J1
PopCYP82J1P
AtCYP82G1
PopCYP82L1
PopCYP82L2
PopCYP82C14P
PopCYP82C12P1
PopCYP82C12P2
PopCYP82C15v1
PopCYP82C15v2
PopCYP82C5
PopCYP82C5P
AtCYP82C4
AtCYP82C2
AtCYP82C3
AtCYP82F1
PopCYP82D2
PopCYP82C11
PopCYP82C11P1
PopCYP82C10
PopCYP82C10P
PopCYP82C13P
PopCYP82C8P2
PopCYP82C8P3
PopCYP82C9P1
PopCYP82C7Pv1
PopCYP82C7Pv2
PopCYP82C9
PopCYP82C9P2
PopCYP82C6P
PopCYP82C8P1

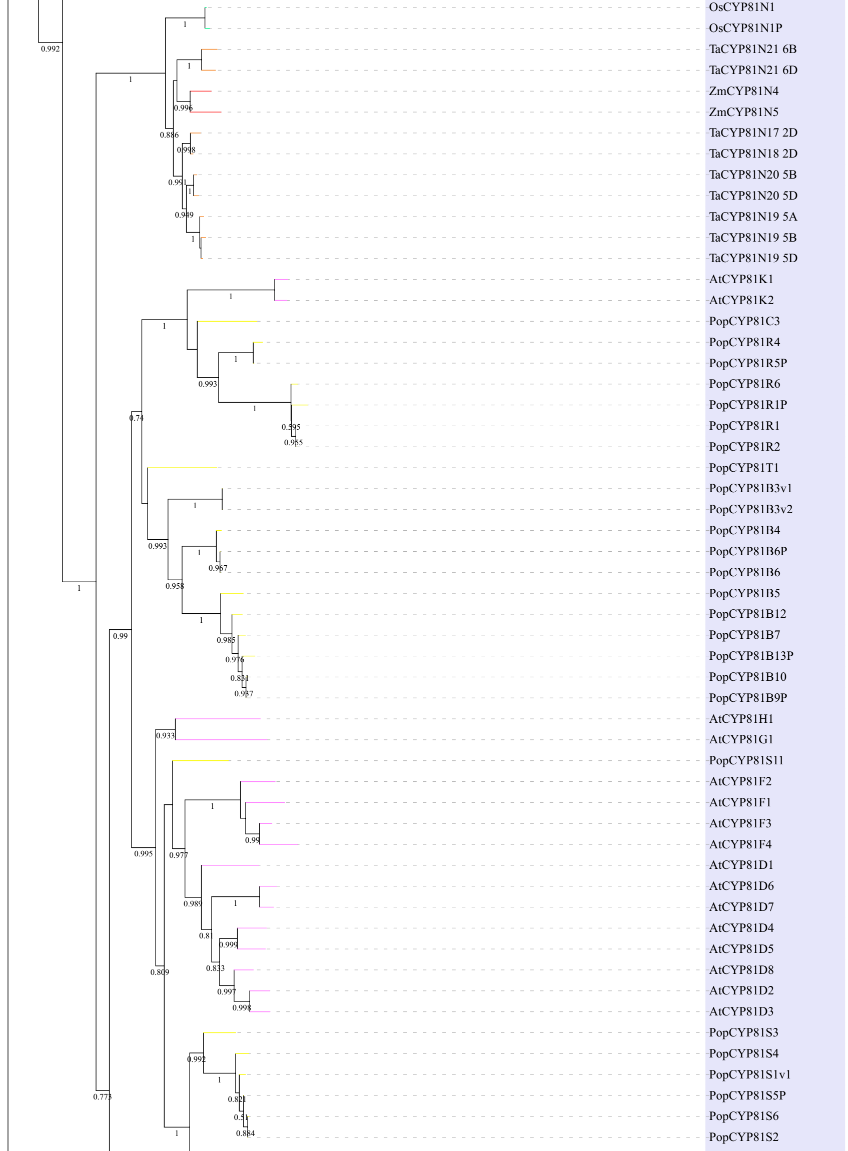
OsCYP81N1
OsCYP81N1P
TaCYP81N21 6B
TaCYP81N21 6D
ZmCYP81N4
ZmCYP81N5
TaCYP81N17 2D
TaCYP81N18 2D
TaCYP81N20 5B
TaCYP81N20 5D
TaCYP81N19 5A
TaCYP81N19 5B
TaCYP81N19 5D
AtCYP81K1
AtCYP81K2
PopCYP81C3
PopCYP81R4
PopCYP81R5P
PopCYP81R6
PopCYP81R1P
PopCYP81R1
PopCYP81R2
PopCYP81T1
PopCYP81B3v1
PopCYP81B3v2
PopCYP81B4
PopCYP81B6P
PopCYP81B6
PopCYP81B5
PopCYP81B12
PopCYP81B7
PopCYP81B13P
PopCYP81B10
PopCYP81B9P
AtCYP81H1
AtCYP81G1
PopCYP81S11
AtCYP81F2
AtCYP81F1
AtCYP81F3
AtCYP81F4
AtCYP81D1
AtCYP81D6
AtCYP81D7
AtCYP81D4
AtCYP81D5
AtCYP81D8
AtCYP81D2
AtCYP81D3
PopCYP81S3
PopCYP81S4
PopCYP81S1v1
PopCYP81S5P
PopCYP81S6
PopCYP81S2
0.992
1
1
1
0.996
0.886
0.998
0.991
1
0.949
1
1
1
0.993
1
1
0.74
0.595
0.955
1
0.993
1
0.967
0.958
1
1
0.985
0.976
0.831
0.937
0.99
0.933
1
0.99
0.995
0.977
0.989
1
0.81
0.999
0.833
0.809
0.997
0.998
0.992
1
0.821
0.773
0.51
0.884
1

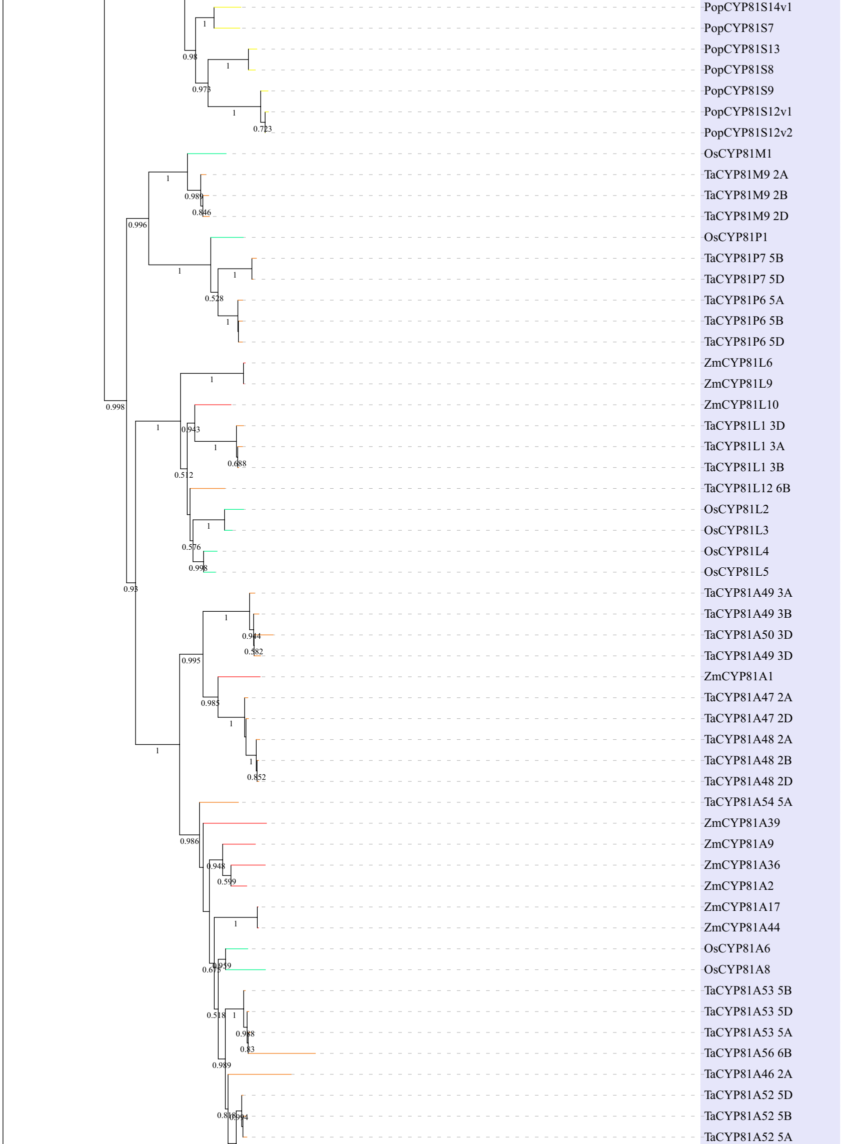
PopCYP81S14v1
PopCYP81S7
PopCYP81S13
PopCYP81S8
PopCYP81S9
PopCYP81S12v1
PopCYP81S12v2
OsCYP81M1
TaCYP81M9 2A
TaCYP81M9 2B
TaCYP81M9 2D
OsCYP81P1
TaCYP81P7 5B
TaCYP81P7 5D
TaCYP81P6 5A
TaCYP81P6 5B
TaCYP81P6 5D
ZmCYP81L6
ZmCYP81L9
ZmCYP81L10
TaCYP81L1 3D
TaCYP81L1 3A
TaCYP81L1 3B
TaCYP81L12 6B
OsCYP81L2
OsCYP81L3
OsCYP81L4
OsCYP81L5
TaCYP81A49 3A
TaCYP81A49 3B
TaCYP81A50 3D
TaCYP81A49 3D
ZmCYP81A1
TaCYP81A47 2A
TaCYP81A47 2D
TaCYP81A48 2A
TaCYP81A48 2B
TaCYP81A48 2D
TaCYP81A54 5A
ZmCYP81A39
ZmCYP81A9
ZmCYP81A36
ZmCYP81A2
ZmCYP81A17
ZmCYP81A44
OsCYP81A6
OsCYP81A8
TaCYP81A53 5B
TaCYP81A53 5D
TaCYP81A53 5A
TaCYP81A56 6B
TaCYP81A46 2A
TaCYP81A52 5D
TaCYP81A52 5B
TaCYP81A52 5A

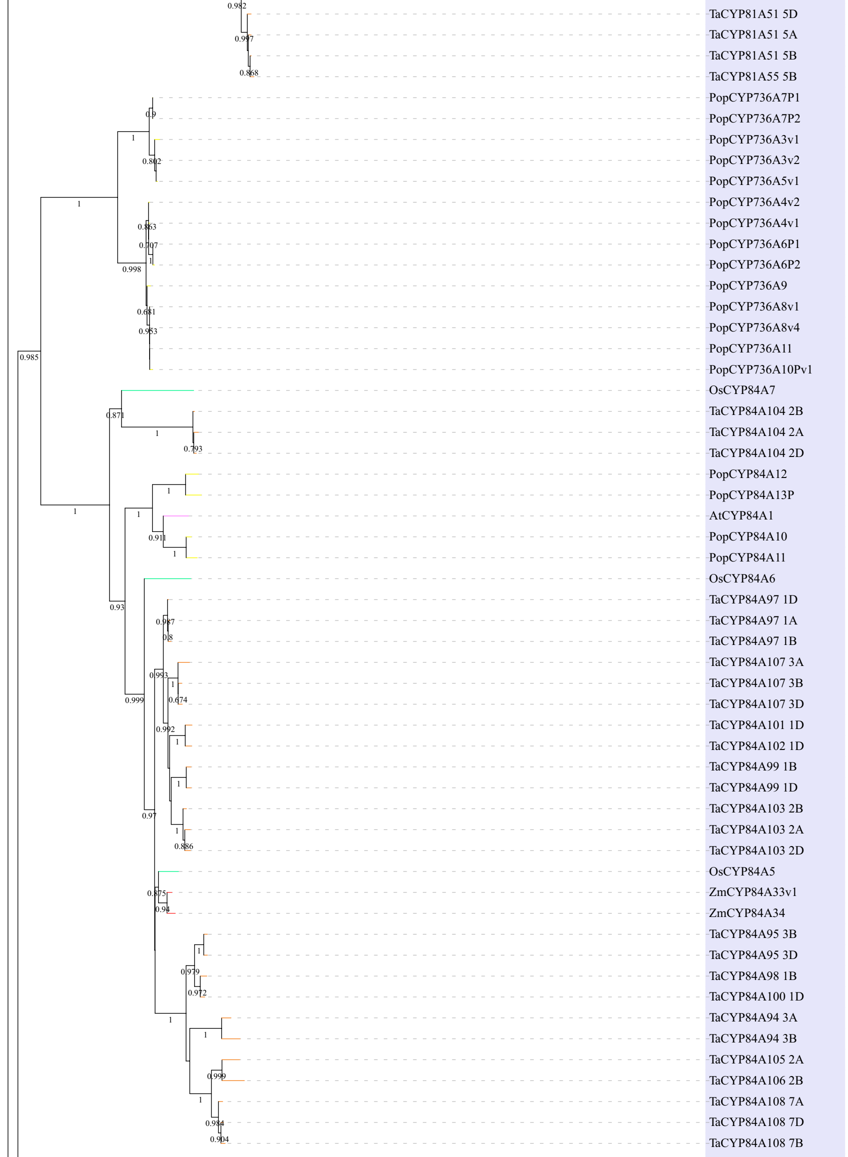
TaCYP81A51 5D
TaCYP81A51 5A
TaCYP81A51 5B
TaCYP81A55 5B
PopCYP736A7P1
PopCYP736A7P2
PopCYP736A3v1
PopCYP736A3v2
PopCYP736A5v1
PopCYP736A4v2
PopCYP736A4v1
PopCYP736A6P1
PopCYP736A6P2
PopCYP736A9
PopCYP736A8v1
PopCYP736A8v4
PopCYP736A11
PopCYP736A10Pv1
OsCYP84A7
TaCYP84A104 2B
TaCYP84A104 2A
TaCYP84A104 2D
PopCYP84A12
PopCYP84A13P
AtCYP84A1
PopCYP84A10
PopCYP84A11
OsCYP84A6
TaCYP84A97 1D
TaCYP84A97 1A
TaCYP84A97 1B
TaCYP84A107 3A
TaCYP84A107 3B
TaCYP84A107 3D
TaCYP84A101 1D
TaCYP84A102 1D
TaCYP84A99 1B
TaCYP84A99 1D
TaCYP84A103 2B
TaCYP84A103 2A
TaCYP84A103 2D
OsCYP84A5
ZmCYP84A33v1
ZmCYP84A34
TaCYP84A95 3B
TaCYP84A95 3D
TaCYP84A98 1B
TaCYP84A100 1D
TaCYP84A94 3A
TaCYP84A94 3B
TaCYP84A105 2A
TaCYP84A106 2B
TaCYP84A108 7A
TaCYP84A108 7D
TaCYP84A108 7B

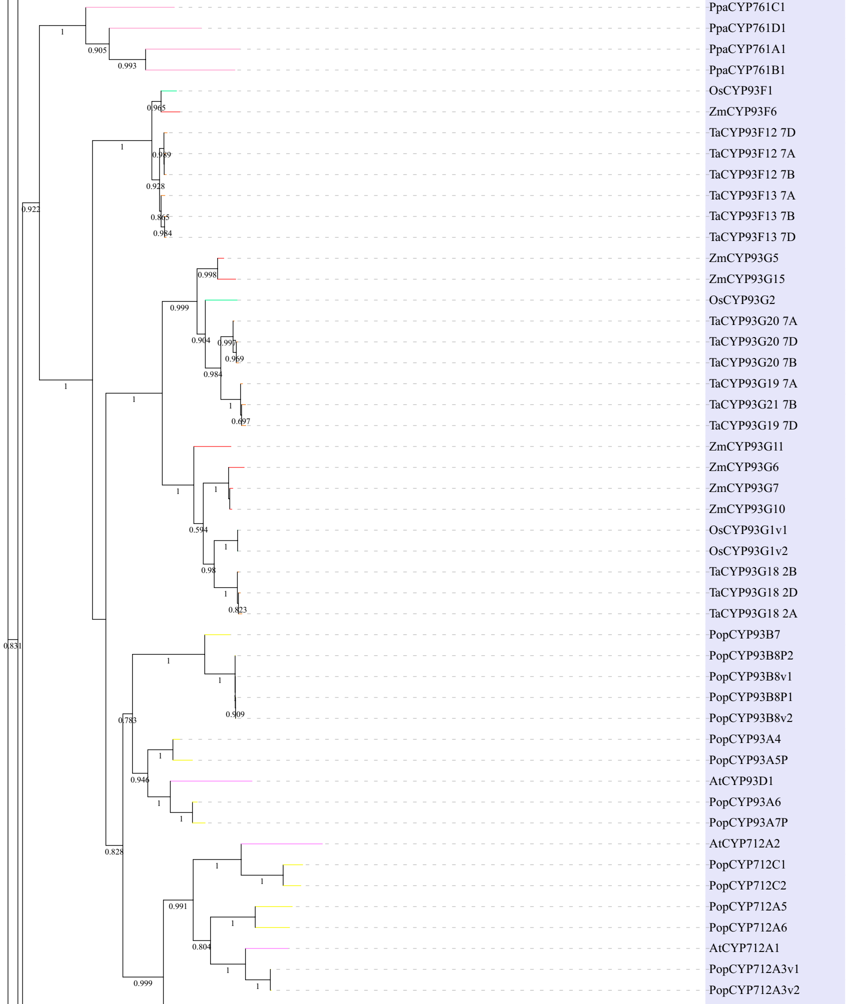
PpaCYP761C1
PpaCYP761D1
PpaCYP761A1
PpaCYP761B1
OsCYP93F1
ZmCYP93F6
TaCYP93F12 7D
TaCYP93F12 7A
TaCYP93F12 7B
TaCYP93F13 7A
TaCYP93F13 7B
TaCYP93F13 7D
ZmCYP93G5
ZmCYP93G15
OsCYP93G2
TaCYP93G20 7A
TaCYP93G20 7D
TaCYP93G20 7B
TaCYP93G19 7A
TaCYP93G21 7B
TaCYP93G19 7D
ZmCYP93G11
ZmCYP93G6
ZmCYP93G7
ZmCYP93G10
OsCYP93G1v1
OsCYP93G1v2
TaCYP93G18 2B
TaCYP93G18 2D
TaCYP93G18 2A
PopCYP93B7
PopCYP93B8P2
PopCYP93B8v1
PopCYP93B8P1
PopCYP93B8v2
PopCYP93A4
PopCYP93A5P
AtCYP93D1
PopCYP93A6
PopCYP93A7P
AtCYP712A2
PopCYP712C1
PopCYP712C2
PopCYP712A5
PopCYP712A6
AtCYP712A1
PopCYP712A3v1
PopCYP712A3v2
1
0.905
0.993
0.965
1
0.989
0.928
0.922
0.865
0.984
0.998
0.999
0.904
0.997
0.969
0.984
1
1
1
0.697
1
1
0.594
1
0.98
1
0.823
0.831
1
1
1
0.909
0.783
1
0.946
1
1
0.828
1
1
0.991
1
0.804
1
0.999
1

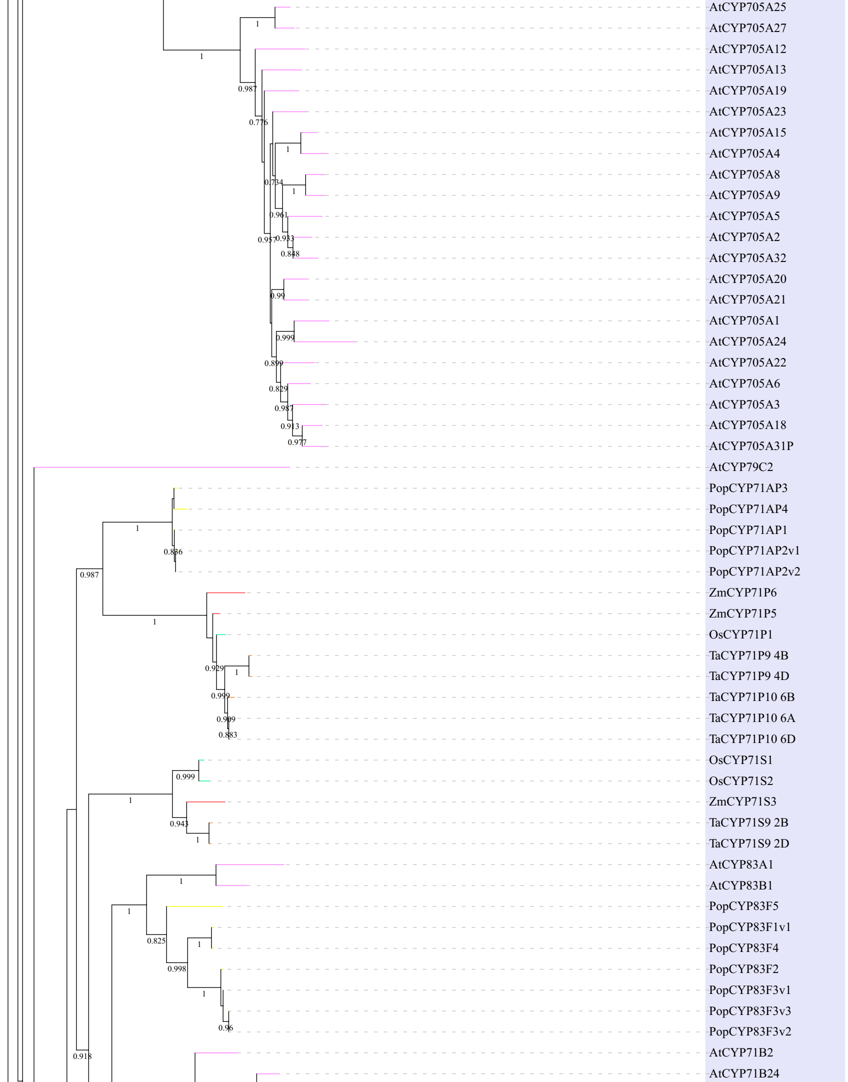
AtCYP705A25
AtCYP705A27
AtCYP705A12
AtCYP705A13
AtCYP705A19
AtCYP705A23
AtCYP705A15
AtCYP705A4
AtCYP705A8
AtCYP705A9
AtCYP705A5
AtCYP705A2
AtCYP705A32
AtCYP705A20
AtCYP705A21
AtCYP705A1
AtCYP705A24
AtCYP705A22
AtCYP705A6
AtCYP705A3
AtCYP705A18
AtCYP705A31P
AtCYP79C2
PopCYP71AP3
PopCYP71AP4
PopCYP71AP1
PopCYP71AP2v1
PopCYP71AP2v2
ZmCYP71P6
ZmCYP71P5
OsCYP71P1
TaCYP71P9 4B
TaCYP71P9 4D
TaCYP71P10 6B
TaCYP71P10 6A
TaCYP71P10 6D
OsCYP71S1
OsCYP71S2
ZmCYP71S3
TaCYP71S9 2B
TaCYP71S9 2D
AtCYP83A1
AtCYP83B1
PopCYP83F5
PopCYP83F1v1
PopCYP83F4
PopCYP83F2
PopCYP83F3v1
PopCYP83F3v3
PopCYP83F3v2
AtCYP71B2
AtCYP71B24
1
1
0.987
0.776
1
0.734
1
0.961
0.957
0.933
0.848
0.99
0.999
0.899
0.829
0.987
0.913
0.977
1
0.836
0.987
1
0.929
1
0.999
0.909
0.883
0.999
1
0.943
1
1
1
0.825
1
0.998
1
0.96
0.918

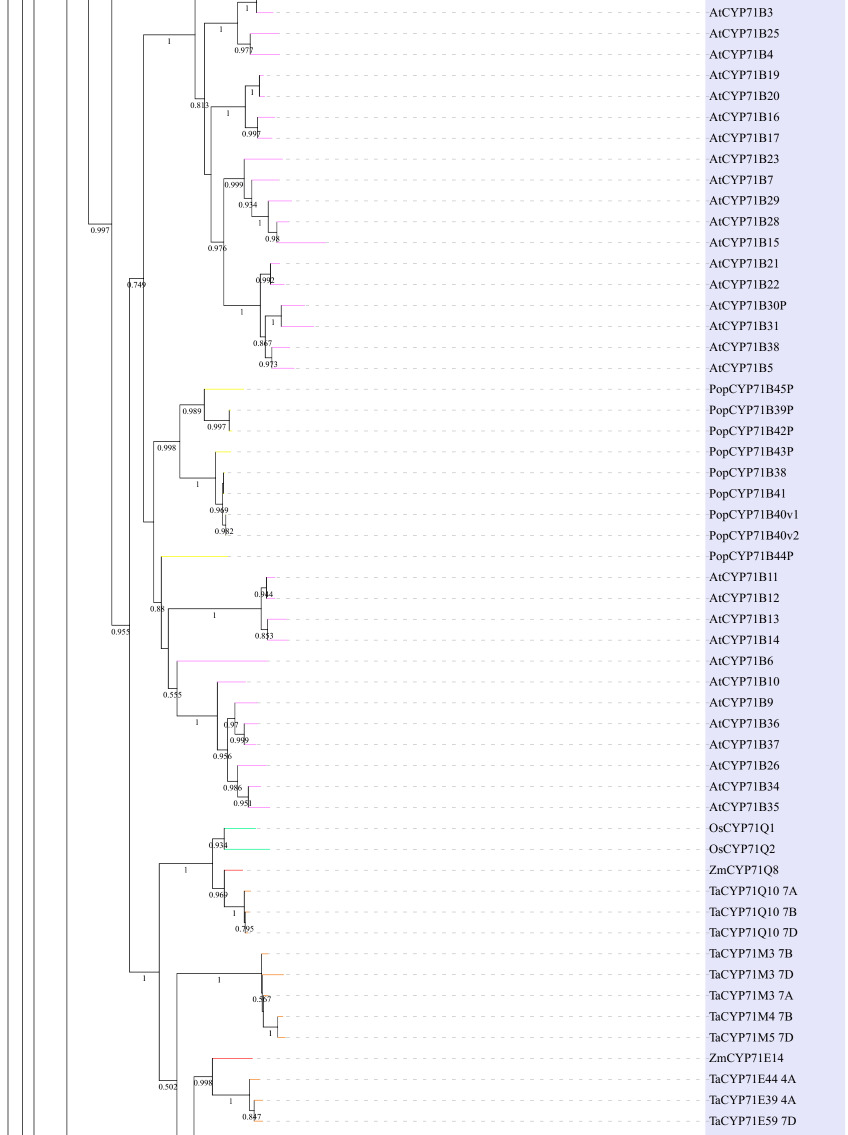
AtCYP71B3
AtCYP71B25
AtCYP71B4
AtCYP71B19
AtCYP71B20
AtCYP71B16
AtCYP71B17
AtCYP71B23
AtCYP71B7
AtCYP71B29
AtCYP71B28
AtCYP71B15
AtCYP71B21
AtCYP71B22
AtCYP71B30P
AtCYP71B31
AtCYP71B38
AtCYP71B5
PopCYP71B45P
PopCYP71B39P
PopCYP71B42P
PopCYP71B43P
PopCYP71B38
PopCYP71B41
PopCYP71B40v1
PopCYP71B40v2
PopCYP71B44P
AtCYP71B11
AtCYP71B12
AtCYP71B13
AtCYP71B14
AtCYP71B6
AtCYP71B10
AtCYP71B9
AtCYP71B36
AtCYP71B37
AtCYP71B26
AtCYP71B34
AtCYP71B35
OsCYP71Q1
OsCYP71Q2
ZmCYP71Q8
TaCYP71Q10 7A
TaCYP71Q10 7B
TaCYP71Q10 7D
TaCYP71M3 7B
TaCYP71M3 7D
TaCYP71M3 7A
TaCYP71M4 7B
TaCYP71M5 7D
ZmCYP71E14
TaCYP71E44 4A
TaCYP71E39 4A
TaCYP71E59 7D

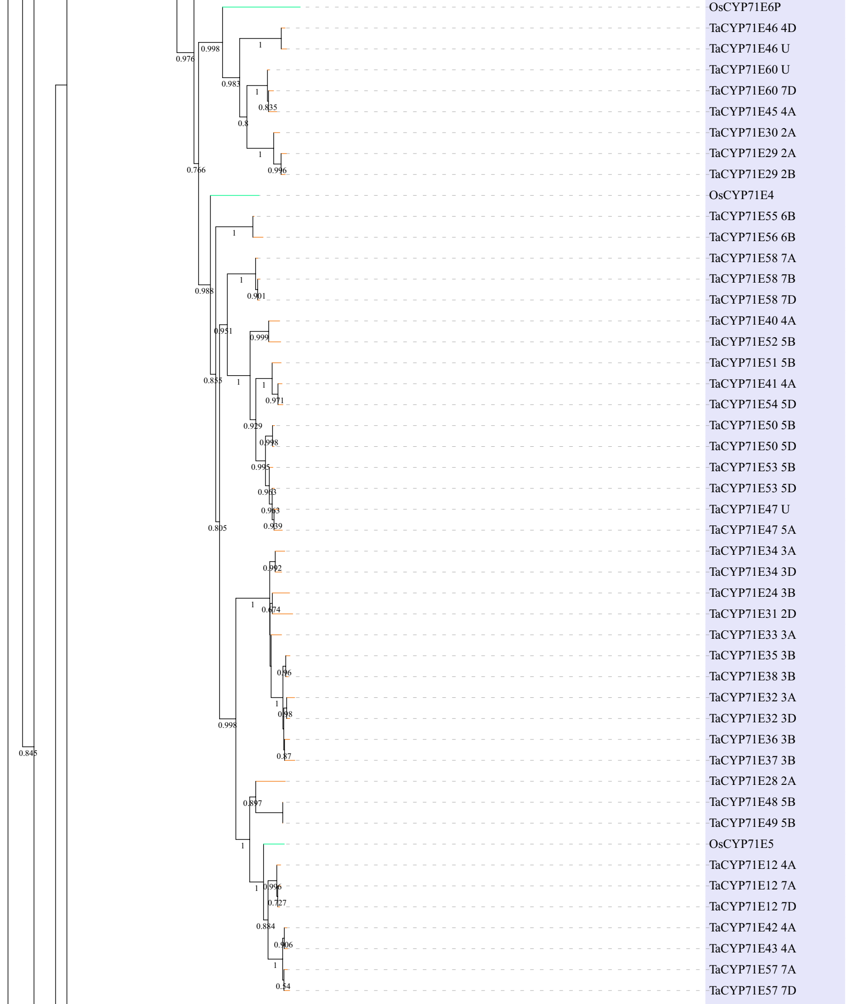
OsCYP71E6P
TaCYP71E46 4D
TaCYP71E46 U
TaCYP71E60 U
TaCYP71E60 7D
TaCYP71E45 4A
TaCYP71E30 2A
TaCYP71E29 2A
TaCYP71E29 2B
OsCYP71E4
TaCYP71E55 6B
TaCYP71E56 6B
TaCYP71E58 7A
TaCYP71E58 7B
TaCYP71E58 7D
TaCYP71E40 4A
TaCYP71E52 5B
TaCYP71E51 5B
TaCYP71E41 4A
TaCYP71E54 5D
TaCYP71E50 5B
TaCYP71E50 5D
TaCYP71E53 5B
TaCYP71E53 5D
TaCYP71E47 U
TaCYP71E47 5A
TaCYP71E34 3A
TaCYP71E34 3D
TaCYP71E24 3B
TaCYP71E31 2D
TaCYP71E33 3A
TaCYP71E35 3B
TaCYP71E38 3B
TaCYP71E32 3A
TaCYP71E32 3D
TaCYP71E36 3B
TaCYP71E37 3B
TaCYP71E28 2A
TaCYP71E48 5B
TaCYP71E49 5B
OsCYP71E5
TaCYP71E12 4A
TaCYP71E12 7A
TaCYP71E12 7D
TaCYP71E42 4A
TaCYP71E43 4A
TaCYP71E57 7A
TaCYP71E57 7D

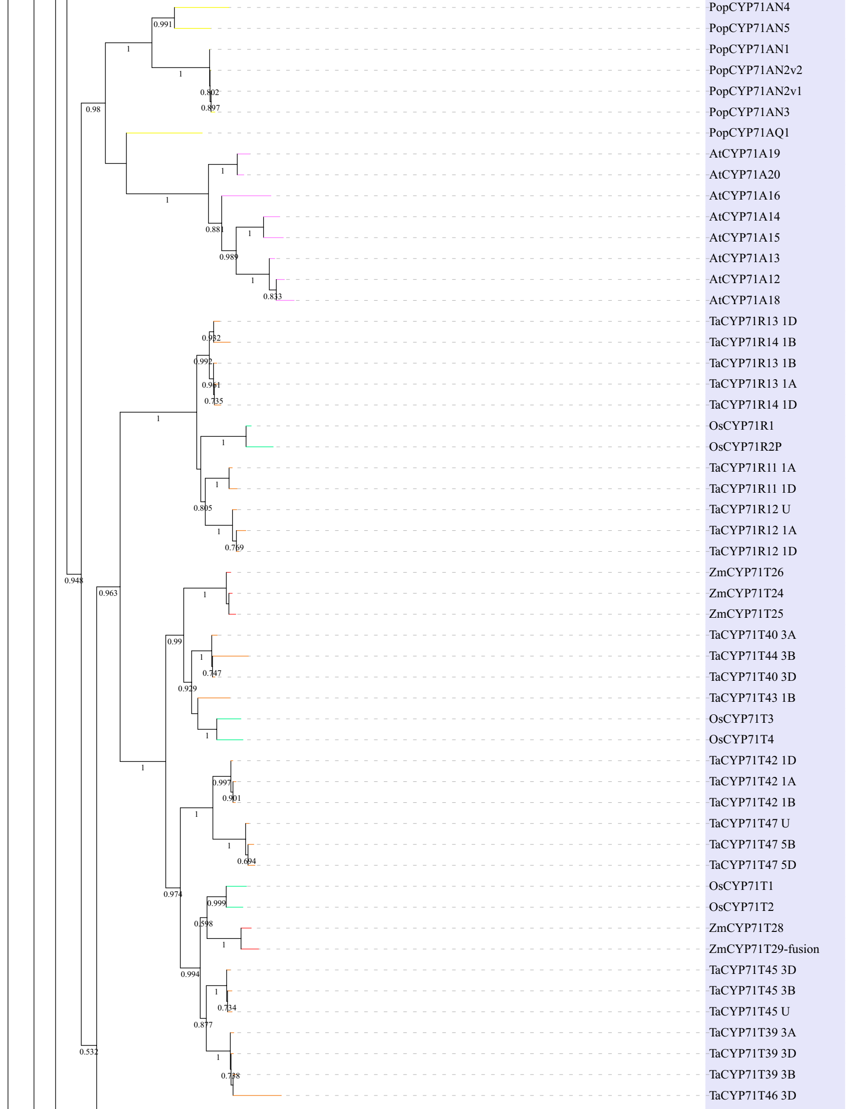
PopCYP71AN4
PopCYP71AN5
PopCYP71AN1
PopCYP71AN2v2
PopCYP71AN2v1
PopCYP71AN3
PopCYP71AQ1
AtCYP71A19
AtCYP71A20
AtCYP71A16
AtCYP71A14
AtCYP71A15
AtCYP71A13
AtCYP71A12
AtCYP71A18
TaCYP71R13 1D
TaCYP71R14 1B
TaCYP71R13 1B
TaCYP71R13 1A
TaCYP71R14 1D
OsCYP71R1
OsCYP71R2P
TaCYP71R11 1A
TaCYP71R11 1D
TaCYP71R12 U
TaCYP71R12 1A
TaCYP71R12 1D
ZmCYP71T26
ZmCYP71T24
ZmCYP71T25
TaCYP71T40 3A
TaCYP71T44 3B
TaCYP71T40 3D
TaCYP71T43 1B
OsCYP71T3
OsCYP71T4
TaCYP71T42 1D
TaCYP71T42 1A
TaCYP71T42 1B
TaCYP71T47 U
TaCYP71T47 5B
TaCYP71T47 5D
OsCYP71T1
OsCYP71T2
ZmCYP71T28
ZmCYP71T29-fusion
TaCYP71T45 3D
TaCYP71T45 3B
TaCYP71T45 U
TaCYP71T39 3A
TaCYP71T39 3D
TaCYP71T39 3B
TaCYP71T46 3D

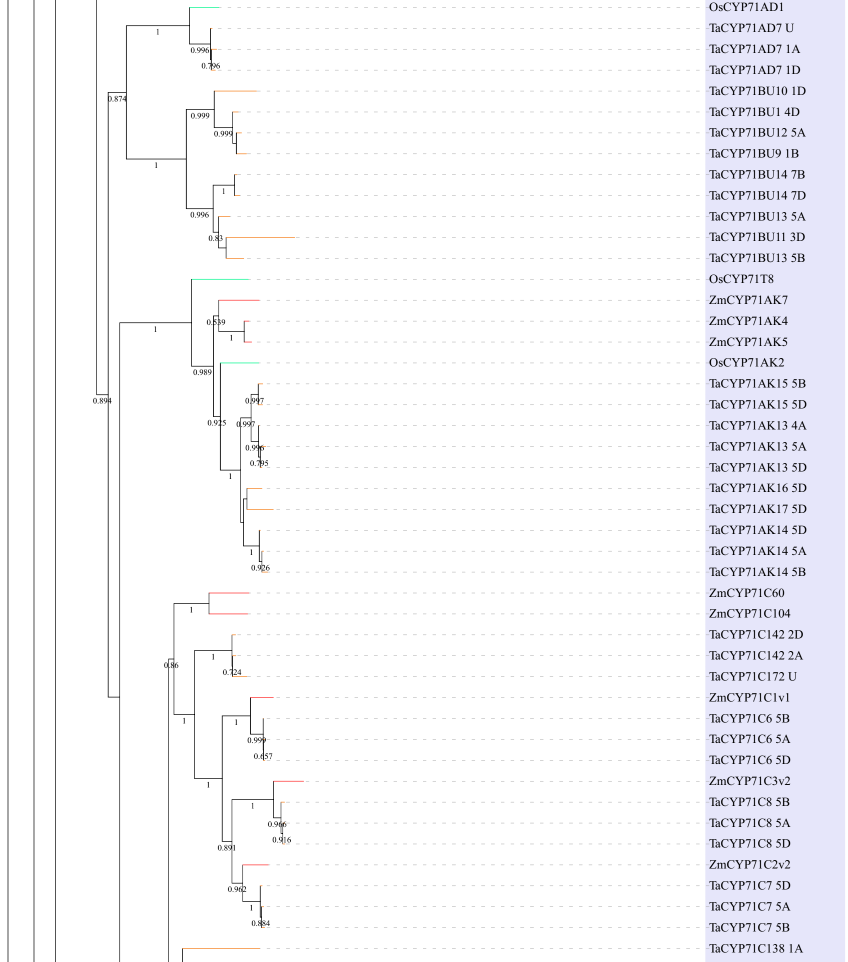
OsCYP71AD1
TaCYP71AD7 U
TaCYP71AD7 1A
TaCYP71AD7 1D
TaCYP71BU10 1D
TaCYP71BU1 4D
TaCYP71BU12 5A
TaCYP71BU9 1B
TaCYP71BU14 7B
TaCYP71BU14 7D
TaCYP71BU13 5A
TaCYP71BU11 3D
TaCYP71BU13 5B
OsCYP71T8
ZmCYP71AK7
ZmCYP71AK4
ZmCYP71AK5
OsCYP71AK2
TaCYP71AK15 5B
TaCYP71AK15 5D
TaCYP71AK13 4A
TaCYP71AK13 5A
TaCYP71AK13 5D
TaCYP71AK16 5D
TaCYP71AK17 5D
TaCYP71AK14 5D
TaCYP71AK14 5A
TaCYP71AK14 5B
ZmCYP71C60
ZmCYP71C104
TaCYP71C142 2D
TaCYP71C142 2A
TaCYP71C172 U
ZmCYP71C1v1
TaCYP71C6 5B
TaCYP71C6 5A
TaCYP71C6 5D
ZmCYP71C3v2
TaCYP71C8 5B
TaCYP71C8 5A
TaCYP71C8 5D
ZmCYP71C2v2
TaCYP71C7 5D
TaCYP71C7 5A
TaCYP71C7 5B
TaCYP71C138 1A

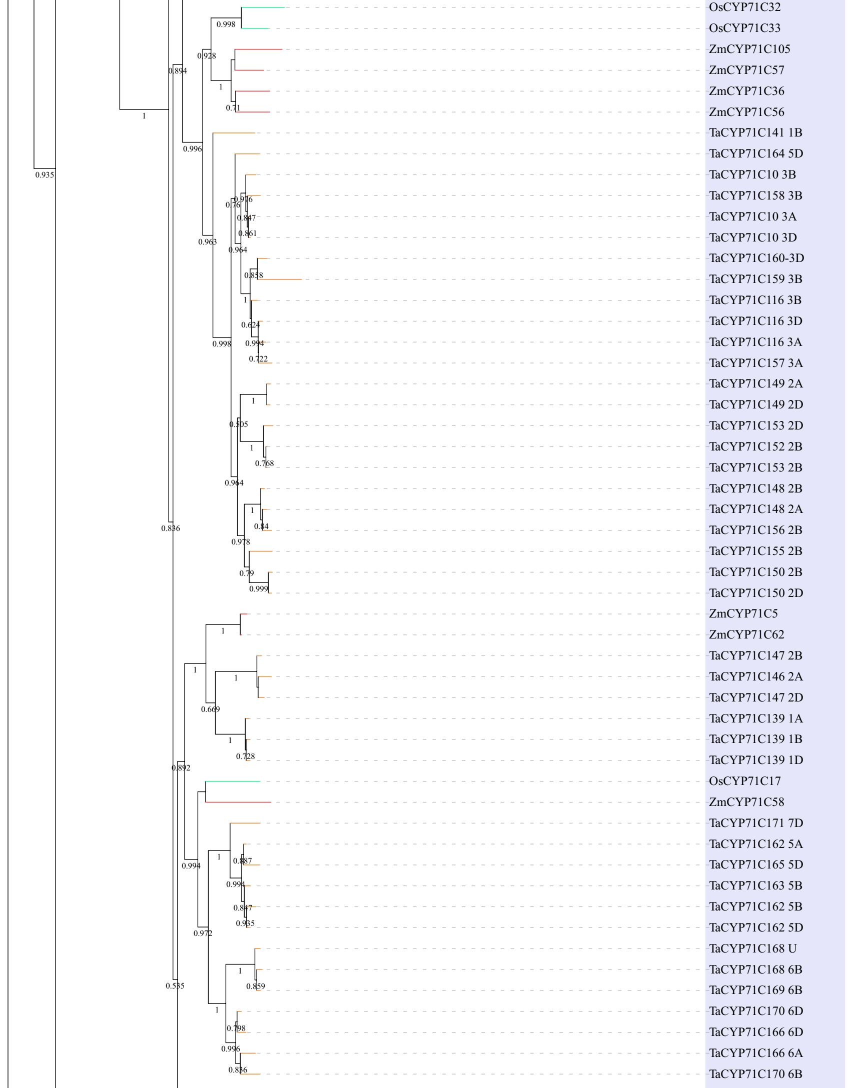
OsCYP71C32
OsCYP71C33
ZmCYP71C105
ZmCYP71C57
ZmCYP71C36
ZmCYP71C56
TaCYP71C141 1B
TaCYP71C164 5D
TaCYP71C10 3B
TaCYP71C158 3B
TaCYP71C10 3A
TaCYP71C10 3D
TaCYP71C160-3D
TaCYP71C159 3B
TaCYP71C116 3B
TaCYP71C116 3D
TaCYP71C116 3A
TaCYP71C157 3A
TaCYP71C149 2A
TaCYP71C149 2D
TaCYP71C153 2D
TaCYP71C152 2B
TaCYP71C153 2B
TaCYP71C148 2B
TaCYP71C148 2A
TaCYP71C156 2B
TaCYP71C155 2B
TaCYP71C150 2B
TaCYP71C150 2D
ZmCYP71C5
ZmCYP71C62
TaCYP71C147 2B
TaCYP71C146 2A
TaCYP71C147 2D
TaCYP71C139 1A
TaCYP71C139 1B
TaCYP71C139 1D
OsCYP71C17
ZmCYP71C58
TaCYP71C171 7D
TaCYP71C162 5A
TaCYP71C165 5D
TaCYP71C163 5B
TaCYP71C162 5B
TaCYP71C162 5D
TaCYP71C168 U
TaCYP71C168 6B
TaCYP71C169 6B
TaCYP71C170 6D
TaCYP71C166 6D
TaCYP71C166 6A
TaCYP71C170 6B

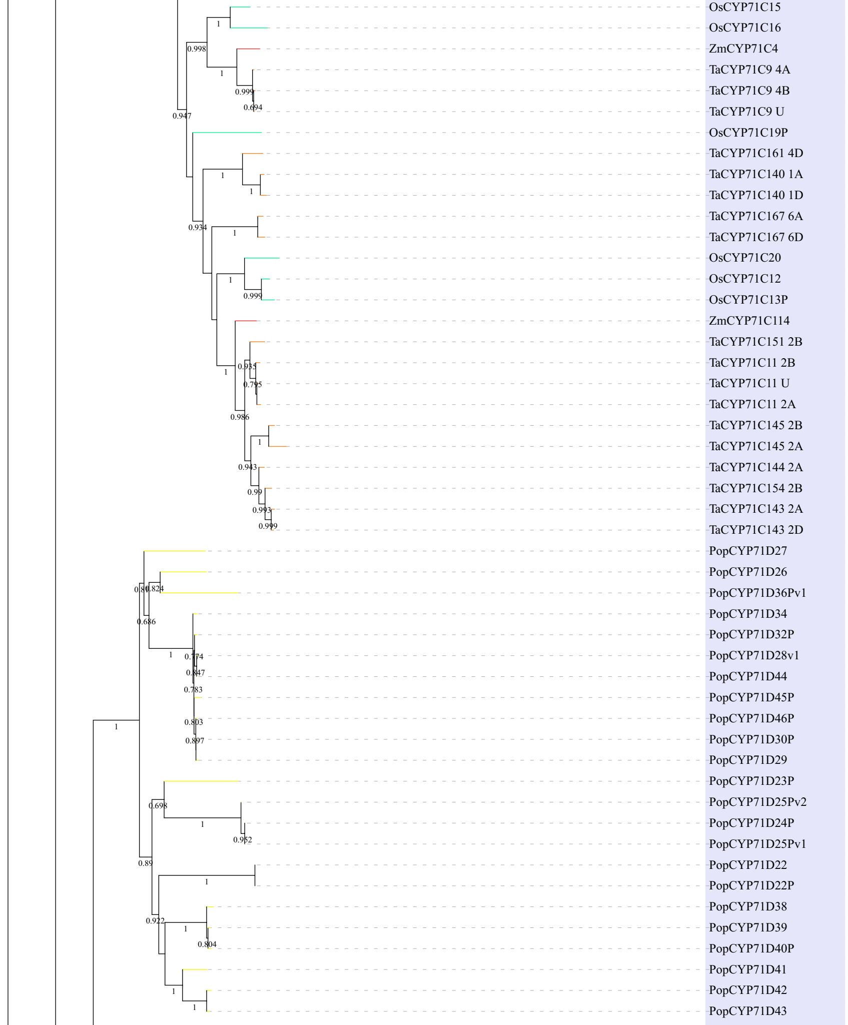
OsCYP71C15
OsCYP71C16
ZmCYP71C4
TaCYP71C9 4A
TaCYP71C9 4B
TaCYP71C9 U
OsCYP71C19P
TaCYP71C161 4D
TaCYP71C140 1A
TaCYP71C140 1D
TaCYP71C167 6A
TaCYP71C167 6D
OsCYP71C20
OsCYP71C12
OsCYP71C13P
ZmCYP71C114
TaCYP71C151 2B
TaCYP71C11 2B
TaCYP71C11 U
TaCYP71C11 2A
TaCYP71C145 2B
TaCYP71C145 2A
TaCYP71C144 2A
TaCYP71C154 2B
TaCYP71C143 2A
TaCYP71C143 2D
PopCYP71D27
PopCYP71D26
PopCYP71D36Pv1
PopCYP71D34
PopCYP71D32P
PopCYP71D28v1
PopCYP71D44
PopCYP71D45P
PopCYP71D46P
PopCYP71D30P
PopCYP71D29
PopCYP71D23P
PopCYP71D25Pv2
PopCYP71D24P
PopCYP71D25Pv1
PopCYP71D22
PopCYP71D22P
PopCYP71D38
PopCYP71D39
PopCYP71D40P
PopCYP71D41
PopCYP71D42
PopCYP71D43

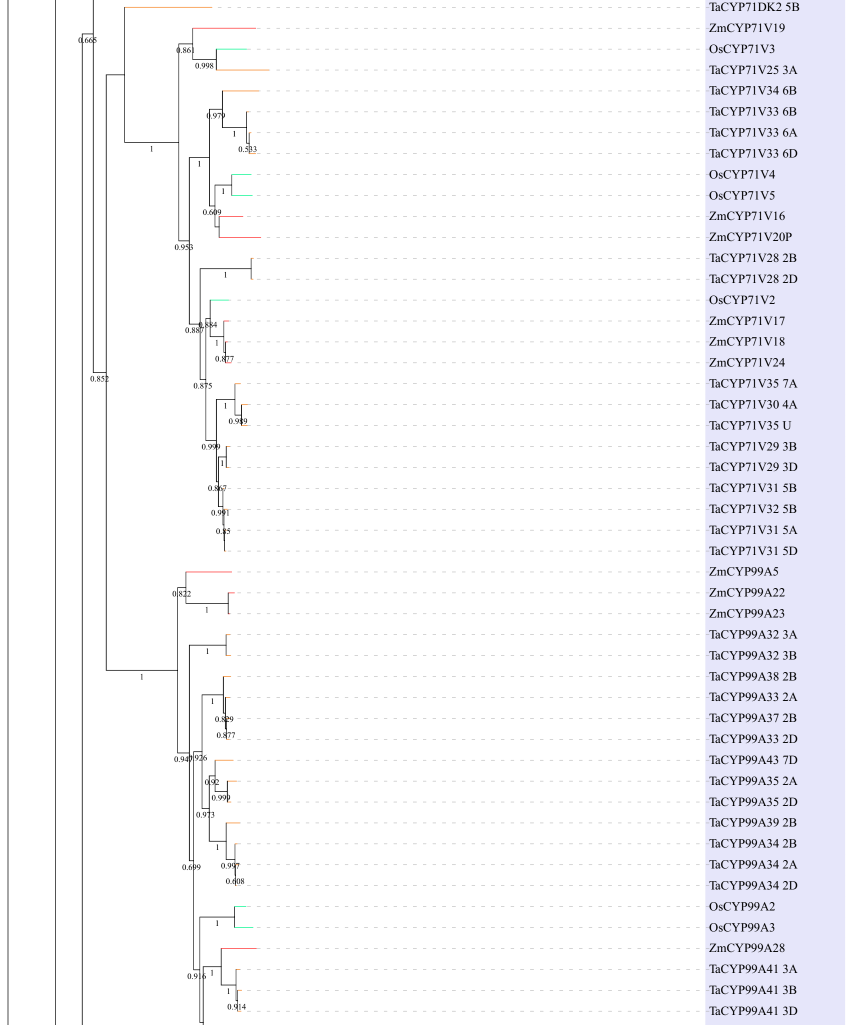
TaCYP71DK2 5B
ZmCYP71V19
OsCYP71V3
TaCYP71V25 3A
TaCYP71V34 6B
TaCYP71V33 6B
TaCYP71V33 6A
TaCYP71V33 6D
OsCYP71V4
OsCYP71V5
ZmCYP71V16
ZmCYP71V20P
TaCYP71V28 2B
TaCYP71V28 2D
OsCYP71V2
ZmCYP71V17
ZmCYP71V18
ZmCYP71V24
TaCYP71V35 7A
TaCYP71V30 4A
TaCYP71V35 U
TaCYP71V29 3B
TaCYP71V29 3D
TaCYP71V31 5B
TaCYP71V32 5B
TaCYP71V31 5A
TaCYP71V31 5D
ZmCYP99A5
ZmCYP99A22
ZmCYP99A23
TaCYP99A32 3A
TaCYP99A32 3B
TaCYP99A38 2B
TaCYP99A33 2A
TaCYP99A37 2B
TaCYP99A33 2D
TaCYP99A43 7D
TaCYP99A35 2A
TaCYP99A35 2D
TaCYP99A39 2B
TaCYP99A34 2B
TaCYP99A34 2A
TaCYP99A34 2D
OsCYP99A2
OsCYP99A3
ZmCYP99A28
TaCYP99A41 3A
TaCYP99A41 3B
TaCYP99A41 3D

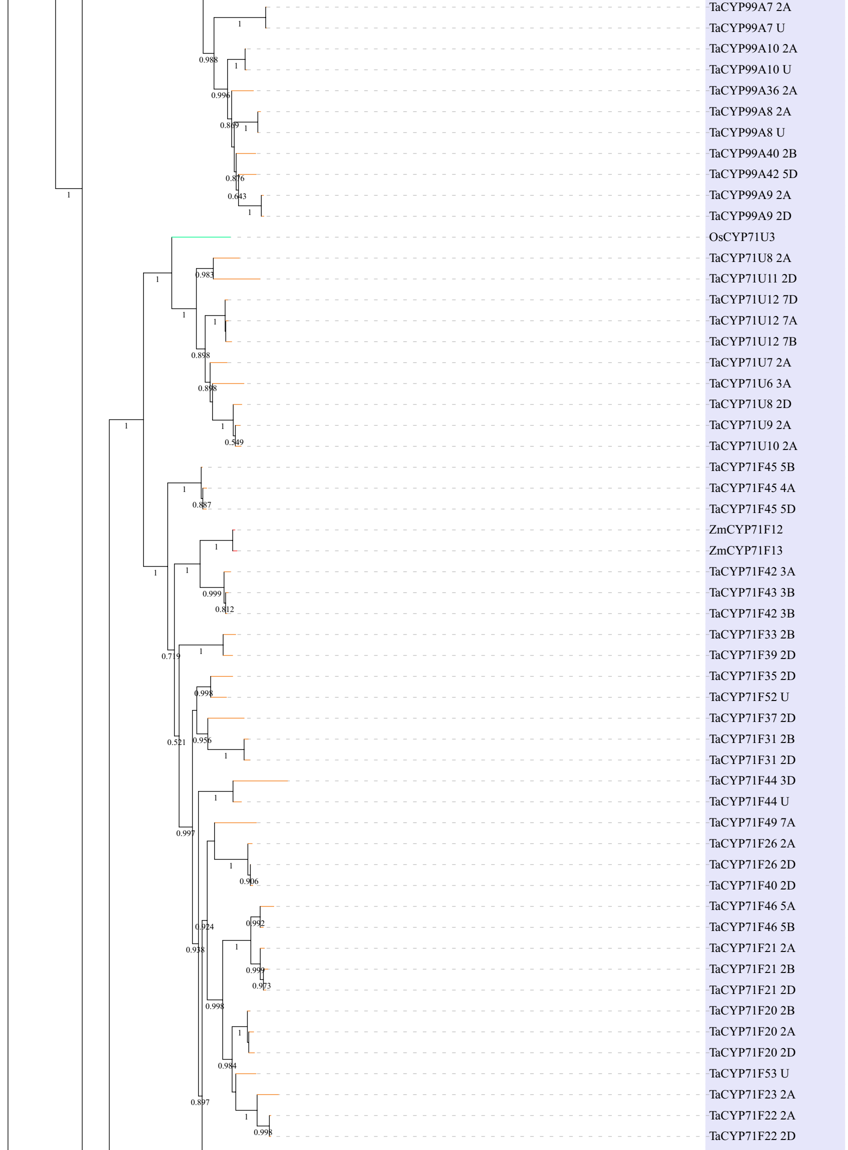
TaCYP99A7 2A
TaCYP99A7 U
TaCYP99A10 2A
TaCYP99A10 U
TaCYP99A36 2A
TaCYP99A8 2A
TaCYP99A8 U
TaCYP99A40 2B
TaCYP99A42 5D
TaCYP99A9 2A
TaCYP99A9 2D
OsCYP71U3
TaCYP71U8 2A
TaCYP71U11 2D
TaCYP71U12 7D
TaCYP71U12 7A
TaCYP71U12 7B
TaCYP71U7 2A
TaCYP71U6 3A
TaCYP71U8 2D
TaCYP71U9 2A
TaCYP71U10 2A
TaCYP71F45 5B
TaCYP71F45 4A
TaCYP71F45 5D
ZmCYP71F12
ZmCYP71F13
TaCYP71F42 3A
TaCYP71F43 3B
TaCYP71F42 3B
TaCYP71F33 2B
TaCYP71F39 2D
TaCYP71F35 2D
TaCYP71F52 U
TaCYP71F37 2D
TaCYP71F31 2B
TaCYP71F31 2D
TaCYP71F44 3D
TaCYP71F44 U
TaCYP71F49 7A
TaCYP71F26 2A
TaCYP71F26 2D
TaCYP71F40 2D
TaCYP71F46 5A
TaCYP71F46 5B
TaCYP71F21 2A
TaCYP71F21 2B
TaCYP71F21 2D
TaCYP71F20 2B
TaCYP71F20 2A
TaCYP71F20 2D
TaCYP71F53 U
TaCYP71F23 2A
TaCYP71F22 2A
TaCYP71F22 2D
1
0.988
1
0.996
0.869
1
0.876
0.643
1
1
1
0.983
1
1
0.898
0.898
1
0.549
1
1
0.887
1
1
0.999
0.812
0.719
1
0.998
0.521
0.956
1
1
0.997
1
0.906
0.992
0.924
1
0.938
0.999
0.973
0.998
1
0.984
0.897
1
0.998

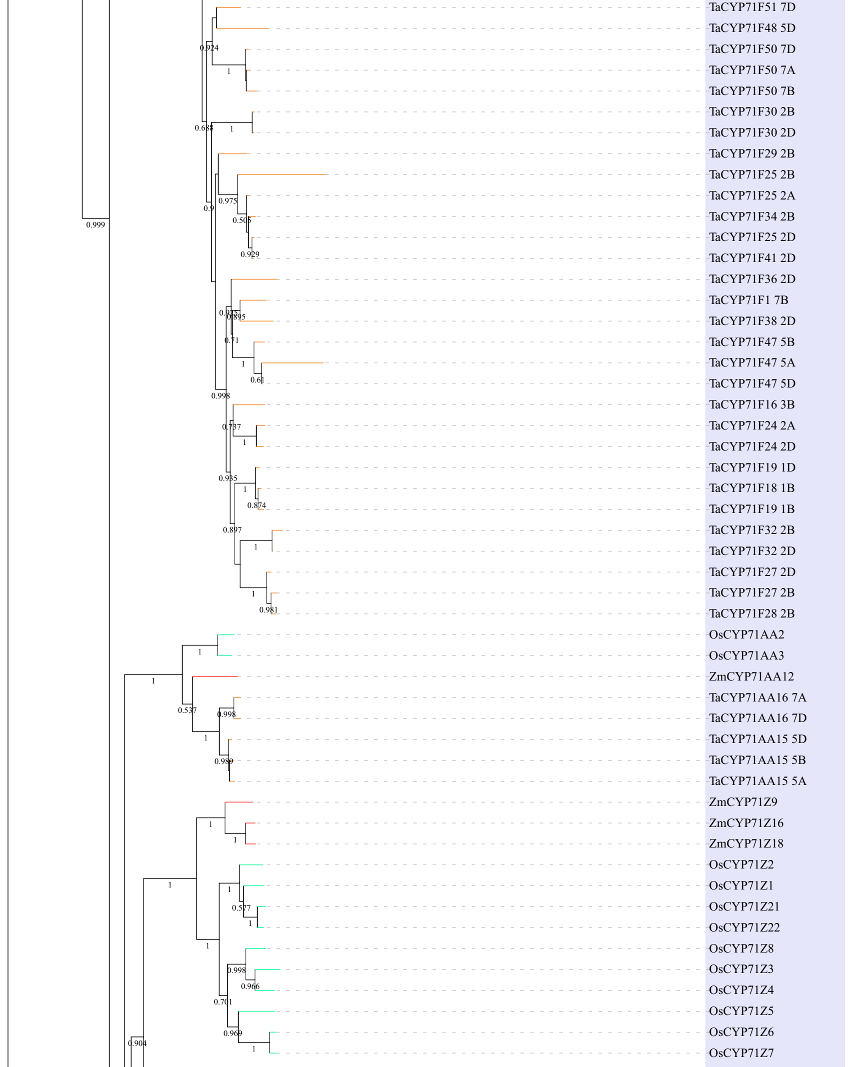

TaCYP71F51 7D
TaCYP71F48 5D
TaCYP71F50 7D
TaCYP71F50 7A
TaCYP71F50 7B
TaCYP71F30 2B
TaCYP71F30 2D
TaCYP71F29 2B
TaCYP71F25 2B
TaCYP71F25 2A
TaCYP71F34 2B
TaCYP71F25 2D
TaCYP71F41 2D
TaCYP71F36 2D
TaCYP71F1 7B
TaCYP71F38 2D
TaCYP71F47 5B
TaCYP71F47 5A
TaCYP71F47 5D
TaCYP71F16 3B
TaCYP71F24 2A
TaCYP71F24 2D
TaCYP71F19 1D
TaCYP71F18 1B
TaCYP71F19 1B
TaCYP71F32 2B
TaCYP71F32 2D
TaCYP71F27 2D
TaCYP71F27 2B
TaCYP71F28 2B
OsCYP71AA2
OsCYP71AA3
ZmCYP71AA12
TaCYP71AA16 7A
TaCYP71AA16 7D
TaCYP71AA15 5D
TaCYP71AA15 5B
TaCYP71AA15 5A
ZmCYP71Z9
ZmCYP71Z16
ZmCYP71Z18
OsCYP71Z2
OsCYP71Z1
OsCYP71Z21
OsCYP71Z22
OsCYP71Z8
OsCYP71Z3
OsCYP71Z4
OsCYP71Z5
OsCYP71Z6
OsCYP71Z7
0.999
0.924
1
0.688
1
0.9
0.975
0.505
0.929
0.995
0.895
0.71
1
0.61
0.998
0.737
1
0.935
1
0.874
0.897
1
1
0.981
1
1
0.537
0.998
1
0.989
1
1
1
1
0.577
1
1
0.998
0.966
0.701
0.969
1
0.904

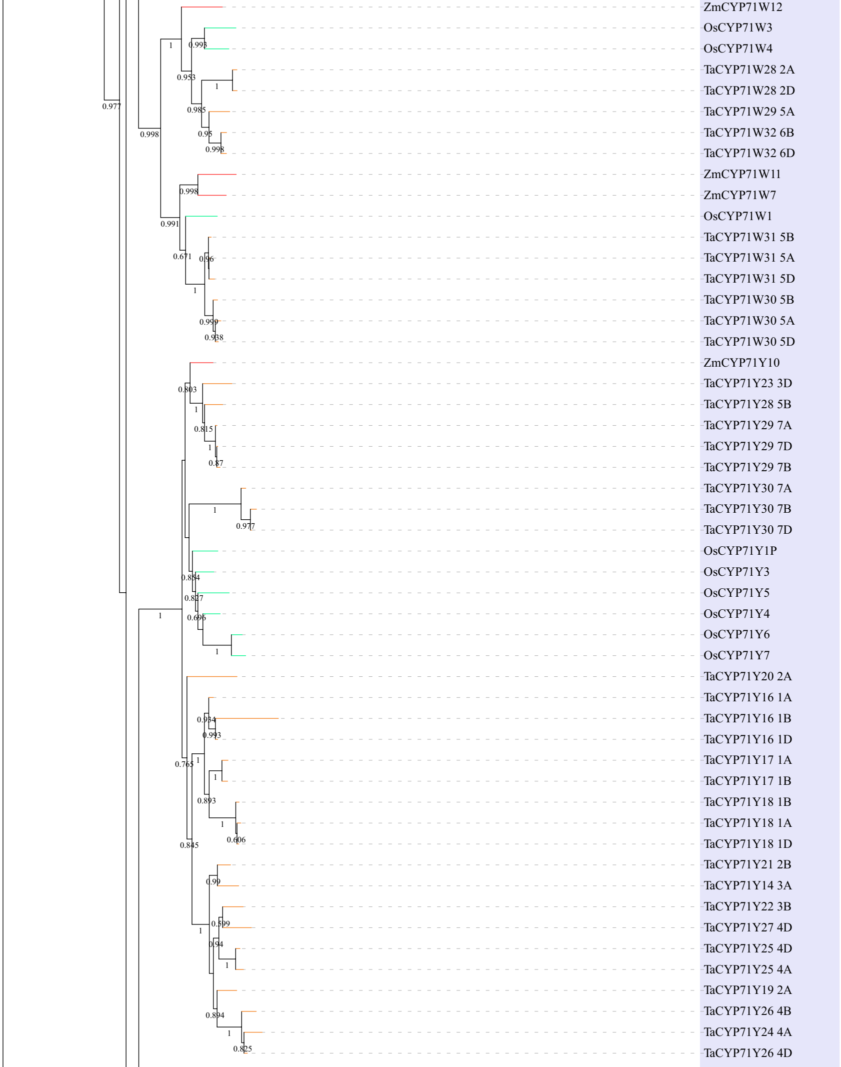

ZmCYP71W12
OsCYP71W3
OsCYP71W4
TaCYP71W28 2A
TaCYP71W28 2D
TaCYP71W29 5A
TaCYP71W32 6B
TaCYP71W32 6D
ZmCYP71W11
ZmCYP71W7
OsCYP71W1
TaCYP71W31 5B
TaCYP71W31 5A
TaCYP71W31 5D
TaCYP71W30 5B
TaCYP71W30 5A
TaCYP71W30 5D
ZmCYP71Y10
TaCYP71Y23 3D
TaCYP71Y28 5B
TaCYP71Y29 7A
TaCYP71Y29 7D
TaCYP71Y29 7B
TaCYP71Y30 7A
TaCYP71Y30 7B
TaCYP71Y30 7D
OsCYP71Y1P
OsCYP71Y3
OsCYP71Y5
OsCYP71Y4
OsCYP71Y6
OsCYP71Y7
TaCYP71Y20 2A
TaCYP71Y16 1A
TaCYP71Y16 1B
TaCYP71Y16 1D
TaCYP71Y17 1A
TaCYP71Y17 1B
TaCYP71Y18 1B
TaCYP71Y18 1A
TaCYP71Y18 1D
TaCYP71Y21 2B
TaCYP71Y14 3A
TaCYP71Y22 3B
TaCYP71Y27 4D
TaCYP71Y25 4D
TaCYP71Y25 4A
TaCYP71Y19 2A
TaCYP71Y26 4B
TaCYP71Y24 4A
TaCYP71Y26 4D

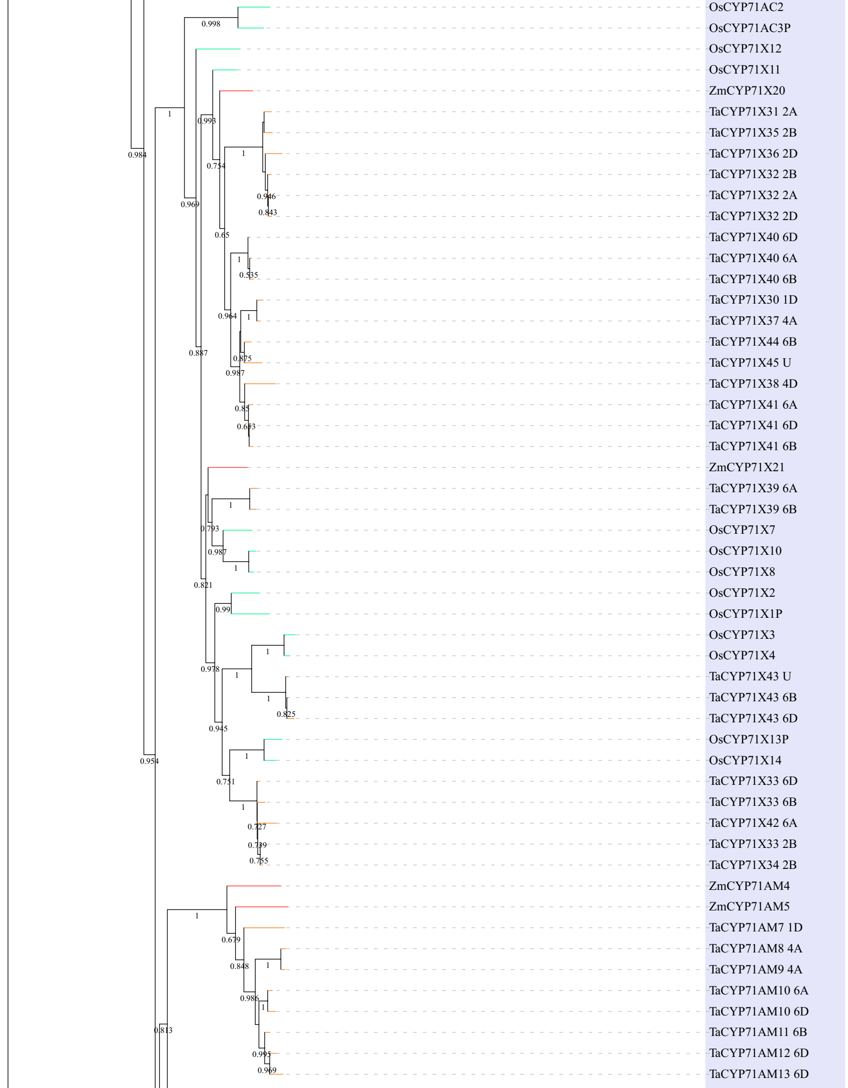
OsCYP71AC2
OsCYP71AC3P
OsCYP71X12
OsCYP71X11
ZmCYP71X20
TaCYP71X31 2A
TaCYP71X35 2B
TaCYP71X36 2D
TaCYP71X32 2B
TaCYP71X32 2A
TaCYP71X32 2D
TaCYP71X40 6D
TaCYP71X40 6A
TaCYP71X40 6B
TaCYP71X30 1D
TaCYP71X37 4A
TaCYP71X44 6B
TaCYP71X45 U
TaCYP71X38 4D
TaCYP71X41 6A
TaCYP71X41 6D
TaCYP71X41 6B
ZmCYP71X21
TaCYP71X39 6A
TaCYP71X39 6B
OsCYP71X7
OsCYP71X10
OsCYP71X8
OsCYP71X2
OsCYP71X1P
OsCYP71X3
OsCYP71X4
TaCYP71X43 U
TaCYP71X43 6B
TaCYP71X43 6D
OsCYP71X13P
OsCYP71X14
TaCYP71X33 6D
TaCYP71X33 6B
TaCYP71X42 6A
TaCYP71X33 2B
TaCYP71X34 2B
ZmCYP71AM4
ZmCYP71AM5
TaCYP71AM7 1D
TaCYP71AM8 4A
TaCYP71AM9 4A
TaCYP71AM10 6A
TaCYP71AM10 6D
TaCYP71AM11 6B
TaCYP71AM12 6D
TaCYP71AM13 6D

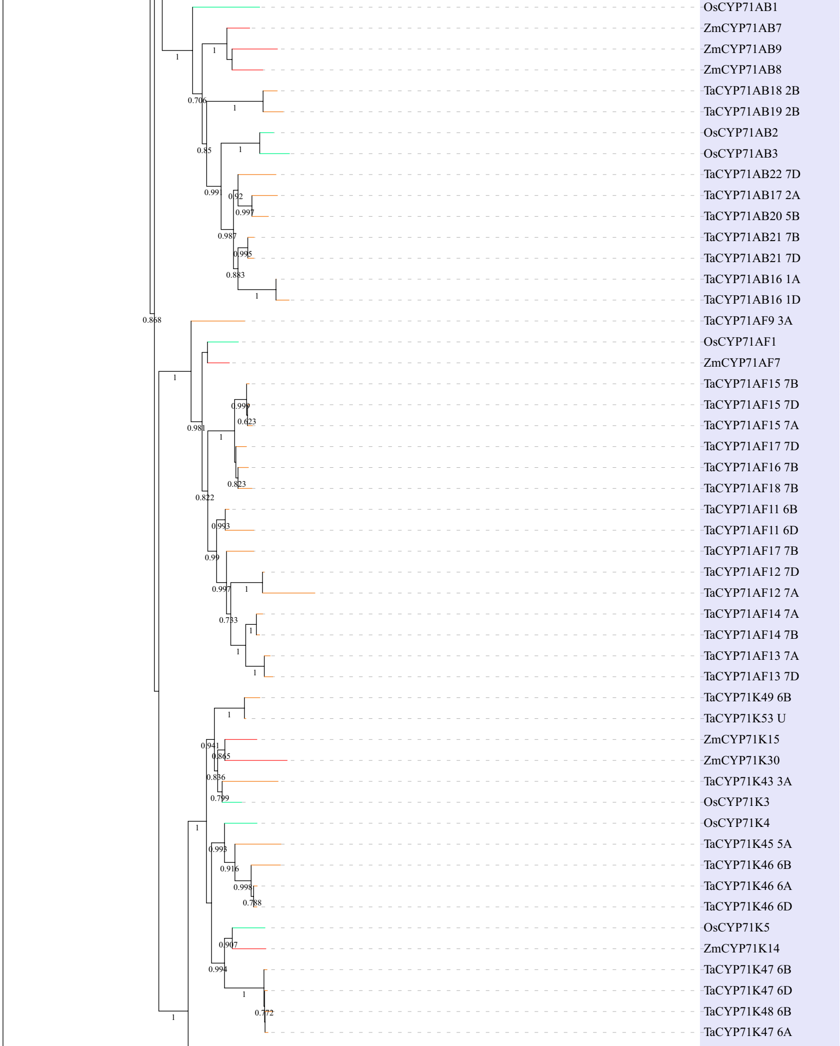
OsCYP71AB1
ZmCYP71AB7
ZmCYP71AB9
ZmCYP71AB8
TaCYP71AB18 2B
TaCYP71AB19 2B
OsCYP71AB2
OsCYP71AB3
TaCYP71AB22 7D
TaCYP71AB17 2A
TaCYP71AB20 5B
TaCYP71AB21 7B
TaCYP71AB21 7D
TaCYP71AB16 1A
TaCYP71AB16 1D
TaCYP71AF9 3A
OsCYP71AF1
ZmCYP71AF7
TaCYP71AF15 7B
TaCYP71AF15 7D
TaCYP71AF15 7A
TaCYP71AF17 7D
TaCYP71AF16 7B
TaCYP71AF18 7B
TaCYP71AF11 6B
TaCYP71AF11 6D
TaCYP71AF17 7B
TaCYP71AF12 7D
TaCYP71AF12 7A
TaCYP71AF14 7A
TaCYP71AF14 7B
TaCYP71AF13 7A
TaCYP71AF13 7D
TaCYP71K49 6B
TaCYP71K53 U
ZmCYP71K15
ZmCYP71K30
TaCYP71K43 3A
OsCYP71K3
OsCYP71K4
TaCYP71K45 5A
TaCYP71K46 6B
TaCYP71K46 6A
TaCYP71K46 6D
OsCYP71K5
ZmCYP71K14
TaCYP71K47 6B
TaCYP71K47 6D
TaCYP71K48 6B
TaCYP71K47 6A
1
1
0.706
1
0.85
1
0.991
0.92
0.997
0.987
0.995
0.883
1
0.868
1
0.999
0.623
0.981
1
0.823
0.822
0.993
0.99
0.997
1
0.733
1
1
1
1
0.941
0.865
0.836
0.799
1
0.993
0.916
0.998
0.788
0.907
0.994
1
0.772
1

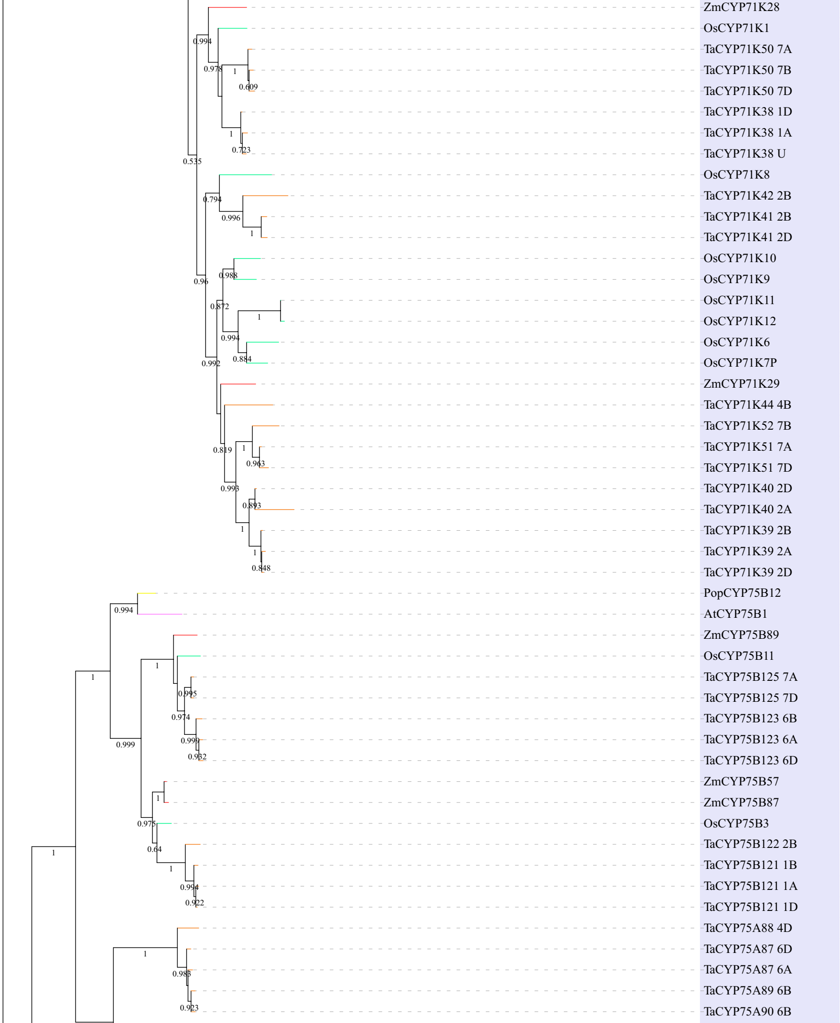
ZmCYP71K28
OsCYP71K1
TaCYP71K50 7A
TaCYP71K50 7B
TaCYP71K50 7D
TaCYP71K38 1D
TaCYP71K38 1A
TaCYP71K38 U
OsCYP71K8
TaCYP71K42 2B
TaCYP71K41 2B
TaCYP71K41 2D
OsCYP71K10
OsCYP71K9
OsCYP71K11
OsCYP71K12
OsCYP71K6
OsCYP71K7P
ZmCYP71K29
TaCYP71K44 4B
TaCYP71K52 7B
TaCYP71K51 7A
TaCYP71K51 7D
TaCYP71K40 2D
TaCYP71K40 2A
TaCYP71K39 2B
TaCYP71K39 2A
TaCYP71K39 2D
PopCYP75B12
AtCYP75B1
ZmCYP75B89
OsCYP75B11
TaCYP75B125 7A
TaCYP75B125 7D
TaCYP75B123 6B
TaCYP75B123 6A
TaCYP75B123 6D
ZmCYP75B57
ZmCYP75B87
OsCYP75B3
TaCYP75B122 2B
TaCYP75B121 1B
TaCYP75B121 1A
TaCYP75B121 1D
TaCYP75A88 4D
TaCYP75A87 6D
TaCYP75A87 6A
TaCYP75A89 6B
TaCYP75A90 6B
0.994
0.978
1
0.609
1
0.723
0.535
0.794
0.996
1
0.988
0.96
0.872
1
0.994
0.884
0.992
1
0.819
0.963
0.993
0.893
1
1
0.848
0.994
1
1
0.995
0.974
0.999
0.999
0.932
1
0.975
0.64
1
0.994
0.922
1
1
0.983
0.923

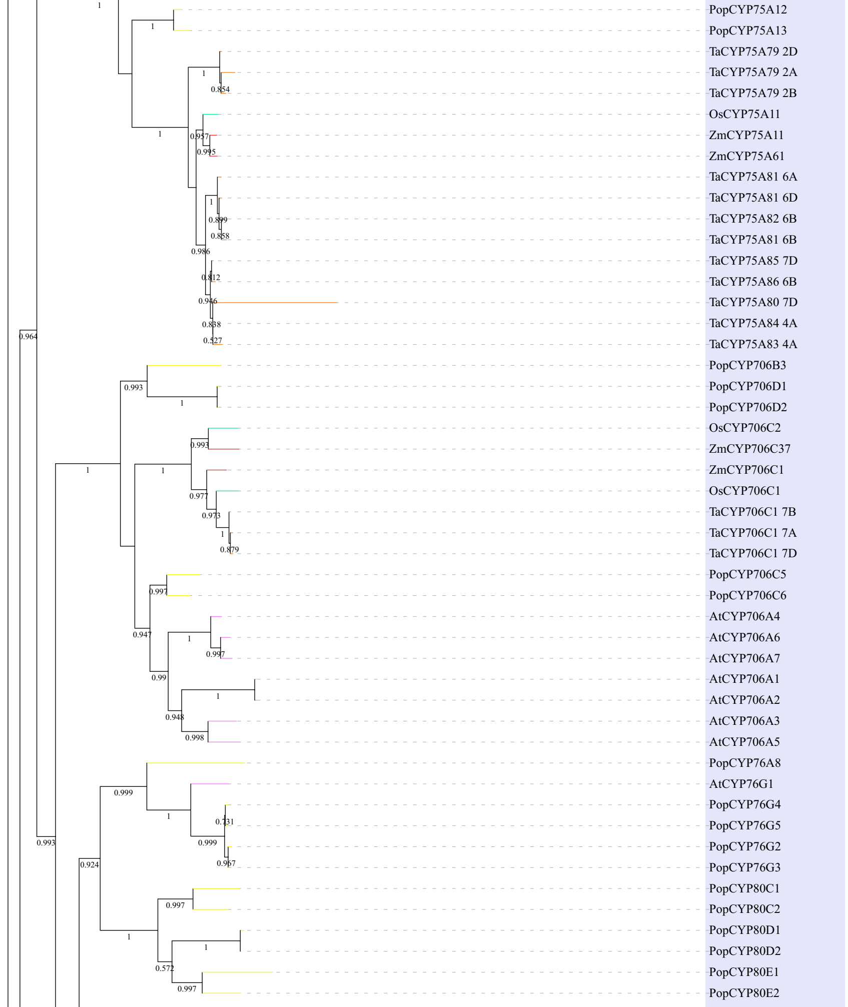
PopCYP75A12
PopCYP75A13
TaCYP75A79 2D
TaCYP75A79 2A
TaCYP75A79 2B
OsCYP75A11
ZmCYP75A11
ZmCYP75A61
TaCYP75A81 6A
TaCYP75A81 6D
TaCYP75A82 6B
TaCYP75A81 6B
TaCYP75A85 7D
TaCYP75A86 6B
TaCYP75A80 7D
TaCYP75A84 4A
TaCYP75A83 4A
PopCYP706B3
PopCYP706D1
PopCYP706D2
OsCYP706C2
ZmCYP706C37
ZmCYP706C1
OsCYP706C1
TaCYP706C1 7B
TaCYP706C1 7A
TaCYP706C1 7D
PopCYP706C5
PopCYP706C6
AtCYP706A4
AtCYP706A6
AtCYP706A7
AtCYP706A1
AtCYP706A2
AtCYP706A3
AtCYP706A5
PopCYP76A8
AtCYP76G1
PopCYP76G4
PopCYP76G5
PopCYP76G2
PopCYP76G3
PopCYP80C1
PopCYP80C2
PopCYP80D1
PopCYP80D2
PopCYP80E1
PopCYP80E2
1
1
1
0.854
1
0.957
0.995
1
0.899
0.858
0.986
0.812
0.946
0.838
0.527
0.964
0.993
1
0.993
1
1
0.977
0.973
1
0.879
0.997
0.947
1
0.997
0.99
1
0.948
0.998
0.999
1
0.731
0.999
0.967
0.993
0.924
0.997
1
1
0.572
0.997

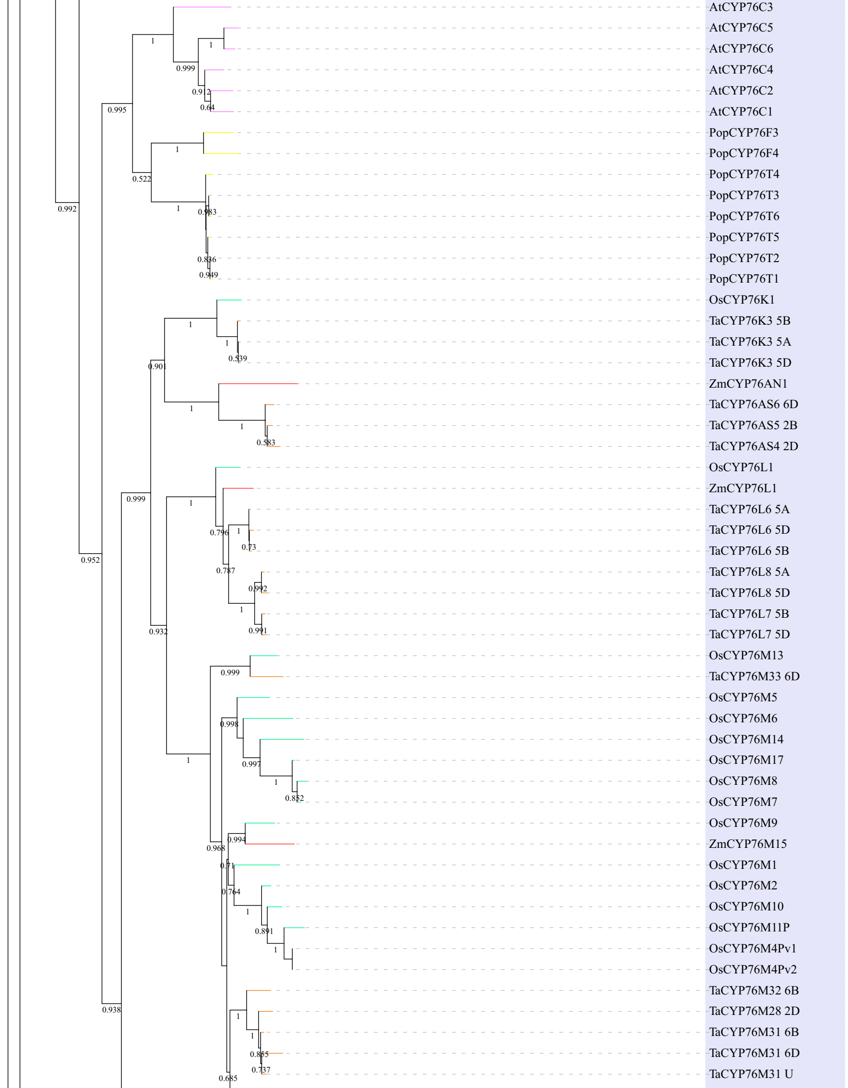
AtCYP76C3
AtCYP76C5
AtCYP76C6
AtCYP76C4
AtCYP76C2
AtCYP76C1
PopCYP76F3
PopCYP76F4
PopCYP76T4
PopCYP76T3
PopCYP76T6
PopCYP76T5
PopCYP76T2
PopCYP76T1
OsCYP76K1
TaCYP76K3 5B
TaCYP76K3 5A
TaCYP76K3 5D
ZmCYP76AN1
TaCYP76AS6 6D
TaCYP76AS5 2B
TaCYP76AS4 2D
OsCYP76L1
ZmCYP76L1
TaCYP76L6 5A
TaCYP76L6 5D
TaCYP76L6 5B
TaCYP76L8 5A
TaCYP76L8 5D
TaCYP76L7 5B
TaCYP76L7 5D
OsCYP76M13
TaCYP76M33 6D
OsCYP76M5
OsCYP76M6
OsCYP76M14
OsCYP76M17
OsCYP76M8
OsCYP76M7
OsCYP76M9
ZmCYP76M15
OsCYP76M1
OsCYP76M2
OsCYP76M10
OsCYP76M11P
OsCYP76M4Pv1
OsCYP76M4Pv2
TaCYP76M32 6B
TaCYP76M28 2D
TaCYP76M31 6B
TaCYP76M31 6D
TaCYP76M31 U
1
1
0.999
0.912
0.64
0.995
1
0.522
0.992
1
0.983
0.836
0.949
1
1
0.539
0.901
1
1
0.583
0.999
1
0.796
1
0.73
0.787
0.992
1
0.991
0.952
0.932
0.999
0.998
0.997
1
0.852
1
0.994
0.968
0.71
0.764
1
0.891
1
0.938
1
1
0.855
0.737
0.685

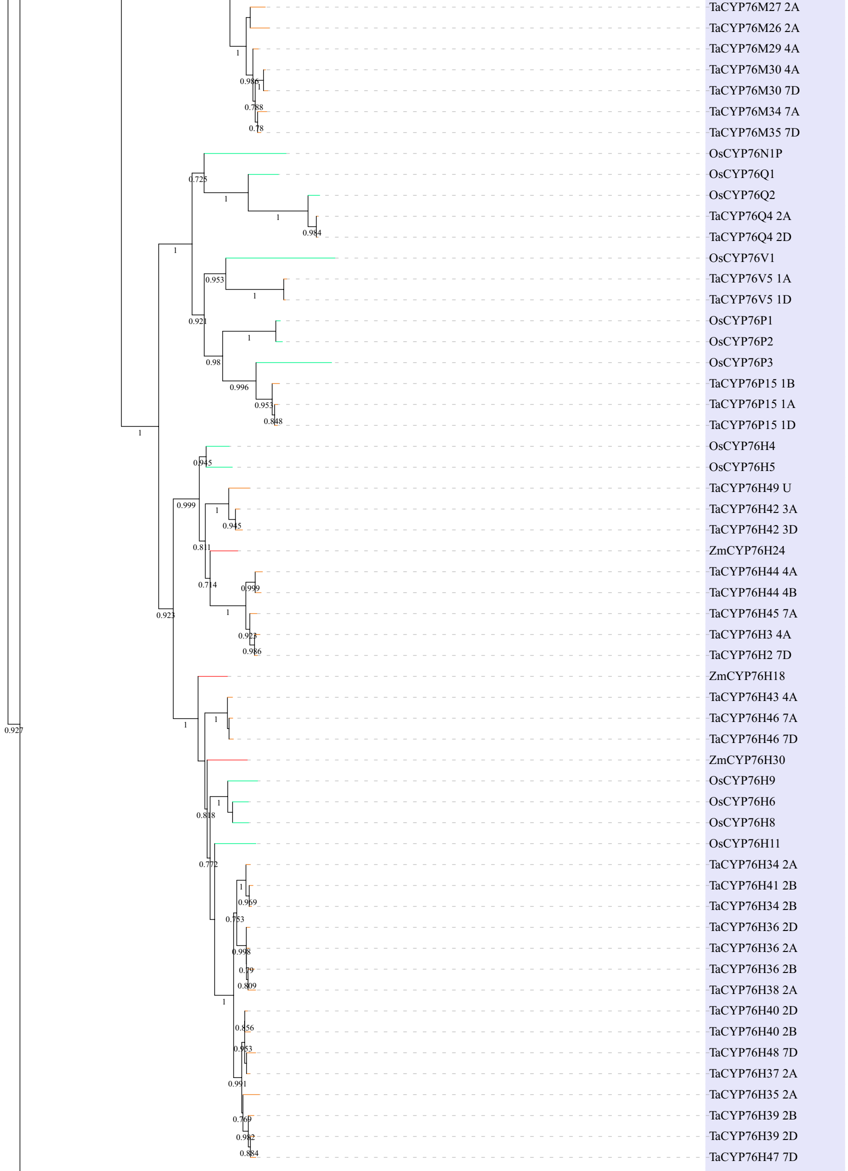
TaCYP76M27 2A
TaCYP76M26 2A
TaCYP76M29 4A
TaCYP76M30 4A
TaCYP76M30 7D
TaCYP76M34 7A
TaCYP76M35 7D
OsCYP76N1P
OsCYP76Q1
OsCYP76Q2
TaCYP76Q4 2A
TaCYP76Q4 2D
OsCYP76V1
TaCYP76V5 1A
TaCYP76V5 1D
OsCYP76P1
OsCYP76P2
OsCYP76P3
TaCYP76P15 1B
TaCYP76P15 1A
TaCYP76P15 1D
OsCYP76H4
OsCYP76H5
TaCYP76H49 U
TaCYP76H42 3A
TaCYP76H42 3D
ZmCYP76H24
TaCYP76H44 4A
TaCYP76H44 4B
TaCYP76H45 7A
TaCYP76H3 4A
TaCYP76H2 7D
ZmCYP76H18
TaCYP76H43 4A
TaCYP76H46 7A
TaCYP76H46 7D
ZmCYP76H30
OsCYP76H9
OsCYP76H6
OsCYP76H8
OsCYP76H11
TaCYP76H34 2A
TaCYP76H41 2B
TaCYP76H34 2B
TaCYP76H36 2D
TaCYP76H36 2A
TaCYP76H36 2B
TaCYP76H38 2A
TaCYP76H40 2D
TaCYP76H40 2B
TaCYP76H48 7D
TaCYP76H37 2A
TaCYP76H35 2A
TaCYP76H39 2B
TaCYP76H39 2D
TaCYP76H47 7D

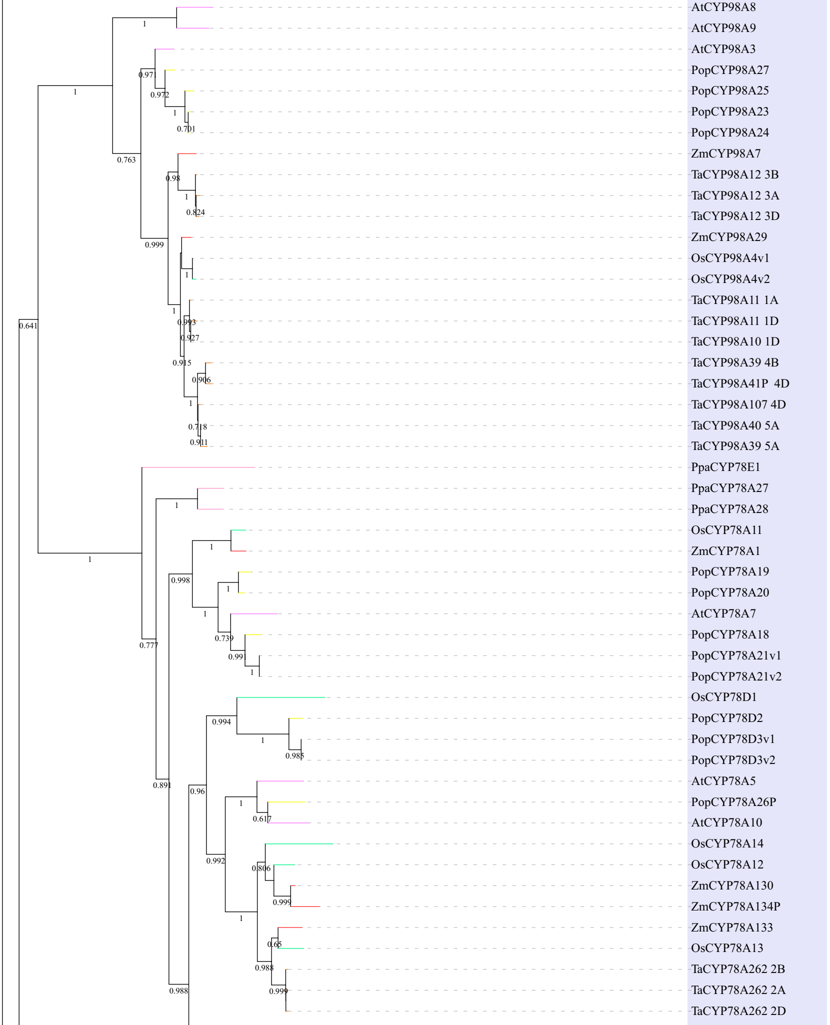
AtCYP98A8
AtCYP98A9
AtCYP98A3
PopCYP98A27
PopCYP98A25
PopCYP98A23
PopCYP98A24
ZmCYP98A7
TaCYP98A12 3B
TaCYP98A12 3A
TaCYP98A12 3D
ZmCYP98A29
OsCYP98A4v1
OsCYP98A4v2
TaCYP98A11 1A
TaCYP98A11 1D
TaCYP98A10 1D
TaCYP98A39 4B
TaCYP98A41P 4D
TaCYP98A107 4D
TaCYP98A40 5A
TaCYP98A39 5A
PpaCYP78E1
PpaCYP78A27
PpaCYP78A28
OsCYP78A11
ZmCYP78A1
PopCYP78A19
PopCYP78A20
AtCYP78A7
PopCYP78A18
PopCYP78A21v1
PopCYP78A21v2
OsCYP78D1
PopCYP78D2
PopCYP78D3v1
PopCYP78D3v2
AtCYP78A5
PopCYP78A26P
AtCYP78A10
OsCYP78A14
OsCYP78A12
ZmCYP78A130
ZmCYP78A134P
ZmCYP78A133
OsCYP78A13
TaCYP78A262 2B
TaCYP78A262 2A
TaCYP78A262 2D
1
0.971
0.972
1
0.701
1
0.763
0.98
1
0.824
0.999
1
1
0.993
0.927
0.915
0.906
1
0.718
0.911
0.641
1
1
1
0.998
1
1
0.739
0.991
1
0.777
0.994
1
0.985
0.891
0.96
1
0.617
0.992
0.806
0.999
1
0.65
0.988
0.999
0.988

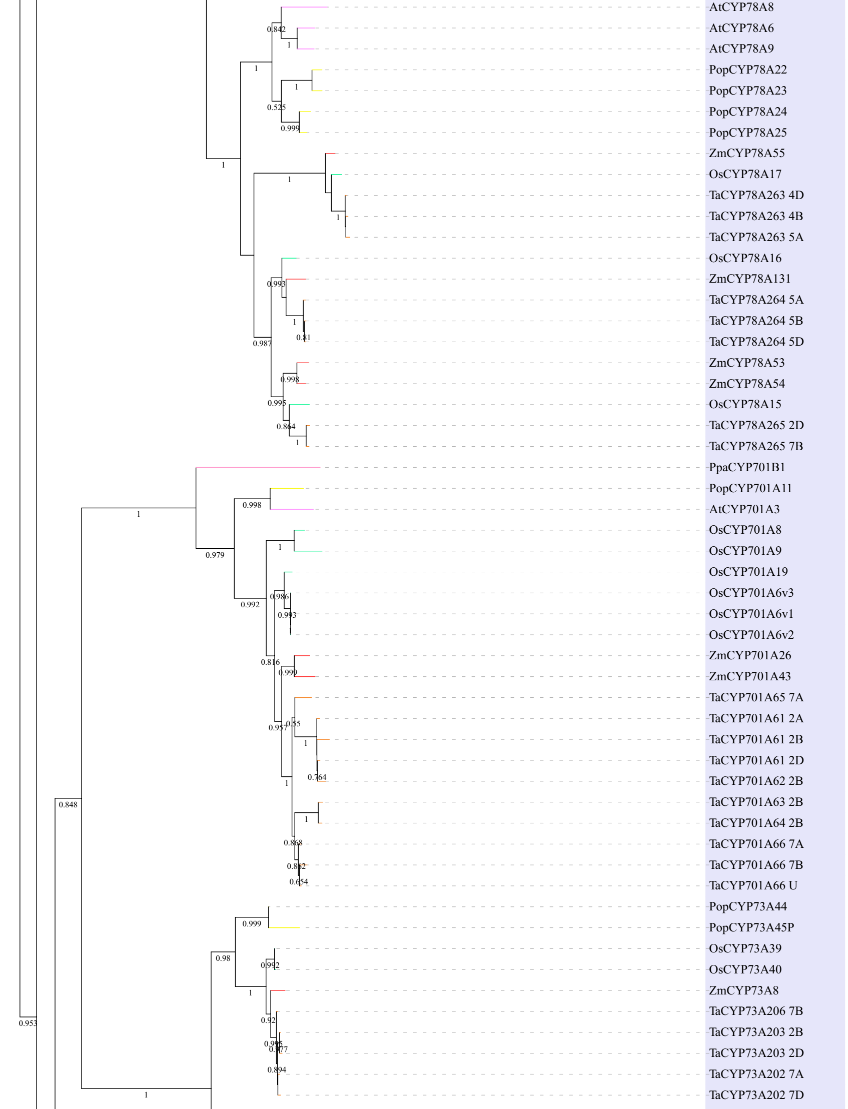
AtCYP78A8
AtCYP78A6
AtCYP78A9
PopCYP78A22
PopCYP78A23
PopCYP78A24
PopCYP78A25
ZmCYP78A55
OsCYP78A17
TaCYP78A263 4D
TaCYP78A263 4B
TaCYP78A263 5A
OsCYP78A16
ZmCYP78A131
TaCYP78A264 5A
TaCYP78A264 5B
TaCYP78A264 5D
ZmCYP78A53
ZmCYP78A54
OsCYP78A15
TaCYP78A265 2D
TaCYP78A265 7B
PpaCYP701B1
PopCYP701A11
AtCYP701A3
OsCYP701A8
OsCYP701A9
OsCYP701A19
OsCYP701A6v3
OsCYP701A6v1
OsCYP701A6v2
ZmCYP701A26
ZmCYP701A43
TaCYP701A65 7A
TaCYP701A61 2A
TaCYP701A61 2B
TaCYP701A61 2D
TaCYP701A62 2B
TaCYP701A63 2B
TaCYP701A64 2B
TaCYP701A66 7A
TaCYP701A66 7B
TaCYP701A66 U
PopCYP73A44
PopCYP73A45P
OsCYP73A39
OsCYP73A40
ZmCYP73A8
TaCYP73A206 7B
TaCYP73A203 2B
TaCYP73A203 2D
TaCYP73A202 7A
TaCYP73A202 7D

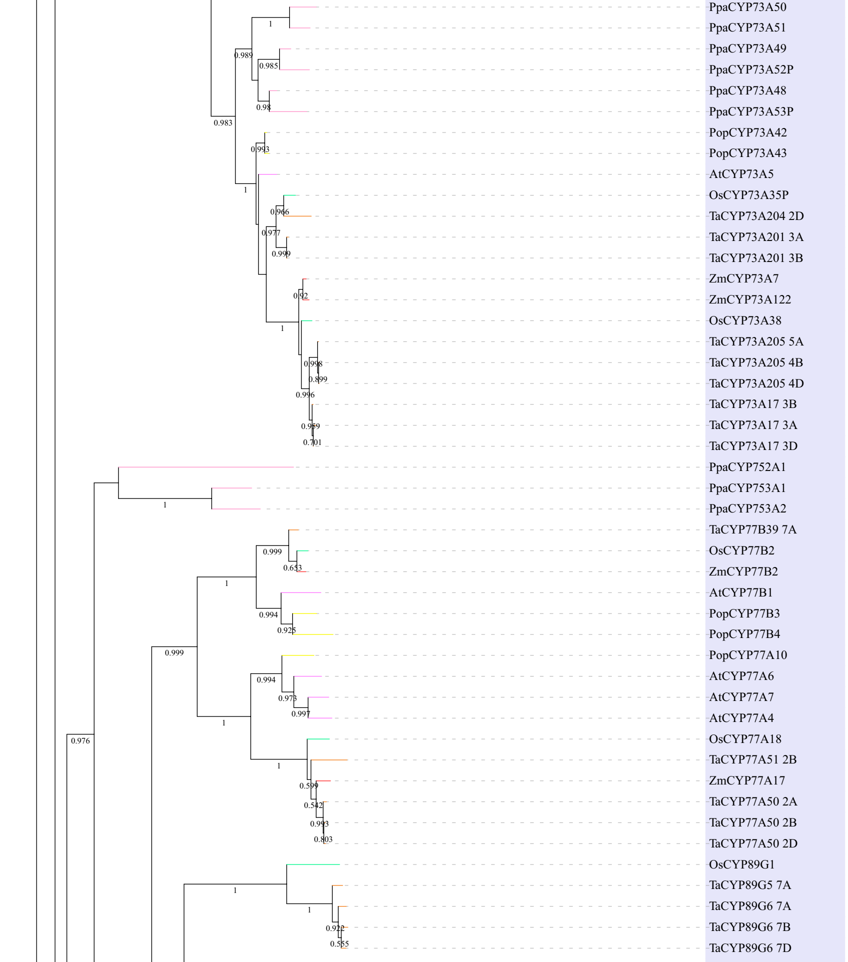
PpaCYP73A50
PpaCYP73A51
PpaCYP73A49
PpaCYP73A52P
PpaCYP73A48
PpaCYP73A53P
PopCYP73A42
PopCYP73A43
AtCYP73A5
OsCYP73A35P
TaCYP73A204 2D
TaCYP73A201 3A
TaCYP73A201 3B
ZmCYP73A7
ZmCYP73A122
OsCYP73A38
TaCYP73A205 5A
TaCYP73A205 4B
TaCYP73A205 4D
TaCYP73A17 3B
TaCYP73A17 3A
TaCYP73A17 3D
PpaCYP752A1
PpaCYP753A1
PpaCYP753A2
TaCYP77B39 7A
OsCYP77B2
ZmCYP77B2
AtCYP77B1
PopCYP77B3
PopCYP77B4
PopCYP77A10
AtCYP77A6
AtCYP77A7
AtCYP77A4
OsCYP77A18
TaCYP77A51 2B
ZmCYP77A17
TaCYP77A50 2A
TaCYP77A50 2B
TaCYP77A50 2D
OsCYP89G1
TaCYP89G5 7A
TaCYP89G6 7A
TaCYP89G6 7B
TaCYP89G6 7D

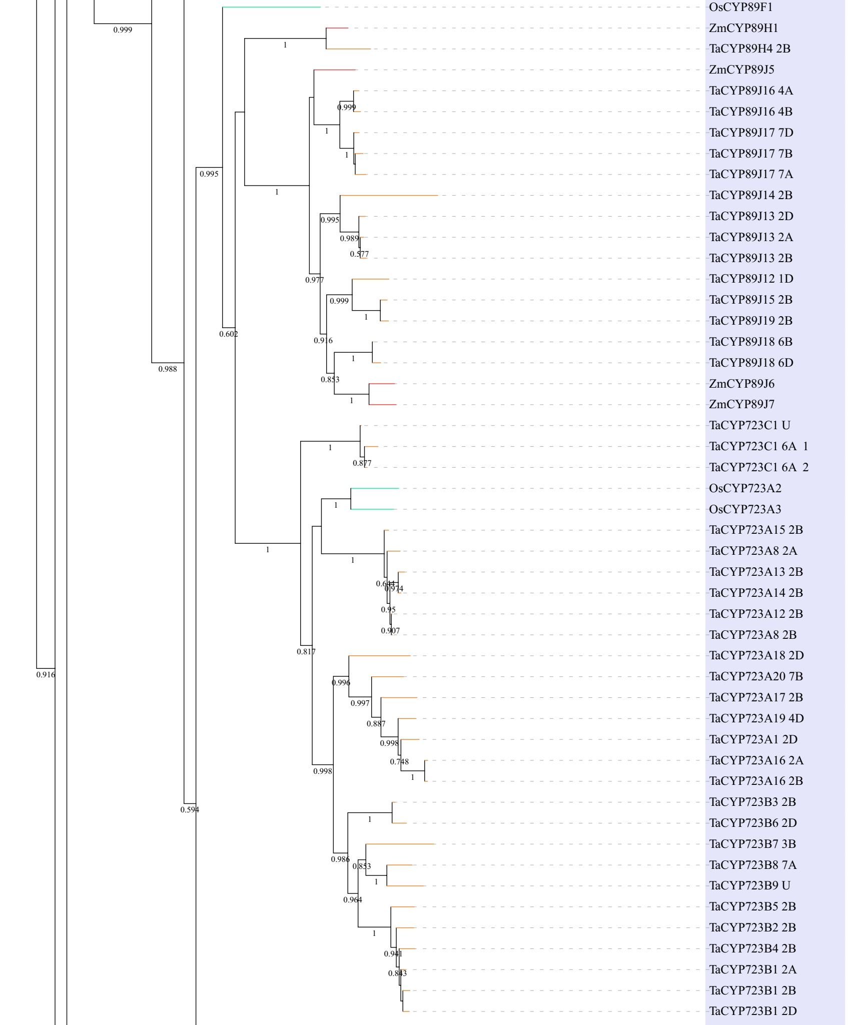

OsCYP89F1
ZmCYP89H1
TaCYP89H4 2B
ZmCYP89J5
TaCYP89J16 4A
TaCYP89J16 4B
TaCYP89J17 7D
TaCYP89J17 7B
TaCYP89J17 7A
TaCYP89J14 2B
TaCYP89J13 2D
TaCYP89J13 2A
TaCYP89J13 2B
TaCYP89J12 1D
TaCYP89J15 2B
TaCYP89J19 2B
TaCYP89J18 6B
TaCYP89J18 6D
ZmCYP89J6
ZmCYP89J7
TaCYP723C1 U
TaCYP723C1 6A 1
TaCYP723C1 6A 2
OsCYP723A2
OsCYP723A3
TaCYP723A15 2B
TaCYP723A8 2A
TaCYP723A13 2B
TaCYP723A14 2B
TaCYP723A12 2B
TaCYP723A8 2B
TaCYP723A18 2D
TaCYP723A20 7B
TaCYP723A17 2B
TaCYP723A19 4D
TaCYP723A1 2D
TaCYP723A16 2A
TaCYP723A16 2B
TaCYP723B3 2B
TaCYP723B6 2D
TaCYP723B7 3B
TaCYP723B8 7A
TaCYP723B9 U
TaCYP723B5 2B
TaCYP723B2 2B
TaCYP723B4 2B
TaCYP723B1 2A
TaCYP723B1 2B
TaCYP723B1 2D
0.999
0.995
0.602
0.988
0.916
0.594
0.999
0.995
0.989
0.577
0.977
0.999
0.916
0.853
0.877
0.817
0.644
0.974
0.95
0.907
0.996
0.997
0.887
0.998
0.748
0.998
0.986
0.853
0.964
0.941
0.843

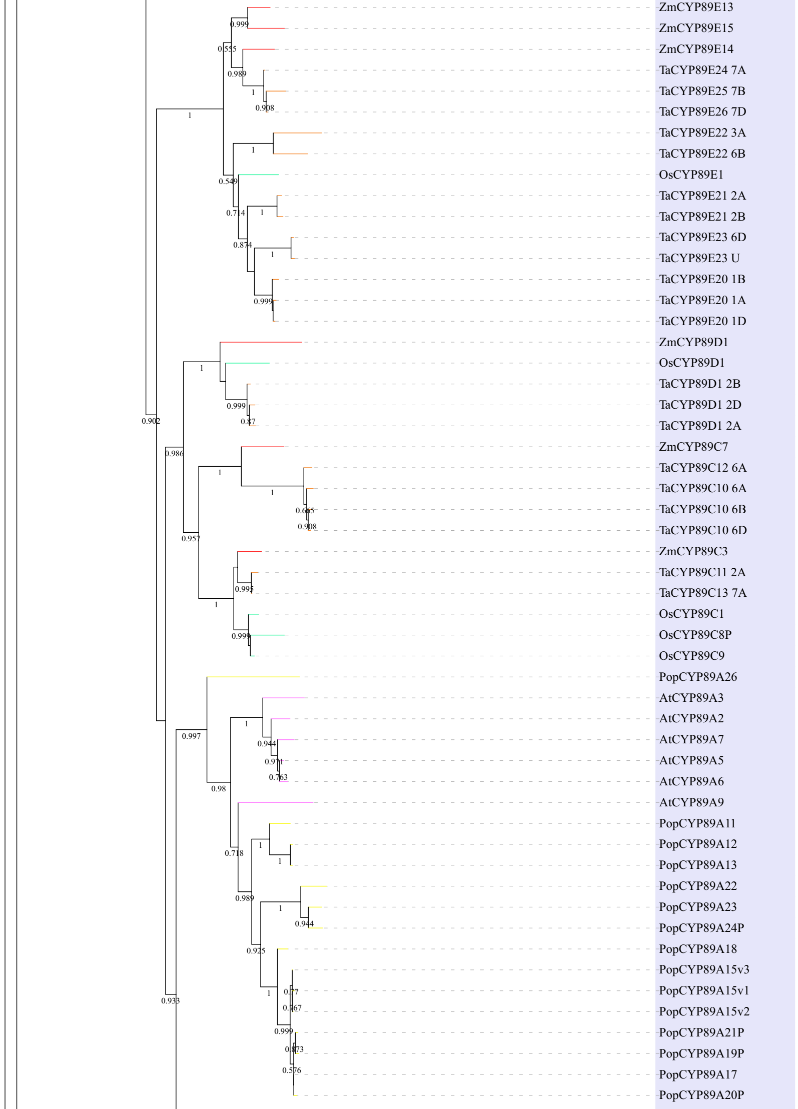
ZmCYP89E13
ZmCYP89E15
ZmCYP89E14
TaCYP89E24 7A
TaCYP89E25 7B
TaCYP89E26 7D
TaCYP89E22 3A
TaCYP89E22 6B
OsCYP89E1
TaCYP89E21 2A
TaCYP89E21 2B
TaCYP89E23 6D
TaCYP89E23 U
TaCYP89E20 1B
TaCYP89E20 1A
TaCYP89E20 1D
ZmCYP89D1
OsCYP89D1
TaCYP89D1 2B
TaCYP89D1 2D
TaCYP89D1 2A
ZmCYP89C7
TaCYP89C12 6A
TaCYP89C10 6A
TaCYP89C10 6B
TaCYP89C10 6D
ZmCYP89C3
TaCYP89C11 2A
TaCYP89C13 7A
OsCYP89C1
OsCYP89C8P
OsCYP89C9
PopCYP89A26
AtCYP89A3
AtCYP89A2
AtCYP89A7
AtCYP89A5
AtCYP89A6
AtCYP89A9
PopCYP89A11
PopCYP89A12
PopCYP89A13
PopCYP89A22
PopCYP89A23
PopCYP89A24P
PopCYP89A18
PopCYP89A15v3
PopCYP89A15v1
PopCYP89A15v2
PopCYP89A21P
PopCYP89A19P
PopCYP89A17
PopCYP89A20P
0.999
0.555
0.989
1
0.908
1
1
0.549
0.714
1
0.874
1
0.999
1
0.999
0.87
0.902
0.986
1
1
0.665
0.908
0.957
0.995
1
0.999
0.997
1
0.944
0.971
0.763
0.98
0.718
1
1
0.989
1
0.944
0.925
1
0.77
0.767
0.999
0.873
0.576
0.933

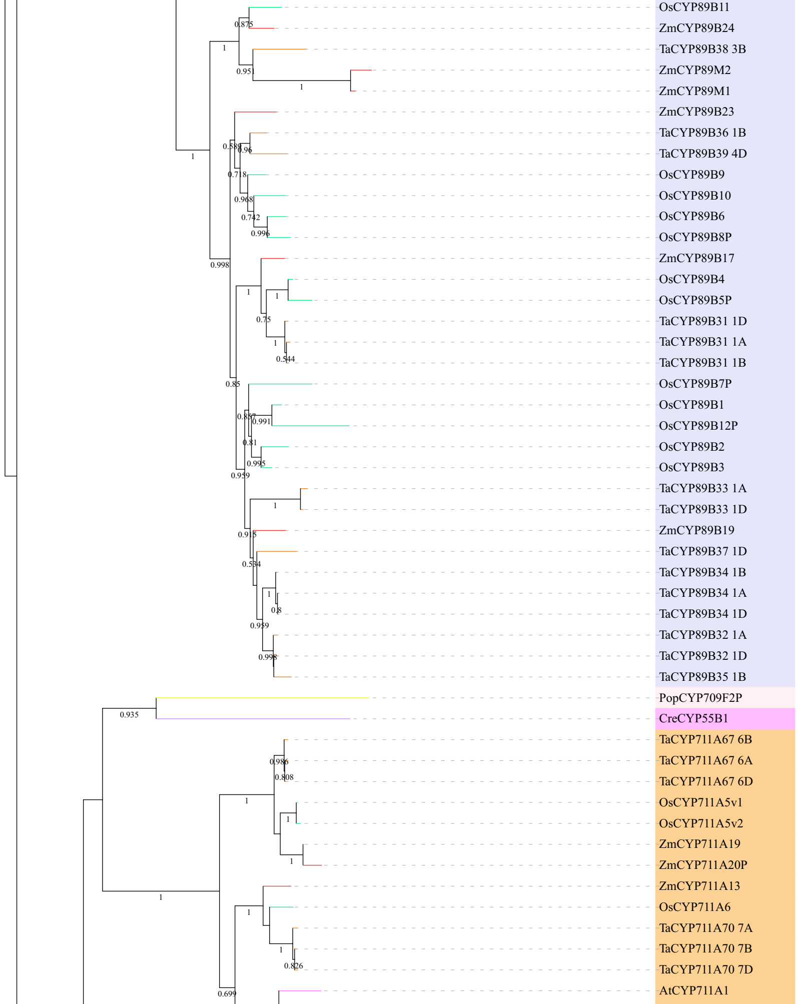
OsCYP89B11
ZmCYP89B24
TaCYP89B38 3B
ZmCYP89M2
ZmCYP89M1
ZmCYP89B23
TaCYP89B36 1B
TaCYP89B39 4D
OsCYP89B9
OsCYP89B10
OsCYP89B6
OsCYP89B8P
ZmCYP89B17
OsCYP89B4
OsCYP89B5P
TaCYP89B31 1D
TaCYP89B31 1A
TaCYP89B31 1B
OsCYP89B7P
OsCYP89B1
OsCYP89B12P
OsCYP89B2
OsCYP89B3
TaCYP89B33 1A
TaCYP89B33 1D
ZmCYP89B19
TaCYP89B37 1D
TaCYP89B34 1B
TaCYP89B34 1A
TaCYP89B34 1D
TaCYP89B32 1A
TaCYP89B32 1D
TaCYP89B35 1B
PopCYP709F2P
CreCYP55B1
TaCYP711A67 6B
TaCYP711A67 6A
TaCYP711A67 6D
OsCYP711A5v1
OsCYP711A5v2
ZmCYP711A19
ZmCYP711A20P
ZmCYP711A13
OsCYP711A6
TaCYP711A70 7A
TaCYP711A70 7B
TaCYP711A70 7D
AtCYP711A1

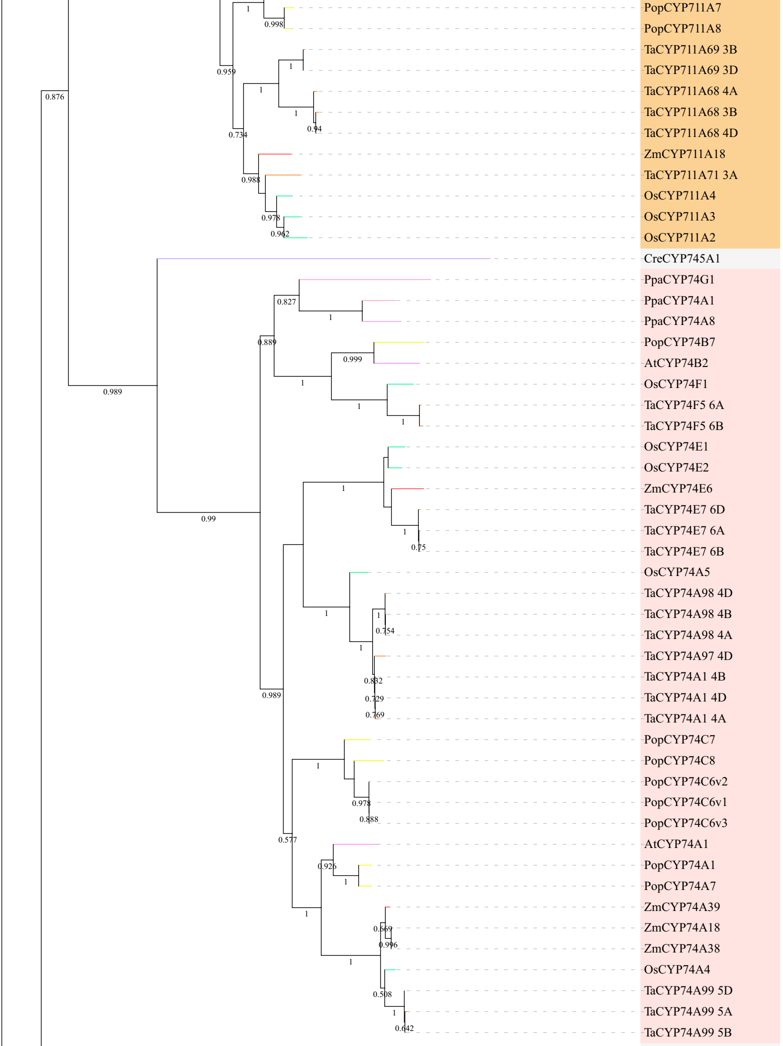
PopCYP711A7
PopCYP711A8
TaCYP711A69 3B
TaCYP711A69 3D
TaCYP711A68 4A
TaCYP711A68 3B
TaCYP711A68 4D
ZmCYP711A18
TaCYP711A71 3A
OsCYP711A4
OsCYP711A3
OsCYP711A2
CreCYP745A1
PpaCYP74G1
PpaCYP74A1
PpaCYP74A8
PopCYP74B7
AtCYP74B2
OsCYP74F1
TaCYP74F5 6A
TaCYP74F5 6B
OsCYP74E1
OsCYP74E2
ZmCYP74E6
TaCYP74E7 6D
TaCYP74E7 6A
TaCYP74E7 6B
OsCYP74A5
TaCYP74A98 4D
TaCYP74A98 4B
TaCYP74A98 4A
TaCYP74A97 4D
TaCYP74A1 4B
TaCYP74A1 4D
TaCYP74A1 4A
PopCYP74C7
PopCYP74C8
PopCYP74C6v2
PopCYP74C6v1
PopCYP74C6v3
AtCYP74A1
PopCYP74A1
PopCYP74A7
ZmCYP74A39
ZmCYP74A18
ZmCYP74A38
OsCYP74A4
TaCYP74A99 5D
TaCYP74A99 5A
TaCYP74A99 5B
0.876
0.989
0.99
0.959
0.734
0.998
0.988
0.978
0.962
0.94
0.827
0.889
0.999
0.989
0.75
0.754
0.832
0.729
0.769
0.978
0.888
0.577
0.926
0.669
0.996
0.508
0.642

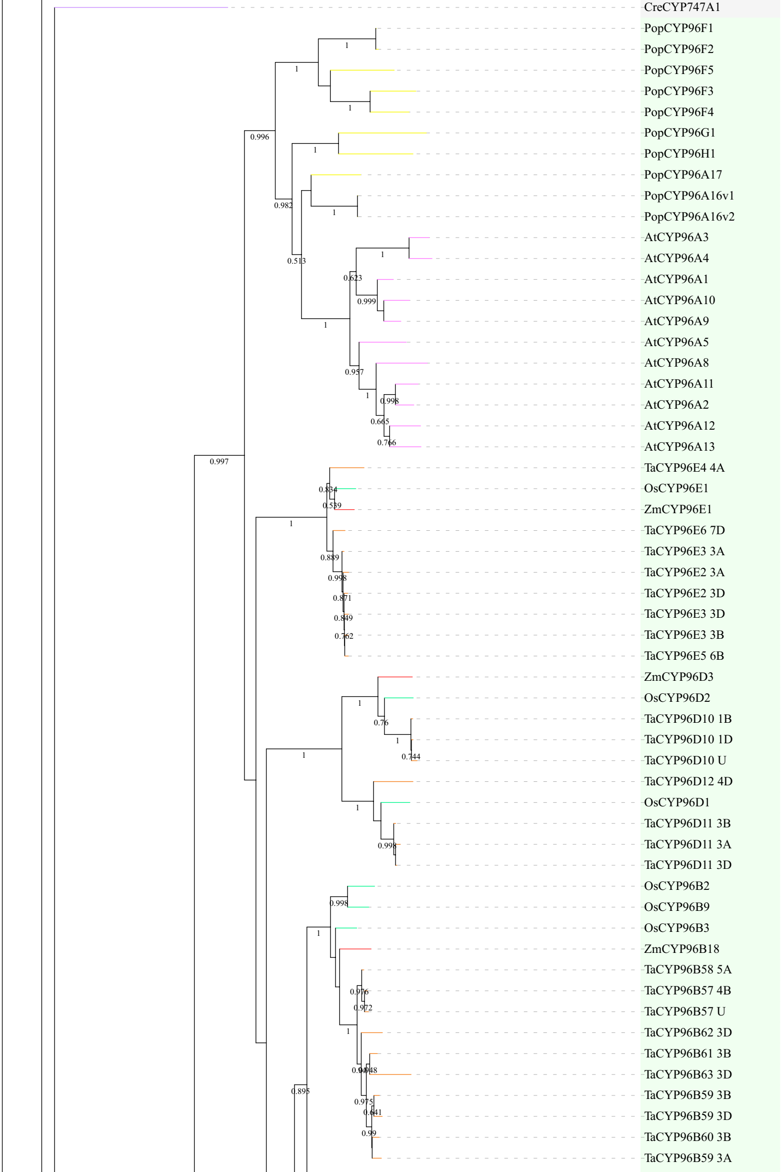
CreCYP747A1
PopCYP96F1
PopCYP96F2
PopCYP96F5
PopCYP96F3
PopCYP96F4
PopCYP96G1
PopCYP96H1
PopCYP96A17
PopCYP96A16v1
PopCYP96A16v2
AtCYP96A3
AtCYP96A4
AtCYP96A1
AtCYP96A10
AtCYP96A9
AtCYP96A5
AtCYP96A8
AtCYP96A11
AtCYP96A2
AtCYP96A12
AtCYP96A13
TaCYP96E4 4A
OsCYP96E1
ZmCYP96E1
TaCYP96E6 7D
TaCYP96E3 3A
TaCYP96E2 3A
TaCYP96E2 3D
TaCYP96E3 3D
TaCYP96E3 3B
TaCYP96E5 6B
ZmCYP96D3
OsCYP96D2
TaCYP96D10 1B
TaCYP96D10 1D
TaCYP96D10 U
TaCYP96D12 4D
OsCYP96D1
TaCYP96D11 3B
TaCYP96D11 3A
TaCYP96D11 3D
OsCYP96B2
OsCYP96B9
OsCYP96B3
ZmCYP96B18
TaCYP96B58 5A
TaCYP96B57 4B
TaCYP96B57 U
TaCYP96B62 3D
TaCYP96B61 3B
TaCYP96B63 3D
TaCYP96B59 3B
TaCYP96B59 3D
TaCYP96B60 3B
TaCYP96B59 3A

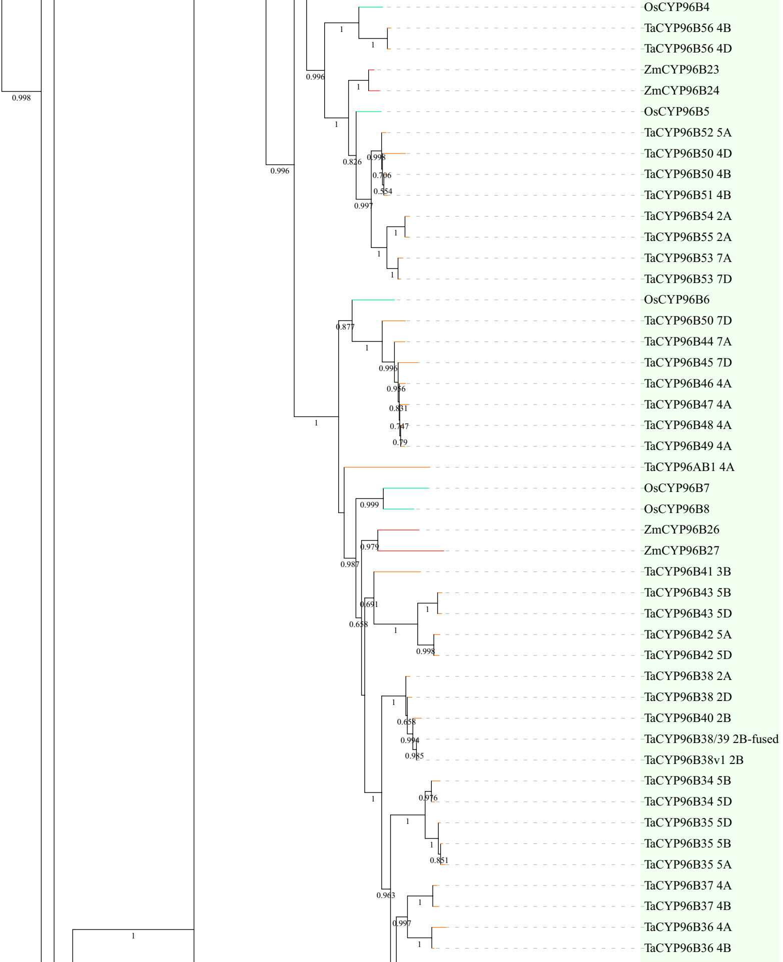

OsCYP96B4
TaCYP96B56 4B
TaCYP96B56 4D
ZmCYP96B23
ZmCYP96B24
OsCYP96B5
TaCYP96B52 5A
TaCYP96B50 4D
TaCYP96B50 4B
TaCYP96B51 4B
TaCYP96B54 2A
TaCYP96B55 2A
TaCYP96B53 7A
TaCYP96B53 7D
OsCYP96B6
TaCYP96B50 7D
TaCYP96B44 7A
TaCYP96B45 7D
TaCYP96B46 4A
TaCYP96B47 4A
TaCYP96B48 4A
TaCYP96B49 4A
TaCYP96AB1 4A
OsCYP96B7
OsCYP96B8
ZmCYP96B26
ZmCYP96B27
TaCYP96B41 3B
TaCYP96B43 5B
TaCYP96B43 5D
TaCYP96B42 5A
TaCYP96B42 5D
TaCYP96B38 2A
TaCYP96B38 2D
TaCYP96B40 2B
TaCYP96B38/39 2B-fused
TaCYP96B38v1 2B
TaCYP96B34 5B
TaCYP96B34 5D
TaCYP96B35 5D
TaCYP96B35 5B
TaCYP96B35 5A
TaCYP96B37 4A
TaCYP96B37 4B
TaCYP96B36 4A
TaCYP96B36 4B
0.998
0.996
0.996
1
0.826
0.998
0.706
0.554
0.997
0.877
0.996
0.956
0.831
0.747
0.79
0.999
0.979
0.987
0.691
0.658
0.998
0.658
0.994
0.985
0.976
0.851
0.963
0.997

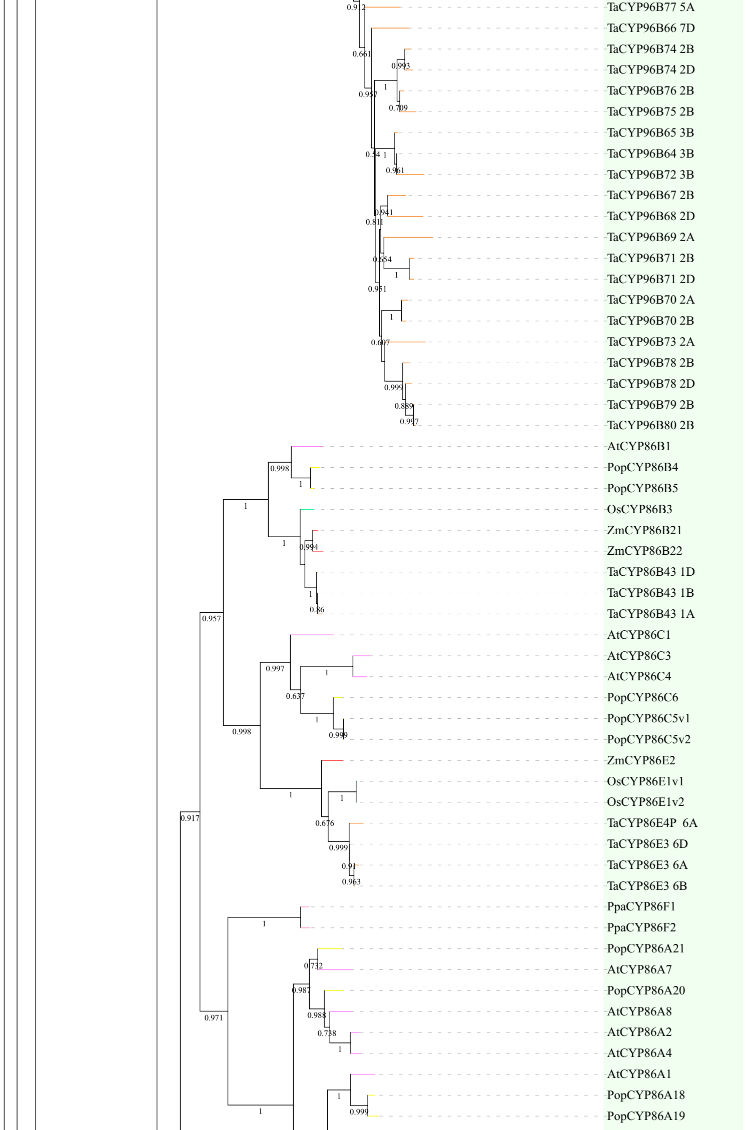

TaCYP96B77 5A
TaCYP96B66 7D
TaCYP96B74 2B
TaCYP96B74 2D
TaCYP96B76 2B
TaCYP96B75 2B
TaCYP96B65 3B
TaCYP96B64 3B
TaCYP96B72 3B
TaCYP96B67 2B
TaCYP96B68 2D
TaCYP96B69 2A
TaCYP96B71 2B
TaCYP96B71 2D
TaCYP96B70 2A
TaCYP96B70 2B
TaCYP96B73 2A
TaCYP96B78 2B
TaCYP96B78 2D
TaCYP96B79 2B
TaCYP96B80 2B
AtCYP86B1
PopCYP86B4
PopCYP86B5
OsCYP86B3
ZmCYP86B21
ZmCYP86B22
TaCYP86B43 1D
TaCYP86B43 1B
TaCYP86B43 1A
AtCYP86C1
AtCYP86C3
AtCYP86C4
PopCYP86C6
PopCYP86C5v1
PopCYP86C5v2
ZmCYP86E2
OsCYP86E1v1
OsCYP86E1v2
TaCYP86E4P 6A
TaCYP86E3 6D
TaCYP86E3 6A
TaCYP86E3 6B
PpaCYP86F1
PpaCYP86F2
PopCYP86A21
AtCYP86A7
PopCYP86A20
AtCYP86A8
AtCYP86A2
AtCYP86A4
AtCYP86A1
PopCYP86A18
PopCYP86A19

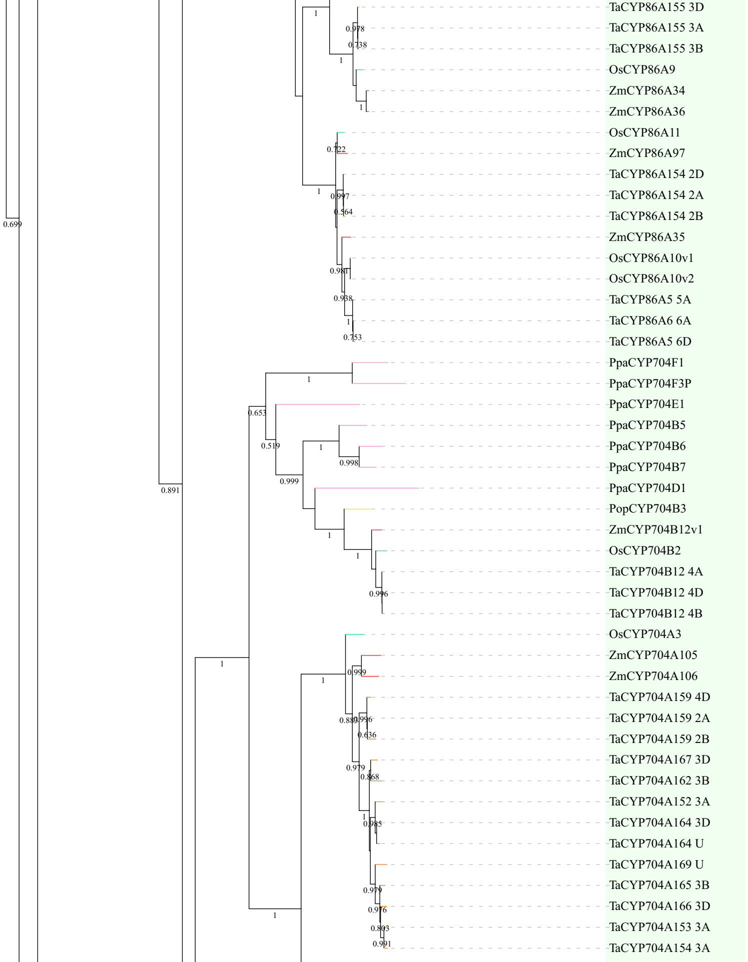
TaCYP86A155 3D
TaCYP86A155 3A
TaCYP86A155 3B
OsCYP86A9
ZmCYP86A34
ZmCYP86A36
OsCYP86A11
ZmCYP86A97
TaCYP86A154 2D
TaCYP86A154 2A
TaCYP86A154 2B
ZmCYP86A35
OsCYP86A10v1
OsCYP86A10v2
TaCYP86A5 5A
TaCYP86A6 6A
TaCYP86A5 6D
PpaCYP704F1
PpaCYP704F3P
PpaCYP704E1
PpaCYP704B5
PpaCYP704B6
PpaCYP704B7
PpaCYP704D1
PopCYP704B3
ZmCYP704B12v1
OsCYP704B2
TaCYP704B12 4A
TaCYP704B12 4D
TaCYP704B12 4B
OsCYP704A3
ZmCYP704A105
ZmCYP704A106
TaCYP704A159 4D
TaCYP704A159 2A
TaCYP704A159 2B
TaCYP704A167 3D
TaCYP704A162 3B
TaCYP704A152 3A
TaCYP704A164 3D
TaCYP704A164 U
TaCYP704A169 U
TaCYP704A165 3B
TaCYP704A166 3D
TaCYP704A153 3A
TaCYP704A154 3A

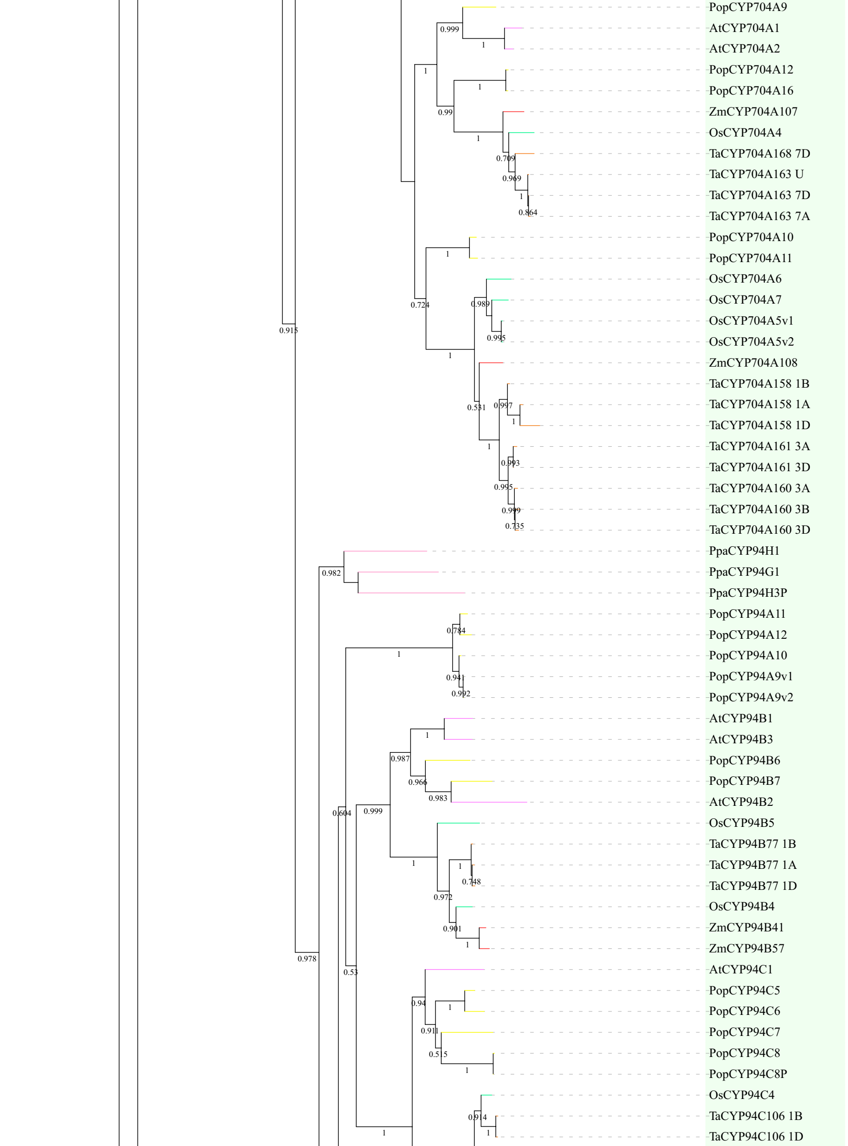

PopCYP704A9
AtCYP704A1
AtCYP704A2
PopCYP704A12
PopCYP704A16
ZmCYP704A107
OsCYP704A4
TaCYP704A168 7D
TaCYP704A163 U
TaCYP704A163 7D
TaCYP704A163 7A
PopCYP704A10
PopCYP704A11
OsCYP704A6
OsCYP704A7
OsCYP704A5v1
OsCYP704A5v2
ZmCYP704A108
TaCYP704A158 1B
TaCYP704A158 1A
TaCYP704A158 1D
TaCYP704A161 3A
TaCYP704A161 3D
TaCYP704A160 3A
TaCYP704A160 3B
TaCYP704A160 3D
PpaCYP94H1
PpaCYP94G1
PpaCYP94H3P
PopCYP94A11
PopCYP94A12
PopCYP94A10
PopCYP94A9v1
PopCYP94A9v2
AtCYP94B1
AtCYP94B3
PopCYP94B6
PopCYP94B7
AtCYP94B2
OsCYP94B5
TaCYP94B77 1B
TaCYP94B77 1A
TaCYP94B77 1D
OsCYP94B4
ZmCYP94B41
ZmCYP94B57
AtCYP94C1
PopCYP94C5
PopCYP94C6
PopCYP94C7
PopCYP94C8
PopCYP94C8P
OsCYP94C4
TaCYP94C106 1B
TaCYP94C106 1D

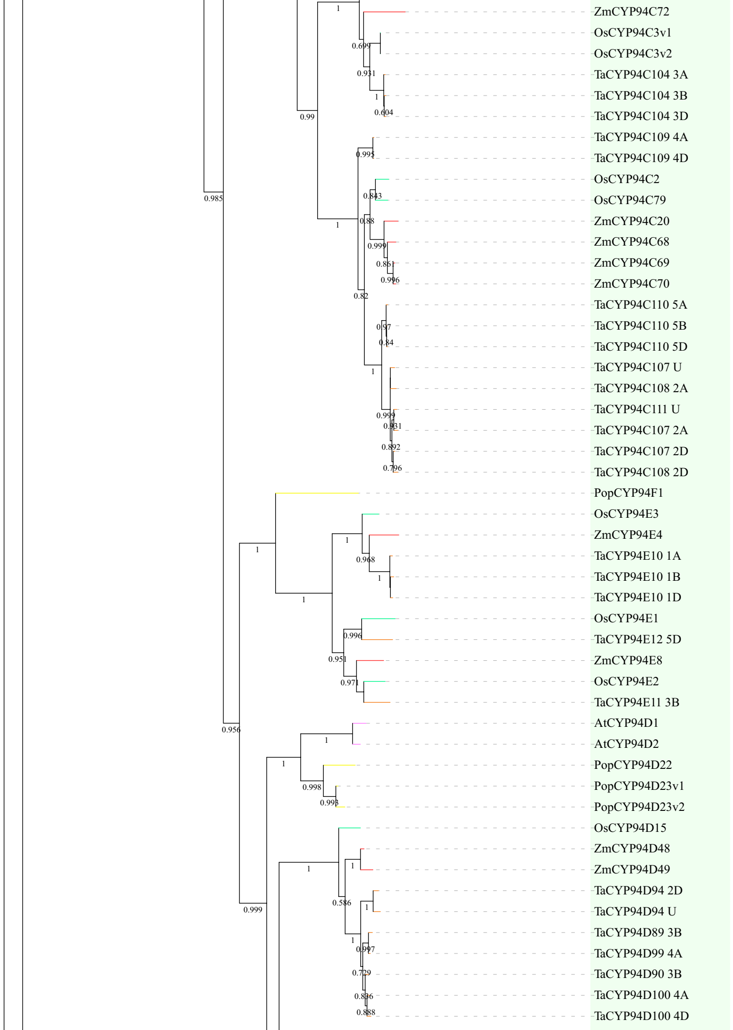
ZmCYP94C72
OsCYP94C3v1
OsCYP94C3v2
TaCYP94C104 3A
TaCYP94C104 3B
TaCYP94C104 3D
TaCYP94C109 4A
TaCYP94C109 4D
OsCYP94C2
OsCYP94C79
ZmCYP94C20
ZmCYP94C68
ZmCYP94C69
ZmCYP94C70
TaCYP94C110 5A
TaCYP94C110 5B
TaCYP94C110 5D
TaCYP94C107 U
TaCYP94C108 2A
TaCYP94C111 U
TaCYP94C107 2A
TaCYP94C107 2D
TaCYP94C108 2D
PopCYP94F1
OsCYP94E3
ZmCYP94E4
TaCYP94E10 1A
TaCYP94E10 1B
TaCYP94E10 1D
OsCYP94E1
TaCYP94E12 5D
ZmCYP94E8
OsCYP94E2
TaCYP94E11 3B
AtCYP94D1
AtCYP94D2
PopCYP94D22
PopCYP94D23v1
PopCYP94D23v2
OsCYP94D15
ZmCYP94D48
ZmCYP94D49
TaCYP94D94 2D
TaCYP94D94 U
TaCYP94D89 3B
TaCYP94D99 4A
TaCYP94D90 3B
TaCYP94D100 4A
TaCYP94D100 4D

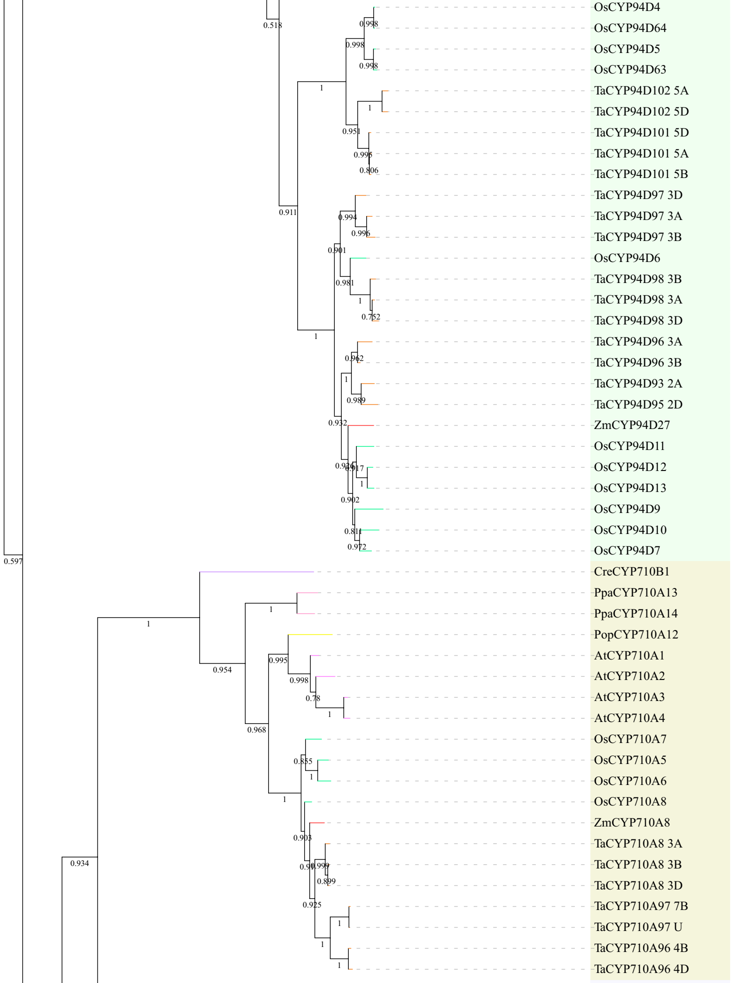
OsCYP94D4
OsCYP94D64
OsCYP94D5
OsCYP94D63
TaCYP94D102 5A
TaCYP94D102 5D
TaCYP94D101 5D
TaCYP94D101 5A
TaCYP94D101 5B
TaCYP94D97 3D
TaCYP94D97 3A
TaCYP94D97 3B
OsCYP94D6
TaCYP94D98 3B
TaCYP94D98 3A
TaCYP94D98 3D
TaCYP94D96 3A
TaCYP94D96 3B
TaCYP94D93 2A
TaCYP94D95 2D
ZmCYP94D27
OsCYP94D11
OsCYP94D12
OsCYP94D13
OsCYP94D9
OsCYP94D10
OsCYP94D7
CreCYP710B1
PpaCYP710A13
PpaCYP710A14
PopCYP710A12
AtCYP710A1
AtCYP710A2
AtCYP710A3
AtCYP710A4
OsCYP710A7
OsCYP710A5
OsCYP710A6
OsCYP710A8
ZmCYP710A8
TaCYP710A8 3A
TaCYP710A8 3B
TaCYP710A8 3D
TaCYP710A97 7B
TaCYP710A97 U
TaCYP710A96 4B
TaCYP710A96 4D
0.518
0.998
0.998
0.998
1
1
0.951
0.995
0.806
0.911
0.994
0.996
0.901
0.981
1
0.752
1
0.962
1
0.989
0.932
0.917
1
0.902
0.811
0.972
0.597
1
1
0.954
0.995
0.998
0.78
1
0.968
0.855
1
1
0.903
0.999
0.899
0.925
1
1
1
0.934

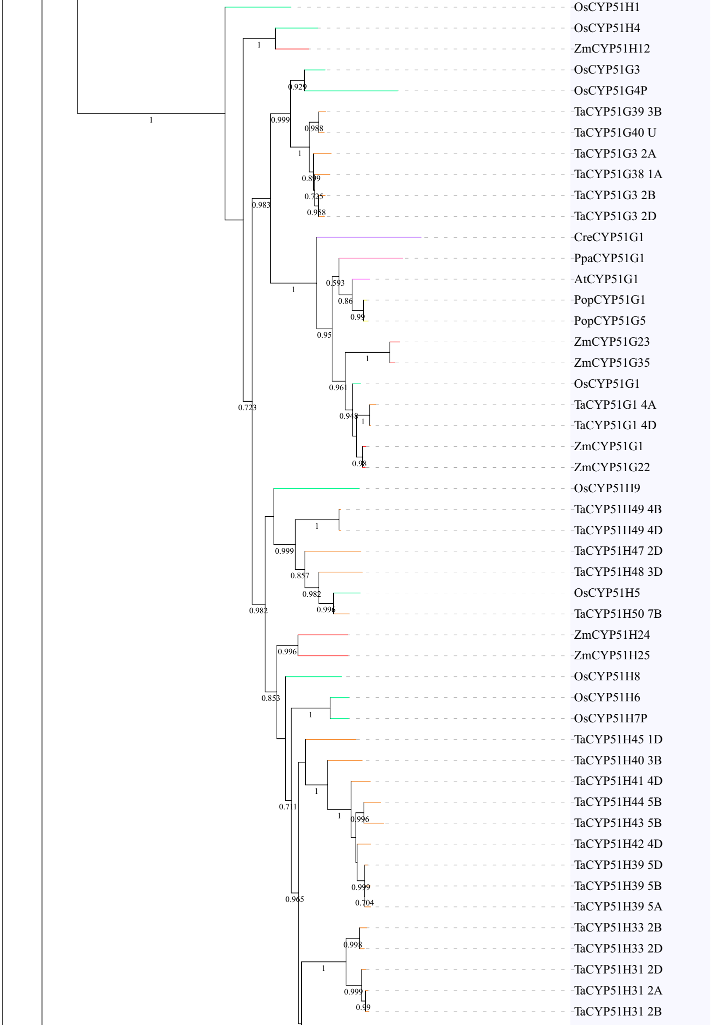
OsCYP51H1
OsCYP51H4
ZmCYP51H12
OsCYP51G3
OsCYP51G4P
TaCYP51G39 3B
TaCYP51G40 U
TaCYP51G3 2A
TaCYP51G38 1A
TaCYP51G3 2B
TaCYP51G3 2D
CreCYP51G1
PpaCYP51G1
AtCYP51G1
PopCYP51G1
PopCYP51G5
ZmCYP51G23
ZmCYP51G35
OsCYP51G1
TaCYP51G1 4A
TaCYP51G1 4D
ZmCYP51G1
ZmCYP51G22
OsCYP51H9
TaCYP51H49 4B
TaCYP51H49 4D
TaCYP51H47 2D
TaCYP51H48 3D
OsCYP51H5
TaCYP51H50 7B
ZmCYP51H24
ZmCYP51H25
OsCYP51H8
OsCYP51H6
OsCYP51H7P
TaCYP51H45 1D
TaCYP51H40 3B
TaCYP51H41 4D
TaCYP51H44 5B
TaCYP51H43 5B
TaCYP51H42 4D
TaCYP51H39 5D
TaCYP51H39 5B
TaCYP51H39 5A
TaCYP51H33 2B
TaCYP51H33 2D
TaCYP51H31 2D
TaCYP51H31 2A
TaCYP51H31 2B

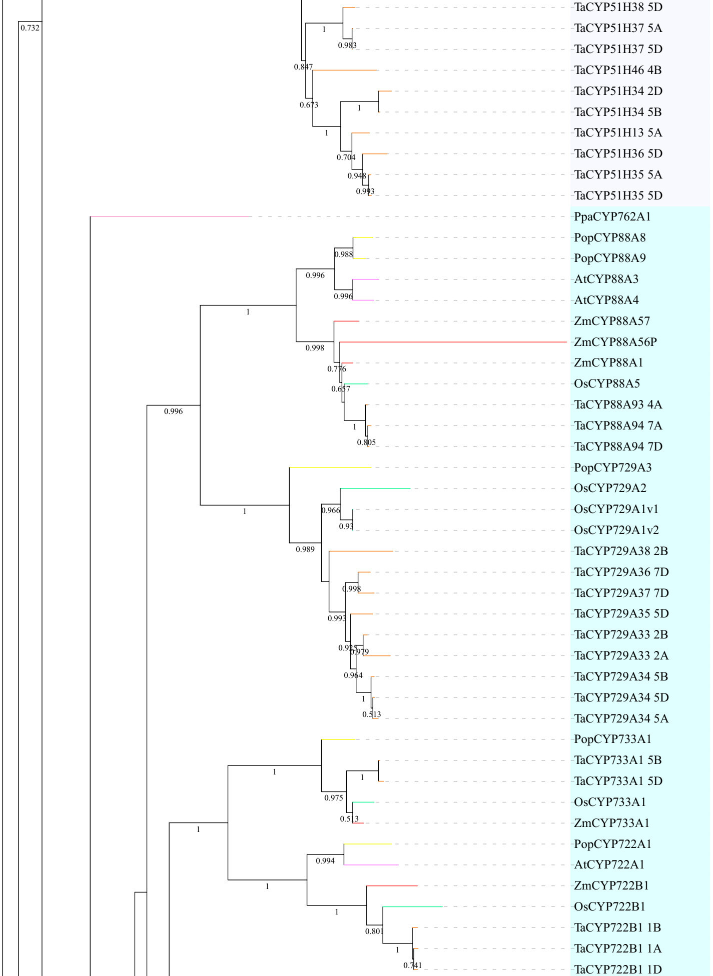
TaCYP51H38 5D
TaCYP51H37 5A
TaCYP51H37 5D
TaCYP51H46 4B
TaCYP51H34 2D
TaCYP51H34 5B
TaCYP51H13 5A
TaCYP51H36 5D
TaCYP51H35 5A
TaCYP51H35 5D
PpaCYP762A1
PopCYP88A8
PopCYP88A9
AtCYP88A3
AtCYP88A4
ZmCYP88A57
ZmCYP88A56P
ZmCYP88A1
OsCYP88A5
TaCYP88A93 4A
TaCYP88A94 7A
TaCYP88A94 7D
PopCYP729A3
OsCYP729A2
OsCYP729A1v1
OsCYP729A1v2
TaCYP729A38 2B
TaCYP729A36 7D
TaCYP729A37 7D
TaCYP729A35 5D
TaCYP729A33 2B
TaCYP729A33 2A
TaCYP729A34 5B
TaCYP729A34 5D
TaCYP729A34 5A
PopCYP733A1
TaCYP733A1 5B
TaCYP733A1 5D
OsCYP733A1
ZmCYP733A1
PopCYP722A1
AtCYP722A1
ZmCYP722B1
OsCYP722B1
TaCYP722B1 1B
TaCYP722B1 1A
TaCYP722B1 1D

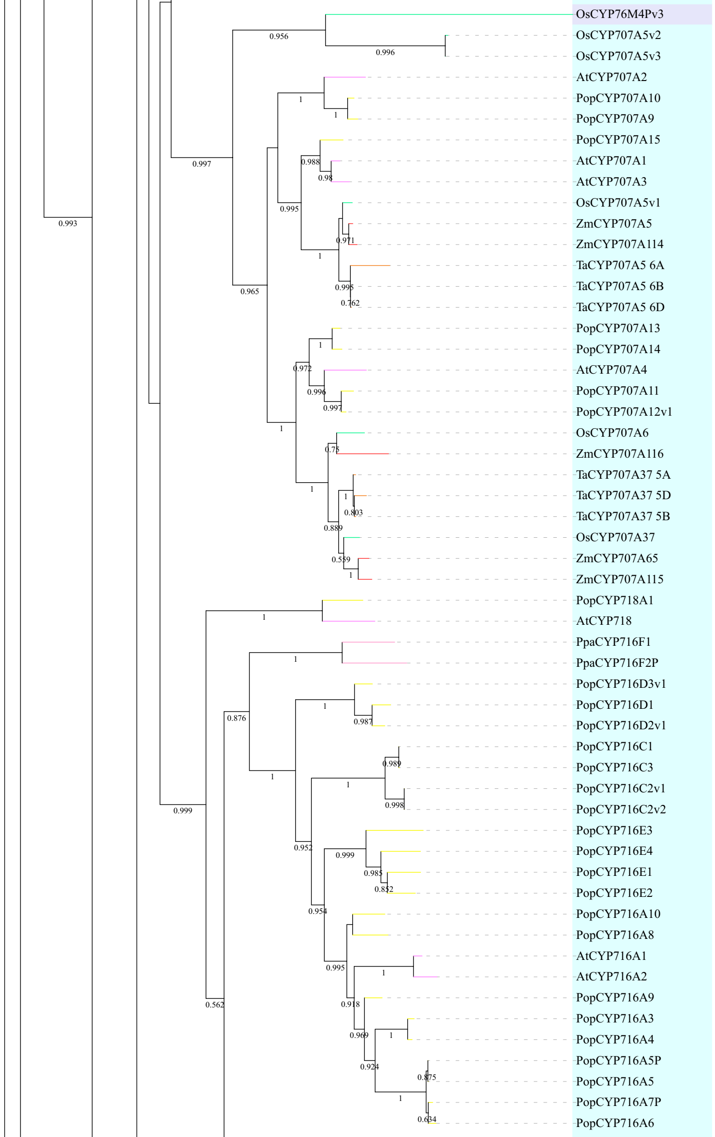
OsCYP76M4Pv3
0.956
OsCYP707A5v2
0.996
OsCYP707A5v3
AtCYP707A2
1
PopCYP707A10
1
PopCYP707A9
PopCYP707A15
0.997
AtCYP707A1
0.988
0.98
AtCYP707A3
OsCYP707A5v1
0.995
0.993
ZmCYP707A5
0.971
ZmCYP707A114
1
TaCYP707A5 6A
TaCYP707A5 6B
0.995
0.965
0.762
TaCYP707A5 6D
PopCYP707A13
1
PopCYP707A14
0.972
AtCYP707A4
0.996
PopCYP707A11
0.997
PopCYP707A12v1
1
OsCYP707A6
0.75
ZmCYP707A116
TaCYP707A37 5A
1
1
TaCYP707A37 5D
0.803
TaCYP707A37 5B
0.889
OsCYP707A37
0.559
ZmCYP707A65
1
ZmCYP707A115
PopCYP718A1
1
AtCYP718
PpaCYP716F1
1
PpaCYP716F2P
PopCYP716D3v1
1
PopCYP716D1
0.876
0.987
PopCYP716D2v1
PopCYP716C1
0.989
PopCYP716C3
1
1
PopCYP716C2v1
0.999
0.998
PopCYP716C2v2
PopCYP716E3
0.952
0.999
PopCYP716E4
0.985
PopCYP716E1
0.852
PopCYP716E2
0.954
PopCYP716A10
PopCYP716A8
AtCYP716A1
0.995
1
AtCYP716A2
0.562
0.918
PopCYP716A9
PopCYP716A3
0.969
1
PopCYP716A4
0.924
PopCYP716A5P
0.875
PopCYP716A5
1
PopCYP716A7P
0.634
PopCYP716A6

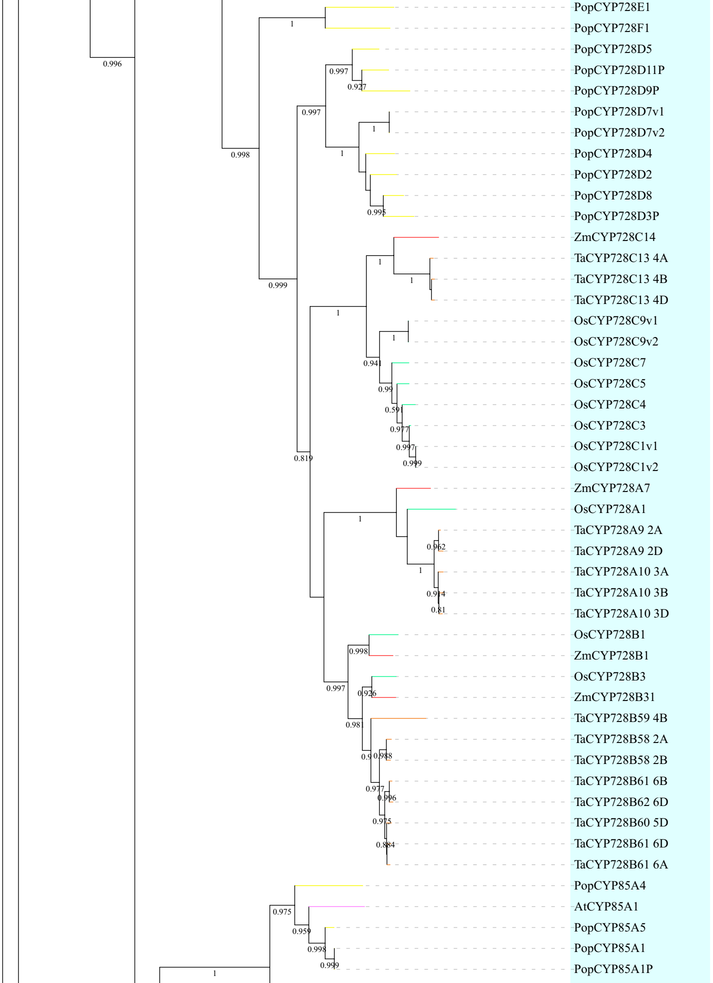
PopCYP728E1
PopCYP728F1
PopCYP728D5
PopCYP728D11P
PopCYP728D9P
PopCYP728D7v1
PopCYP728D7v2
PopCYP728D4
PopCYP728D2
PopCYP728D8
PopCYP728D3P
ZmCYP728C14
TaCYP728C13 4A
TaCYP728C13 4B
TaCYP728C13 4D
OsCYP728C9v1
OsCYP728C9v2
OsCYP728C7
OsCYP728C5
OsCYP728C4
OsCYP728C3
OsCYP728C1v1
OsCYP728C1v2
ZmCYP728A7
OsCYP728A1
TaCYP728A9 2A
TaCYP728A9 2D
TaCYP728A10 3A
TaCYP728A10 3B
TaCYP728A10 3D
OsCYP728B1
ZmCYP728B1
OsCYP728B3
ZmCYP728B31
TaCYP728B59 4B
TaCYP728B58 2A
TaCYP728B58 2B
TaCYP728B61 6B
TaCYP728B62 6D
TaCYP728B60 5D
TaCYP728B61 6D
TaCYP728B61 6A
PopCYP85A4
AtCYP85A1
PopCYP85A5
PopCYP85A1
PopCYP85A1P

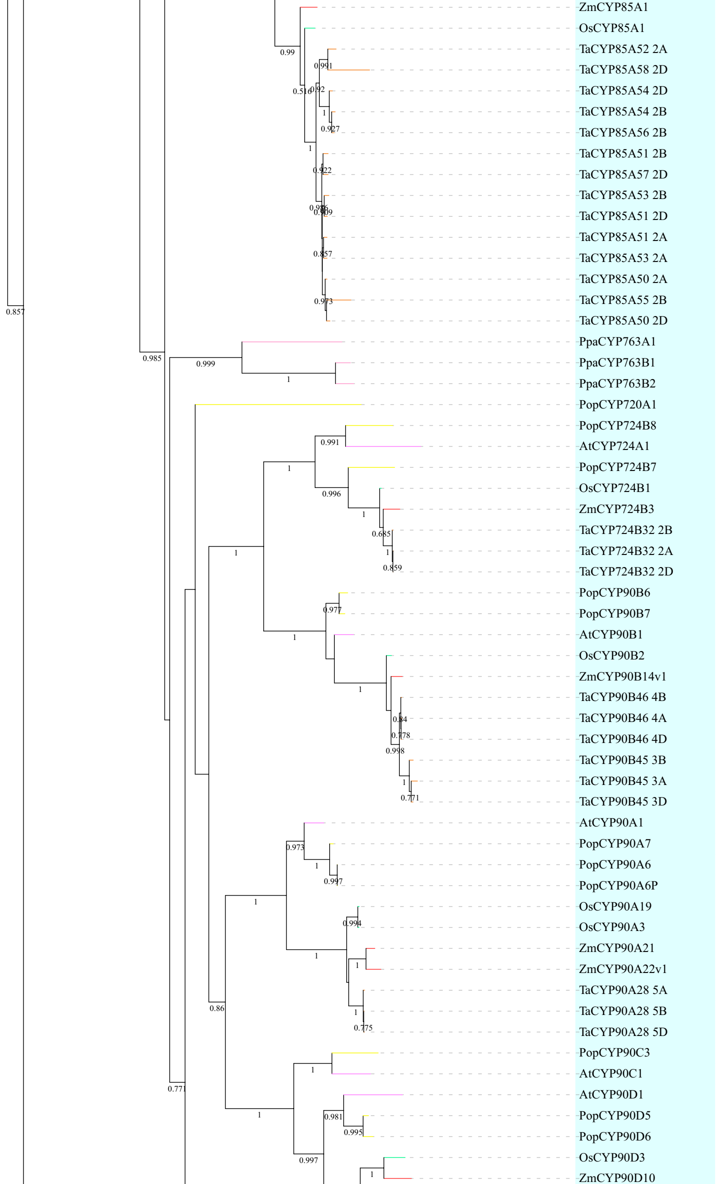
ZmCYP85A1
OsCYP85A1
TaCYP85A52 2A
TaCYP85A58 2D
TaCYP85A54 2D
TaCYP85A54 2B
TaCYP85A56 2B
TaCYP85A51 2B
TaCYP85A57 2D
TaCYP85A53 2B
TaCYP85A51 2D
TaCYP85A51 2A
TaCYP85A53 2A
TaCYP85A50 2A
TaCYP85A55 2B
TaCYP85A50 2D
PpaCYP763A1
PpaCYP763B1
PpaCYP763B2
PopCYP720A1
PopCYP724B8
AtCYP724A1
PopCYP724B7
OsCYP724B1
ZmCYP724B3
TaCYP724B32 2B
TaCYP724B32 2A
TaCYP724B32 2D
PopCYP90B6
PopCYP90B7
AtCYP90B1
OsCYP90B2
ZmCYP90B14v1
TaCYP90B46 4B
TaCYP90B46 4A
TaCYP90B46 4D
TaCYP90B45 3B
TaCYP90B45 3A
TaCYP90B45 3D
AtCYP90A1
PopCYP90A7
PopCYP90A6
PopCYP90A6P
OsCYP90A19
OsCYP90A3
ZmCYP90A21
ZmCYP90A22v1
TaCYP90A28 5A
TaCYP90A28 5B
TaCYP90A28 5D
PopCYP90C3
AtCYP90C1
AtCYP90D1
PopCYP90D5
PopCYP90D6
OsCYP90D3
ZmCYP90D10
0.99
0.991
0.516
0.92
0.927
0.922
0.857
0.973
0.857
0.985
0.999
0.991
0.996
0.685
0.859
0.977
0.84
0.778
0.998
0.771
0.973
0.997
0.994
0.775
0.86
0.771
0.981
0.995
0.997

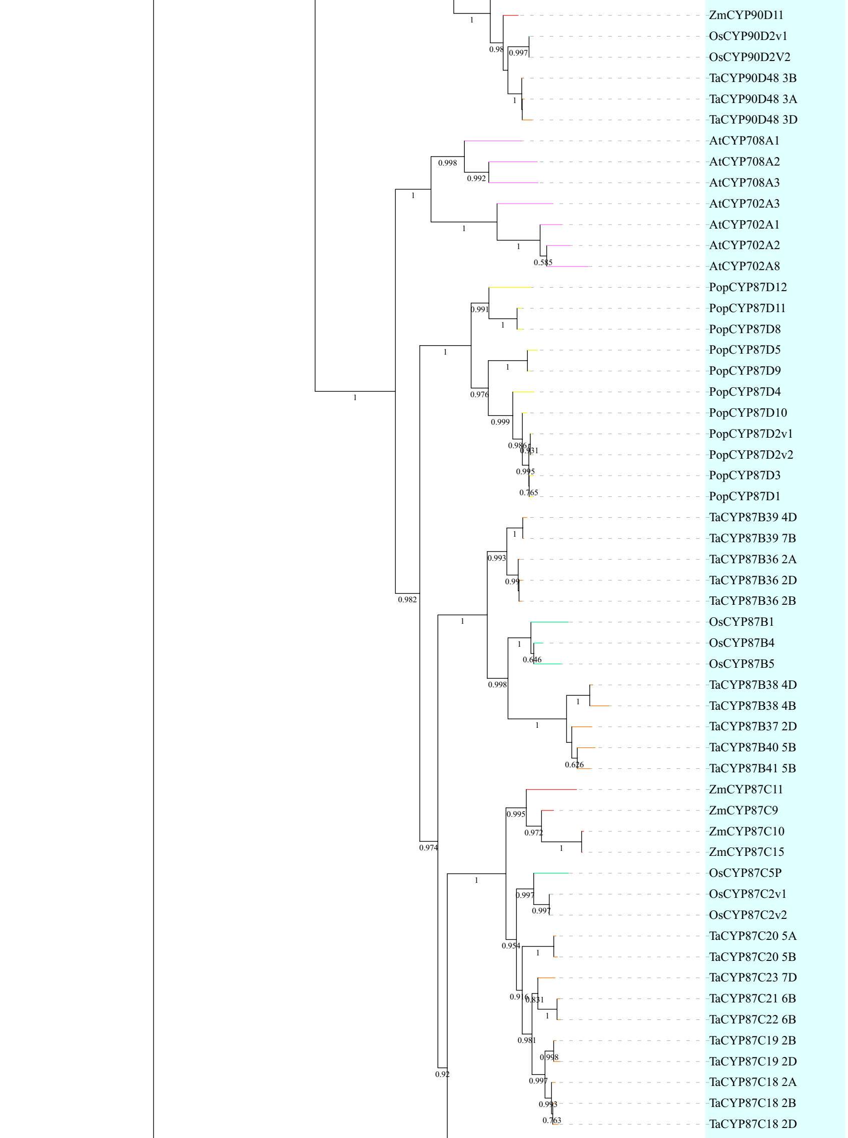
ZmCYP90D11
OsCYP90D2v1
OsCYP90D2V2
TaCYP90D48 3B
TaCYP90D48 3A
TaCYP90D48 3D
AtCYP708A1
AtCYP708A2
AtCYP708A3
AtCYP702A3
AtCYP702A1
AtCYP702A2
AtCYP702A8
PopCYP87D12
PopCYP87D11
PopCYP87D8
PopCYP87D5
PopCYP87D9
PopCYP87D4
PopCYP87D10
PopCYP87D2v1
PopCYP87D2v2
PopCYP87D3
PopCYP87D1
TaCYP87B39 4D
TaCYP87B39 7B
TaCYP87B36 2A
TaCYP87B36 2D
TaCYP87B36 2B
OsCYP87B1
OsCYP87B4
OsCYP87B5
TaCYP87B38 4D
TaCYP87B38 4B
TaCYP87B37 2D
TaCYP87B40 5B
TaCYP87B41 5B
ZmCYP87C11
ZmCYP87C9
ZmCYP87C10
ZmCYP87C15
OsCYP87C5P
OsCYP87C2v1
OsCYP87C2v2
TaCYP87C20 5A
TaCYP87C20 5B
TaCYP87C23 7D
TaCYP87C21 6B
TaCYP87C22 6B
TaCYP87C19 2B
TaCYP87C19 2D
TaCYP87C18 2A
TaCYP87C18 2B
TaCYP87C18 2D

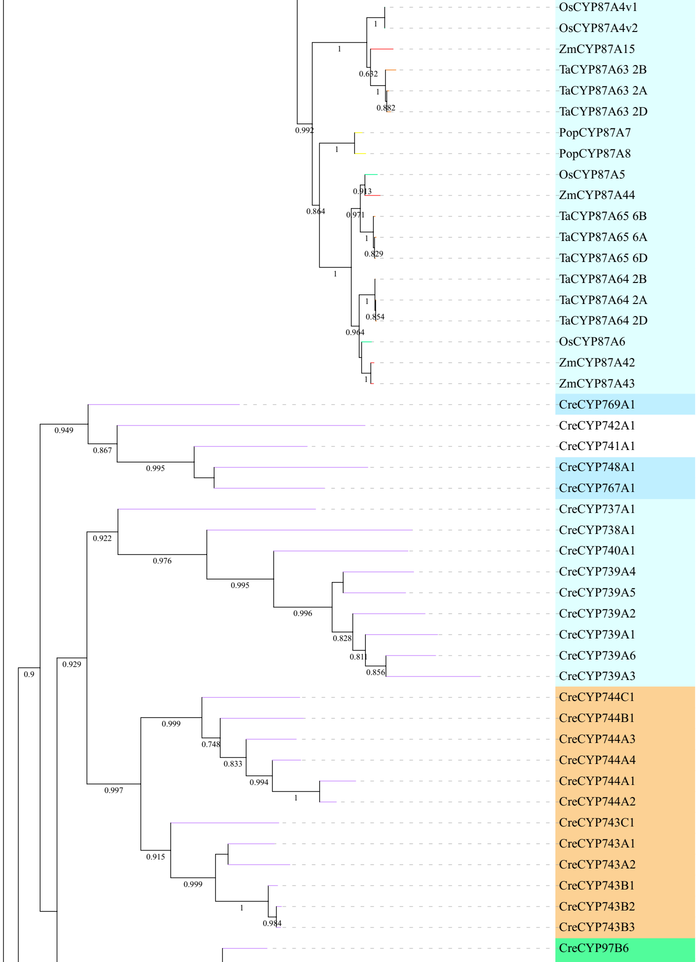
OsCYP87A4v1
OsCYP87A4v2
ZmCYP87A15
TaCYP87A63 2B
TaCYP87A63 2A
TaCYP87A63 2D
PopCYP87A7
PopCYP87A8
OsCYP87A5
ZmCYP87A44
TaCYP87A65 6B
TaCYP87A65 6A
TaCYP87A65 6D
TaCYP87A64 2B
TaCYP87A64 2A
TaCYP87A64 2D
OsCYP87A6
ZmCYP87A42
ZmCYP87A43
CreCYP769A1
CreCYP742A1
CreCYP741A1
CreCYP748A1
CreCYP767A1
CreCYP737A1
CreCYP738A1
CreCYP740A1
CreCYP739A4
CreCYP739A5
CreCYP739A2
CreCYP739A1
CreCYP739A6
CreCYP739A3
CreCYP744C1
CreCYP744B1
CreCYP744A3
CreCYP744A4
CreCYP744A1
CreCYP744A2
CreCYP743C1
CreCYP743A1
CreCYP743A2
CreCYP743B1
CreCYP743B2
CreCYP743B3
CreCYP97B6
1
1
0.632
1
0.882
0.992
1
0.913
0.864
0.971
1
0.829
1
1
0.854
0.964
1
0.949
0.867
0.995
0.922
0.976
0.995
0.996
0.828
0.811
0.856
0.929
0.9
0.999
0.748
0.833
0.994
1
0.997
0.915
0.999
1
0.984

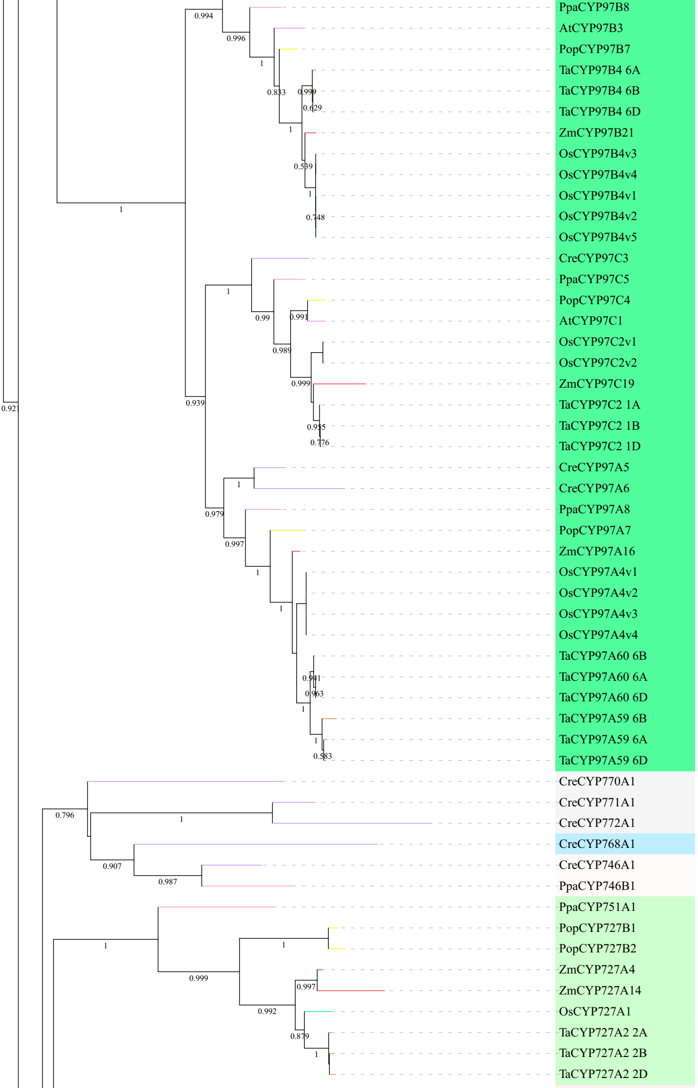
PpaCYP97B8
AtCYP97B3
PopCYP97B7
TaCYP97B4 6A
TaCYP97B4 6B
TaCYP97B4 6D
ZmCYP97B21
OsCYP97B4v3
OsCYP97B4v4
OsCYP97B4v1
OsCYP97B4v2
OsCYP97B4v5
CreCYP97C3
PpaCYP97C5
PopCYP97C4
AtCYP97C1
OsCYP97C2v1
OsCYP97C2v2
ZmCYP97C19
TaCYP97C2 1A
TaCYP97C2 1B
TaCYP97C2 1D
CreCYP97A5
CreCYP97A6
PpaCYP97A8
PopCYP97A7
ZmCYP97A16
OsCYP97A4v1
OsCYP97A4v2
OsCYP97A4v3
OsCYP97A4v4
TaCYP97A60 6B
TaCYP97A60 6A
TaCYP97A60 6D
TaCYP97A59 6B
TaCYP97A59 6A
TaCYP97A59 6D
CreCYP770A1
CreCYP771A1
CreCYP772A1
CreCYP768A1
CreCYP746A1
PpaCYP746B1
PpaCYP751A1
PopCYP727B1
PopCYP727B2
ZmCYP727A4
ZmCYP727A14
OsCYP727A1
TaCYP727A2 2A
TaCYP727A2 2B
TaCYP727A2 2D
0.994
0.996
1
0.833
0.999
0.629
1
0.539
1
0.748
1
1
0.99
0.991
0.989
0.999
0.955
0.776
0.939
0.921
1
0.979
0.997
1
1
0.941
0.963
1
1
0.583
0.796
1
0.907
0.987
1
0.999
1
0.992
0.997
0.879
1

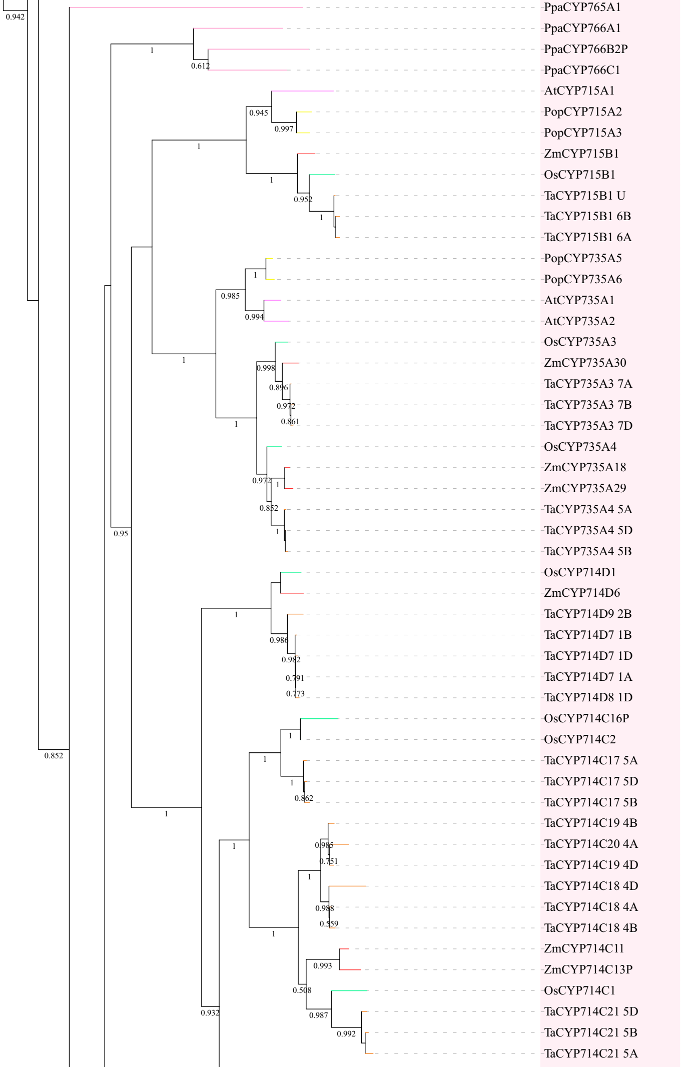

PpaCYP765A1
PpaCYP766A1
PpaCYP766B2P
PpaCYP766C1
AtCYP715A1
PopCYP715A2
PopCYP715A3
ZmCYP715B1
OsCYP715B1
TaCYP715B1 U
TaCYP715B1 6B
TaCYP715B1 6A
PopCYP735A5
PopCYP735A6
AtCYP735A1
AtCYP735A2
OsCYP735A3
ZmCYP735A30
TaCYP735A3 7A
TaCYP735A3 7B
TaCYP735A3 7D
OsCYP735A4
ZmCYP735A18
ZmCYP735A29
TaCYP735A4 5A
TaCYP735A4 5D
TaCYP735A4 5B
OsCYP714D1
ZmCYP714D6
TaCYP714D9 2B
TaCYP714D7 1B
TaCYP714D7 1D
TaCYP714D7 1A
TaCYP714D8 1D
OsCYP714C16P
OsCYP714C2
TaCYP714C17 5A
TaCYP714C17 5D
TaCYP714C17 5B
TaCYP714C19 4B
TaCYP714C20 4A
TaCYP714C19 4D
TaCYP714C18 4D
TaCYP714C18 4A
TaCYP714C18 4B
ZmCYP714C11
ZmCYP714C13P
OsCYP714C1
TaCYP714C21 5D
TaCYP714C21 5B
TaCYP714C21 5A

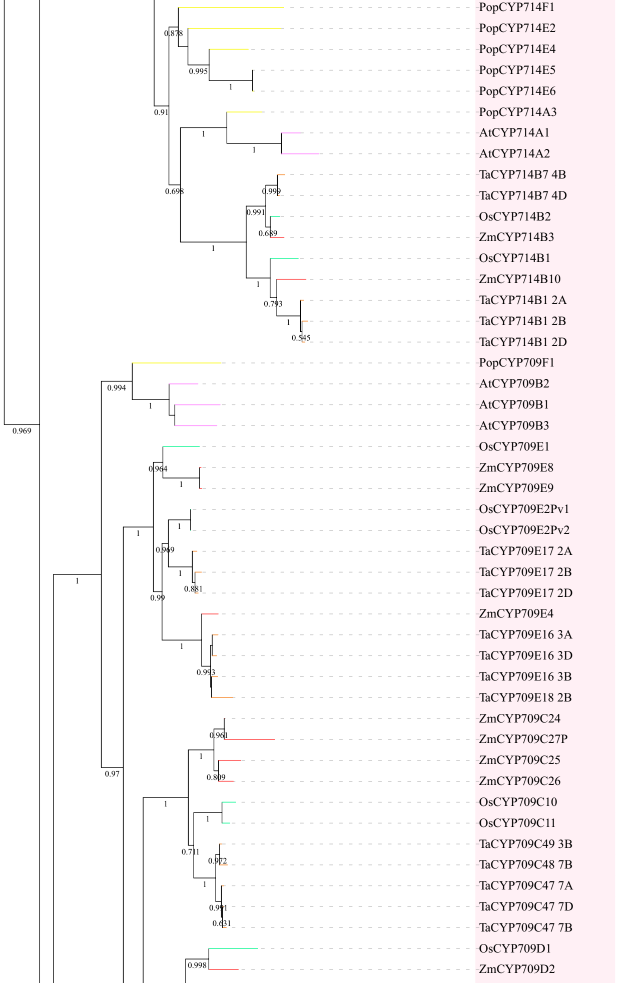
PopCYP714F1
PopCYP714E2
PopCYP714E4
PopCYP714E5
PopCYP714E6
PopCYP714A3
AtCYP714A1
AtCYP714A2
TaCYP714B7 4B
TaCYP714B7 4D
OsCYP714B2
ZmCYP714B3
OsCYP714B1
ZmCYP714B10
TaCYP714B1 2A
TaCYP714B1 2B
TaCYP714B1 2D
PopCYP709F1
AtCYP709B2
AtCYP709B1
AtCYP709B3
OsCYP709E1
ZmCYP709E8
ZmCYP709E9
OsCYP709E2Pv1
OsCYP709E2Pv2
TaCYP709E17 2A
TaCYP709E17 2B
TaCYP709E17 2D
ZmCYP709E4
TaCYP709E16 3A
TaCYP709E16 3D
TaCYP709E16 3B
TaCYP709E18 2B
ZmCYP709C24
ZmCYP709C27P
ZmCYP709C25
ZmCYP709C26
OsCYP709C10
OsCYP709C11
TaCYP709C49 3B
TaCYP709C48 7B
TaCYP709C47 7A
TaCYP709C47 7D
TaCYP709C47 7B
OsCYP709D1
ZmCYP709D2
0.878
0.995
1
0.91
1
1
0.698
0.999
0.991
0.689
1
1
0.793
1
0.545
0.994
1
0.969
0.964
1
1
0.969
1
0.881
0.99
1
1
0.993
0.961
1
0.809
0.97
1
1
0.711
0.972
1
0.991
0.631
0.998

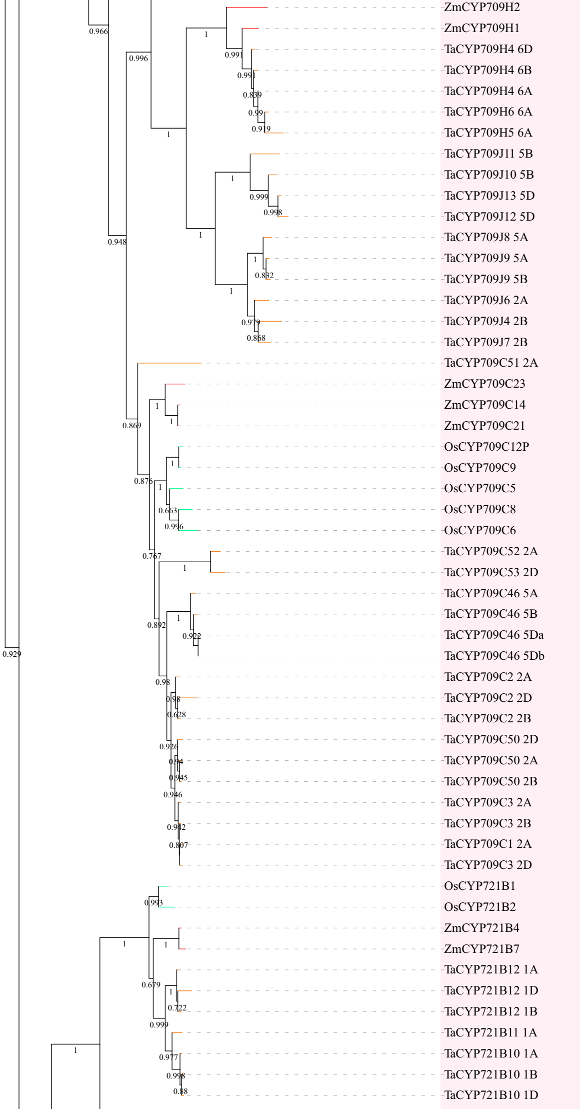
ZmCYP709H2
ZmCYP709H1
TaCYP709H4 6D
TaCYP709H4 6B
TaCYP709H4 6A
TaCYP709H6 6A
TaCYP709H5 6A
TaCYP709J11 5B
TaCYP709J10 5B
TaCYP709J13 5D
TaCYP709J12 5D
TaCYP709J8 5A
TaCYP709J9 5A
TaCYP709J9 5B
TaCYP709J6 2A
TaCYP709J4 2B
TaCYP709J7 2B
TaCYP709C51 2A
ZmCYP709C23
ZmCYP709C14
ZmCYP709C21
OsCYP709C12P
OsCYP709C9
OsCYP709C5
OsCYP709C8
OsCYP709C6
TaCYP709C52 2A
TaCYP709C53 2D
TaCYP709C46 5A
TaCYP709C46 5B
TaCYP709C46 5Da
TaCYP709C46 5Db
TaCYP709C2 2A
TaCYP709C2 2D
TaCYP709C2 2B
TaCYP709C50 2D
TaCYP709C50 2A
TaCYP709C50 2B
TaCYP709C3 2A
TaCYP709C3 2B
TaCYP709C1 2A
TaCYP709C3 2D
OsCYP721B1
OsCYP721B2
ZmCYP721B4
ZmCYP721B7
TaCYP721B12 1A
TaCYP721B12 1D
TaCYP721B12 1B
TaCYP721B11 1A
TaCYP721B10 1A
TaCYP721B10 1B
TaCYP721B10 1D
0.966
1
0.996
0.991
0.991
0.839
0.99
0.919
1
1
0.999
0.998
1
0.948
1
0.832
1
0.979
0.868
0.869
1
1
1
0.876
1
0.663
0.996
0.767
1
1
0.892
0.922
0.929
0.98
0.98
0.628
0.926
0.94
0.945
0.946
0.942
0.807
0.993
1
1
0.679
1
0.722
0.999
1
0.977
0.998
0.88

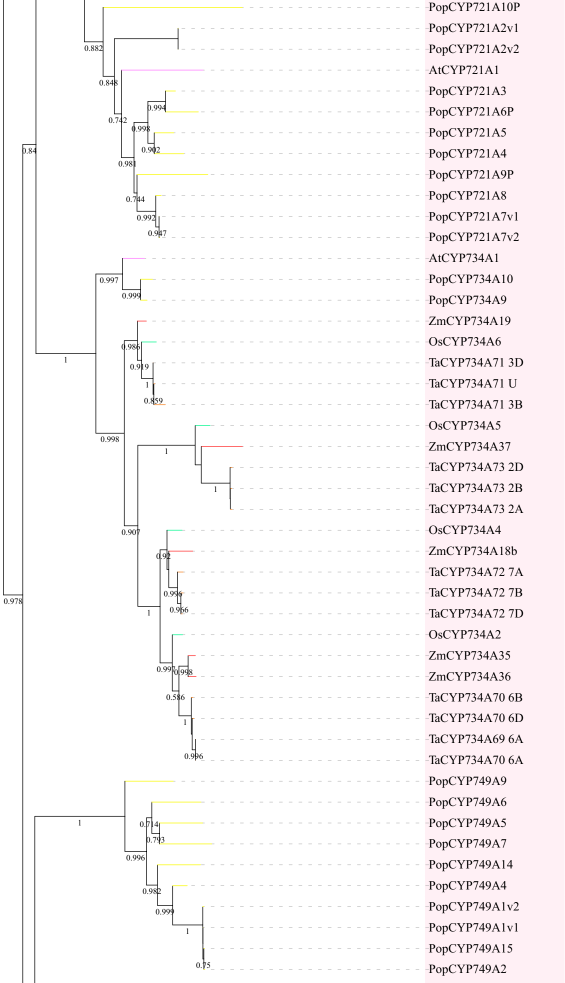
PopCYP721A10P
PopCYP721A2v1
PopCYP721A2v2
AtCYP721A1
PopCYP721A3
PopCYP721A6P
PopCYP721A5
PopCYP721A4
PopCYP721A9P
PopCYP721A8
PopCYP721A7v1
PopCYP721A7v2
AtCYP734A1
PopCYP734A10
PopCYP734A9
ZmCYP734A19
OsCYP734A6
TaCYP734A71 3D
TaCYP734A71 U
TaCYP734A71 3B
OsCYP734A5
ZmCYP734A37
TaCYP734A73 2D
TaCYP734A73 2B
TaCYP734A73 2A
OsCYP734A4
ZmCYP734A18b
TaCYP734A72 7A
TaCYP734A72 7B
TaCYP734A72 7D
OsCYP734A2
ZmCYP734A35
ZmCYP734A36
TaCYP734A70 6B
TaCYP734A70 6D
TaCYP734A69 6A
TaCYP734A70 6A
PopCYP749A9
PopCYP749A6
PopCYP749A5
PopCYP749A7
PopCYP749A14
PopCYP749A4
PopCYP749A1v2
PopCYP749A1v1
PopCYP749A15
PopCYP749A2
0.882
0.848
0.994
0.742
0.998
0.84
0.902
0.981
0.744
0.992
0.947
0.997
0.999
0.986
1
0.919
1
0.859
0.998
1
1
0.907
0.92
0.996
0.966
0.978
1
0.997
0.998
0.586
1
0.996
1
0.714
0.793
0.996
0.982
0.999
1
0.75

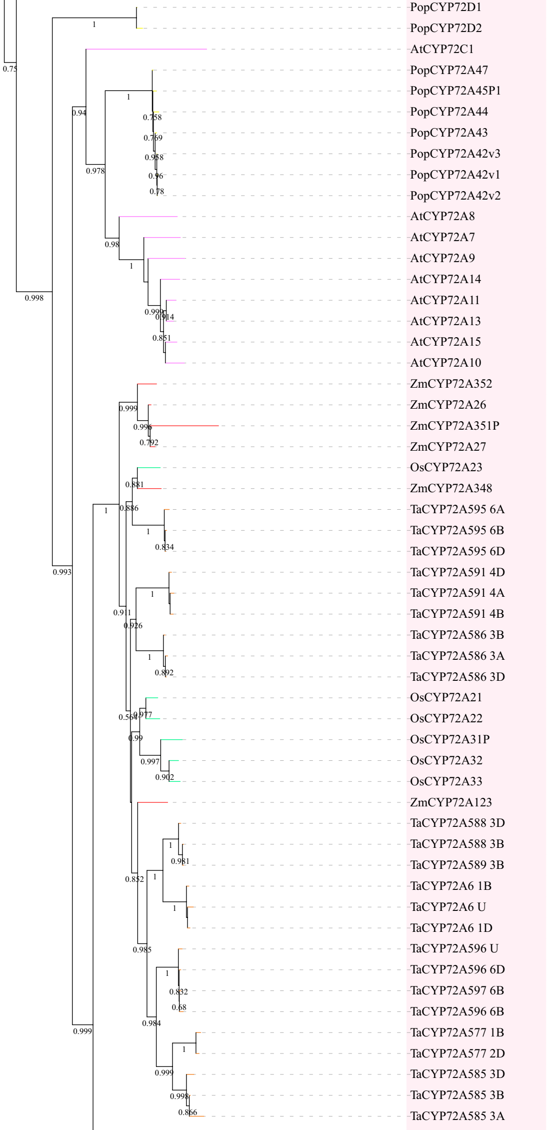
PopCYP72D1
PopCYP72D2
AtCYP72C1
PopCYP72A47
PopCYP72A45P1
PopCYP72A44
PopCYP72A43
PopCYP72A42v3
PopCYP72A42v1
PopCYP72A42v2
AtCYP72A8
AtCYP72A7
AtCYP72A9
AtCYP72A14
AtCYP72A11
AtCYP72A13
AtCYP72A15
AtCYP72A10
ZmCYP72A352
ZmCYP72A26
ZmCYP72A351P
ZmCYP72A27
OsCYP72A23
ZmCYP72A348
TaCYP72A595 6A
TaCYP72A595 6B
TaCYP72A595 6D
TaCYP72A591 4D
TaCYP72A591 4A
TaCYP72A591 4B
TaCYP72A586 3B
TaCYP72A586 3A
TaCYP72A586 3D
OsCYP72A21
OsCYP72A22
OsCYP72A31P
OsCYP72A32
OsCYP72A33
ZmCYP72A123
TaCYP72A588 3D
TaCYP72A588 3B
TaCYP72A589 3B
TaCYP72A6 1B
TaCYP72A6 U
TaCYP72A6 1D
TaCYP72A596 U
TaCYP72A596 6D
TaCYP72A597 6B
TaCYP72A596 6B
TaCYP72A577 1B
TaCYP72A577 2D
TaCYP72A585 3D
TaCYP72A585 3B
TaCYP72A585 3A

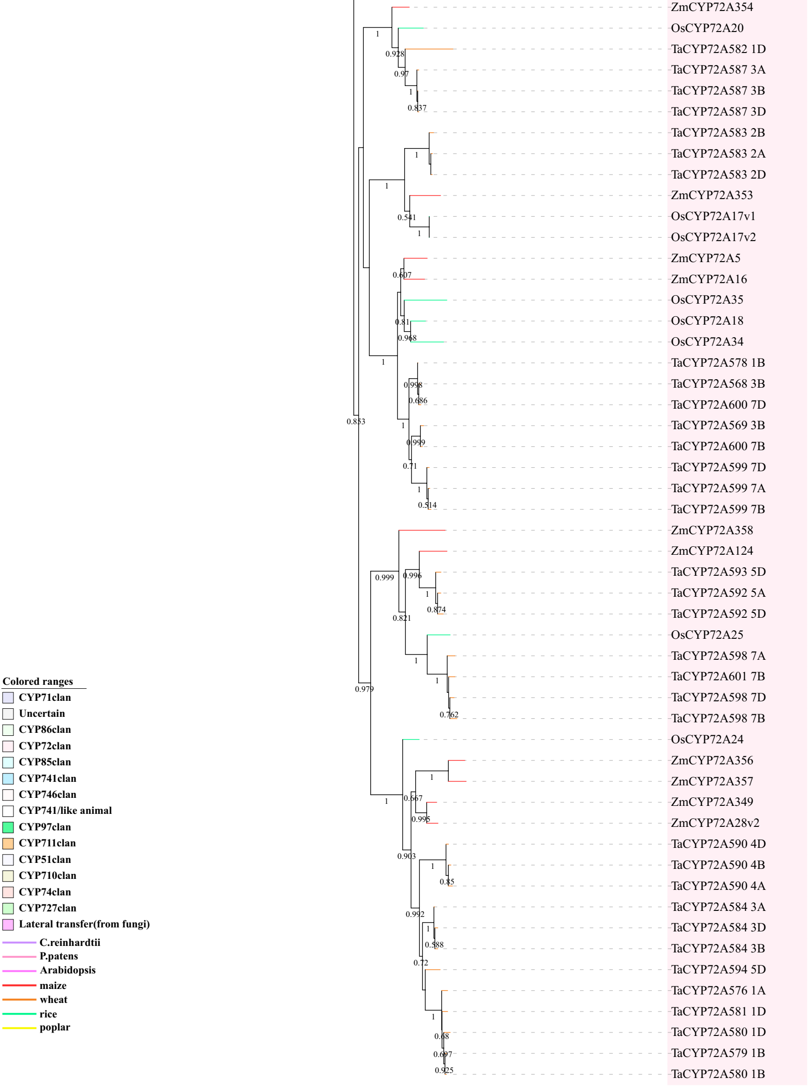

Figure S6. Phylogeny of CYP450s from C.reinhardtii, P.patens, poplar, Arabidopsis, rice, wheat and maize.
