# Supplementary material for: Comparative functional genomics analysis of cytochrome P450 gene superfamily in wheat and maize
Source: BMC Plant Biol. 2020 Mar 2;20:93. doi: 10.1186/s12870-020-2288-7 (PMC7052972; doi:10.1186/s12870-020-2288-7)
Supplement: Supplementary file 9 — Additional file 9: Figure S7. Gene structure of TaCYP450s. [file 12870_2020_2288_MOESM9_ESM.pdf]

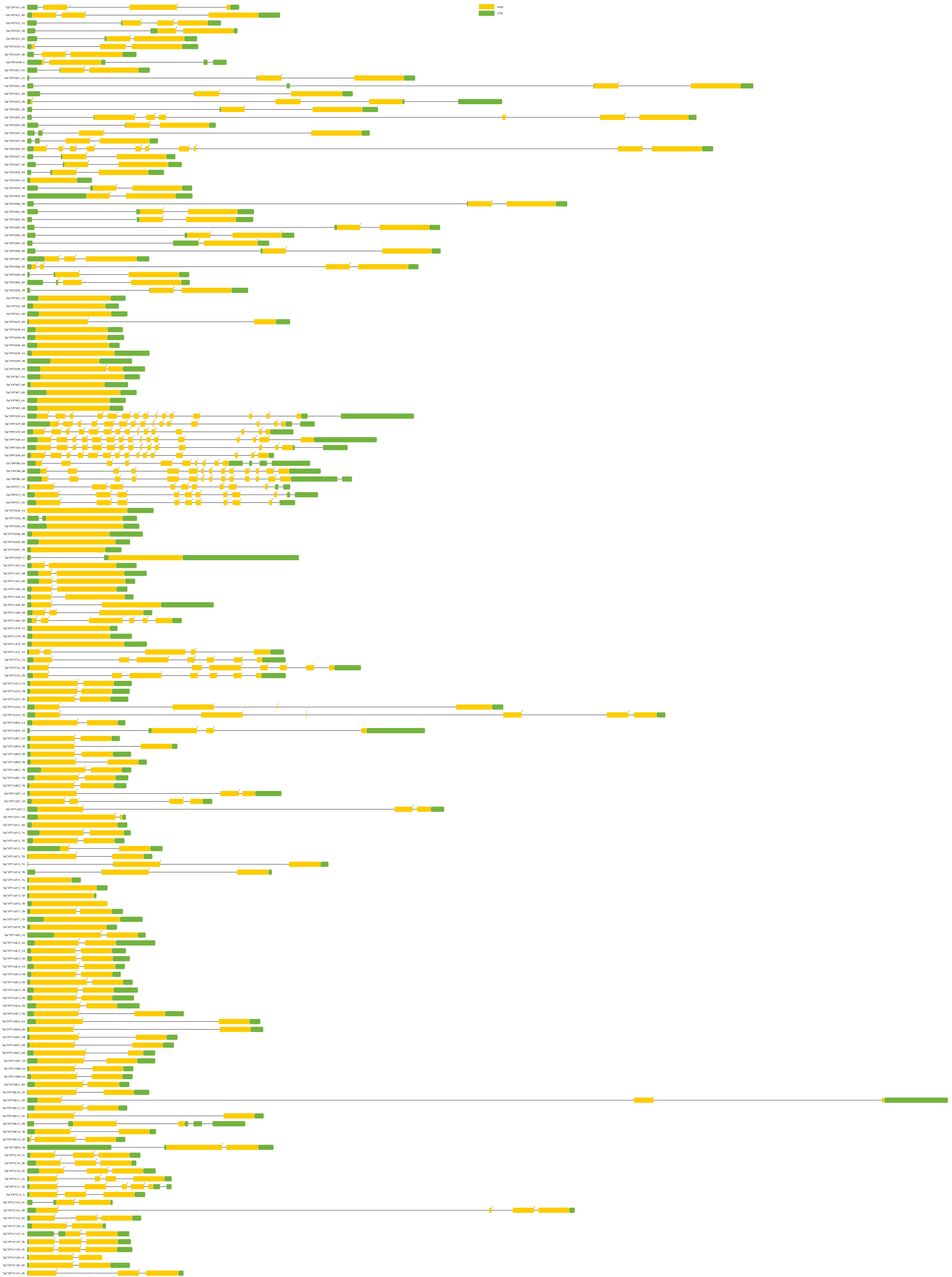

exon
UTR

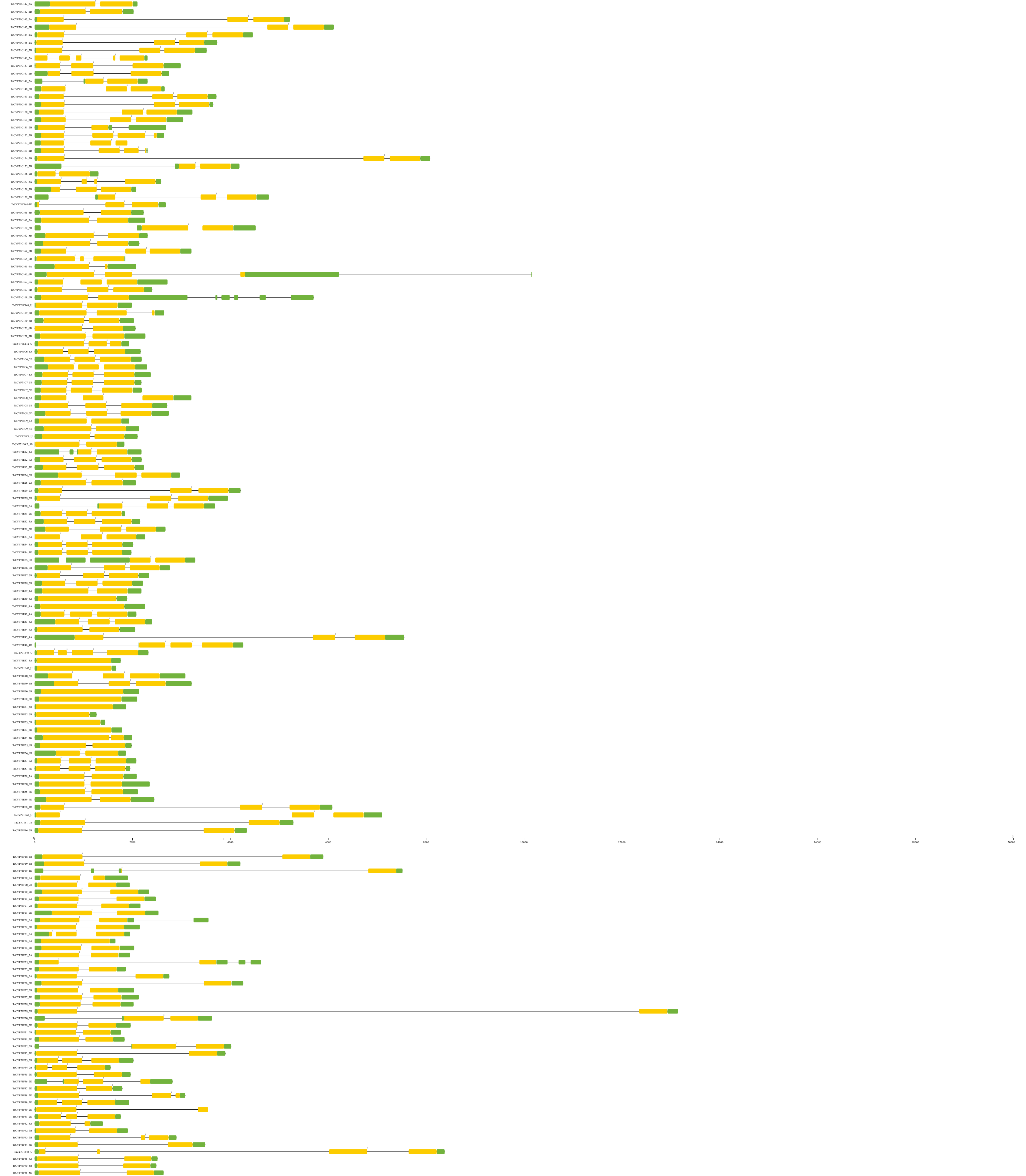
0
2000
4000
6000
8000
10000
12000
14000
16000
18000
20000

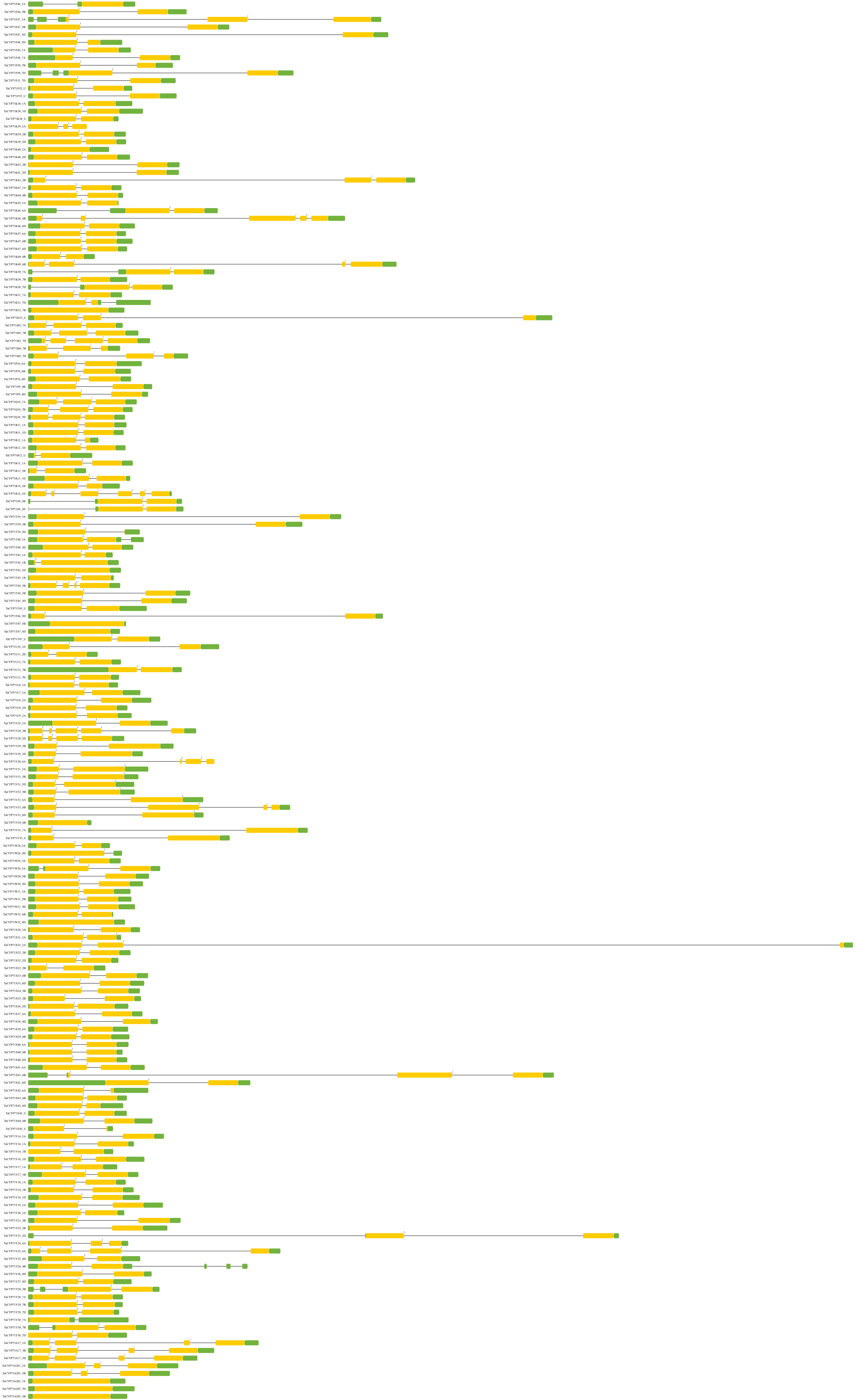
TaCYP71F46_5A
TaCYP71F46_5B
TaCYP71F47_5A
TaCYP71F47_5B
TaCYP71F47_5D
TaCYP71F48_5D
TaCYP71F49_7A
TaCYP71F50_7A
TaCYP71F50_7B
TaCYP71F50_7D
TaCYP71F51_7D
TaCYP71F52_U
TaCYP71F53_U
TaCYP71K38_1A
TaCYP71K38_1D
TaCYP71K38_U
TaCYP71K39_2A
TaCYP71K39_2B
TaCYP71K39_2D
TaCYP71K40_2A
TaCYP71K40_2D
TaCYP71K41_2B
TaCYP71K41_2D
TaCYP71K42_2B
TaCYP71K43_3A
TaCYP71K44_4B
TaCYP71K45_5A
TaCYP71K46_6A
TaCYP71K46_6B
TaCYP71K46_6D
TaCYP71K47_6A
TaCYP71K47_6B
TaCYP71K47_6D
TaCYP71K48_6B
TaCYP71K49_6B
TaCYP71K50_7A
TaCYP71K50_7B
TaCYP71K50_7D
TaCYP71K51_7A
TaCYP71K51_7D
TaCYP71K52_7B
TaCYP71K53_U
TaCYP71M3_7A
TaCYP71M3_7B
TaCYP71M3_7D
TaCYP71M4_7B
TaCYP71M5_7D
TaCYP71P10_6A
TaCYP71P10_6B
TaCYP71P10_6D
TaCYP71P9_4B
TaCYP71P9_4D
TaCYP71Q10_7A
TaCYP71Q10_7B
TaCYP71Q10_7D
TaCYP71R11_1A
TaCYP71R11_1D
TaCYP71R12_1A
TaCYP71R12_1D
TaCYP71R12_U
TaCYP71R13_1A
TaCYP71R13_1B
TaCYP71R13_1D
TaCYP71R14_1B
TaCYP71R14_1D
TaCYP71S9_2B
TaCYP71S9_2D
TaCYP71T39_3A
TaCYP71T39_3B
TaCYP71T39_3D
TaCYP71T40_3A
TaCYP71T40_3D
TaCYP71T42_1A
TaCYP71T42_1B
TaCYP71T42_1D
TaCYP71T43_1B
TaCYP71T44_3B
TaCYP71T45_3B
TaCYP71T45_3D
TaCYP71T45_U
TaCYP71T46_3D
TaCYP71T47_5B
TaCYP71T47_5D
TaCYP71T47_U
TaCYP71U10_2A
TaCYP71U11_2D
TaCYP71U12_7A
TaCYP71U12_7B
TaCYP71U12_7D
TaCYP71U6_3A
TaCYP71U7_2A
TaCYP71U8_2A
TaCYP71U8_2D
TaCYP71U9_2A
TaCYP71V25_3A
TaCYP71V28_2B
TaCYP71V28_2D
TaCYP71V29_3B
TaCYP71V29_3D
TaCYP71V30_4A
TaCYP71V31_5A
TaCYP71V31_5B
TaCYP71V31_5D
TaCYP71V32_5B
TaCYP71V33_6A
TaCYP71V33_6B
TaCYP71V33_6D
TaCYP71V34_6B
TaCYP71V35_7A
TaCYP71V35_U
TaCYP71W28_2A
TaCYP71W28_2D
TaCYP71W29_5A
TaCYP71W30_5A
TaCYP71W30_5B
TaCYP71W30_5D
TaCYP71W31_5A
TaCYP71W31_5B
TaCYP71W31_5D
TaCYP71W32_6B
TaCYP71W32_6D
TaCYP71X30_1D
TaCYP71X31_2A
TaCYP71X32_2A
TaCYP71X32_2B
TaCYP71X32_2D
TaCYP71X33_2B
TaCYP71X33_6B
TaCYP71X33_6D
TaCYP71X34_2B
TaCYP71X35_2B
TaCYP71X36_2D
TaCYP71X37_4A
TaCYP71X38_4D
TaCYP71X39_6A
TaCYP71X39_6B
TaCYP71X40_6A
TaCYP71X40_6B
TaCYP71X40_6D
TaCYP71X41_6A
TaCYP71X41_6B
TaCYP71X41_6D
TaCYP71X42_6A
TaCYP71X43_6B
TaCYP71X43_6D
TaCYP71X43_U
TaCYP71X44_6B
TaCYP71X45_U
TaCYP71Y14_3A
TaCYP71Y16_1A
TaCYP71Y16_1B
TaCYP71Y16_1D
TaCYP71Y17_1A
TaCYP71Y17_1B
TaCYP71Y18_1A
TaCYP71Y18_1B
TaCYP71Y18_1D
TaCYP71Y19_2A
TaCYP71Y20_2A
TaCYP71Y21_2B
TaCYP71Y22_3B
TaCYP71Y23_3D
TaCYP71Y24_4A
TaCYP71Y25_4A
TaCYP71Y25_4D
TaCYP71Y26_4B
TaCYP71Y26_4D
TaCYP71Y27_4D
TaCYP71Y28_5B
TaCYP71Y29_7A
TaCYP71Y29_7B
TaCYP71Y29_7D
TaCYP71Y30_7A
TaCYP71Y30_7B
TaCYP71Y30_7D
TaCYP73A17_3A
TaCYP73A17_3B
TaCYP73A17_3D
TaCYP73A201_3A
TaCYP73A201_3B
TaCYP73A202_7A
TaCYP73A202_7D
TaCYP73A203_2B

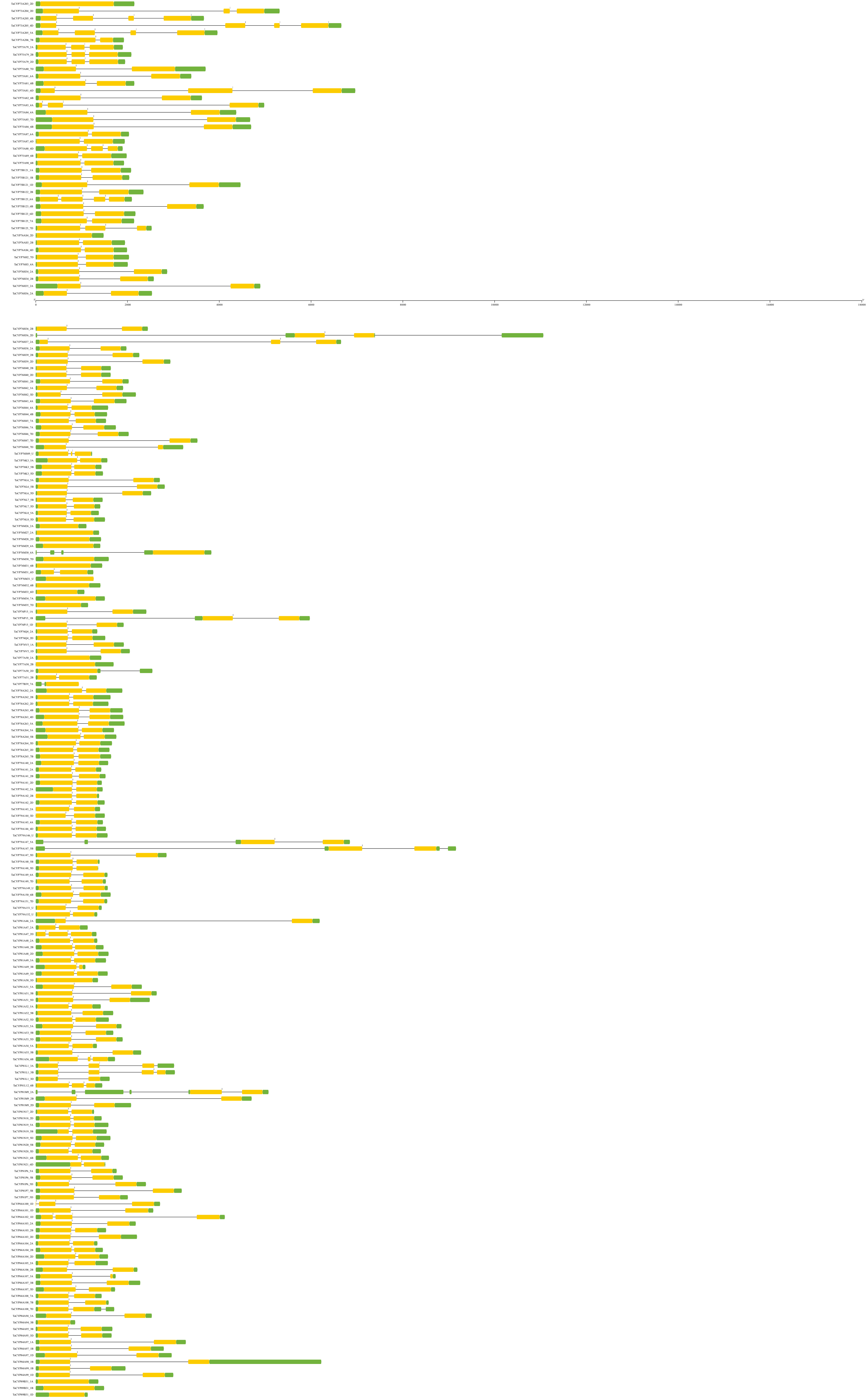
0
2000
4000
6000
8000
10000
12000
14000
16000
18000

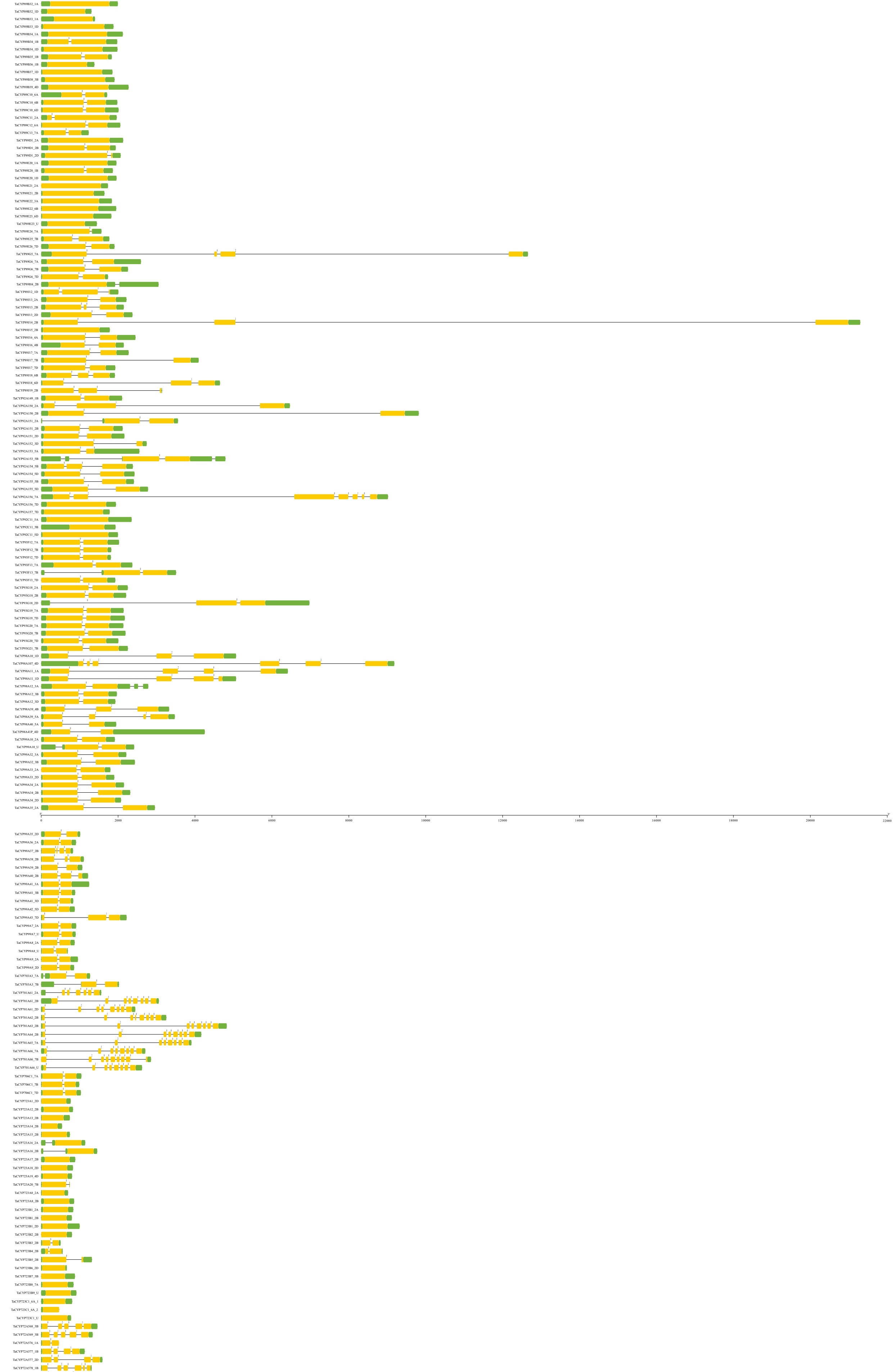
0
2000
4000
6000
8000
10000
12000
14000
16000
18000
20000
22000

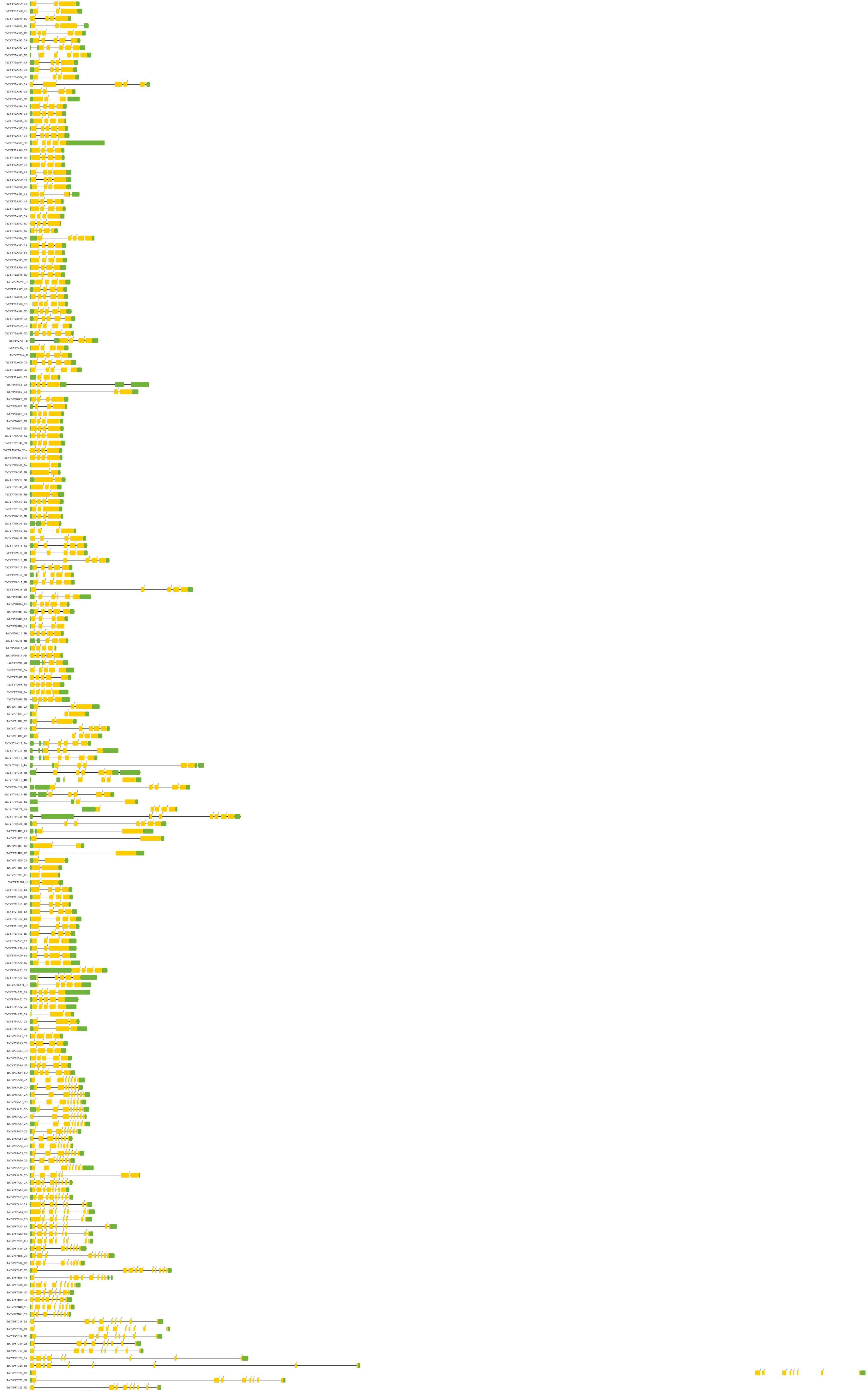

TaCYP72A579_1B
TaCYP72A580_1B
TaCYP72A580_1D
TaCYP72A581_1D
TaCYP72A582_1D
TaCYP72A583_2A
TaCYP72A583_2B
TaCYP72A583_2D
TaCYP72A584_3A
TaCYP72A584_3B
TaCYP72A584_3D
TaCYP72A585_3A
TaCYP72A585_3B
TaCYP72A585_3D
TaCYP72A586_3A
TaCYP72A586_3B
TaCYP72A586_3D
TaCYP72A587_3A
TaCYP72A587_3B
TaCYP72A587_3D
TaCYP72A588_3B
TaCYP72A588_3D
TaCYP72A589_3B
TaCYP72A590_4A
TaCYP72A590_4B
TaCYP72A590_4D
TaCYP72A591_4A
TaCYP72A591_4B
TaCYP72A591_4D
TaCYP72A592_5A
TaCYP72A592_5D
TaCYP72A593_5D
TaCYP72A594_5D
TaCYP72A595_6A
TaCYP72A595_6B
TaCYP72A595_6D
TaCYP72A596_6B
TaCYP72A596_6D
TaCYP72A596_U
TaCYP72A597_6B
TaCYP72A598_7A
TaCYP72A598_7B
TaCYP72A598_7D
TaCYP72A599_7A
TaCYP72A599_7B
TaCYP72A599_7D
TaCYP72A6_1B
TaCYP72A6_1D
TaCYP72A6_U
TaCYP72A600_7B
TaCYP72A600_7D
TaCYP72A601_7B
TaCYP709C1_2A
TaCYP709C2_2A
TaCYP709C2_2B
TaCYP709C2_2D
TaCYP709C3_2A
TaCYP709C3_2B
TaCYP709C3_2D
TaCYP709C46_5A
TaCYP709C46_5B
TaCYP709C46_5Da
TaCYP709C46_5Db
TaCYP709C47_7A
TaCYP709C47_7B
TaCYP709C47_7D
TaCYP709C48_7B
TaCYP709C49_3B
TaCYP709C50_2A
TaCYP709C50_2B
TaCYP709C50_2D
TaCYP709C51_2A
TaCYP709C52_2A
TaCYP709C53_2D
TaCYP709E16_3A
TaCYP709E16_3B
TaCYP709E16_3D
TaCYP709E17_2A
TaCYP709E17_2B
TaCYP709E17_2D
TaCYP709E18_2B
TaCYP709H4_6A
TaCYP709H4_6B
TaCYP709H4_6D
TaCYP709H5_6A
TaCYP709H6_6A
TaCYP709J10_5B
TaCYP709J11_5B
TaCYP709J12_5D
TaCYP709J13_5D
TaCYP709J4_2B
TaCYP709J6_2A
TaCYP709J7_2B
TaCYP709J8_5A
TaCYP709J9_5A
TaCYP709J9_5B
TaCYP714B1_2A
TaCYP714B1_2B
TaCYP714B1_2D
TaCYP714B7_4B
TaCYP714B7_4D
TaCYP714C17_5A
TaCYP714C17_5B
TaCYP714C17_5D
TaCYP714C18_4A
TaCYP714C18_4B
TaCYP714C18_4D
TaCYP714C19_4B
TaCYP714C19_4D
TaCYP714C20_4A
TaCYP714C21_5A
TaCYP714C21_5B
TaCYP714C21_5D
TaCYP714D7_1A
TaCYP714D7_1B
TaCYP714D7_1D
TaCYP714D8_1D
TaCYP714D9_2B
TaCYP715B1_6A
TaCYP715B1_6B
TaCYP715B1_U
TaCYP721B10_1A
TaCYP721B10_1B
TaCYP721B10_1D
TaCYP721B11_1A
TaCYP721B12_1A
TaCYP721B12_1B
TaCYP721B12_1D
TaCYP734A69_6A
TaCYP734A70_6A
TaCYP734A70_6B
TaCYP734A70_6D
TaCYP734A71_3B
TaCYP734A71_3D
TaCYP734A71_U
TaCYP734A72_7A
TaCYP734A72_7B
TaCYP734A72_7D
TaCYP734A73_2A
TaCYP734A73_2B
TaCYP734A73_2D
TaCYP735A3_7A
TaCYP735A3_7B
TaCYP735A3_7D
TaCYP735A4_5A
TaCYP735A4_5B
TaCYP735A4_5D
TaCYP85A50_2A
TaCYP85A50_2D
TaCYP85A51_2A
TaCYP85A51_2B
TaCYP85A51_2D
TaCYP85A52_2A
TaCYP85A53_2A
TaCYP85A53_2B
TaCYP85A54_2B
TaCYP85A54_2D
TaCYP85A55_2B
TaCYP85A56_2B
TaCYP85A57_2D
TaCYP85A58_2D
TaCYP87A63_2A
TaCYP87A63_2B
TaCYP87A63_2D
TaCYP87A64_2A
TaCYP87A64_2B
TaCYP87A64_2D
TaCYP87A65_6A
TaCYP87A65_6B
TaCYP87A65_6D
TaCYP87B36_2A
TaCYP87B36_2B
TaCYP87B36_2D
TaCYP87B37_2D
TaCYP87B38_4B
TaCYP87B38_4D
TaCYP87B39_4D
TaCYP87B39_7B
TaCYP87B40_5B
TaCYP87B41_5B
TaCYP87C18_2A
TaCYP87C18_2B
TaCYP87C18_2D
TaCYP87C19_2B
TaCYP87C19_2D
TaCYP87C20_5A
TaCYP87C20_5B
TaCYP87C21_6B
TaCYP87C22_6B
TaCYP87C23_7D

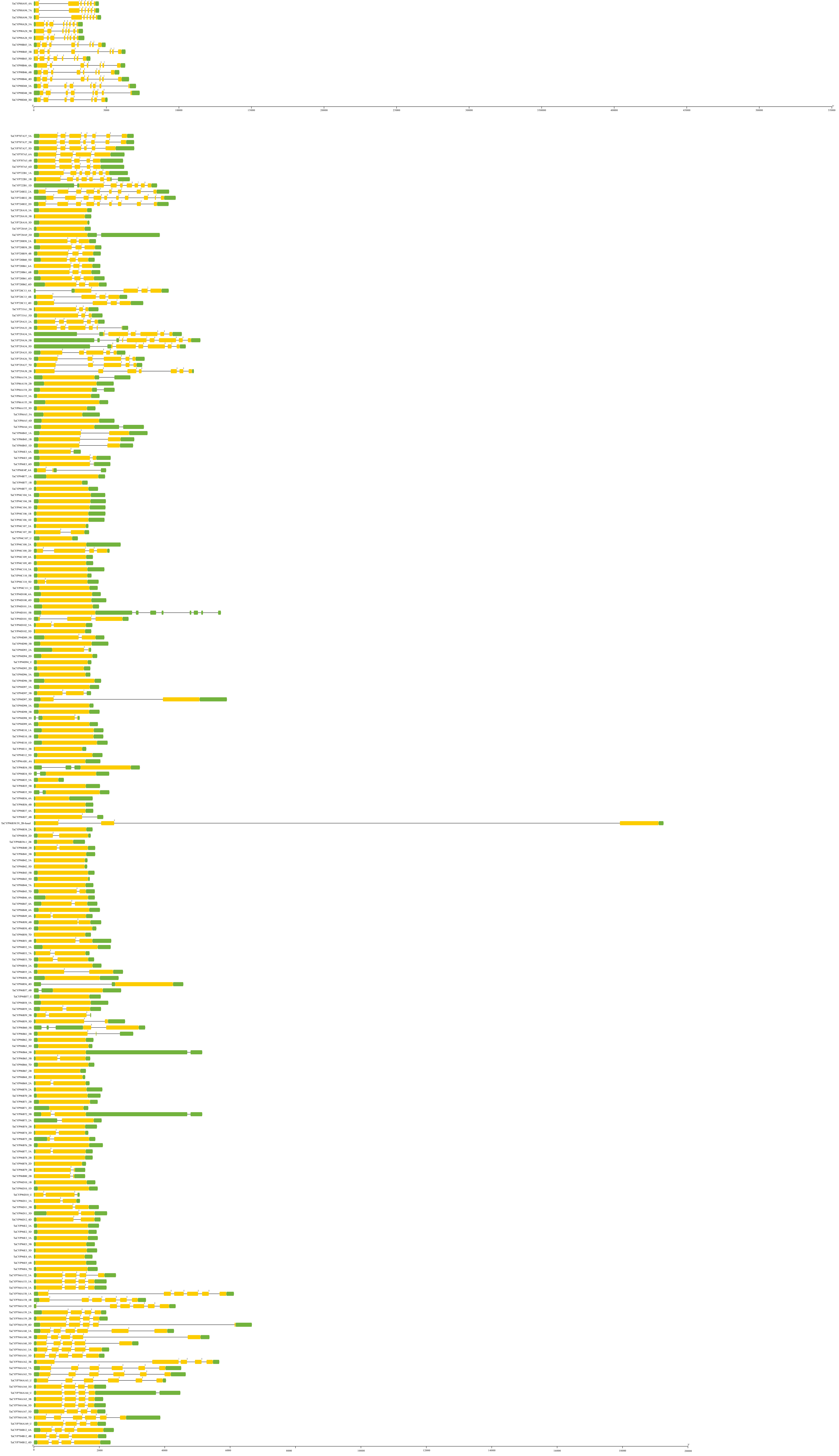

Figure S7. Gene structure of TaCYP450s.
