# Supplementary material for: Comparative functional genomics analysis of cytochrome P450 gene superfamily in wheat and maize
Source: BMC Plant Biol. 2020 Mar 2;20:93. doi: 10.1186/s12870-020-2288-7 (PMC7052972; doi:10.1186/s12870-020-2288-7)
Supplement: Supplementary file 16 — Additional file 16: Figure S12. The multiple sequence alignment of CYP97 members. Sites whose posterior probability is larger than cutoff value (0.67) are marked in star or triangle. [file 12870_2020_2288_MOESM16_ESM.pdf]

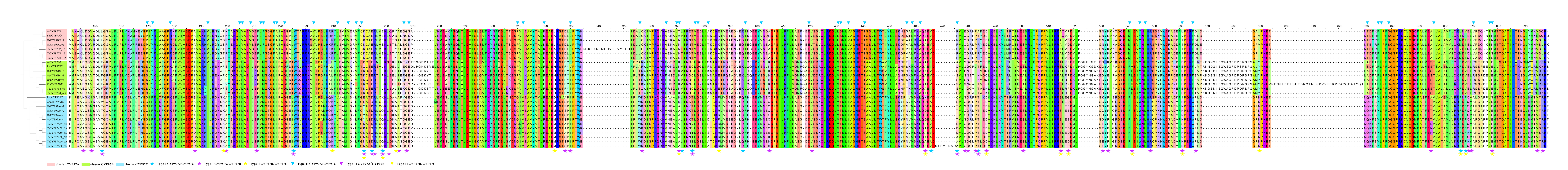

Figure S10. The multiple sequence alignment of CYP97 members. Sites whose posterior probability is larger than cutoff value (0.67) are marked in star or triangle.
